# Supplementary material for: A systematic review of trial registry entries for randomized clinical trials investigating COVID-19 medical prevention and treatment
Source: PLoS One. 2020 Aug 20;15(8):e0237903. doi: 10.1371/journal.pone.0237903 (PMC7444584; doi:10.1371/journal.pone.0237903)
Supplement: S1 Appendix — (PDF) [file pone.0237903.s001.pdf]

Appendix 1: Trial registration sites

Trials registration databases represented in the WHO COVID-19 data file

Australian New Zealand Clinical Trials Registry (ANZCTR)
Specific COVID-19 site available at the ANZCTR site
https://www.anzctr.org.au/TrialSearch.aspx#&&conditionCode=&dateOfRegistrationFrom=&interventionDescription=&interventionCodeOperator=OR&primarySponsorType=&gender=&distance=&postcode=&pageSize=20&ageGroup=&recruitmentCountryOperator=OR&recruitmentRegion=&ethicsReview=&countryOfRecruitment=Australia%7cNew+Zealand&registry=&searchTxt=COVID+19+OR+%22SARS-CoV2%22+OR+%222019-nCoV%22&studyType=&allocationToIntervention=&dateOfRegistrationTo=&recruitmentStatus=&interventionCode=&healthCondition=&healthyVolunteers=&page=1&conditionCategory=&fundingSource=&trialStartDateTo=&trialStartDateFrom=&phase=

Chinese Clinical Trial Registry (ChiCTR)
Specific COVID-19 site available at the ChiCTR site http://www.chictr.org.cn/enIndex.aspx

ClinicalTrials.gov
Specific COVID-19 site available at the clinicaltrials.gov site
https://clinicaltrials.gov/ct2/results?cond=COVID-19

EU Clinical Trials Register (EduraCT)
Specific COVID-19 site available at the EduraCT site https://www.clinicaltrialsregister.eu/ctr-search/search?query=covid-19

German Clinical Trials Register (DRKS)
DRKS site https://www.drks.de/drks_web/

Iranian Registry of Clinical Trials (IRCT)
IRCT site https://www.irct.ir

Japan Primary Registries Network (JPRN)
Several registries in this network (jRCT, UMIN-CTR, JMACCT, JAPIC). An overall search is available at the JPRM site https://rctportal.niph.go.jp/en/

Netherlands Trial Register (NTR)
NTR site https://www.trialregister.nl

Swiss National Clinical Trials Portal (SNCTP)
SNCTP site https://www.kofam.ch/en/snctp-portal/searching-for-a-clinical-trial/

Thai Clinical Trials Registry (TCTR)
TCTR site https://www.clinicaltrials.in.th

Trials registration databases not represented in the WHO COVID-19 data file

AMIS: drugs with approval since 2004 in Germany
AMIS site https://www.dimdi.de/dynamic/de/arzneimittel/arzneimittel-recherchieren/amis/

Be Part of Research
Be Part of Research site https://bepartofresearch.nihr.ac.uk

Brazilian Clinical Trials Registry / Registro Brasileiro de Ensaios Clinicos (ReBec)
ReBec site http://www.ensaiosclinicos.gov.br

Centerwatch
Centerwatch site **https://www.centerwatch.com**

China Drug Trials
This service is available in Chinese at site http://www.chinadrugtrials.org.cn/eap/main

ClinicalStudyDataRequest.com
Site https://clinicalstudydatarequest.com

Cuban Public Registry of Clinical Trials (RPCEC)
RPCEC site http://registroclinico.sld.cu/en/faq/¿What-Cuban-Public-Registry-Clinical-Trials

Hong Kong University Clinical Trial Register (HKUCTR)
HKUCTR site http://www.hkuctr.com/search

Clinical Trials Registry India (CTRI)
CTRI site http://ctri.nic.in

International Clinical Trials Registry Platform (WHO)
The portal provides access to 17 clinical trial registries at the WHO site
https://www.who.int/ictrp/network/factsheet/en/

International Standard Randomised Controlled Trial Number Register (ISRCTN)
ISRCTN site https://apps.who.int/trialsearch/

Korea, Republic of - Clinical Research Information Service (CRiS)
CRiS site https://cris.nih.go.kr/cris/en/

Medline Databank Sources
Medline site https://www.nlm.nih.gov/bsd/medline_databank_source.html

metaRegister of Controlled Trials
This service is currently under review

Pan African Clinical Trials Registry (PACTR)
PACTR site http://www.edctp.org/pan-african-clinical-trials-registry/

Peruvian Clinical Trials Registry (REPEC)
REPEC site https://ensayosclinicos-repec.ins.gob.pe/en/

PharmNet.Bund Clinical Trials
PharmNet.Bund site https://www.pharmnet-bund.de/static/en/clinical-trials/index.html

Research Registry
Research Registry site https://www.researchregistry.com

South African National Clinical Trials Register
South African National Clinical Trials Register site http://www.sanctr.gov.za

Sri Lanka Clinical Trials Registry (SLCTR)
SLCTR site https://slctr.lk

TrialExplorer
TrialExplorer site https://ctms.geminidsystems.com/dashboard

Yale University Open Data Access (YODA) project
YODA project site https://yoda.yale.edu/browsetrials/generic-name
