# Supplementary material for: A systematic review of trial registry entries for randomized clinical trials investigating COVID-19 medical prevention and treatment
Source: PLoS One. 2020 Aug 20;15(8):e0237903. doi: 10.1371/journal.pone.0237903 (PMC7444584; doi:10.1371/journal.pone.0237903)
Supplement: S2 Appendix — (PDF) [file pone.0237903.s002.pdf]

Appendix 2: Excluded trial registry entries

Registry entry ID	Title	Database ID (ID all)	Reason for exclusion
NCT04269525	Umbilical Cord(UC)-Derived Mesenchymal Stem Cells(MSCs) Treatment for the 2019-novel Coronavirus(nCOV) Pneumonia	7	Clinical, not randomised
NCT04257656	Severe 2019-nCoV Remdesivir RCT	10	Cancelled
NCT04252664	Mild/Moderate 2019-nCoV Remdesivir RCT	11	Cancelled
NCT04275245	Clinical Study of Anti-CD147 Humanized Meplazumab for Injection to Treat With 2019-nCoV Pneumonia	15	Clinical, not randomised
NCT04280588	Fingolimod in COVID-19	18	Cinical, not randomised
NCT04281693	A New Screening Strategy for 2019 Novel Coronavirus Infection	21	Clinical, not randomised
NCT04252118	Mesenchymal Stem Cell Treatment for Pneumonia Patients Infected With 2019 Novel Coronavirus	23	Cinical, not randomised
NCT04276987	A Pilot Clinical Study on Inhalation of Mesenchymal Stem Cells Exosomes Treating Severe Novel Coronavirus Pneumonia	24	Clinical, not randomised
NCT04285190	The Effect of T89 on Improving Oxygen Saturation and Clinical Symptoms in Patients With COVID-19	26	Cancelled
NCT04296643	Medical Masks vs N95 Respirators for COVID-19	28	No medical treatment intervention
NCT04302519	Novel Coronavirus Induced Severe Pneumonia Treated by Dental Pulp Mesenchymal Stem Cells	31	Clinical, not randomised
NCT04307693	Comparison of Lopinavir/Ritonavir or Hydroxychloroquine in Patients With Mild Coronavirus Disease (COVID-19)	33	Cancelled
NCT04299724	Safety and Immunity of Covid-19 aAPC Vaccine	34	Clinical, not randomised
NCT04306055	Blood Donor Recruitment During Epidemic of COVID-19	35	Observational
IRCT20160625028622N1	The effect of Noscapine on clinical and pulmonary manifestations of COVID-19 patients	37	Cinical, not randomised
IRCT20100228003449N27	Interferon ß in treatment of COVID-19	38	Duplicate
IRCT20151227025726N12	Evaluting the therapeutic and adverse effects of Interferon beta 1-a in patients with novel Coronavirus(COVID-19)	40	Clinical, not randomised
IRCT20100228003449N30	Combination therapy in COVID 19	41	Clinical, not randomised
JPRN-jRCTs031190226	Favipiravir in patients infected with COVID-19	42	Clinical, not randomised
NCT04226157	Home Blood Pressure Study for Recent Stroke Survivors With High Blood Pressure *Due to COVID-19 Outbreak in United States the Investigators Have Paused All In-person Enrollment and Will	46	Not COVID-19 study
NCT04251767	Washed Microbiota Transplantation for Patients With 2019-nCoV Infection	48	Cancelled
NCT04287686	Recombinant Human Angiotensin-converting Enzyme 2 (rhACE2) as a Treatment for Patients With COVID-19	53	Cancelled
NCT04276896	Immunity and Safety of Covid-19 Synthetic Minigene Vaccine	54	Clinical, not randomised
NCT04293692	Therapy for Pneumonia Patients iInfected by 2019 Novel Coronavirus	57	Cancelled
NCT04264858	Treatment of Acute Severe 2019-nCoV Pneumonia With Immunoglobulin From Cured Patients	59	Cinical, not randomised
NCT04278963	Yinhu Qingwen Decoction for the Treatment of Mild / Common CoVID-19	60	Cancelled
NCT04304313	A Pilot Study of Sildenafil in COVID-19	61	Clinical, not randomised
NCT04313322	Treatment of COVID-19 Patients Using Wharton's Jelly-Mesenchymal Stem Cells	63	Clinical, not randomised
NCT04310865	Yinhu Qingwen Granula for the Treatment of Severe CoVID-19	64	Cancelled
NCT04316728	Clinical Performance of the VivaDiag ™ COVID-19 lgM / IgG Rapid Test in Early Detecting the Infection of COVID-19	68	Clinical, not randomised
NCT04317092	Tocilizumab in COVID-19 Pneumonia (TOCIVID-19)	70	Clinical, not randomised
NCT03331445	Inhaled Gaseous Nitric Oxide (gNO) Antimicrobial Treatment of Difficult Bacterial and Viral Lung (COVID-19) Infections	71	Not COVID-19 study
NCT04290871	Nitric Oxide Gas Inhalation for Severe Acute Respiratory Syndrome in COVID-19.	72	Cancelled
NCT03680274	Lessening Organ Dysfunction With VITamin C	73	Not COVID-19 study
NCT04283461	Safety and Immunogenicity Study of 2019-nCoV Vaccine (mRNA-1273) for Prophylaxis SARS CoV-2 Infection	77	Cinical, not randomised
NCT04275414	Bevacizumab in Severe or Critical Patients With COVID-19 Pneumonia	81	Observational
NCT04291729	Evaluation of Ganovo (Danoprevir) Combined With Ritonavir in the Treatment of Novel Coronavirus Infection	83	Clinical, not randomised
NCT04315987	NestCell® Mesenchymal Stem Cell to Treat Patients With Severe COVID-19 Pneumonia	88	Cinical, not randomised
NCT04313127	A Phase I Clinical Trial in 18-60 Adults	95	Cinical, not randomised
NCT04319445	Mindfulness During COVID-19	97	Clinical, not randomised
NCT04320277	Baricitinib in Symptomatic Patients Infected by COVID-19: an Open-label, Pilot Study.	99	Cinical, not randomised
NCT04320238	Experimental Trial of rhIFNa Nasal Drops to Prevent 2019-nCOV in Medical Staff	100	Cinical, not randomised
NCT04321993	Treatment of Moderate to Severe Coronavirus Disease (COVID-19) in Hospitalized Patients	101	Cinical, not randomised
NCT04322344	Escin in Patients With Covid-19 Infection	102	Cinical, not randomised
NCT04324190	DIgital Online SuPport for COVID-19 StrEss	106	Observational
NCT04320953	Non-contact Endoscopy at Covid-19 Outbreak	107	Clinical, not randomised
NCT03808922	Phase III DAS181 Lower Tract PIV Infection in Immunocompromised Subjects (Substudy: DAS181 for COVID-19): RCT Study	111	Not COVID-19 study
NCT04273321	Efficacy and Safety of Corticosteroids in COVID-19	113	Clinical, not randomised
NCT04324489	DAS181 for Severe COVID-19: Compassionate Use	115	Clinical, not randomised
NCT04326114	Effectiveness and Safety of Respiratory Training in the Prevention and Severity of COVID-19	118	No medical treatment intervention
NCT04321421	Hyperimmune Plasma for Critical Patients With COVID-19	119	Clinical, not randomised
NCT04323514	Use of Ascorbic Acid in Patients With COVID 19	121	Clinical, not randomised
NCT04327349	Investigating Effect of Convalescent Plasma on COVID-19 Patients Outcome: A Clinical Trial	128	Clinical, not randomised
NCT04323332	Traditional Chinese Medicine for Severe COVID-19	130	Cinical, not randomised
NCT04323592	Efficacy of Methylprednisolone for Patients With COVID-19 Severe Acute Respiratory Syndrome	132	Cinical, not randomised
NCT04329611	Hydroxychloroquine to Prevent Covid19 Pneumonia (ALBERTA HOPE-Covid19)	136	Clinical, not randomised
NCT04326036	Use of cSVF Via IV Deployment for Residual Lung Damage After Symptomatic COVID-19 Infection	139	Cinical, not randomised
NCT04321928	Personalized Health Education Against the Health Damage of Novel Coronavirus (COVID-19) Outbreak in Hungary	142	Observational
NCT04326452	Treating COVID-19 With a Bidirectional Oxygenation Valve	147	Clinical, not randomised
NCT04329533	Effects of Using Mobile App on Perceived Stress During Coronavirus ("COVID 19") Pandemic	158	Observational
NCT04325672	Convalescent Plasma to Limit Coronavirus Associated Complications	160	Clinical, not randomised
NCT04325867	Integrated Distance Management Strategy for Patients With Cardiovascular Diseases in the Context of COVID-19	161	Clinical, not randomised
NCT04328129	Household Transmission Investigation Study for COVID-19 in French Guiana	164	Cinical, not randomised
NCT04331795	Tocilizumab to Prevent Clinical Decompensation in Hospitalized, Non-critically Ill Patients With COVID-19 Pneumonitis	166	Cinical, not randomised
NCT04331613	Safety and Efficacy of CAStem for Severe COVID-19 Associated With/Without ARDS	175	Clinical, not randomised
NCT04332081	Hyperbaric Oxygen for COVID-19 Patients	177	Cinical, not randomised
NCT04331366	Bidirectional Oxygenation Valve in the Management of Pulmonary Complications of COVID-19	181	Clinical, not randomised
NCT04331665	Study of the Efficacy and Safety of Ruxolitinib to Treat COVID-19 Pneumonia	183	Clinical, not randomised
NCT04333355	Safety in Convalescent Plasma Transfusion to COVID-19	185	Clinical, not randomised
NCT04334044	Treatment of SARS Caused by COVID-19 With Ruxolitinib	194	Clinical, not randomised
ISRCTN14966673	Does point-of-care testing for coronavirus in hospital improve patient care compared to laboratory testing?	195	Cinical, not randomised
NCT04333225	Hydroxychloroquine in the Prevention of COVID-19 Infection in Healthcare Workers	198	Cinical, not randomised
NCT04329572	Efficacy and Safety of Hydroxychloroquine and Azithromycin for the Treatment of Hospitalized Patients With Moderate to Severe COVID-19	199	Cancelled
NCT04332380	Convalescent Plasma for Patients With COVID-19: A Pilot Study	201	Clinical, not randomised
NL8483	A weapon against COVID-19: (Social) media and influencers	204	Observational
NL8490	An open label randomized controlled trial of chloroquine, hydroxychloroquine or only supportive care in patients admitted with moderate to severe COVID-19	208	Cinical, not randomised
NL8491	Countering Lung Damage in COVID-19 infection (CounterCovid) study	209	Cinical, not randomised
ISRCTN86534580	A trial evaluating treatments for suspected coronavirus infection in people aged 50 years and above with pre-existing conditions and those aged 65 years and above	214	Duplicate
ACTRN12620000448943	Expressive Writing To Combat Distress Associated With The COVID-19 Pandemic In People With Inflammatory Bowel Disease	215	Observational
ACTRN12620000443998	Home rehabilitation for people with COVID-19: Implementing telehealth approaches to care	218	Observational
ACTRN12620000447954	Use of therapeutic drug monitoring (TDM) to optimise oral/enteral hydroxychloroquine dosing in critically ill patients with COVID-19	219	Cinical, not randomised
EUCTR2015-002340-14-NL	Adaptive trial in severe pneumonia (REMAP-CAP)	220	Not COVID-19 study
EUCTR2020-000890-25-FR	Hydroxychloroquine as a treatment for coronavirus disease COVID-19	221	Cinical, not randomised
EUCTR2020-001162-12-FR	Sarilumab COVID-19	222	Duplicate
EUCTR2020-001224-33-DE	Systematic study of the medicine hydroxychloroquine against placebo for the treatment of adult patients with acute coronavirus disease 2019 – COVID-19	225	Duplicate
EUCTR2020-001270-29-GB	Hydroxychloroquine for COVID 19 in moderately to severely ill hospitalized patients	228	Cancelled
EUCTR2020-001172-15-DK	Recombinant human angiotensin-converting enzyme 2 (rhACE2) as a treat-ment for patients with COVID-19	230	Duplicate
EUCTR2020-001386-37-IT	Efficacy of Tocilizumab - COVID-19	231	Duplicate
EUCTR2020-001010-38-NO	NORWEGIAN CORONAVIRUS DISEASE 2019 [NO-COVID-19] STUDY: A TRIAL TO EVALUATE THE ANTIVIRAL EFFECT OF CHLOROQUINE IN ADULT PATIENTS WITH	232	Duplicate
EUCTR2020-000936-23-FR	Multi-centre, adaptive, randomized trial of the safety and efficacy of treatments of COVID-19 in hospitalized adults - DisCoVeRy	233	Duplicate
EUCTR2020-001254-22-BE	Sargramostim in patients with acute hypoxic respiratory failure due to COVID-19	234	Duplicate
EUCTR2020-001039-29-GR	MANAGEMENT OF NOVEL SARS CORONAVIRUS	235	Cinical, not randomised
EUCTR2020-001345-38-GR	CHLOROQUINE PHOSPHATE AGAINST INFECTION BY THE NOVEL CORONAVIRUS	236	Cinical, not randomised
EUCTR2020-001271-33-FR	HYCOVID - Hydroxychloroquine versus placebo chez les patients ayant une infection COVID-19 à risque d'aggravation secondaire : étude prospective multicentrique randomisée en double	237	Duplicate
EUCTR2020-001281-11-FR	Evaluation of the concentration/viral effect relationship of hydroxychloroquine in COVID-19 patients in the intensive care unit..	238	Cinical, not randomised
EUCTR2020-001275-32-DK	Anti-il6 treatment of serious COVID-19 disease with threatening respiratory failure	239	Duplicate
EUCTR2020-001500-41-BE	Treatment of COVID-19 patients with anti-interleukin drugs	240	Duplicate
ChiCTR2000029935	A Single-arm Clinical Trial for Chloroquine Phosphate in the treatment of Novel Coronavirus Pneumonia 2019 (COVID-19)	241	Clinical, not randomised
ChiCTR2000029850	Study on convalescent plasma treatment for severe patients with novel coronavirus pneumonia (COVID-19)	245	Cinical, not randomised
ChiCTR2000029814	Clinical Trial for Integrated Chinese and Western Medicine in the Treatment of Children with Novel Coronavirus Pneumonia (COVID-19)	246	Cinical, not randomised
ChiCTR2000029778	Clinical Study for Traditional Chinese Medicine Combined With Western Medicine in Treatment of Novel Coronavirus Pneumonia (COVID-19)	254	Cinical, not randomised
ChiCTR2000029819	Ba-Bao-Dan in the adjuvant therapy of novel coronavirus pneumonia (COVID-19) patients	260	Cinical, not randomised
ChiCTR2000029639	Study for Mental health and psychological status of doctors, nurses and patients in novel coronavirus pneumonia (COVID-19) designated hospital and effect of interventions	273	Observational
ChiCTR2000029636	Efficacy and safety of aerosol inhalation of vMIP in the treatment of novel coronavirus pneumonia (COVID-19): a single arm clinical trial	274	Clinical, not randomised
ChiCTR2000029621	Clinical study of arbidol hydrochloride tablets in the treatment of novel coronavirus pneumonia (COVID-19)	275	Duplicate
ChiCTR2000029600	Clinical study for safety and efficacy of Favipiravir in the treatment of novel coronavirus pneumonia (COVID-19)	279	Cinical, not randomised
ChiCTR2000029609	A prospective, open-label, multiple-center study for the efficacy of chloroquine phosphate in patients with novel coronavirus pneumonia (COVID-19)	280	Cinical, not randomised
ChiCTR2000029542	Study for the efficacy of chloroquine in patients with novel coronavirus pneumonia (COVID-19)	282	Cinical, not randomised
ChiCTR2000029603	A Randomized, Open-Label, Multi-Centre Clinical Trial Evaluating and Comparing the Safety and Efficiency of ASC09/Ritonavir and Lopinavir/Ritonavir for Confirmed Cases of Novel	287	Duplicate
ChiCTR2000029589	An open, prospective, multicenter clinical study for the efficacy and safety of Reduning injection in the treatment of ovel coronavirus pneumonia (COVID-19)	290	Cinical, not randomised
ChiCTR2000029578	Chinese medicine prevention and treatment program for novel coronavirus pneumonia (COVID-19): a perspective, sing-arm trial	291	Cinical, not randomised
ChiCTR2000029468	A real-world study for lopinavir/ritonavir (LPV/r) and emtritabine (FTC) / Tenofovir alafenamide Fumarate tablets (TAF) regimen in the treatment of novel coronavirus pneumonia (COVID-19)	293	Cinical, not randomised
ChiCTR2000029558	Recommendations of Integrated Traditional Chinese and Western Medicine for Diagnosis and Treatment of Novel Coronavirus Pneumonia (COVID-19) in Sichuan Province	294	Clinical, not randomised
ChiCTR2000029460	The effect of shadowboxing for pulmonary function and quality of life in patients with novel coronavirus pneumonia (COVID-19) in rehabilitation period	300	No medical treatment intervention
ChiCTR2000029436	A single arm study for evaluation of integrated traditional Chinese and western medicine in the treatment of novel coronavirus pneumonia (COVID-19)	301	Clinical, not randomised
ChiCTR2000029459	The effect of pulmonary rehabilitation for pulmonary function and quality of life in patients with novel coronavirus pneumonia (COVID-19) in rehabilitation period	305	No medical treatment intervention
ChiCTR2000029400	Clinical Controlled Trial for Traditional Chinese Medicine in the treatment of Novel Coronavirus Pneumonia (COVID-19)	307	Cinical, not randomised
ChiCTR2000029432	A Real World Study for the Efficacy and Safety of Large Dose Tanreqing Injection in the Treatment of Patients with Novel Coronavirus Pneumonia (COVID-19)	308	Cinical, not randomised
ChiCTR2000029381	A prospective comparative study for Xue-Bi-Jing injection in the treatment of novel coronavirus pneumonia (COVID-19)	310	Cinical, not randomised
ChiCTR2000030170	Study for safety and efficacy of Jakotinib hydrochloride tablets in the treatment severe and acute exacerbation patients of novel coronavirus pneumonia (COVID-19)	315	Cinical, not randomised
ChiCTR2000030165	Clinical study for ozonated autohemotherapy in the treatment of Novel Coronavirus Pneumonia (COVID-19)	317	Cinical, not randomised
ChiCTR2000030117	A multicenter, randomized, open, parallel controlled trial for the evaluation of the effectiveness and safety of Xiyanping injection in the treatment of common type novel coronavirus pneumonia	320	Duplicate
ChiCTR2000030090	A Prospective Randomized Controlled Trial for Home Exercise Prescription Intervention During Epidemic of Novel Coronary Pneumonia (COVID-19) in College Students	323	Observational
ChiCTR2000030093	Study for application of simplified cognitive-behavioral therapy for related emergency psychological stress reaction of medical providers working in the position of treatment and control of novel	324	Observational
ChiCTR2000030084	A multicenter study for efficacy of intelligent psychosomatic adjustment system intervention in the treatment of novel coronavirus pneumonia (COVID-19) patients with mild to moderate anxiety	325	No medical treatment intervention
ChiCTR2000030136	Humanistic Care in Patients With Novel Coronavirus Pneumonia (COVID-19)	326	No medical treatment intervention
ChiCTR2000030041	A single-arm, single-center clinical trial for Azivudine tablets in the treatment of adult novel coronavirus pneumonia (COVID-19)	330	Clinical, not randomised
ChiCTR2000030046	A single arm trial to evaluate the efficacy and safety of anti-2019-nCoV inactivated convalescent plasma in the treatment of novel coronavirus pneumonia patient (COVID-19)	333	Clinical, not randomised
ChiCTR2000030029	A multi-center study on the efficacy and safety of suramin sodium in adult patients with novel coronavirus pneumonia (COVID-19)	336	Clinical, not randomised
ChiCTR2000030027	Traditional Chinese medicine cooperative therapy for patients with Novel coronavirus pneumonia (COVID-19) and its effect on spermatogenesis: a randomized controlled trial	337	Clinical, not randomised
ChiCTR2000030039	Clinical study for infusing convalescent plasma to treat patients with new coronavirus pneumonia (COVID-19)	339	Cinical, not randomised
ChiCTR2000030020	The clinical application and basic research related to mesenchymal stem cells to treat novel coronavirus pneumonia (COVID-19)	345	Cinical, not randomised
ChiCTR2000030000	An open, controlled clinical trial for evaluation of ganovo combined with ritonavir and integrated traditional Chinese and Western medicine in the treatment of novel coronavirus infection	346	Cinical, not randomised
ChiCTR2000029999	A clinical study for probiotics in the regulation of intestinal function and microflora structure of novel coronavirus pneumonia (COVID-19)	349	Cinical, not randomised
ChiCTR2000029960	Comparative study for integrate Chinese and conventional medicine the the treatment of novel coronavirus pneumonia (COVID-19) in Hu'nan province	360	Cinical, not randomised
ChiCTR2000029954	Efficacy and safety of honeysuckle oral liquid in the treatment of novel coronavirus pneumonia (COVID-19): a multicenter, randomized, controlled, open clinical trial	366	Duplicate
ChiCTR2000030433	Application of Rehabilitation and Lung Eight-segment Exercise in Home Rehabilitation of Survivors from novel coronavirus pneumonia (COVID-19)	367	Clinical, not randomised
ChiCTR2000030420	A Clinical Trial Study for the Influence of TCM Psychotherapy on Negative Emotion of Patients with Novel Coronavirus Pneumonia (COVID-19) Based on Network Platform	369	No medical treatment intervention
ChiCTR2000030386	Study for moxibustion in the preventing of novel coronavirus pneumonia (COVID-19)	370	Cinical, not randomised
ChiCTR2000030432	Application of rehabilitation and Lung eight-segment exercise in front-line nurses in the prevention of novel coronavirus pneumonia (COVID-19) epidemic	371	Cinical, not randomised
ChiCTR2000030442	Combination of Tocilizumab, IVIG and CRRT in severe patients with novel coronavirus pneumonia (COVID-19)	374	Cinical, not randomised
ChiCTR2000030424	A single-center, single-arm clinical trial for azvudine in the treatment of novel coronavirus pneumonia (COVID-19)	375	Clinical, not randomised
ChiCTR2000030317	Clinical study for a new type of Gastroscope isolation mask for preventing and controlling the novel coronavirus pneumonia (COVID-19) Epidemic period	376	No medical treatment intervention
ChiCTR2000030314	Traditional Chinese medicine Ma-Xing-Shi-Gan-Tang and Sheng-Jiang-San in the treatment of children with novel coronavirus pneumonia (COVID-19)	377	Clinical, not randomised
ChiCTR2000030324	Traditional Chinese Medicine 'Zang-Fu Point-pressing' massage for children with novel coronavirus pneumonia (COVID-19)	379	Cinical, not randomised
ChiCTR2000030300	Umbilical cord mesenchymal stem cells (hucMSCs) in the treatment of high risk novel coronavirus pneumonia (COVID-19) patients	380	Cinical, not randomised
ChiCTR2000030196	A multicenter, single arm, open label trial for the efficacy and safety of CMAB806 in the treatment of cytokine release syndrome of novel coronavirus pneumonia (COVID-19)	385	Clinical, not randomised
ChiCTR2000030013	A prospective clinical study for recombinant human interferon alpha 1b spray in the prevention of novel coronavirus (COVID-19) infection in highly exposed medical staffs.	390	Cinical, not randomised
ChiCTR2000030219	Study for evaluation of integrated traditional Chinese and Western Medicine in the treatment of Novel Coronavirus Pneumonia (COVID-19)	392	Cinical, not randomised
ChiCTR2000030091	A prospective randomized controlled trial for the home exercise prescription intervention in nursing students during epidemic of novel coronary pneumonia (COVID-19)	393	No medical treatment intervention
ChiCTR2000029975	Single arm study for exploration of chloroquine phosphate aerosol inhalation in the treatment of novel coronavirus pneumonia (COVID-19)	396	Clinical, not randomised
ChiCTR2000029761	Cancelled due to lack of patient Clinical study on the safety and effectiveness of Hydroxychloroquine Sulfate tablets in the treatment of patients with novel coronavirus pneumonia	397	Cancelled
ChiCTR2000029762	Cancelled due to lack of patient Clinical study for the effect and	399	Cancelled

Registry entry ID	Title	Database ID (ID all)	Reason for exclusion
ChiCTR2000029760	Cancelled due to lack of patient A study for the efficacy of hydroxychloroquine for mild and moderate COVID-19 infectious diseases	400	Cancelled
ChiCTR2000029759	Retracted due to lack of patient A multicenter, randomized, open label, controlled trial for the efficacy and safety of ASC09/ Ritonavir compound tablets and Lopinavir/	402	Cancelled
ChiCTR2000030162	Cancelled by the investigator Study for the water extract of soybean in the treatment of patients with novel coronavirus pneumonia (COVID-19)	404	Cancelled
ChiCTR2000030503	Extracorporeal blood purification therapy using Li's Artifical Liver System for patients with severe novel coronavirus pneumonia (COVID19) patient	409	Cinical, not randomised
ChiCTR2000030487	A single-center, single-arm clinical trial for azvudine in the treatment of novel coronavirus pneumonia (COVID-19)	413	Cinical, not randomised
ChiCTR2000030490	To evaluate the efficacy and safety of diammonium glycyrrhizinate enteric-coated capsules combined with hydrogen-rich water in the treatment of novel coronavirus pneumonia (COVID-19)	415	Cinical, not randomised
ChiCTR2000030472	An open and controlled clinical study to evaluate the efficacy and safety of Ganovo combined with ritonavir in the treatment of novel coronavirus pneumonia (COVID-19)	416	Cinical, not randomised
ChiCTR2000030475	Cytosorb in Treating Critically Ill Hospitalized Adult Patients with novel coronavirus pneumonia (COVID-19)	422	Clinical, not randomised
ChiCTR2000030312	Cancelled, due to modify the protocol A single-center, open-label and single arm trial to evaluate the efficacy and safety of anti-SARS-CoV-2 inactivated convalescent	425	Clinical, not randomised
ChiCTR2000030465	Cancelled by the investigator Influence of nasal high-fow preoxygenation on video laryngoscope for emergency intubation in patients with critical novel coronavirus pneumonia	426	Cancelled
ChiCTR2000030014	Cancelled due to lack of patient Effect of early pulmonary training on lung function and quality of life for novel coronavirus pneumonia (COVID-19) patients	427	Cancelled
ChiCTR2000030477	oXiris Membrane in Treating Critically Ill Hospitalized Adult Patients with novel coronavirus pneumonia (COVID-19)	430	Cinical, not randomised
ChiCTR2000029550	Recommendations for Diagnosis and Treatment of Influenza Patients in the Hospital of Chengdu University of Traditional Chinese Medicine Under the Raging of Novel Coronavirus Pneumonia	431	Cinical, not randomised
ChiCTR2000029433	A randomized, open-label, blank-controlled trial for Lian-Hua Qing-Wen Capsule/Granule in the treatment of suspected novel coronavirus pneumonia (COVID-19)	434	Duplicate
ChiCTR2000030896	Clinical Application and Theoretical Discussion of Fu-Zheng Qing-Fei Thought in Treating Non-Critical Novel Coronavirus Pneumonia (COVID-19)	439	Cinical, not randomised
ChiCTR2000030894	Favipiravir Combined With Tocilizumab in the Treatment of novel coronavirus pneumonia (COVID-19) - A Multicenter, Randomized, Controlled Trial	442	Duplicate
ChiCTR2000030864	Traditional Chinese Medicine for novel coronavirus pneumonia (COVID-19)	443	Cinical, not randomised
ChiCTR2000030855	Study for the effect of external diaphragmatic pacing assisted invasive ventilation and weaning in patients with severe novel coronavirus pneumonia (COVID-19)	445	Cinical, not randomised
ChiCTR2000030836	Novel coronavirus pneumonia (COVID-19) combined with Chinese and Western medicine based on "Internal and External Relieving -Truncated Torsion" strategy	446	Cinical, not randomised
ChiCTR2000030841	Exploratory study for Immunoglobulin From Cured COVID-19 Patients in the Treatment of Acute Severe novel coronavirus pneuvirus (COVID-19)	447	Cinical, not randomised
ChiCTR2000030849	Investigation on psychological status of novel coronavirus pneumonia (COVID-19) rehabilitation patients in Zhengzhou City and research on coping strategies	448	Cinical, not randomised
ChiCTR2000030835	Clinical study for the efficacy of Mesenchymal stem cells (MSC) in the treatment of severe novel coronavirus pneumonia (COVID-19)	449	Clinical, not randomised
ChiCTR2000030832	Study for the pathogenesis and effective intervention of mood disorders caused by the novel coronavirus pneumonia (COVID-19)	450	No medical treatment intervention
ChiCTR2000030810	Clinical observation and evaluation of traditional Chinese medicine in the treatment of novel coronavirus pneumonia (COVID-19) in Hubei 672 Orthopaedics Hospital of Integrated	451	Observational
ChiCTR2000030761	Continuous renal replacement therapy (CRRT) alleviating inflammatory response in severe patients with novel coronavirus pneumonia (COVID-19) associated with renal injury: A Prospective	455	Cinical, not randomised
ChiCTR2000030773	Application of blood purification in the treatment of novel coronavirus pneumonia (COVID-19)	456	Cinical, not randomised
ChiCTR2000030751	Clinical Research for Traditional Mongolian Medicine in the treatment of novel coronavirus pneumonia (COVID-19)	457	Cinical, not randomised
ChiCTR2000030744	Clinical Application of ECMO(or Ultra-Protective Lung Mechanical Ventilation) in the Treatment of Patients with ARDS due to novel Coronavirus Pneumonia (COVID-19)	460	Cinical, not randomised
ChiCTR2000030718	Randomized controlled trial for Chloroquine Phosphate in the Treatment of novel coronavirus pneumonia (COVID-19)	461	Duplicate
ChiCTR2000030681	An anaesthesia procedure and extubation strategy for reducing patient agitation and cough after extubation that can be used to prevent the spread of Novel Coronavirus Pneumonia (COVID-19)	468	No medical treatment intervention
ChiCTR2000030483	Cancelled by the investigator A multicentre, randomized, controlled trial for the Ba-Duan-Jin in the adjunctive treatment of patients with common type novel coronavirus pneumonia (COVID-	469	Cancelled
ChiCTR2000030478	Cancelled by the investigator Study for the Effectiveness and Safety of Compound Yuxingcao Mixture in the Treatment of the Novel Coronavirus Pneumonia (COVID-19)	470	Cancelled
ChiCTR2000030329	Cancelled by the investigator Clinical trial for umbilical cord blood CIK and NK cells in the treatment of mild and general patients infected with novel coronavirus pneumonia	471	Cancelled
ChiCTR2000030509	Cancelled by the investigator Clinical Study of NK Cells in the Treatment of Novel Coronavirus Pneumonia (COVID-19)	472	Cancelled
ChiCTR2000030031	Cancelled by the investigator A randomized, double-blind, parallel, controlled trial for comparison of phosphoric chloroquine combined with standard therapy and standard	473	Cancelled
ChiCTR2000030476	Study for meditation assists for the rehabilitation of patients with novel coronaviruse pneumonia (COVID-19)	475	No medical treatment intervention
ChiCTR2000030417	Cancelled by the investigator Efficacy and safety of chloroquine phosphate inhalation combined with standard therapy in the treatment of novel coronavirus pneumonia (COVID-	476	Cancelled
ChiCTR2000030082	Cancelled by the investigator Randomized controlled trial for the efficacy of dihydroartemisinine piperaquine in the treatment of mild/common novel coronavirus pneumonia (COVID-19)	477	Cancelled
ChiCTR2000029984	Cancelled by the investigator Study on the effect of human placenta biological preparation on the defense of immune function against novel coronavirus pneumonia (COVID-19)	478	Cancelled
ChiCTR2000030381	Cancelled by investigator A randomized, open-label, controlled and single-center trial to evaluate the efficacy and safety of anti-SARS-CoV-2 inactivated convalescent plasma in the	479	Cancelled
ChiCTR2000030135	Cancelled by the investigator A randomized controlled trial for high-dose Vitamin C in the treatment of severe and critical novel coronavirus pneumonia (COVID-19) patients	480	Cancelled
ChiCTR2000029895	Cancelled by investigator Single arm study for the efficacy and safety of GD31 in patients with novel coronavirus pneumonia (COVID-19)	482	Cancelled
ChiCTR2000029817	Cancelled by the investigator Clinical Study of Cord Blood NK Cells Combined with Cord Blood Mesenchymal Stem Cells in the Treatment of Acute Novel Coronavirus Pneumonia	483	Cancelled
ChiCTR2000029818	Cancelled by the investigator Clinical Study for Umbilical Cord Blood Plasma in the Treatment of Acute Novel Coronavirus Pneumonia (COVID-19)	484	Cancelled
ChiCTR2000029837	Cancelled by the investigator A randomized, double-blind, parallel, controlled trial for comparison of phosphoric chloroquine combined with standard therapy and standard therapy in	485	Cancelled
ChiCTR2000029826	Cancelled by the investigator A randomized, double-blind, parallel, controlled trial for comparison of phosphoric chloroquine combined with standard therapy and standard therapy in	486	Cancelled
ChiCTR2000029816	Cancelled by the investigator Clinical Study for Cord Blood Mesenchymal Stem Cells in the Treatment of Acute Novel Coronavirus Pneumonia (COVID-19)	487	Cancelled
ChiCTR2000029812	Cancelled by the investigator Clinical Study for Umbilical Cord Blood Mononuclear Cells in the Treatment of Acute Novel Coronavirus Pneumonia (COVID-19)	488	Cancelled
ChiCTR2000031139	Safety and Effectiveness of Human embryonic stem cell-derived M cells (CAStem) for Pulmonary Fibrosis Correlated with novel coronavirus pneumonia (COVID-19)	489	Cinical, not randomised
ChiCTR2000031089	A medical records based study for Tou-Jie-Qu-Wen Granules in the Treatment of mild and moderate patients with novel coronavirus pneumonia (COVID-19)	490	Cinical, not randomised
ChiCTR2000031090	Psychological Support for Diagnosed Patients with novel coronavirus pneumonia (COVID-19)	492	No medical treatment intervention
ChiCTR2000031023	Perioperative immune prediction and intervention of tumor patients undergoing surgery during the novel coronavirus pneumonia (COVID-19) outbreak period	493	Observational
ChiCTR2000030989	Internet based Solution Focused Brief Therapy treating adolescent anxiety under the novel coronavirus pneumonia (COVID-19) outbreak: A randomized controlled trial	494	Observational
ChiCTR2000030939	Preliminary evaluation of the safety and efficacy of oral LL-37 antiviral peptide (CAS001) in the treatment of novel coronavirus pneumonia (COVID-19)	497	Cinical, not randomised
ChiCTR2000030946	Effects of different VTE prevention methods on the prognosis of hospitalized patients with novel coronavirus pneumonia (COVID-19)	498	Cinical, not randomised
ChiCTR2000030539	Study for clinical oral characteristics of patients with novel coronavirus pneumonia (COVID-19) and Effect of 3% hydrogen peroxide gargle on the Intraoral novel coronavirus	502	Cinical, not randomised
ChiCTR2000030920	Evaluation of the effect of taking tricholoma matsutake, cannabis sativa capsule and dendrobium candidum to nutrition intervention of patients with novel coronavirus pneumonia (COVID-19)	503	No medical treatment intervention
ChiCTR2000030224	Cancelled by the investigator Clinical study of mesenchymal stem cells in treating severe novel coronavirus pneumonia (COVID-19)	509	Cancelled
ChiCTR2000031328	The protective effect of sleep psychology and music therapy of novel coronavirus pneumonia (COVID-19) mild and moderate type patients	512	No medical treatment intervention
ChiCTR2000031272	The effectiveness of mindfulness intervention in improving people's psychological and physical well-being during novel coronavirus pneumonia (COVID-19) outbreak	514	Cinical, not randomised
ChiCTR2000030940	Study for "Bai-Du Duan Fang" application on the acupoint in the treatment of general type novel coronavirus pneumonia (COVID-19)	517	Cinical, not randomised
ChiCTR2000031196	Efficacy and optimization of antiviral therapy for novel coronavirus pneumonia (COVID-19) patients	519	Cinical, not randomised
ChiCTR2000031630	Clinical study for Celebrex in the treatment of novel coronavirus pneumonia (COVID-19)	527	Cinical, not randomised
ChiCTR2000031540	Study for clinical effect of rehabilitation nursing program for patients with novel coronavirus pneumonia (COVID-19)	528	No medical treatment intervention
ChiCTR2000031639	Effects of a single session internet-delivered cognitive behavioral therapy for Acute Insomnia during the pandemic of novel coronavirus pneumonia (COVID-19)	529	No medical treatment intervention
ChiCTR2000031503	Application of non-invasive multichannel cardiopulmonary monitoring cloud management platform in patients with Novel Coronavirus Pneumonia (COVID-19)	531	No medical treatment intervention
ChiCTR2000031501	The efficacy of convalescent plasma in patients with critical novel coronavirus pneumonia (COVID-19): a pragmatic, prospective cohort study	532	Cinical, not randomised
ChiCTR2000031430	Clinical study of human umbilical cord mesenchymal stem cells in the treatment of novel coronavirus pneumonia (COVID-19) induced pulmonary fibrosis	533	Cinical, not randomised
ChiCTR2000031494	Clinical study for stem cells in the treatment of severe novel coronavirus pneumonia (COVID-19)	534	Cinical, not randomised
RBR-9d8z6m	Open and controlled trial of hydroxychloroquine and azytromicyn use to prevent complications in patients infected by new coronavirus (COVID-19): a randomized controlled trial	538	Duplicate
IRCT20160310026998N10	Effect of metformin in COVID-19 mortality rate and symptom improvement	548	Duplicate
IRCT20200324046852N1	Evaluation of efficacy of pharmacotherapy treatment of COVID- 19 infection using oral Levamisole and Formoterol+Budesonide inhaler and comparison of this treatment protocol with standard	553	Duplicate
IRCT20200321046828N1	The effect of genetical nanocomposit drug with cell imunity level on human coronavirus (COVID-19)	559	Cinical, not randomised
IRCT20200324046850N2	Comparison of the effect of Sofosbuvir + Daclatasvir (Sovodac) and Ribavirin in Covid-19 Patients with Severe Symptoms	565	Cinical, not randomised
IRCT20200324046851N1	Effects of nano curcumin supplementation on the reduction of inflammation and mortality in patients with coronavirus 2019 admitted to ICU ward of Imam Reza hospital in Tabriz	585	Cinical, not randomised
EUCTR2020-001154-22	A RANDOMIZED, DOUBLE-BLIND, PLACEBO-CONTROLLED, MULTICENTER STUDY TO EVALUATE THE SAFETY AND EFFICACY OF TOCILIZUMAB IN PATIENTS WITH	590	Duplicate
EUCTR2020-001385-11	Prevention of SARS-CoV-2 (COVID-19) through Pre-Exposure Prophylaxis with Tenofovir disoproxil/Emtricitabine and Hydroxychloroquine in Healthcare Personnel: Randomized Clinical Trial	591	Duplicate
EUCTR2020-001565-37	PRE-EXPOSURE PROPHYLAXIS WITH HYDROXYCHLOROQUINE FOR HIGH-RISK HEALTHCARE WORKERS DURING THE COVID-19 PANDEMIC (PrEP_COVID): A	592	Duplicate
EUCTR2020-001200-42	The Impact of Camostat Mesilate on COVID-19 Infection: An investigator-initiated randomized, placebo-controlled, phase IIa trial	593	Duplicate
EUCTR2020-001250-21	COVID-19 - Epidemiology of SARS-CoV-2 and Mortality to Covid19 Disease upon Hydroxychloroquine and Azithromycin Therapy in French Cancer patients	595	Cinical, not randomised
EUCTR2020-001335-28	A pragmatic adaptive open label, randomized Phase II/III multicenter study of IFX-1 in Patients with severe COVID-19 Pneumonia - "PANAMO"	596	Duplicate
EUCTR2020-001052-18	A Multicenter, Adaptive, Randomised Blinded Controlled Trial of the Safety and Efficacy of Investigational Therapeutics for the Treatment of COVID-19 in Hospitalized Adults - Version for	598	Duplicate
EUCTR2020-001306-35	Protective role of inhaled steroids for COVID-19 infection	600	Duplicate
EUCTR2020-001373-70	IMMUNONCOVID-20 : A prospective, controlled, randomized, multicenter study to compare the efficacy of a chloroquine analog (GNS561), anti PD-1 (nivolumab) and anti-interleukine-6	601	Duplicate
EUCTR2020-000842-32	A Phase 3 Randomized Study to Evaluate the Safety and Antiviral Activity of Remdesivir (GS-5734™) in Participants with Moderate COVID-19 Compared to Standard of Care Treatment	603	Duplicate
EUCTR2020-000841-15	A Phase 3 Randomized Study to Evaluate the Safety and Antiviral Activity of Remdesivir (GS-5734™) in Participants with Severe COVID-19	604	Duplicate
EUCTR2020-001188-96	Chemoprophylaxis of SARS-CoV-2 infection (COVID-19) in exposed healthcare workers: a randomized double-blind placebo-controlled clinical trial	605	Duplicate
EUCTR2020-001442-19	Pilot, randomized, multicenter, open-label clinical trial of combined use of hydroxychloroquine, azithromycin, and tocilizumab for the treatment of SARS-CoV-2 infection (COVID-19)	607	Duplicate
EUCTR2020-001409-21	A prospective, multicenter, randomized, parallel, double-blind, placebo-controlled phase IIb clinical trial to evaluate intravenous infusion of Defibrotide in the prevention and treatment of	609	Duplicate
ChiCTR2000029626	Immune Repertoire (TCR & BCR) Evaluation and Immunotherapy Research in Peripheral Blood of Novel Coronavirus Pneumonia (COVID-19)	612	No medical treatment intervention
ChiCTR2000030015	Study for the correlation between the incidence and outcome of novel coronary pneumonia (COVID-2019) and ovarian function in women	613	No medical treatment intervention
ChiCTR2000030687	Novel coronavirus pneumonia (COVID-19) associated kidney injury in children	614	No medical treatment intervention
ChiCTR2000030690	Study for immune cell subsets in convalescent patients with novel coronavirus pneumonia (COVID-19)	615	No medical treatment intervention
ChiCTR2000030950	Study for novel coronavirus pneumonia (COVID-19) patients etiology and immune response and guidance for vaccine design	616	No medical treatment intervention
ChiCTR2000031432	A single center clinical study of evaluating the injection of microRNA2911 plasmid in healthy adults	617	No medical treatment intervention
ChiCTR2000031627	Study for the Mechanism of the Relationship between Infection of novel coronavirus pneumonia (COVID-19) and Host Innate Immune	618	No medical treatment intervention
ChiCTR2000029883	A comparative study on the sensitivity of nasopharyngeal and oropharyngeal swabbing for the detection of SARS-CoV-2 by real-time PCR	619	No medical treatment intervention
ChiCTR2000029810	Clinical study of a novel high sensitivity nucleic acid assay for novel coronavirus pneumonia (COVID-19) based on CRISPR-cas protein	620	No medical treatment intervention
ChiCTR2000029870	Evaluation of Rapid Diagnostic Kit (IgM/IgG) for Novel Coronavirus Pneumonia (COVID-19)	621	No medical treatment intervention
ChiCTR2000029779	Study for the key issues of the diagnosis and treatment of novel coronavirus pneumonia (COVID-19) based on the medical imaging	622	No medical treatment intervention
ChiCTR2000029695	Early Detection of Novel Coronavirus Pneumonia (COVID-19) Based on a Novel High-Throughput Mass Spectrometry Analysis With Exhaled Breath	623	No medical treatment intervention
ChiCTR2000029625	Construction of Early Warning and Prediction System for Patients with Severe / Critical Novel Coronavirus Pneumonia (COVID-19)	624	No medical treatment intervention
ChiCTR2000030185	The Value of Critical Care Ultrasound in Rapid Screening, Diagnosis, Evaluation of Effectiveness and Intensive Prevention of Novel Coronavirus Pneumonia (COVID-19)	625	No medical treatment intervention
ChiCTR2000029981	Study for nucleic acid detection of novel coronavirus pneumonia (COVID-2019) in female vaginal secretions	626	No medical treatment intervention
ChiCTR2000029866	Early warning prediction of patients with severe novel coronavirus pneumonia (COVID-19) based on multiomics	627	No medical treatment intervention
ChiCTR2000030390	Research and Development of Diagnostic Assistance Decision Support System for novel coronavirus pneumonia (COVID-19) Based on Big Data Technology	628	No medical treatment intervention
ChiCTR2000030334	microRNA as a marker for early diagnosis of novel coronavirus infection (COVID-19)	629	No medical treatment intervention
ChiCTR2000030253	Exploration and Research for a new method for detection of novel coronavirus (COVID-19) nucleic acid	630	No medical treatment intervention
ChiCTR2000030556	Clinical study of nano-nose and its extended technology in diagnosis of novel coronavirus pneumonia (COVID-19)	631	No medical treatment intervention
ChiCTR2000030856	An artificial intelligence assistant system for suspected novel coronavirus pneumonia (COVID-19) based on chest CT	632	No medical treatment intervention
ChiCTR2000030838	Development of warning system with clinical differential diagnosis and prediction for severe type of novel coronavirus pneumonia (COVID-19) patients based on artificial intelligence and CT	633	No medical treatment intervention
ChiCTR2000030833	Clinical validation and application of high-throughput novel coronavirus (2019-nCoV) screening detection kit	634	No medical treatment intervention
ChiCTR2000030799	Establishment and validation of Premonitory model of deterioration of the 2019 novel corona virus pneumonia (COVID-19)	635	No medical treatment intervention
ChiCTR2000030800	Cancelled by the investigator Study on levels of inflammatory factors in peripheral blood of patients with novel coronavirus pneumonia (COVID-19) and their diagnostic and prognostic	636	No medical treatment intervention
ChiCTR2000030754	Medical records based study for the accuracy of SARS-CoV-2 IgM antibody screening for diagnosis of novel coronavirus pneumonia (COVID-19)	637	No medical treatment intervention
ChiCTR2000030721	A comparative study for the sensitivity of induced sputum and throat swabs for the detection of SARS-CoV-2 by real-time PCR in patients with novel coronavirus pneumonia (COVID-19)	638	No medical treatment intervention
ChiCTR2000030706	Cancelled by the investigator Application of cas13a-mediated RNA detection in the assay of novel coronavirus nucleic acid (COVID-19)	639	No medical treatment intervention
ChiCTR2000030683	The prediction value of prognosis of novel coronavirus pneumonia (COVID-19) in elderly patients by modified early warning score (MEWS): a medical records based retrospective observational	640	No medical treatment intervention
ChiCTR2000030114	Lung ultrasound in the diagnosis, treatment and prognosis of pulmonary lesions of novel coronavirus pneumonia (COVID-19)	641	No medical treatment intervention
ChiCTR2000030005	Nucleic acid analysis of novel coronavirus pneumonia (COVID-19) in morning sputum samples and pharyngeal swabs-a prospectively diagnostic test	642	No medical treatment intervention
ChiCTR2000030085	Cancelled by the investigator Study for the false positive rate of IgM / IgG antibody test kit for novel coronavirus pneumonia (COVID-19) in different inpatients	643	No medical treatment intervention
ChiCTR2000030992	A medical records based study for the diagnosis and prognosis prediction AI model of novel coronavirus pneumonia (COVID-19)	644	No medical treatment intervention
ChiCTR2000030993	A medical records based study for the value of Upper Respiratory Tract Virus Detection in the assessment of rehabilitation of novel coronavirus pneumonia (COVID-19) patient	645	No medical treatment intervention
ChiCTR2000030951	Developing and evaluating of artificial intelligence triage system for suspected novel coronavirus pneumonia (COVID-19): a retrospective study	646	No medical treatment intervention
ChiCTR2000029955	Evaluation of myocardial injury of novel coronavirus pneumonia (COVID-19) assessed by multimodal MRI imaging	647	No medical treatment intervention
ChiCTR2000030771	Screening and identification of peripheral blood biomarkers in patients with COVID-19 infection based on multiomics studies	648	No medical treatment intervention
ChiCTR2000031336	AI based prognostic evaluation of novel coronavirus pneumonia (COVID-19)	649	No medical treatment intervention
ChiCTR2000031365	To explore the pathogenesis and course prediction of novel coronavirus pneumonia (COVID-19) severe patients	650	No medical treatment intervention
ChiCTR2000031427	Novel coronavirus infection (COVID-19) IgM detection kit (magnetic particle chemiluminescence) clinical trial	651	No medical treatment intervention
ChiCTR2000031465	Novel coronavirus (2019-nCOV) IgG detection kit (magnetic particle chemiluminescence) clinical trial	652	No medical treatment intervention
ChiCTR2000031428	Clinical application value of multiple tests for novel coronavirus pneumonia (COVID-19)	653	No medical treatment intervention
ChiCTR2000030331	Construction of a Bio information platform for novel coronavirus pneumonia (COVID-19) patients follow-up in Anhui	654	Observational
ChiCTR2000030263	Investigation and analysis of psychological status of hospital staff during the novel coronavirus pneumonia (COVID-19) epidemic	655	Observational
ChiCTR2000030489	Study for the route of ocular surface transmission of novel coronavirus pneumonia (COVID-19) infection and related eye diseases	656	Observational
ChiCTR2000030464	Study for the clinical characteristics of novel coronavirus pneumonia (COVID-19)	657	Observational
ChiCTR2000030768	Study for the Psychological Status of Medical Staff of Otolaryngology Head and Neck Surgery in Hubei Province under the Epidemic of novel coronavirus pneumonia (COVID-19)	658	Observational
ChiCTR2000030762	Diagnosis and treatment of novel coronavirus pneumonia (COVID-19) in common and severe cases based on the theory of "Shi-Du-Yi (damp and plague)"	659	Observational
ChiCTR2000030558	Cancelled by the investigator Epidemiological research of novel coronavirus pneumonia (COVID-19) suspected cases based on virus nucleic acid test combined with low-dose chest CT	660	Observational
ChiCTR2000030765	Cancelled by the investigator Clinical guidance of diagnose and treatment for novel coronavirus pneumonia (COVID-19) based on "Shi-Du-Yi"	661	Observational
ChiCTR2000031187	A medical records based retrospective study for novel coronavirus pneumonia (COVID-19)	662	Observational
ChiCTR2000030256	Epidemiological and clinical characteristics of COVID-19: a large-scale investigation in epicenter Wuhan, China	663	Observational
NCT04302766	Expanded Access Remdesivir (RDV; GS-5734™)	664	Cinical, not randomised
NCT04288713	Eculizumab (Soliris) in Covid-19 Infected Patients	665	Cinical, not randomised
ChiCTR2000030223	Quality of life among Chinese residents during and after novel coronavirus pneumonia (COVID19) outbreak: an online survey	666	No medical treatment intervention
ChiCTR2000030861	Development and application of a new intelligent robot for novel coronavirus (2019-nCOV) oropharygeal sampling	667	No medical treatment intervention
ChiCTR2000030221	Cancelled by the investigator Study for sleep status of medical staffs of hospitals administrating suspected cases of Novel Coronavirus Pneumonia (COVID-19)	668	Cancelled
ChiCTR2000031150	Application of radiology in the prevention and control system of emergency respiratory infectious diseases	669	Observational
ChiCTR2000031538	Assessment of mental health status of first-line dental emergency medical personnel during novel coronavirus pneumonia (COVID-19) pandemic	670	No medical treatment intervention
ChiCTR2000031500	Impact of measuring distance and cold outdoor environment on the screening of novel coronavirus pneumonia (COVID-19)	671	Observational
ChiCTR2000031533	Survey of psychological status and related factors of emergency medical staff in the country during the novel coronavirus pneumonia (COVID-19) pandemic	672	No medical treatment intervention
NCT04245631	Development of a Simple, Fast and Portable Recombinase Aided Amplification Assay for 2019-nCoV	673	Observational
NCT04255940	2019-nCoV Outbreak and Cardiovascular Diseases	674	Observational
NCT04260308	A Survey of Psychological Status of Medical Workers and Residents in the Context of 2019 Novel Coronavirus Pneumonia	675	Observational
NCT04259892	Viral Excretion in Contact Subjects at High/Moderate Risk of Coronavirus 2019-nCoV Infection	676	Observational

Registry entry ID	Title	Database ID (ID all)	Reason for exclusion
NCT04279899	The Investigation of the Neonates With or With Risk of COVID-19	677	Observational
NCT04256395	Efficacy of a Self-test and Self-alert Mobile Applet in Detecting Susceptible Infection of COVID-19	678	Observational
NCT04270383	Clinical Characteristics and Long-term Prognosis of 2019-nCoV Infection in Children	679	Observational
NCT04275947	The COVID-19 Mobile Health Study (CMHS)	680	Observational
NCT04274322	Identifying Critically-ill Patients With COVID-19 Who Will Benefit Most From Nutrition Support Therapy: Validation of the NUTRIC Nutritional Risk Assessment Tool	681	Observational
NCT04279782	Clinical Features of Suspected and Confirmed Patients of 2019 Novel Coronavirus Infection	682	Observational
NCT04279795	Detection of 2019 Novel Coronavirus in Multiple Organ System and Its Relationship With Clinical Manifestations	683	Observational
NCT04284046	CT Scores Predict Mortality in 2019-nCoV Pneumonia	684	Observational
NCT04292327	Clinical Progressive Characteristics and Treatment Effects of 2019-novel Coronavirus	685	Observational
NCT04299711	Mental Health and Its Correlates Among Chinese Adolescents Exposed to the Novel Coronavirus Disease 2019	686	Observational
NCT04292340	Anti-SARS-CoV-2 Inactivated Convalescent Plasma in the Treatment of COVID-19	687	Observational
NCT04298814	Safety Related Factors of Endotracheal Intubation in Patients With Severe Covid-19 Pneumonia	688	Observational
NCT04285801	Critically Ill Patients With COVID-19 in Hong Kong: a Multicentre Observational Cohort Study	689	Observational
NCT04302688	Accurate Classification System for Patients With COVID-19 Pneumonitis	690	Observational
DRKS00021134	Identification of host cell respiratory tract cell gene expression and epigenetic pattern after human SARS-CoV-2 infection	691	Observational
TCTR20200321001	ED Response against Covid-19 pandemic	692	Observational
JPRN-UMIN000039686	ExtraCorporeal Membrane Oxygenation for 2019 novel Coronavirus Acute Respiratory Disease	693	Observational
JPRN-UMIN000039873	COVID-19 Registry	694	Observational
TCTR20200324001	Covid-19 preparedness among Emergency Departments: a cross-sectional study in France.	695	Observational
NCT04283396	Study for Novel Coronavirus Pneumonia (NCP)	696	Observational
NCT04292964	Prognostic Factors of Patients With COVID-19	697	Observational
NCT04272710	Prognositc Factors in COVID-19 Patients Complicated With Hypertension	698	Observational
NCT04306705	Tocilizumab vs CRRT in Management of Cytokine Release Syndrome (CRS) in COVID-19	699	Observational
NCT04314232	Mechanisms to Morbidity and Mortality for Covid-19	700	Observational
NCT04314817	Adverse Events Related to Treatments Used Against Coronavirus Disease 2019	701	Observational
NCT04312464	Myocardial Damage in COVID-19	702	Observational
NCT04305574	Social Media Use During COVID-19	703	Observational
NCT04311398	Development and Verification of a New Coronavirus Multiplex Nucleic Acid Detection System	704	Observational
NCT04306497	Clinical Trial on Regularity of TCM Syndrome and Differentiation Treatment of COVID-19.	705	Observational
NCT04312100	Sequential Oxygen Therapy Strategy for Patients With COVID-19	706	Observational
NCT04318418	ACE Inhibitors, Angiotensin II Type-I Receptor Blockers and Severity of COVID-19	707	Observational
NCT04316884	Mechanisms for Organ Dysfunction in Covid-19	708	Observational
NCT04061382	Sero-epidemiological Survey of England in 2019/2020	709	Observational
NCT04313946	Artificial Intelligence Algorithms for Discriminating Between COVID-19 and Influenza Pneumonitis Using Chest X-Rays	710	Observational
NCT04316299	Acute Kidney Injury in Patients Hospitalized With COVID-19	711	Observational
NCT04319211	Effects of Social Isolation From Coronavirus; on Physical Activity, Quality of Life and Stress	712	Observational
NCT04318301	Hypertension in Patients Hospitalized With COVID-19	713	Observational
NCT04320017	Joint Use of Electrocardiogram and Transthoracic Echocardiography in an Observational Study to Monitor Cardio-vascular Events in Patients Diagnosed With COVID-19	714	Observational
NCT04323644	Outcomes of Surgery in COVID-19 Infection: International Cohort Study (CovidSurg)	715	Observational
NCT04324047	Cohort Multiple Randomized Controlled Trials Open-label of Immune Modulatory Drugs and Other Treatments in COVID-19 Patients	716	Observational
NCT04320511	Beaumont Quantitative Lung Function Imaging to Characterize Patients With SARS-COV 2	717	Observational
NCT04320732	Risk Factors for Community- and Workplace Transmission of COVID-19	718	Observational
NCT04322188	An Observational Case-control Study of the Use of Siltuximab in ARDS Patients Diagnosed With COVID-19 Infection	719	Observational
NCT04322513	Biomarkers for Identification of SARS-COV-2 Infection	720	Observational
NCT04322487	Simple, Safe, Same: Lung Ultrasound for COVID-19	721	Observational
NCT04318366	COVID-19 Patients Characterization, Biobank, Treatment Response and Outcome Predictor	722	Observational
NCT04319315	Social Media Effect on Knowledge Dissemination During COVID-19 Virus Outbreak	723	Observational
NCT04327531	Evaluation of Covid 19 Knowledge Anxiety and Expectation Levels of Turkish Physicians, Survey Study	724	Observational
NCT04320862	Pandemic Response Network	725	Observational
NCT04321265	Outcomes and Prognostic Factors in COVID-19	726	Observational
NCT04329507	Non-invasive Detection of Pneumonia in Context of Covid-19 Using Gas Chromatography - Ion Mobility Spectrometry (GC-IMS)	727	Observational
NCT04325919	Coronavirus Disease 2019 (COVID-19) Study of Hospitalized Patients in Hong Kong	728	Observational
NCT04323787	Viral Infection and Respiratory Illness Universal Study[VIRUS]: COVID19 Registry	729	Observational
NCT04322786	The Use of Angiotensin Converting Enzyme Inhibitors and Incident Respiratory Infections, Are They Harmful or Protective?	730	Observational
NCT04330261	Clinical Characteristics and Outcomes of Pediatric COVID-19	731	Observational
NCT04330521	Impact of the Coronavirus (COVID-19) on Patients With Cancer	732	Observational
NCT04324866	Prevalence and Incidence of COVID-19 Infection in Patients With Chronic Plaque Psoriasis on Immunosuppressant Therapy	733	Observational
NCT04326387	Evaluation of Novel Diagnostic Tests for 2019-nCOV	734	Observational
NCT04325646	Sero-epidemiological Study of the SARS-CoV-2 Virus in France: Constitution of a Collection of Human Biological Samples	735	Observational
NCT04331886	An Observational Study of Patients With Coronavirus Disease 2019	736	Observational
NCT04330599	Longitudinal Population-based Observational Study of Coronavirus Disease in the UK Population	737	Observational
NCT04326309	Audio Data Collection for Identification and Classification of Coughing	738	Observational
NCT04326725	Proflaxis Using Hydroxychloroquine Plus Vitamins-Zinc During COVID-19 Pandemia	739	Observational
NCT04327180	PREdiction of DIagnosed Covid-19 infecTion in IUC Patients	740	Observational
NCT04333862	Assessment of Covid-19 Infection Rates in Healthcare Workers Using a Desynchronization Strategy	741	Observational
ISRCTN13694948	Distress and resilience of healthcare professionals during the COVID-19 (coronavirus) pandemic	742	Observational
ISRCTN51287266	Aspergillosis in patients with severe influenza or coronavirus	743	Observational
NCT04332913	Efficacy and Safety of Tocilizumab in the Treatment of SARS-Cov-2 Related Pneumonia	744	Observational
NCT04328454	Clinical Characteristics and Prognostic Factors of Patients With COVID-19	745	Observational
NCT04332016	COVID-19 Biological Samples Collection	746	Observational
ISRCTN40092247	UKOSS: Pandemic COVID-19 in pregnancy	747	Observational
NCT04329546	Understanding COVID	748	Observational
NCT04331509	COVID-19 Symptom Tracker	749	Observational
NCT04333849	Telephony Or Videophony for Isolated elDerly in Maine-Et-Loire 49	750	Observational
NL8485	COVID-19 during pregnancy: a prospective observational cohort	751	Observational
ACTRN12620000421932	ExtraCorporeal Membrane Oxygenation for 2019 novel Coronavirus Acute Respiratory Disease (COVID-19)	752	Observational
ACTRN12620000449932	Maternal and neonatal outcomes from women infected with SARS-COV2 (COVID-19) during pregnancy	753	Observational
ACTRN12620000444987	COVID-19 Research Response (ISARIC/WHO Clinical Characterisation Protocol for Severe Emerging Infections COVID-19 Research Response Trial)	754	Observational
NL8460	Biomarkers for prognosis in critically ill COVID-19 patients : a prospective cohort study	755	Observational
NL8473	Immunity against SARS-CoV-2 in Dutch population	756	Observational
ACTRN12620000438954	Utilizing lung ultrasound in COVID-19 patients in ICU – comparison of management strategies on survival and time on ventilation.	757	Observational
NCT04308187	Influence of the COvid-19 Epidemic on STRESS	758	Observational
NCT04314271	The Observational Study of Cardiac and Pulmonary Ultrasound and Evaluation of Treatment of Severe Patients With Novel Coronavirus Pneumonia	759	Observational
NCT04315870	Clinical Characteristics of Coronavirus Disease 2019 (COVID-19) in Pregnancy: The Italian Registry on Coronavirus in Pregnancy.	760	Observational
NCT04319016	Clinical Characteristics of Coronavirus Disease 2019 (COVID-19) in Pregnancy	761	Observational
NCT04318314	COVID-19: Healthcare Worker Bioresource: Immune Protection and Pathogenesis in SARS-CoV-2	762	Observational
NCT04320472	Acute Encephalopathy in Critically Ill Patients With SARS-Cov-2 Infection	763	Observational
NCT04321811	Behavior, Environment And Treatments for Covid-19	764	Observational
NCT04323839	PRIORITY (Pregnancy CoRonavIrus Outcomes RegIsTrY)	765	Observational
NCT04327674	The Use of Focused Lung Ultrasound in Patients Suspected of COVID-19	766	Observational
NCT04327570	In-depth Immunological Investigation of COVID-19.	767	Observational
NCT04323878	Early CPAP in COVID Patients With Respiratory Failure. A Prospective Cohort Study.	768	Observational
NCT04325412	Cardiac complicAtions in Patients With SARS Corona vIrus 2 regisTrY	769	Observational
NCT04331171	Epidemiological Observation From a Smartphone Self-monitoring Application for Suspected COVID-19 Patients' Triage	770	Observational
NCT04327479	Characterization of Cardiovascular Diseases and Risk Factors in Patients With Suspected SARS-CoV2/Covid-19 Infection	771	Observational
NCT04333953	COVID-19 in Patients With HIV	772	Observational
NCT04331860	COVID-19 Public Image Registry	773	Observational
NCT04331574	Renin-Angiotensin System Inhibitors and COVID-19	774	Observational
NCT04329559	COVID-19 in Patients With Pre-existing Cirrhosis (COVID-Cirrhosis-CHESS2002): A Multicentre Observational Study	775	Observational
NCT04333693	Outcomes of Vascular Surgery in COVID-19 Infection: National Cohort Study	776	Observational
ChiCTR2000029949	A Medical Records Based Study for the Effectiveness of Extracorporeal Membrane Oxygenation in Patients with Severe Novel Coronavirus Pneumonia (COVID-19)	777	Observational
ChiCTR2000029953	Construction and Analysis of Prognostic Predictive Model of Novel Coronavirus Pneumonia (COVID-19)	778	Observational
ChiCTR2000029896	Evaluate the effectiveness of Traditional Chinese medicine in the treatment of novel coronavirus pneumonia (COVID-19)	779	Observational
ChiCTR2000029907	Study for construction and assessment of early warning score of the clinical risk of novel coronavirus (COVID-19) infected patients	780	Observational
ChiCTR2000029905	A medical records based study of novel coronavirus pneumonia (COVID-19) and influenza virus co-infection	781	Observational
ChiCTR2000029805	Analysis of clinical characteristics of severe novel coronavirus pneumonia (COVID-19)	782	Observational
ChiCTR2000029815	Psychological survey of frontline medical staff in various regions of China during the epidemic of novel coronavirus pneumonia (COVID-19)	783	Observational
ChiCTR2000029839	An observational study on the clinical characteristics, treatment and outcome of novel coronavirus pneumonia (COVID-19)	784	Observational
ChiCTR2000029782	Clinical study for the changes in mental state of medical staff in the department of radiotherapy in a comprehensive tertiary hospital in Zhejiang Province during the epidemic of novel coronavirus	785	Observational
ChiCTR2000029764	Imaging Features and Mechanisms of Novel Coronavirus Pneumonia (COVID-19): a Multicenter Study	786	Observational
ChiCTR2000029754	Study for the Effect of Novel Coronavirus Pneumonia (COVID-19) on the Health of Different People	787	Observational
ChiCTR2000029758	Cohort Study of Novel Coronavirus Pneumonia (COVID-19) Critical Ill Patients	788	Observational
ChiCTR2000029624	A real world study for traditional Chinese Medicine in the treatment of novel coronavirus pneumonia (COVID-19)	789	Observational
ChiCTR2000029751	Clinical Study for Traditional Chinese Medicine in the Prevention and Treatment of Novel Coronavirus Pneumonia (COVID-19)	790	Observational
ChiCTR2000029735	Risks of Death and Severe cases in Patients with 2019 Novel Coronavirus Pneumonia (COVID-19)	791	Observational
ChiCTR2000029732	Impact of vitamin D deficiency on prognosis of patients with novel coronavirus pneumonia (COVID-19)	792	Observational
ChiCTR2000029637	An observational study for Xin-Guan-1 formula in the treatment of novel coronavirus pneumonia (COVID-19)	793	Observational
ChiCTR2000029734	Epidemiological investigation and clinical characteristics analysis of novel coronavirus pneumonia (COVID-19)	794	Observational
ChiCTR2000029628	A clinical observational study for Xin-Guan-2 formula in the treatment of suspected novel coronavirus pneumonia (COVID-19)	795	Observational
ChiCTR2000029462	Study for clinical characteristics and distribution of TCM syndrome of novel coronavirus pneumonia (COVID-19)	796	Observational
ChiCTR2000029437	A single arm study for combination of traditional Chinese and Western Medicine in the treatment of novel coronavirus pneumonia (COVID-19)	797	Observational
ChiCTR2000029430	Study for the TCM syndrome characteristics of novel coronavirus pneumonia (COVID-19)	798	Observational
ChiCTR2000030164	A cross-sectional study of novel coronavirus pneumonia (COVID-19) patients in ICU	799	Observational
ChiCTR2000030137	Humanistic Care in Healthcare Workers in Novel Coronavirus Pneumonia (COVID-19)	800	Observational
ChiCTR2000030115	A clinical research for the changes of Blood cortisol ACTH level and adrenal morphology in blood cortisol to guide the application of individualized hormone in severe novel coronavirus	801	Observational
ChiCTR2000030096	Study for establishment of correlation between virological dynamics and clinical features in noveal coronavirus pneumonia (COVID-19)	802	Observational
ChiCTR2000030094	Effects of novel coronavirus pneumonia (COVID-19) on menstruation, TCM body construction and psychological state for female at different ages	803	Observational
ChiCTR2000030092	Assessment of cardiac function in patients with Novel Coronavirus Pneumonia (COVID-19) by echocardiography and its new techniques	804	Observational
ChiCTR2000030056	Study for the effect of early endotracheal intubation on the outcome of novel coronavirus pneumonia (COVID-19) patients	805	Observational
ChiCTR2000030030	A medical records based study for acute kidney injury in novel coronavirus pneumonia (COVID-19)	806	Observational
ChiCTR2000030032	Study on ultrasonographic manifestations of new type of novel coronavirus pneumonia (covid-19) in non-critical stage of pulmonary lesions	807	Observational
ChiCTR2000030087	Clinical study for the diagnostic value of pulmonary ultrasound for novel coronavirus pneumonia (COVID-19)	808	Observational
ChiCTR2000030086	Study for the effects on medical providers' infection rate and mental health after performing different anesthesia schemes in cesarean section for novel coronavirus pneumonia (COVID-19)	809	Observational
ChiCTR2000030017	Feature of Multiple Organs in Ultrasound Investigation for Clinical Management and Prognostic Evaluation of Novel Coronavirus Pneumonia (COVID-19)	810	Observational
ChiCTR2000030008	Correalation between anxiety as well as depression and gut microbiome among staff of hospital during the novel coronavirus pneumonia (COVID-19) outbreak	811	Observational
ChiCTR2000030021	A medical records based analysis for the clinical characteristics of novel coronavirus pneumonia (COVID-19) in immunocompromised patients	812	Observational
ChiCTR2000030019	The COVID-19 Mobile Health Study (CMHS), a large-scale clinical observational registration study using nCapp	813	Observational
ChiCTR2000030004	Effect of Novel Coronavirus Pneumonia (COVID-19) on the Mental Health of College Students	814	Observational
ChiCTR2000029995	Study on anxiety of different populations under novel coronavirus (COVID-19) infection	815	Observational
ChiCTR2000029959	Clinical observation and research of Severe acute respiratory syndrome coronavirus 2(COVID-19) infection in perinatal newborns	816	Observational
ChiCTR2000029985	Study for mental health status and influencing factors of nurses during epidemic prevention of novel coronavirus pneumonia (COVID-19)	817	Observational
ChiCTR2000029865	Descriptive study on the clinical characteristics and outcomes of novel coronavirus pneumonia (COVID-19) in cardiovascular patients	818	Observational
ChiCTR2000030382	Construction and application of non-contact doctor-patient interactive diagnosis and treatment mode of moxibustion therapy for novel coronary pneumonia (COVID-19) based on mobile internet	819	Observational
ChiCTR2000030363	Novel Coronavirus Infected Disease (COVID-19) in children: epidemiology, clinical features and treatment outcome	820	Observational
ChiCTR2000030389	A Comparative Study for the Effectiveness of "triple energizer treatment" Method in Repairing Lung Injury in Patients with Novel coronavirus pneumonia (COVID-19)	821	Observational
ChiCTR2000030387	Clinical observation and research of multiple organs injury in severe patients with novel coronavirus pneumonia (COVID-19)	822	Observational
ChiCTR2000030322	Identification and Clinical Treatment of Severe novel coronavirus pneumonia (COVID-19) Patients	823	Observational
ChiCTR2000030315	Clinical Study for Traditional Chinese Medicine in the Prevention and Treatment of Novel Coronavirus Pneumonia (COVID-19)	824	Observational
ChiCTR2000030391	A medical records based analysis for antiviral therapy effect on novel coronavirus pneumonia COVID-19 patients	825	Observational
ChiCTR2000030327	Analysis of clinical characteristics of novel coronavirus pneumonia (COVID-19)	826	Observational
ChiCTR2000030305	Multiomics study and emergency plan optimization of spleen strengthening clearing damp and stomach therapy combined with antiviral therapy for novel coronavirus pneumonia (COVID-19)	827	Observational
ChiCTR2000030325	A survey of mental health of first-line medical service providers and construction of crisis intervention model for novel coronavirus pneumonia (COVID-19) in Xiangyang	828	Observational
ChiCTR2000030283	Correlation between imaging characteristics and laboratory tests of new coronavirus pneumonia (COVID-19)	829	Observational
ChiCTR2000030264	ICU healthcare personnel burnout investigation during the fight against novel coronavirus pneumonia (COVID-19)	830	Observational

Registry entry ID	Title	Database ID (ID all)	Reason for exclusion
ChiCTR2000030293	Clinical observation and research plan of novel coronavirus pneumonia (COVID-19) patients	831	Observational
ChiCTR2000030290	Health related quality of life and its influencing factors among front line nurses caring patients with new coronavirus pneumonia (COVID-19) from two hospitals in China	832	Observational
ChiCTR2000030222	Cancelled due to lack of participants Study for the Impact of Novel Coronavirus Pneumonia (COVID-19) to the Health of the elderly People	833	Observational
ChiCTR2000029952	Cancelled due to lack of patient Medical records based study for epidemiological and clinical characteristics of 2019 novel coronavirus pneumonia (COVID-19) in Chongqing	834	Observational
ChiCTR2000030257	The coagulation function of novel coronavirus pneumonia (COVID-19) patients	835	Observational
ChiCTR2000030226	The treatment status and risk factors related to prognosis of hospitalized patients with novel coronavirus pneumonia (COVID-19) in intensive care unit, Hebei, China: a descriptive study	836	Observational
ChiCTR2000030042	Cancelled due to lack of patient A Medical Records Based Study for Construction and Analysis of Diagnosis Predictive&	837	Observational
ChiCTR2000029900	Research for Risks Associated with Novel Coronavirus Pneumonia (COVID-19) in the Hospital Workers and Nosocomial Prevention and Control Strategy	838	Observational
ChiCTR2000029728	A Retrospective Study for Preventive Medication in Tongji Hospital During the Epidemic of Novel Coronavirus Pneumonia (COVID-19)	839	Observational
ChiCTR2000029829	Cancelled due to lack of patient Medical records based study for Heart-type fatty acid-binding protein on prognosis of novel coronavirus pneumonia (COVID-19)	840	Observational
ChiCTR2000030552	Critical ultrasound in evaluating cardiopulmonary function in critical patients infected with 2019 novel coronavirus pneumonia (COVID-19)	841	Observational
ChiCTR2000030593	Novel coronavirus pneumonia (COVID-19) antiviral related liver dysfunction: a multicenter, retrospective, observational study	842	Observational
ChiCTR2000030581	The immediate psychological impact of novel coronavirus pneumonia (COVID-19) outbreak on medical students in anesthesiology and how the cope	843	Observational
ChiCTR2000030559	A medical records based study for the safety of artificial liver cluster nursing in critically ill patients with novel coronavirus pneumonia (COVID-19)	844	Observational
ChiCTR2000030557	A retrospective study on virus typing, Hematological Immunology and case Review of novel coronavirus infected and convalescent patients (COVID-19)	845	Observational
ChiCTR2000030574	Research on peripheral immunity function monitoring and its clinical application in patients with novel coronavirus pneumonia (COVID-19)	846	Observational
ChiCTR2000030543	Detection of coronavirus in simultaneously collecting tears and throat swab samples collected from the patients with novel coronavirus pneumonia (COVID-19)	847	Observational
ChiCTR2000030541	Novel coronavirus pneumonia (COVID-19) epidemic survey of medical students in various provinces and municipalities throughout the country	848	Observational
ChiCTR2000030542	A clinical study about the diagnosis and prognosis evaluation of novel coronacirus pneumonia (COVID-19) based on viral genome, host genomic sequencing, relative cytokines and other	849	Observational
ChiCTR2000030491	A medical records based study for Comparing Differences of Clinical Features and Outcomes of Novel Coronavirus Pneumonia (COVID-19) Patients between Sichuan Province and Wuhan City	850	Observational
ChiCTR2000030485	Study for timing of mechanical ventilation for critically ill patients with novel coronavirus pneumonia (COVID-19): A medical records based retrospective Cohort study	851	Observational
ChiCTR2000030579	Risk Factors for Outcomes of Novel Coronavirus Pneumonia (COVID-19)	852	Observational
ChiCTR2000030492	Retrospective study for integrate Chinese and conventional medicine treatment of novel coronavirus pneumonia (COVID-19) in Hu'nan province	853	Observational
ChiCTR2000030564	Psychological Intervention of Children with Novel Coronavirus Disease (COVID-19)	854	Observational
ChiCTR2000030544	Study for the risk factors of critically ill patients with novel coronavirus pneumonia (COVID-19)	855	Observational
ChiCTR2000030468	Study for the key technique of integrative therapy of Novel Coronavirus Pneumonia (COVID-19): the TCM symptoms and treatment regulation	856	Observational
ChiCTR2000030482	A Multicenter, Long- term Follow-up and Registration Study for Myocardial Injury and Prognosis of Novel coronavirus pneumonia (COVID-19)	857	Observational
ChiCTR2000030494	Early risk stratification of the Novel coronavirus infected diseases (COVID-19): a multicenter retrospective study (ERS-COVID-19 study)	858	Observational
ChiCTR2000030493	Survey for sleep, anxiety and depression status of Chinese residents during the outbreak of novel coronavirus infected disases (COVID-19)	859	Observational
ChiCTR2000030453	Clinical study for the effects of ACEIs/ARBs on the infection of novel coronavirus pneumonia (CoVID-19)	860	Observational
ChiCTR2000030163	A Medical Based Retrospective Real World Study for Assessment of Effectiveness of Comprehensive Traditional Chinese Medicine in the treatment of Novel Coronavirus Pneumonia (COVID-19)	861	Observational
ChiCTR2000030436	Application of flash glucose monitoring to evaluate the effect of blood glucose changes on prognosis in patients with novel coronavirus pneumonia (COVID-19)	862	Observational
ChiCTR2000030304	Protective factors of mental resilience in first-line nurses with novel coronavirus pneumonia (COVID-19)	863	Observational
ChiCTR2000029579	The investigation of cytokine expression profile of novel coronavirus pneumonia (COVID-19) and its clinical significance	864	Observational
ChiCTR2000029958	A medical records based study for the clinical characteristics of anesthesia novel coronavirus pneumonia (COVID-19) patients during perioperative period and assessment of infection and mental	865	Observational
ChiCTR2000030902	Analysis for the mental health status of residents in Jingzhou during the outbreak of the novel coronavirus pneumonia (COVID-19) and corresponding influencing factors	866	Observational
ChiCTR2000030898	Evaluation on the effect of Chushifangyi prescription in preventing novel coronavirus pneumonia (COVID-19)	867	Observational
ChiCTR2000030895	Retrospective and Prospective Study for Nosocomial infection in Stomatology Department under the Background of novel coronavirus pneumonia (COVID-19) epidemic period	868	Observational
ChiCTR2000030866	Open-label, observational study of human umbilical cord derived mesenchymal stem cells in the treatment of severe and critical patients with novel coronavirus pneumonia (COVID-19)	869	Observational
ChiCTR2000030901	Retrospective analysis of epidemiology and transmission dynamics of patients confirmed with Coronavirus Disease (COVID-19) in Hong Kong	870	Observational
ChiCTR2000030854	A clinical multicenter study for the occurrence, development and prognosis of novel coronavirus pneumonia (COVID-19)	871	Observational
ChiCTR2000030893	Study for effects of crisis intervention based on positive psychology for medical staffs working in the novel coronavirus pneumonia (COVID-19) field	872	Observational
ChiCTR2000030863	Clinical and CT imaging Characteristics of novel coronavirus pneumonia (COVID-19): An Multicenter Cohort Study	873	Observational
ChiCTR2000030852	Factors associated with death in patients with novel coronavirus pneumonia (COVID-19)	874	Observational
ChiCTR2000030883	Clinical research and preparation development of qingfei detoxification decoction (mixture) for prevention and treatment of novel coronavirus pneumonia (COVID-19)	875	Observational
ChiCTR2000030865	Establishment of an early warning model for maternal and child vertical transmission of COVID-19 infection	876	Observational
ChiCTR2000030859	A medical based analysis for influencing factors of death of novel coronavirus pneumonia (COVID-19) patients in Wuhan third hospital	877	Observational
ChiCTR2000030857	Clinical study for bronchoscopic alveolar lavage in the treatment of critically trachea intubation patients with new coronavirus pneumonia (COVID-19)	878	Observational
ChiCTR2000030862	Correlation analysis of blood eosinophil cell levels and clinical type category of novel coronavirus pneumonia (COVID-19): a medical records based retrospective study	879	Observational
ChiCTR2000030850	Study for the physical and mental health status of medical workers under the novel coronavirus pneumonia (COVID-19) epidemic	880	Observational
ChiCTR2000030858	Clinical characteristics and outcomes of 483 mild patients with novel coronavirus pneumonia (COVID-19) in Wuhan, China during the outbreak: A single-center, retrospective study from the	881	Observational
ChiCTR2000030860	A medical records based study for investigation of dynamic profile of RT-PCR test for SARS-CoV-2 nucleic acid of novel coronavirus pneumonia (COVID-19) patients	882	Observational
ChiCTR2000030812	Study for the virus molecular evolution which driven the immune-pathological responses and the protection mechanisms of novel coronavirus pneumonia (COVID-19) patients	883	Observational
ChiCTR2000030834	Epidemiological Characteristics and Antibody Levels of novel coronavirus pneumonia (COVID-19) of Pediatric Medical Staff working in Quarantine Area	884	Observational
ChiCTR2000030830	Development and application of novel coronavirus pneumonia (COVID-19) intelligent image classification system based on deep learning	885	Observational
ChiCTR2000030809	A medical records based study for clinical outcomes and follow-up of novel coronavirus pneumonia (COVID-19) patients	886	Observational
ChiCTR2000030831	The analysis of related factors on improving oxygenation status by endotracheal intubation ventilation in severe patients suffered from novel coronavirus pneumonia (COVID-19): a single center	887	Observational
ChiCTR2000030816	Nutritional risk assessment and outcome prediction of critically ill novel coronavirus pneumonia (COVID-19) patients	888	Observational
ChiCTR2000030819	Retrospective analysis of digestive system symptoms in 600 cases of novel coronavirus pneumonia (COVID-19) in Guanggu district, Wuhan	889	Observational
ChiCTR2000030818	A medical records based study for the value of Lymphocyte subsets in the diagnose and treatment of novel coronavirus pneumonia (COVID-19)	890	Observational
ChiCTR2000030806	Retrospective study for the efficacy of ulinastatin combined with "clear lung detoxification soup" in the treatment of novel coronavirus pneumonia (COVID-19)	891	Observational
ChiCTR2000030814	A medical records based analysis of clinical evidence of human-to-human transmission of 2019 novel coronavirus pneumonia (COVID-19) by conjunctival route	892	Observational
ChiCTR2000030807	Clinical characteristics and prognosis of cancer patients with novel coronavirus pneumonia (COVID-19)	893	Observational
ChiCTR2000030803	Collection and analysis of clinical data in severe and critically ill patients with novel coronavirus pneumonia (COVID-19)	894	Observational
ChiCTR2000030802	A retrospective study of clinical drug therapy in patients with novel coronavirus pneumonia (COVID-19)	895	Observational
ChiCTR2000030797	clinical study for hemodynamics and cardiac arrhythmia of novel coronavirus pneumonia (COVID-19) patients	896	Observational
ChiCTR2000030805	Quantitative CT characteristic estimate the severity of novel coronavirus pneumonia (COVID-19)	897	Observational
ChiCTR2000030798	A medical records based study for clinical characteristics of novel coronavirus pneumonia (COVID-19)	898	Observational
ChiCTR2000030795	A multicenter retrospective study of rheumatic patients with novel coronavirus pneumonia (COVID-19)	899	Observational
ChiCTR2000030778	A medical records based study for epidemic and clinical features of novel coronavirus pneumonia (COVID-19) in Ningbo First Hospital	900	Observational
ChiCTR2000030801	Analysis of risk factors affecting prognosis of elderly patients infected with novel coronavirus pneumonia (COVID-19): a single-center retrospective observational study	901	Observational
ChiCTR2000030763	A Medical Records Based analysis for Risk Factors for Outcomes After Respiratory Support in Patients with ARDS Due to Novel Coronavirus Pneumonia (COVID-19)	902	Observational
ChiCTR2000030796	Clinical characteristics and treatment of novel coronavirus pneumonia (COVID-19)	903	Observational
ChiCTR2000030782	The value of CD4 / CD8 cells, CRP / ALB and APCHEII in novel coronavirus pneumonia (COVID-19)	904	Observational
ChiCTR2000030758	A medical records based study for sedation and Analgesia Usage in critically ill patients with novel coronavirus pneumonia (COVID-19)	905	Observational
ChiCTR2000030784	A study for clinical characteristics of novel coronavirus pneumonia (COVID-19) patients follow-up in Guangxi	906	Observational
ChiCTR2000030764	Research for the influence of epidemic of novel coronavirus pneumonia (COVID-19) on sleep, psychological and chronic diseases among different populations	907	Observational
ChiCTR2000030757	Impact of Novel Coronavirus Pneumonia (COVID-19) Epidemic Period on the Management of investigator-initiated clinical trial and the resilience of medical service providers	908	Observational
ChiCTR2000030756	Detection of SARS-CoV-2 in EPS / semen of patients with novel coronavirus pneumonia (COVID-19)	909	Observational
ChiCTR2000030760	A medical records based study for clinical characteristics of 2019 novel coronavirus pneumonia (COVID-19) in Zhejiang province, China	910	Observational
ChiCTR2000030752	A medical records based analysis for risk factors for death in patients with Novel Coronavirus Pneumonia (COVID-19)	911	Observational
ChiCTR2000030747	A prospective cohort study for comprehensive treatment of Chinese medicine in the treatment of convalescent patients of novel coronavirus pneumonia (COVID-19)	912	Observational
ChiCTR2000030755	A medical records based study for characteristics, prognosis of ederly patients with Novel Coronavirus Pneumonia (COVID-19) in Wuhan area	913	Observational
ChiCTR2000030753	A medical records based analysis of the Incidence and Risk Factors of Ventilator-associated Pneumonia in ARDS Patients with Novel Coronavirus Pneumonia (COVID-19)	914	Observational
ChiCTR2000030742	Characteristics, prognosis, and treatments effectiveness of critically ill patients with Novel Coronavirus Pneumonia (COVID-19)	915	Observational
ChiCTR2000030741	Observational Study for Prone Position Ventilation and Conventional Respiratory Support in ARDS Patients with Novel Coronavirus Pneumonia (COVID-19)	916	Observational
ChiCTR2000030722	Auscultatory characteristics of novel coronavirus pneumonia (COVID-19)	917	Observational
ChiCTR2000030739	Exploration of the Clinical Characteristics of Patients with Novel Coronavirus Pneumonia (COVID-19) and Its Differences from Patients with Severe Influenza A and MERS	918	Observational
ChiCTR2000030717	A medical records based study for risk assessment and treatment timing of invasive fungal infection in novel coronavirus pneumonia (COVID-19) critical patients	919	Observational
ChiCTR2000030740	Analysis of the incidence and risk factors of ARDS in patients with Novel Coronavirus Pneumonia (COVID-19).	920	Observational
ChiCTR2000030708	Cross sectional study of dialysis treatment and mental status under the outbreak of novel coronavirus pneumonia (COVID-2019) in China	921	Observational
ChiCTR2000030719	A retrospective cohort study for integrated traditional Chinese and western medicine in the treatment of 1071 patients with novel coronavirus pneumonia (COVID-19) in Wuhan	922	Observational
ChiCTR2000030707	Retrospective study on novel coronavirus pneumonia (COVID-19) in Tibetan Plateau	923	Observational
ChiCTR2000030716	Shedding of SARS-CoV-2 in human semen and evaluation of reproductive health of novel coronavirus pneumonia (COVID-19) male patients	924	Observational
ChiCTR2000030697	A multi-center, open-label observation study for psychological status and intervention efficacy of doctors, nurses, patients and their families in novel coronavirus pneumonia (COVID-19)	925	Observational
ChiCTR2000030686	The effects of prevention and control measures on treatment and psychological status of cancer patients during the novel coronavirus pneumonia (COVID-19) outbreak	926	Observational
ChiCTR2000030679	Cohort study of Novel Coronavirus Infected Diseases (COVID-19) in children	927	Observational
ChiCTR2000030466	Cancelled due to lack of patients Investigation on Mental Health Status and Psychological Intervention of Hospitalized Patients with Novel Coronavirus Pneumonia (COVID-	928	Observational
ChiCTR2000030619	A medical records based real world study for the characteristics and correlation mechanism of traditional Chinese medicine combined with western medicine in the treatment of patients with novel	929	Observational
ChiCTR2000030606	Medical records based study for the correlation between Chinese medicine certificate and lung image of novel coronavirus pneumonia (COVID-19)	930	Observational
ChiCTR2000030597	Medical records based study for the correlation between Chinese medicine certificate and lung image of novel coronavirus pneumonia (COVID-19)	931	Observational
ChiCTR2000030528	Application of TCM Nursing Scheme in Patients with Novel Coronavirus Pneumonia (COVID-19)	932	Observational
ChiCTR2000030306	Cancelled by the investigator Mechanism of novel coronavirus pneumonia (COVID-19) virus with silent latent immune system induced by envelope protein and vaccine	933	Observational
ChiCTR2000029592	Clinical Study of Arbidol Hydrochloride Using for Post-exposure Prophylaxis of 2019-nCoV in High-risk Population Including Medical Staff	934	Observational
ChiCTR2000029957	Cancelled by the investigator An observational study of high-dose Vitamin C in the treatment of severe and critical patients with novel coronavirus pneumonia (COVID-19)	935	Observational
ChiCTR2000029770	Study for epidemiology, diagnosis and treatment of novel coronavirus pneumonia (COVID-19)	936	Observational
ChiCTR2000029820	Cancelled by investigator A cox regression analysis of prognosis of novel coronavirus pneumonia (COVID-19)	937	Observational
ChiCTR2000031104	Study for metagenomics of patients with novel coronavirus pneumonia (COVID-19)	938	Observational
ChiCTR2000031163	The relationship between Vitamin D andnovel coronavirus pneumonia (COVID-19)	939	Observational
ChiCTR2000031140	Study for the impact on fetus and neonates of vertical transmission of 2019-nCoV	940	Observational
ChiCTR2000031115	A medical records based study of peripheral blood T lymphocyte subsets in patients with novel coronavirus pneumonia (COVID-19)	941	Observational
ChiCTR2000031088	A prognosis study of novel coronavirus pneumonia (COVID-19)	942	Observational
ChiCTR2000031014	A Study for SARS-COV-2 RNA Level in Blood and Excrement of Novel Coronavirus Pneumonia (COVID-19) Convalescent Patients	943	Observational
ChiCTR2000030947	A medical records based study for clinical application of extracorporeal membrane oxygenation in the treatment of severe respiratory failure patients with novel coronavirus pneumonia (COVID-	944	Observational
ChiCTR2000030986	Correlation of T lymphocytes level and clinical severity in novel coronavirus pneumonia (COVID-19) patients: a medical records based retrospective study	945	Observational
ChiCTR2000030941	A medical records based study for clinical features and prognosis of severe patients with novel coronavirus pneumonia (COVID-19) in Huanggan, Hubei in Huanggang.	946	Observational
ChiCTR2000030985	Psychological Responses of Medical Staff during the Metaphase of novel coronavirus pneumonia (COVID-19) Outbreak in Hubei, China	947	Observational
ChiCTR2000030961	A Medical Records Based Retrospective Study for Clinical Characteristics, Treatments and Prognosis of Patients with Novel Coronavirus Pneumonia (COVID-19) in WuHan	948	Observational
ChiCTR2000030952	Myocardial injury and arrythmias in the novel coronavirus pneumonia (COVID-19) patients	949	Observational
ChiCTR2000030942	Combined diagnostic value of novel coronavirus (2019-CoV) infection detected by NLR and CRP	950	Observational
ChiCTR2000030943	Venous Thrombosis Risk of novel coronavirus pneumonia (COVID-19) Patients: A Prospective Study	951	Observational
ChiCTR2000030932	Correlation between virological negative conversion and clinical factors and prognosis in patients with novel coronavirus pneumonia (COVID-19)	952	Observational
ChiCTR2000030931	A Medical Records Based Study for Clinical Characteristic and Outcomes of Hospitalized Patients With Novel Coronavirus Pneumonia (COVID-19)	953	Observational
ChiCTR2000030934	A Platform for Rapid Immuno-detection and Emergency Vaccine Development of Novel Coronavirus (2019-COV)	954	Observational
ChiCTR2000030919	An observational study for cardiac and pulmonary ultrasound and evaluation of treatment of severe patients with novel coronavirus pneumonia (COVID-19)	955	Observational
ChiCTR2000030095	A medical records based study for optimization and evaluation of the comprehensive diagnosis and treatment of novel coronavirus pneumonia (COVID-19) and the assessment of risk factors for	956	Observational
ChiCTR2000030519	Study for the clinical characteristics and digestive system damage of novel coronavirus pneumonia (COVID-19)	957	Observational
ChiCTR2000030168	Cancelled by the investigator A real world study for Compound Houttuyniae Mixture for prevention of novel coronavirus pneumonia (COVID-19)	958	Observational
ChiCTR2000029830	A study for the psychological status, social support, and care needs of tumor patients admitted to a general hospital during the novel coronavirus pneumonia (COVID-19) outbreak	959	Observational
ChiCTR2000031366	Causes of fever in outpatient outside Wuhan during novel coronavirus pneumonia pandemic: a medical records based retrospective analysis	960	Observational
ChiCTR2000031356	Study for the changes of the brain structure and function in healed patients of novel coronavirus pneumonia (COVID-19)	961	Observational
ChiCTR2000031361	Retrospective analysis of anesthesia management of emergency cesarean section in non-pneumonia hospital of Wuhan during pandemic of novel coronavirus pneumonia (COVID-19)	962	Observational
ChiCTR2000031324	The investigation on medical adhesive-related skin injury caused by protective dressings of medical staffs during the period of novel coronavirus pneumonia (COVID-19)	963	Observational
ChiCTR2000031330	Analysis for prevention and control effects of orthokeratology lens on myopia adolescents during the novel coronavirus pneumonia (COVID-19) pandemic situation	964	Observational
ChiCTR2000031329	Influence of novel coronavirus pneumonia (COVID-19) on disease activity, medical and mental condition of patients with rheumatic diseases	965	Observational
ChiCTR2000031327	Clinical characteristics and death risk factors in severe novel coronavirus pneumonia (COVID-19) patients: a single-center respective analysis based on medical records	966	Observational
ChiCTR2000031337	The relationship between habits of using eyes, degree of visual fatigue and myopia progression in students during the novel coronavirus pneumonia (COVID-19) pandemic	967	Observational
ChiCTR2000031301	A retrospective study of clinical characteristics and prognosis of novel coronavirus pneumonia (COVID-19) patients with myocardial injury	968	Observational
ChiCTR2000031247	Study on the mental state of medical staff during the epidemic period of novel coronavirus pneumonia (COVID-19)	969	Observational
ChiCTR2000031252	Outcomes and infectivity of patients with asymptomatic novel coronavirus (COVID-19) infection	970	Observational
ChiCTR2000031296	A medical records based study for clinical features of novel coronavirus pneumonia (COVID-19) patients and risk factors of death	971	Observational
ChiCTR2000031293	Epidemiological study of novel coronavirus infection (COVID-19) in children at medium/low risk	972	Observational
ChiCTR2000031246	Epidemiological survey of close contacts of novel coronavirus pneumonia (COVID-19)	973	Observational
ChiCTR2000031244	Shedding virus and functional prognosis in patients with novel coronavirus pneumonia (COVID-19): a medical records based study	974	Observational
ChiCTR2000031245	Epidemiologic and Clinical Characteristics of novel coronavirus pneumonia (COVID-19) in surgical patients: a medical records based retrospective study	975	Observational
ChiCTR2000031227	Clinical features and prognosis of invasive echanicalventilation patients with novel coronavirus pneumonia (COVID-19) in Wuhan, China: a single-centered, retrospective, observational study	976	Observational
ChiCTR2000031214	Clinical study on the correlation between novel coronavirus pneumonia (COVID-19) clinical rehabilitation assessment and prognosis	977	Observational
ChiCTR2000031198	A medical records based study for clinical characteristics and risk factors of novel coronavirus pneumonia (COVID-19)	978	Observational
ChiCTR2000031176	Study on home pharmaceutical care for chronic patients over the novel coronavirus pneumonia (COVID-19) epidemic	979	Observational
ChiCTR2000030962	Clinical efficacy of TCM syndrome differentiation in the treatment of severe/critical type of novel coronavirus pneumonia (COVID-19): a prospective, observational, one-arm clinical study	980	Observational
ChiCTR2000030938	Clinical investigation and reseach on TCM syndrome of novel coronavirus pneumonia (COVID-19)	981	Observational
ChiCTR2000030330	Cancelled by the investigator Clinical research of 6-minute walk training on motor function of novel coronavirus pneumonia (COVID-19)	982	Observational
ChiCTR2000030817	Multicenter clinical study of evaluation of multi-organ function in patients with novel coronavirus pneumonia (COVID-19) by ultrasound	983	Observational
ChiCTR2000029982	Prediction of prognosis and antiviral effect of patients with neocoronavirus infected pneumonia based on Peripheral blood exosomal metabolomics analysis and radiomics in CT of lung and its	984	Observational

Registry entry ID	Title	Database ID (ID all)	Reason for exclusion
ChiCTR2000031589	Investigation for the sleep quality in non-health care workers during the pandemic of novel coronavirus pneumonia (COVID-19)	985	Observational
ChiCTR2000031587	A paired clinical study for novel coronavirus pneumonia (COVID-19) patients with ordinary, severe, critical, and deceased patients in Tongji Hospital	986	Observational
ChiCTR2000031504	A multi-center telephone follow-up study of disease management and infection in rheumatic patients during novel coronavirus pneumonia (COVID-19) pandemic in Hubei province	987	Observational
ChiCTR2000031499	Sleep quality of patients with novel coronavirus pneumonia (COVID-19) treated in mobile cabin hospital: a retrospective study	988	Observational
ChiCTR2000031438	Analysis of changes in high risk factors of cervical spondylosis during novel coronavirus pneumonia (COVID-19) pandemic	989	Observational
ChiCTR2000031429	The Effects of novel coronavirus pneumonia (COVID-19) Risk Factors on Dynamic Changes of Immunological, biochemical, and Metabolic Parameters: a Retrospective Observational Study	990	Observational
ChiCTR2000031539	A Medical Records Based Study for the Correlation between Angiotensin II Type 1 Receptor Blockers (ARBs) and the Progression and Outcome of Novel Coronavirus Pneumonia (COVID-19)	991	Observational
ChiCTR2000031199	A Medical Records Based Retrospective Study for Clinical Characteristics of severe Novel Coronavirus Pneumonia (COVID-19)	992	Observational
ChiCTR2000031439	A medical records based study for novel coronavirus pneumonia (COVID-19) patients undergoing endotracheal intubation	993	Observational
ChiCTR2000031376	A medical records based study for safety and effectiveness analysis of data from novel coronavirus pneumonia (COVID-19) patients with conventional therapy	994	Observational
ChiCTR2000029821	Based on Delphi Method to Preliminarily Construct a Recommended Protocol for the Prevention of Novel Coronavirus Pneumonia (COVID-19) in Deyang Area by Using Chinese Medicine	995	No medical treatment intervention
ChiCTR2000029487	Clinical Study for Gu-Biao Jie-Du-Ling in Preventing of Novel Coronavirus Pneumonia (COVID-19) in Children	996	Cinical, not randomised
ChiCTR2000030906	A phase I clinical trial for recombinant novel coronavirus (2019-COV) vaccine (adenoviral vector)	997	Cinical, not randomised
ChiCTR2000029804	Clinical Application of ECMO in the Treatment of Patients with Very Serious Respiratory Failure due to novel Coronavirus Pneumonia (COVID-19)	998	Cinical, not randomised
ChiCTR2000030839	Preliminary screening of novel coronavirus pneumonia (COVID-19) by special laboratory examination and CT imaging before surgery	999	Cinical, not randomised
ChiCTR2000030012	Development of anti-2019-nCoV therapeutic antibody from the recovered novel coronavirus pneumonia patients (COVID-19)	1000	Cinical, not randomised
ChiCTR2000030258	A multicenter, randomized, controlled trial for efficacy and safety of hydrogen inhalation in the treatment of novel coronavirus pneumonia (COVID-19) patients	1001	Clinical, not randomised
ChiCTR2000030936	A real-world study for the Chinese medicines "Xinguan 2" and "Xinguan 3" in the treatment of novel coronavirus pneumonia (COVID-19)	1002	Cinical, not randomised
IRCT20200204046369N1	Evaluation of Methylprednisolone Administration as a Therapeutic Option in the 2019 Novel Coronavirus (COVID-19): A Non-Randomized Controlled Study	1003	Cinical, not randomised
IRCT20151228025732N53	Evaluation of the therapeutic effects of Convalescent Plasma (CP) of recovered people from Covid-19 in improving clinical and laboratory symptoms of hospitalized patients	1007	Cinical, not randomised
IRCT20200401046909N2	Investigating preventive effects of oral 25-hydroxyvitamin D3 on COVID-19 in adults: A Randomized, Controlled Double-Blind Clinical Trial.	1012	Duplicate
IRCT20200405046960N1	Evaluating efficacy of Kelofan syrup, a traditional Iranian medicine product, on pulmonary and other clinical and laboratory manifestations of patients with probable or confirmed COVID-19	1014	Cinical, not randomised
IRCT20200401046909N1	The efficacy of oral 25-hydroxyvitamin D3 on COVID-19 treatment in adults: A Randomized, Controlled Double-Blind Clinical Trial	1020	Duplicate
IRCT20180923041093N5	Evaluation the efficacy of hot foot-bath on COVID-19	1023	No medical treatment intervention
IRCT20200217046526N1		1024	Cinical, not randomised
IRCT20171122037571N2	A single-arm multicenter clinical trial to evaluate the safety and efficacy of Remdesivir in COVID-19 patients with progressive severe acute respiratory syndrome coronavirus 2 (SARS-CoV-2)	1025	Clinical, not randomised
IRCT20150303021315N17	Evaluation of the safety and efficacy of tocilizumab (AryoGen Pharmed Co., Iran) in patients with severe COVID-19	1026	Clinical, not randomised
IRCT20180429039463N2	The effect of family-centered empowerment model on stress, anxiety and family satisfaction in patients with Covid-19	1028	No medical treatment intervention
EUCTR2020-001262-11	An international randomised trial of additional treatments for COVID-19 in hospitalised patients who are all receiving the local standard of care	1030	Duplicate
EUCTR2020-001435-27		1032	Duplicate
EUCTR2020-001278-31		1034	Duplicate
EUCTR2020-001110-38		1035	Cinical, not randomised
EUCTR2020-001456-18		1038	Duplicate
EUCTR2020-001457-43		1039	Duplicate
EUCTR2020-001492-33		1040	Clinical, not randomised
EUCTR2020-001455-40		1041	Duplicate
EUCTR2020-001301-23		1042	Duplicate
ChiCTR2000031699	Study for immune antibody and multi-group function of novel coronavirus pneumonia (COVID-19) patients	1044	Cinical, not randomised
ChiCTR2000031700	Development and clinical application of novel coronavirus pneumonia (COVID-19) antigen reagent	1045	Clinical, not randomised
ChiCTR2000031705	Study for early biological warning signals in patients with acute renal injury caused by novel coronavirus pneumonia (COVID-19)	1046	Clinical, not randomised
ChiCTR2000031707	Study for sleep quality of medical teams members moved to Hubei for the fighting of novel coronavirus pneumonia (COVID-19) pandemic	1047	Observational
ChiCTR2000031712	Study for investigation of contamination by novel coronavirus pneumonia (COVID-19) in the clinical environment	1048	Observational
ChiCTR2000031734	a randomized, open-label, controlled trial	1049	Clinical, not randomised
ChiCTR2000031751	Novel coronavirus pneumonia (COVID-2019) patients' prognosis and their influence on heart and lung function	1051	Observational
ChiCTR2000031752	A medical records based observational study for myocardial injury and cardiac function in critically ill patients with novel coronavirus pneumonia (COVID-19)	1052	Observational
ChiCTR2000031770	Experiences in nursing care for patients with suspected novel coronavirus pneumonia (COVID-19) in Shanghai	1053	Observational
ChiCTR2000031779	Effect of novel coronavirus pneumonia (COVID-19) upon male reproductive system	1054	Observational
ChiCTR2000031781	A randomized, double-blinded, placebo-controlled phase II clinical trial for Recombinant Novel Coronavirus (2019-nCOV) Vaccine (Adenovirus Vector)	1055	Duplicate
ChiCTR2000031782	A questionnaire investigation of hydroxychloroquine for its potential protective effect against novel coronavirus infection (COVID-19)	1056	Observational
ChiCTR2000031794	A medical records based retrospective study for analysis clinical characteristics and risk factors of death in patients with novel coronavirus pneumonia (COVID-19)	1057	Observational
ChiCTR2000031834	A clinical study for the effect of remote monitoring exercise rehabilitation on the discharged patients with novel coronavirus pneumonia (COVID-19)	1059	No medical treatment intervention
ChiCTR2000031836	A Medical Records Based study for the Clinical Characteristics Of Hospitalized Novel Coronavirus Pneumonia (COVID-19) Patients With Acute Respiratory Distress Syndrome	1060	Observational
ChiCTR2000031860	Study for the exercise rehabilitation therapy for the dysfunction of cured discharged novel coronavirus pneumonia (COVID-19) patients	1061	No medical treatment intervention
NCT04334252	Descriptive Study Regarding Ambulant Screening During COVID-19 Pandemia	1063	Observational
NCT04334291	International COVID19 Clinical Evaluation Registry,	1065	Observational
NCT04334434	Telerehabilitation in Individuals Over 65 Years of Age Having Social Isolation Due to Coronavirus (Covid-19)	1067	Observational
NCT04334512	A Study of Quintuple Therapy to Treat COVID-19 Infection	1069	Clinical, not randomised
NCT04334850	Use of a Respiratory Multiplex PCR and Procalcitonin to Reduce Antibiotics Exposure in Patients With Severe Confirmed COVID-19 Pneumonia	1071	No medical treatment intervention
NCT04334876	Rapid SARS-CoV-2 IgG Antibody Testing in High Risk Healthcare Workers	1072	Observational
NCT04334954	SARS-COV2 Pandemic Serosurvey and Blood Sampling	1074	Observational
NCT04334967	Hydroxychloroquine in Patients With Newly Diagnosed COVID-19 Compared to Standard of Care	1075	Clinical, not randomised
NCT04335019	Interest of the Use of Pulmonary Ultrasound in the Referral of Patients With or Suspected COVID-19 +	1077	Observational
NCT04335032	EPA-FFA to Treat Hospitalised Patients With COVID-19 (SARS-CoV-2)	1078	Clinical, not randomised
NCT04335084	A Study of Hydroxychloroquine, Vitamin C, Vitamin D, and Zinc for the Prevention of COVID-19 Infection	1080	Clinical, not randomised
NCT04335097	Sensor Based Vital Signs Monitoring of Covid 19 Patients During Home Isolation	1081	No medical treatment intervention
NCT04335123	Study of Open Label Losartan in COVID-19	1082	Clinical, not randomised
NCT04335162	Cardiovascular Complications and COVID-19 (CovCardioVasc)	1084	Observational
NCT04335188	COVID-19 Registry Rhineland-Palatinate (Germany)	1085	Observational
NCT04335201	Defibrotide in COVID-19 Pneumonia	1086	Clinical, not randomised
NCT04335279	Evaluation of the Scleroderma Patient-centered Intervention Network COVID-19 Home-isolation Activities Together Program	1087	Observational
NCT04335630	Cardiovascular Manifestations of COVID-19	1090	Observational
NCT04335747	COVID-19 Infection in Vulnerable Patients With Inflammatory Rheumatic Diseases	1091	Observational
NCT04335773	COVID-19 in Hospitalised Norwegian Children - Risk Factors, Outcomes and Immunology	1092	Observational
NCT04335851	Video-Based Exercises and Well-Being During Social Isolation	1094	Clinical, not randomised
NCT04336215	Rutgers COVID-19 Cohort Study	1095	Observational
NCT04336254	Safety and Efficacy Study of Allogeneic Human Dental Pulp Mesenchymal Stem Cells to Treat Severe COVID-19 Patients	1096	Duplicate
NCT04336345	Outcomes of Patients With COVID-19 in the Intensive Care Unit	1098	Observational
NCT04336384	Impact of Covid-19 in Congenital Heart Disease	1099	Observational
NCT04336410	Safety, Tolerability and Immunogenicity of INO-4800 for COVID-19 in Healthy Volunteers	1100	Cinical, not randomised
NCT04336657	Corona-Like Illness: Did we Got it Before WHO Announcement of the Disease?	1102	Observational
NCT04336761	Prevalence of Covid-19 in Children Admitted to Paediatric Emergency Departments During the Pandemic Period in France	1104	Observational
NCT04336774	CAPTION AI to Minimize Risk of COVID Exposure	1105	Clinical, not randomised
NCT04336787	Physical Activity Level, Stress Level, Sleep Quality in Pregnant Women During Covid-19 Quarantine	1106	Observational
NCT04336956	Covid-19 Pediatric Observatory	1108	Observational
NCT04337008	Renin Angiotesnin System - CoronaVirus	1109	Cinical, not randomised
NCT04337047	Assessment of Distress in Crisis Situations During COVID-19	1110	Observational
NCT04337151	Blood Titanium Analysis of Patients With MAGEC Spine Rods in the COVID-19 Environment	1111	Observational
NCT04337190	Impact of Angiotensin II Receptor Blockers Treatment in Patients With COVID 19 (COVID-ARA2)	1112	Observational
NCT04337216	Mavrilimumab to Reduce Progression of Acute Respiratory Failure in Patients With Severe COVID-19 Pneumonia and Systemic Hyper-inflammation	1113	Clinical, not randomised
NCT04337320	Evaluation of Postnatal Outcomes of Covid 19 Positive Mothers Newborns	1114	Observational
NCT04337346	Evaluation of Covid 19 Anxiety in Endometriosis Patients	1115	Observational
NCT04337359	Ruxolitinib Managed Access Program (MAP) for Patients Diagnosed With Severe/Very Severe COVID-19 Illness	1116	Observational
NCT04337424	Evaluation of Quickly Diagnostic Saliva Tests for SARS-CoV-2	1117	Observational
NCT04337489	Remote Monitoring Use In Cases Of Suspected COVID-19 (Coronavirus)	1118	Observational
NCT04337502	Clinical and Radiomic Model of COVID-19	1119	Observational
NCT04337541	Reduction in COVID-19 Infection Using Surgical Facial Masks Outside the Healthcare System	1120	No medical treatment intervention
NCT04337762	Beat COVID-19 - Observational Trial	1121	Observational
NCT04337788	Gerontological Telemonitoring of Older Adults Living in Nursing Homes With COVID-19 Disease	1122	Cinical, not randomised
NCT04337918	Nitric Oxide Releasing Solutions to Prevent and Treat Mild/Moderate COVID-19 Infection	1123	Clinical, not randomised
NCT04337983	Hemodynamic Characteristics of Patients With SARS-CoV-2	1124	Observational
NCT04337996	Dynamic Evaluation of COVID-19 Diagnostic Tests	1125	Clinical, not randomised
NCT04338009	Elimination or Prolongation of ACE Inhibitors and ARB in Coronavirus Disease 2019	1126	Clinical, not randomised
NCT04338100	Point Of Care UltraSonography for Risk-stratification of COVID-19 Patients	1128	Observational
NCT04338347	CAP-1002 in Severe COVID-19 Disease	1130	Observational
NCT04338360	Expanded Access to Convalescent Plasma for the Treatment of Patients With COVID-19	1131	Observational
NCT04338568	Screening COVID-19 by Point-of-care Lung Ultrasound: a Validation Study	1132	Cinical, not randomised
NCT04338672	The Impact of the Covid-19 Outbreak on Emergency Room Attendances of Surgical Patients	1133	Observational
NCT04338841	HOME-CoV: Hospitalization or Outpatient ManagEment of Patients With a SARS-CoV-2 Infection	1137	Cinical, not randomised
NCT04338932	COVID-19 and Deep Venous Thrombosis	1139	Observational
NCT04338945	Impact of Pandemic COVID-19 on Surgical Specialities Residents in Italy	1140	Observational
NCT04338958	Ruxolitinib in Covid-19 Patients With Defined Hyperinflammation	1141	Clinical, not randomised
NCT04339322	Characteristics and Outcome of Coronavirus Disease 2019 (COVID-19) in Egypt	1142	Observational
NCT04339387	COVID-19 Risk Stratification	1143	Observational
NCT04339426	Atovaquone and Azithromycin Combination for Confirmed COVID-19 Infection	1144	Clinical, not randomised
NCT04339608	Max COVID19- Study	1145	Observational
NCT04339634	Simulation of Risk of Adverse Drug Events Associated With the Initiation of Drugs Repurposed for the Treatment of COVID-19 in Frail Elderly Adults With Polypharmacy	1146	Observational
NCT04339686	Multicentric Retrospective Observational Study of Thoracic Scanner Performance in COVID Screening.	1148	Observational
NCT04339712	Personalised Immunotherapy for SARS-CoV-2 (COVID-19) Associated With Organ Dysfunction	1149	Cinical, not randomised
NCT04339790	Mental Health Impact of COVID-19 Pandemic on NIMH Research Participants and Volunteers	1150	Observational
NCT04339842	Eating Habits of Adults During the Quarantine	1152	Observational
NCT04339881	NOsocomial Dissemination Risk of SARS-Cov2	1153	Observational
NCT04339998	Assessment of Exam Findings in Coronavirus Disease 2019 (COVID-19) With Point-of-Care Ultrasonography (POCUS)	1154	Observational
NCT04340050	COVID-19 Convalescent Plasma	1155	Clinical, not randomised
NCT04340219	Oncology-patient-reported Anxiety, Mood, and QoL During the COVID-19 Pandemic	1156	Observational
NCT04340232	Safety and Efficacy of Baricitinib for COVID-19	1157	Clinical, not randomised
NCT04340414	Safety and Effectiveness of Low-flow ECMO Driving by CVVH Machine in Severe NCP	1159	Clinical, not randomised
NCT04340466	French Multicentre Observational Study on SARS-Cov-2 Infections (COVID-19) ICU Management Study	1160	Observational
NCT04340479	The Use of Ultrasound in Establishing COVID-19 Infection as Part of a Trauma Evaluation	1161	Observational
NCT04340921	The Role of Adaptive Immunity in COVID-19 Associated Myocardial Injury	1164	Observational
NCT04341012	Breath Analysis Based Disease Biomarkers of COVID-19 and Other Diseases	1165	Observational
NCT04341038	Clinical Trial to Evaluate Methylprednisolone Pulses and Tacrolimus in Patients With COVID-19 Lung Injury	1166	Clinical, not randomised
NCT04341103	AlloSure Guided Immuno-Optimization for COVID-19: An Early Experience	1167	Observational
NCT04341142	Assessment of Serological Techniques for Screening Patients Regarding COVID-19-COVID-SER	1169	Clinical, not randomised
NCT04341168	Clinical and Immunological Characterisation of COVID-19 in Children, Adolescents and Adults	1170	Observational
NCT04341207	Epidemiology of SARS-CoV-2 and Mortality to Covid19 Disease in French Cancer Patients	1171	Cinical, not randomised
NCT04341415	Impact of Auricular Vagus Nerve Neuromodulation on COVID-19 Positive Inpatients Outcome	1174	No medical treatment intervention
NCT04341480	The Safety of Chemotherapy for Patients With Gynecological Malignancy in High-risk Region of COVID-19	1176	Observational
NCT04341493	Hydroxychloroquine vs Nitazoxanide in Patients With COVID-19	1177	Clinical, not randomised
NCT04341506	Non-contact ECG Sensor System for COVID19	1178	Observational
NCT04341519	Psychological Burden in ICU Survivors of Severe COVID-19 Pneumonia, Their Relatives and Their Healthcare Providers	1179	Observational
NCT04341610	ASC Therapy for Patients With Severe Respiratory COVID-19	1181	Cancelled
NCT04341688	A Clinical Trial of Gargling Agents in Reducing Intraoral Viral Load in COVID-19 Patients	1183	No medical treatment intervention
NCT04341714	Efficiency and Satisfaction With Telephone Consultation in Neuro-urology: Experience of the COVID-19 Pandemic	1184	Observational
NCT04341766	Evolution of Pulmonary Ultrasound in Patients Hospitalized for Covid (Coronavirus Disease) 19	1186	Observational
NCT04341792	Predictive Biomarkers of Secondary Aggravation in Covid-19 Suspect Patient	1187	Observational
NCT04341922	A Online-delivered Cognitive-behavioral Intervention for Dysfunctional Worry Related to the Covid-19 Pandemic	1189	No medical treatment intervention
NCT04342104	NIV and CPAP Failure Predictors in COVID-19 Associated Respiratory Failure	1191	Observational
NCT04342182	Convalescent Plasma as Therapy for Covid-19 Severe SARS-CoV-2 Disease (CONCOVID Study)	1194	Clinical, not randomised
NCT04342195	Acquiring Convalescent Specimens for COVID-19 Antibodies	1195	Observational
NCT04342208	Evaluate the Working Environments of Individuals Working From Home During Covid-19 Outbreak	1196	Observational
NCT04342637	COVID-19 Endoscopy Survey	1198	Observational
NCT04342702	A Study on the Prospective Cohort Library of COVID-19 in Southeran	1202	Observational
NCT04342728	Coronavirus 2019- Using Ascorbic Acid and Zinc Supplementation	1203	Clinical, not randomised
NCT04342806	Healthcare Worker Exposure Response and Outcomes (HERO) Registry Study, COVID-19	1204	Observational

Registry entry ID	Title	Database ID (ID all)	Reason for exclusion
NCT04342884	COVID-19 Community Research Partnership	1205	Observational
NCT04343053	Pro-thrombotic Status in Patients With SARS-Cov-2 Infection	1208	Clinical, not randomised
NCT04343183	Hyperbaric Oxygen Therapy (HBOT) as a Treatment for COVID-19 (COVID-19) Infection	1211	Clinical, not randomised
NCT04343261	Convalescent Plasma in the Treatment of COVID 19	1213	Clinical, not randomised
NCT04343339	Evolution of Psychoactive Substances Consumption in Connection With COVID-19 Containement	1214	Observational
NCT04343404	PLACE OF VENOVENOUS ECMO IN THE MANAGEMENT OF SEVERE REFRACTORY ARDS ASSOCIATED WITH SARS-COV-2	1215	Observational
NCT04343664	Mental Health Assessment Among Community Member During the Covid-19 Pandemic in Indonesia	1217	Observational
NCT04343690	COPING With COVID-19(CWC-19)	1219	Clinical, not randomised
NCT04343742	Determination of the Effectiveness of Oral Chlorine Dioxide in the Treatment of COVID 19	1221	Observational
NCT04343755	Convalescent Plasma as Treatment for Hospitalized Subjects With COVID-19 Infection	1222	Clinical, not randomised
NCT04343781	National Survey of Symptoms of People Aged 70 and Overs Diagnosed With COVID-19	1224	Observational
NCT04343898	Study of the Treatment and Outcomes in Critically Ill Patients With COVID-19	1226	Observational
NCT04344002	LunG canceR pAtients coVId19 Disease (GRAVID)	1230	Observational
NCT04344015	COVID-19 Plasma Collection	1231	Clinical, not randomised
NCT04344106	Prone Positioning in Spontaneously Breathing Nonintubated Covid-19 Patient: a Pilot Study	1234	Clinical, not randomised
NCT04344119	Assessment of Chilbains Occuring During Covid-19 Infection	1235	Observational
NCT04344145	Exhaustion and Needs in Frontline COVID-19 Healthcare Workers: Cross-sectional Study in a Belgian Population	1236	Observational
NCT04344171	CovidDB: The Covid-19 Inpatient Database	1237	Observational
NCT04344197	Surgery During Covid-19: The Role of Asymptomatic Patients, a Survey	1239	Observational
NCT04344210	Impact of Tele-interventions During the COVID-19 Pandemic in Patients With Diabetes Mellitus	1240	No medical treatment intervention
NCT04344236	Gargling and Nasal Rinses to Reduce Oro- and Nasopharyngeal Viral Load in Patients With COVID-19	1241	No medical treatment intervention
NCT04344249	Cohort of Patients With Inflammatory Bowel Disease During COVID-19 Pandemic	1242	Observational
NCT04344327	COVID-19 in Cardiology Unit in France : Risk Factors for Mortality (CCF)	1244	Observational
NCT04344379	Prevention of SARS-CoV-2 in Hospital Workers s Exposed to the Virus	1245	Cancelled
NCT04344431	Management by Hyperbaric Oxygen Therapy of Patients With Hypoxaemic Pneumonia With SARS-CoV-2 (COVID-19)	1246	Clinical, not randomised
NCT04344457	Evaluate the Efficacy and Safety of Oral Hydroxychloroquine, Indomethacin and Zithromax in Subjects With Mild Symptoms of COVID-19	1248	Clinical, not randomised
NCT04344509	Microbial Etiology of Ventilator-associated Pneumonia in SARS-CoV-2 Infected Patients	1249	Observational
NCT04344548	Phase I / II Clinical Study of Immunotherapy Based on Adoptive Cell Transfer as a Therapeutic Alternative for Patients With COVID-19 in Colombia	1251	Clinical, not randomised
NCT04344587	COVID-19 smArtphone-based Trial of Non-ICU Admission Prone Positioning	1253	Observational
NCT04344600	Peginterferon Lambda-1a for the Prevention and Treatment of SARS-CoV-2 Infection	1254	Clinical, not randomised
NCT04344834	Impact of Covid-19 on Egyptian Health Care Workers and General Population Mental Health	1258	Observational
NCT04344912	Impact of COVID19 Outbreak in Cardiac Acute Care	1259	Observational
NCT04344925	Non Invasive Positive Pressure Ventilation to Minimize Aerosolization for COVID 19	1260	Observational
NCT04344938	Covid 19 Pandemic. Emerging Legal and Ehical Disputes Over Patient Confidentiality	1261	Observational
NCT04344951	Chloroquine Phosphate Against Infection by the Novel Coronavirus SARS-CoV-2 (COVID-19): The HOPE Open-Label, Non Randomized Clinical Trial	1262	Clinical, not randomised
NCT04344964	Endoscopy-related Phone Consultation During the COVID-19 Outbreak	1263	Observational
NCT04344977	COVID-19 Plasma Collection	1264	Observational
NCT04345159	Prevalence of COVID-19 Symptoms History in Patients With Autoimmune Diseases	1265	Observational
NCT04345276	Efficacy and Safety of Ganovo (Danoprevir) Combined With Ritonavir in the Treatment of SARS-CoV-2 Infection	1266	Clinical, not randomised
NCT04345315	Correlative Study on Cancer Patients and Healthcare Professionals Exposed to Severe Acute Respiratory Syndrome-Corona Virus-2 Infection	1268	Observational
NCT04345419	A Real-life Experience on Treatment of Patients With COVID 19	1270	Clinical, not randomised
NCT04345510	Testing for COVID-19 Infection in Asymptomatic Persons	1272	Observational
NCT04345536	Prospective Quality Register of Patients With Confirmed Covid-19 at Oslo University Hospital	1274	Observational
NCT04345549	Ayurveda Self-Management for Flu Like Symptoms During the Covid-19 Outbreak	1275	Clinical, not randomised
NCT04345601	Mesenchymal Stromal Cells for the Treatment of SARS-CoV-2 Induced Acute Respiratory Failure (COVID-19 Disease)	1276	Clinical, not randomised
NCT04345640	The Spectrum and Profile of COVID-19 Infection and Its Impact on Liver.	1278	Observational
NCT04345653	Hydroxychloroquine as Chemoprevention for COVID-19 for High Risk Healthcare Workers	1279	Clinical, not randomised
NCT04345679	Anti COVID-19 Convalescent Plasma Therapy	1280	Clinical, not randomised
SNCTP000003718	Décubitus ventral chez les patients hospitalisés pour pneumonie COVID-19 sous oxygénothérapie	1282	Clinical, not randomised
SNCTP000003725	Früherkennung von COVID-19 im Fürstentum Liechtenstein mittels einem Zyklus- & Fertilitätstracker	1283	No medical treatment intervention
SNCTP000003726	Studie über die Sicherheit und Wirksamkeit einer passiven Immunisierung von SARS-CoV-2 positiven Patienten mit Plasma von COVID-19 geheilten Patienten	1284	Clinical, not randomised
TCTR20200405001	Use of Covid-19 Rapid Test for Hospital Care: GI Procedure (Use of Covid-19 Rapid Test for Hospital Care: GI Procedure)	1286	No medical treatment intervention
NL8497	Lung ultrasound vs CT scan in COVID-19 disease	1288	No medical treatment intervention
NL8498	Lung ultrasound (LUS) as a tool to determine the clinical course in COVID-19 patients	1289	No medical treatment intervention
NL8501	Using eHealth to support COVID-19 self-assessment and collection of self-reported health data	1290	Observational
NL8504	Pre-emptive tocilizumab in hypoxic COVID-19 patients, a prospective randomized trial	1291	Duplicate
NL8512	The use of ACE2 receptor increasing medication at hospital admission and	1292	Observational
NL8513	COVID-19 in rheumatic patients: a prospective cohort study	1293	Cinical, not randomised
UMIN000040033	Nosocomial infection of coronavirus disease 2019 in the hospital without negative pressure chamber	1294	Observational
jRCT2031190264	A Multicenter, Adaptive, Randomized Blinded Controlled Trial of the Safety and Efficacy of Investigational Therapeutics for the Treatment of COVID-19 in Hospitalized Adults	1295	Duplicate
JapicCTI-205238	Phase 3 Clinical Study of Favipiravir in Patients with COVID-19 Non-Severe Pneumonia	1297	No medical treatment intervention
ACTRN12620000448943p	Expressive Writing To Combat Distress Associated With The COVID-19 Pandemic In People With Inflammatory Bowel Disease	1299	No medical treatment intervention
ACTRN12620000443998p	Home telerehabilitation for people with COVID-19: Implementing telehealth approaches to care and its effect on reintegration into the community	1300	Observational
ACTRN12620000438954p	Utilizing lung ultrasound in COVID-19 patients in ICU – comparison of management strategies on survival and time on ventilation	1301	No medical treatment intervention
EUCTR2020-001381-11	COVID-19 - ACORES-2 study: ACE inhibitors or ARBs discontinuation for Clinical Outcome Risk reduction in patients hospitalized for the Endemic Severe acute respiratory syndrome	1304	No medical treatment intervention
ISRCTN43900695	Testing an early online intervention for the treatment of disturbed sleep during the COVID-19 pandemic	1305	No medical treatment intervention
ISRCTN77353041	Cohort study for predicting severe disease courses in patients presenting for coronavirus testing to the University of Zurich COVID-19 Testing Center	1306	Observational
NCT04304690	COVID-19 Seroconversion Among Front Line Medical and Paramedical Staff in Emergency, Intensive Care Units and Infectious Disease Departments During the 2020 Epidemic	1307	Clinical, not randomised
NCT04315480	Tocilizumab for SARS-CoV2 (COVID-19) Severe Pneumonitis	1308	Clinical, not randomised
NCT04321369	Impact of Swab Site and Sample Collector on Testing Sensitivity for COVID-19 Virus in Symptomatic Individuals	1309	Observational
NCT04331106	Survey of the Anxiety Associated With the COVID-19 Pandemic	1310	Observational
NCT04324684	Prognostic Factors Keeping Track for Covid-19 Pneumonia	1311	Observational
ACTRN12620000468921	Effect of a Brief Problem Management Intervention on Covid 19-Related Anxiety and Depression	1312	No medical treatment intervention
ISRCTN10077335	Comparison of lung water in COVID-19 patients and other patients with acute respiratory failure	1313	Observational
ISRCTN15281137	A phase I/II study to determine efficacy, safety and immunogenicity of the candidate coronavirus disease (COVID-19) vaccine ChAdOx1 nCoV-19 in UK healthy adult volunteers	1314	Observational
ChiCTR2000031772	Impact of WeChat-based parenting education for children with autism spectrum disorder and their mothers during the epidemic of Coronavirus disease 2019 (COVID-19)	1315	Cinical, not randomised
ACTRN12620000470998	Virucidal pilot study of Nasodine¬Æ Antiseptic Nasal Spray (povidone-iodine 0.5%) in people with COVID-19 and confirmed nasal shedding of SARS-CoV-2 virus.	1316	Cinical, not randomised
ChiCTR2000031783	Investigation for Prevention Awareness against COVID-19	1317	Observational
ChiCTR2000031675	A medical records based retrospective analysis of maternal and infant outcomes in Cesarean delivery in Hangzhou non pneumonia Hospital during pandemic of Novel coronovirus	1318	Observational
NCT04290780	COVID-19 - Multicenter Study on Nosocomial Transmission of SARS-CoV-2 Virus	1319	Observational
researchregistry5451	Prediction of Peak and Termination of Novel Coronavirus Covid-19 Epidemic in Iran	1320	No medical treatment intervention
researchregistry5464	Understanding and modelling COVID-19 mortality and severity	1321	No medical treatment intervention
UMIN000039986	Risk factors of death from coronavirus disease 2019	1322	No medical treatment intervention
UMIN000039997	Prevention of COVID-19 infection in a medical institution and maintenance of medical system	1323	Cinical, not randomised
UMIN000040033	Nosocomial infection of coronavirus disease 2019 in the hospital without negative pressure chamber	1324	Observational
RBR-6m69fc	Therapeutic Pulmonary Telerehabilitation Protocol in patients affected by COVID-19, confined in their homes: Randomized Control Trial	1325	No medical treatment intervention
CTRI/2020/04/024482	A pan-india non-interventional virtual registry evaluating the prophylactic efficacy of different regimens against SARS-CoV2 infection (COVID-2019) in asymptomatic health care workers	1326	No medical treatment intervention
CTRI/2020/04/024413	Knowledge, attitude and fear of COVID-19 in Bangladesh	1327	No medical treatment intervention
CTRI/2020/04/024442	Screening for symptoms of COVID-19	1328	No medical treatment intervention
CTRI/2020/04/024473	Viral Infection and Respiratory illness Universal Study	1329	No medical treatment intervention
EUCTR2020-001281-11	Evaluation of the concentration/viral effect relationship of hydroxychloroquine in COVID-19 patients in the intensive care unit.	1330	Cinical, not randomised
EUCTR2020-001031-27	Treatment of non-severe confirmed cases of COVID-19 and chemoprophylaxis of their contacts as prevention strategy: a Cluster Randomized Clinical Trial (PEP CoV-2 Study)	1331	No medical treatment intervention
EUCTR2020-000890-25	Treatment of Coronavirus SARS-Cov2 Respiratory Infections with Hydroxychloroquine	1332	Cinical, not randomised
EUCTR2015-002340-14	Randomized, Embedded, Multifactorial, Adaptive Platform trial for Community-Acquired Pneumonia (REMAP-CAP)	1333	Clinical, not randomised
EUCTR2020-001039-29	EFFICIENCY IN MANAGEMENT OF ORGAN DYSFUNCTION ASSOCIATED WITH INFECTION BY THE NOVEL SARS-CoV-2 VIRUS (COVID-19) THROUGH A PERSONALIZED	1334	Cinical, not randomised
EUCTR2020-000919-69	Reducing health care workers absenteeism in SARS-CoV-2 pandemic by enhanced trained immune responses through Bacillus Calmette-Guérin vaccination, a randomized controlled trial	1335	Duplicate
EUCTR2020-001345-38	CHROLOQUINE PHOSPHATE AGAINST INFECTION BY THE NOVEL CORONAVIRUS SARS-CoV-2 (COVID-19): THE HOPE OPEN-LABEL, NON-RANDOMIZED CLINICAL TRIAL	1336	Cinical, not randomised
IRCT20200324046847N1	Study the Efficacy and Safety of SeptimebTM in the Treatment of COVID-19 Infection in Iranian Patients: A Clinical Trial (Case Series)	1337	Observational
IRCT20140911019125N6	Study the effect of intravenous injection of dental pulp mesenchymal stem cells in treatment of patients with COVID-19 pneumonia	1338	Observational
IRCT20200325046860N1	Evaluation of Convalescent Plasma as a Potential Therapy for COVID-19 infected patients	1339	Observational
IRCT20150704023055N2	To evaluate the effectiveness of hemoperfusion in patients with severe coronavirus disease 2019 (COVID-19)	1340	Observational
IRCT20170611034452N11	Investigating the effectiveness of psychological interventions on mental health of Kerman University of Medical Sciences's mental health in hospitals and reference clinics	1341	No medical treatment intervention
IRCT20151227025726N13	Evaluation the Efficacy and Safety of Tocilizumab in Patients with Novel Coronavirus (COVID-19)	1342	Observational
IRCT20200325046859N1	Evaluation of the efficacy of intravenous immunoglobulin (IVIg) in patients with severe COVID-19 (Before intubation phase) who have not responded to treatment with the standard three-drug	1343	Observational
IRCT20200325046860N2	Mesenchymal stem cell utilization in reducing complications and enhancing pneumonia healing in patients infected with 2019-nCoV (phase I clinical trial)	1344	Observational
IRCT20131113015393N6	Effect of Benson Relaxation on Quality of Life and job stress in nurses in intensive care units with Covid-19 patients	1345	No medical treatment intervention
IRCT20181030041503N1	Clinical trial comparing pregnancy rate in transfer of blastocyst vs cleavage stage in assisted reproductive technology	1346	Not COVID-19 study
IRCT201109067492N1	A compression of complications in Gomco and classic methods of circumcision	1347	Not COVID-19 study
EUCTR2020-001459-42	Ruxolitinib Treatment in Patients with Severe COVID-19 Infection. A Danish Safety and Efficacy Study.	1348	Cinical, not randomised
ACTRN12620000482965	Impact of high intensity interval training and aerobic training on stress, depression symptoms, anxiety and resilience in self-isolated people during the COVID-19 health emergency in Spain	1349	No medical treatment intervention
DRKS00021300	Cytokine adsorption in severe COVID-19 pneumonia requiring extracorporeal membrane oxygenation	1351	Duplicate
NCT03042143	Repair of Acute Respiratory Distress Syndrome by Stromal Cell Administration (REALIST) (COVID-19)	1352	Not COVID-19 study
NCT03891420	A Study to Evaluate the Safety, Pharmacokinetics and Antiviral Effects of Galidesivir in Yellow Fever or COVID-19	1353	Not COVID-19 study
NCT04345861	Hydroxychloroquine Plus Azithromycin Versus Hydroxychloroquine for COVID-19 Pneumonia (COVIDOC Trial)	1354	Cancelled
NCT04346355	Efficacy of Early Administration of Tocilizumab in COVID-19 Patients	1359	Cancelled
ChiCTR2000032010	Novel coronavirus pneumonia (COVID-19) Intelligent Assistant Analysis System:A Multi-center Clinical Research	1389	Clinical, not randomised
NCT04323761	Expanded Access Treatment Protocol: Remdesivir (RDV; GS-5734) for the Treatment of SARS-CoV2 (CoV) Infection (COVID-19)	1390	Clinical, not randomised
NCT04346277	Compassionate Use Open-Label Anti-CD14 Treatment in Patients With SARS-CoV-2 (COVID-19)	1391	Clinical, not randomised
NCT04346017	Evaluation of Interleukine 6 (and Other Cytokines and Inflammatory Markers) in COVID-19 Patients With a Systemic Inflammatory Response Syndrome	1392	Clinical, not randomised
NCT04347538	Impact of Nasal Saline Irrigations on Viral Load in Patients With COVID-19	1393	Clinical, not randomised
NCT04346589	Convalescent Antibodies Infusion in Critically Ill COVID 19 Patients	1394	Clinical, not randomised
NCT04346082	Feasibility of Online Based Mindfulness Interventions During COVID-19 Outbreak	1395	Clinical, not randomised
NCT04347681	Potential Efficacy of Convalescent Plasma to Treat Severe COVID-19 and Patients at High Risk of Developing Severe COVID-19	1396	Clinical, not randomised
NCT04348071	Safety and Efficacy of Ruxolitinib for COVID-19	1397	Clinical, not randomised
NCT04351906	Low-flow Extracorporeal Carbon Dioxide Removal in COVID-19-associated Acute Respiratory Distress Syndrome	1398	Clinical, not randomised
NCT04348877	Plasma Rich Antibodies From Recovered Patients From COVID19	1399	Clinical, not randomised
NCT04348474	Efficacy and Safety of Hydroxychloroquine and Azithromycin for the Treatment of Ambulatory Patients With Mild COVID-19	1400	Clinical, not randomised
NCT04348929	Birth Experience During COVID-19 Confinement	1401	Clinical, not randomised
NCT04350086	Use of Dexmedetomidine in Light to Moderate Sedation in the Patient in the Palliative Situation of a Sars-cov-2 / COVID-19 Infection	1402	Clinical, not randomised
NCT04350073	Longitudinal Energy Expenditure and Metabolic Effects in Patients With COVID-19 (LEEP-COVID)	1403	Clinical, not randomised
NCT04349631	A Clinical Trial to Determine the Safety and Efficacy of Hope Biosciences Autologous Mesenchymal Stem Cell Therapy (HB-adMSCs) to Provide Protection Against COVID-19	1404	Clinical, not randomised
NCT04350450	Hydroxychloroquine Treatment of Healthcare Workers With COVID19 Illness at Montefiore	1405	Clinical, not randomised
NCT04350476	COVID-19 Remote Monitoring	1406	Clinical, not randomised
NCT04351620	High-dose Hydroxychloroquine for the Treatment of Ambulatory Patients With Mild COVID-19	1407	Clinical, not randomised
ACTRN12620000492954	A Facebook delivered physical activity focused group lifestyle intervention for older adults during the COVID-19 pandemic	1408	Clinical, not randomised
NCT04351542	Ayurveda for Flu Like Illness During Covid-19 Outbreak	1409	Clinical, not randomised
ACTRN12620000477921	The design of an updated iron lung ventilator which can be easily built and used by people with minimal training in situations such as COVID-19 where skilled doctors or normal ventilators are	1410	Clinical, not randomised
ACTRN12620000472976	Adapting the Decathlon Group Easybreathe¬Æ snorkelling face mask for the safer administration of oxygen and/or continuous positive airway pressure and in the intra/interhospital transportation	1411	Clinical, not randomised
NL8521	Continuous positive airway pressure in severe Covid-19 pneumonia	1412	Clinical, not randomised
DRKS00021420	Observational clinical trial for the evaluation of an automated nasal and pharyngeal swab exam to test COVID-19 patients	1413	Clinical, not randomised
DRKS00021301	An e-Mental Health Intervention to Support burdened People in Times of the COVID-19 pandemic: CoPE It	1414	Clinical, not randomised
EUCTR2020-001765-37-ES	Pragmatic clinical trial of hydroxychloroquine in the treatment of oncohematological patients and health professionals with COVID-19 infection without radiological alteration.	1415	Clinical, not randomised
EUCTR2020-001466-11-GR	Prevention of severe respiratory failure in Covid-19	1416	Clinical, not randomised
ChiCTR2000032098	Danggui Shaoyao Powder in the synergistic treatment of novel coronavirus pneumonia (COVID-19)	1417	Clinical, not randomised
ChiCTR2000031955	Study for prevention of novel coronavirus pneumonia (COVID-19) in high risk population by Chinese medicine	1418	Clinical, not randomised
ChiCTR2000032013	The prevention and treatment strategy of the protective respirator related facial pressure injuries among healthcare professionals fighting novel coronavirus pneumonia (COVID-19)	1419	Clinical, not randomised
NCT04346420	Impact of the Double-Trunk Mask on Oxygenation Titration in COVID-19	1420	Clinical, not randomised
NCT04348864	COVID-19 Antibody Self-testing Using Virtual Point-of-care	1421	Clinical, not randomised
CTRI/2020/03/024402	Hydroxy Chloroquine, in open labelled, Randomised intervention for prevention of new infection and adverse outcomes following COVID-19 infection -A Tertiary Hospital based study	1426	Duplicate
CTRI/2020/04/024479	Study of the effect of Chloroquine in addition to standard therapy in COVID-19 patients	1427	Duplicate
EUCTR2020-001156-18-ES	Efficacy of different treatments in patients infected with COVID-19	1429	Duplicate
EUCTR2020-001160-28-ES	Tocilizumab plus Pembrolizumab in COVID-19	1430	Duplicate
EUCTR2020-001262-11-ES	Clinical trial to assess the efficacy and safety of cyclosporin A treatment associated with standard treatment versus standard treatment only in hospitalized patients with confirmed coronavirus	1432	Duplicate
EUCTR2020-001290-74-ES	Efficacy and safety of sarilumab in the early treament of hospitalized patients with mild-moderate neumonia and COVID19 infection versus standard of care	1434	Duplicate

Registry entry ID	Title	Database ID (ID all)	Reason for exclusion
EUCTR2020-001303-16-FR	Efficacy of Hydroxychloroquine, Telmisartan and Azithromycin on Survival in Elderly Hospitalized Patients with VIDOC-19 : A Randomized, Multi-Centre, Adaptive, Blinded Study	1435	Duplicate
EUCTR2020-001320-34-NL	A clinicial trial to investigate te effect of valsartan compared to placebo on acute respiratory failure in hospitalized SARS-CoV-2-infected patients	1436	Duplicate
EUCTR2020-001321-31-ES	Clinical trial phase II to evaluate the efficacy of 3 types of treatment in patients with pneumonia by COVID-19	1437	Duplicate
EUCTR2020-001331-26-GB	Preventative Drug Treatment for COVID-19 Infectious Disease	1438	Duplicate
EUCTR2020-001367-88-DK	Efficacy and safety of novel treatment options for adults with COVID-19 pneumonia	1439	Duplicate
EUCTR2020-001379-34-ES	Sedation with sevoflurane versus propofol in patients with Acute Respiratory Distress Syndrome caused by COVID-19 infection	1440	Duplicate
EUCTR2020-001413-20-ES	Efficacy and safety of siltuximab vs. corticosteroids in hospitalized patients with COVID-19 pneumonia	1442	Duplicate
EUCTR2020-001511-25-ES	Randomized open-blind controlled trial to study the benefit of Colchicine in Patients with COVID-19	1445	Duplicate
EUCTR2020-001530-35-ES	Clinical trial to avoid infection by SARS-CoV-2 in high-risk population. COVID-19	1446	Duplicate
EUCTR2020-001553-48-FR	Cortico√Ødes au cours de la pneumonie virale Covid-19 li√©e √† l‚Äôinfection par le SARS-Cov-2	1447	Duplicate
EUCTR2020-001602-34-FR	COvid-19 and Vitamin D supplementation: a multicenter randomized controlled Trial of high dose versus standard dose vitamin D3 in high-risk COVID-19 patients	1450	Duplicate
EUCTR2020-001634-36-ES	Randomized open pilot study to evaluate the efficacy of subcutaneous sarilumab in patients with moderate-severe COVID-19 infection.	1451	Duplicate
IRCT20160131026298N2	The effect of PHR spray on patients with coronavirus-19	1456	Observational
IRCT20190727044343N2	Atorvastatin effect on COVID-19	1460	Observational
JPRN-jRCTs031190269	A multicenter, open-label, randomized controlled trial to evaluate the efficacy and safety of inhaled ciclesonide for asymptomatic and mild patients with COVID-19	1479	Duplicate
CTRI/2020/04/024659	Study of Shreepad Shree Vallabh SSV Formulation to improve immunity in quarantine patients of COVID-19	1480	Clinical, not randomised
EUCTR2020-001324-33-FR	Use of a respiratory multiplex PCR and procalcitonin to reduce antibiotic exposure in patients with severe confirmed COVID-19 pneumonia	1483	No medical treatment intervention
NCT04346927	Telerehabilitation for Patients Diagnosed With Coronavirus	1491	No medical treatment intervention
NCT04346953	Video-Based Exercise for Individuals With Social Isolation Due to Coronavirus (COVID-19)	1492	No medical treatment intervention
ACTRN12620000480987	Stress-reduction Using Probiotics to Promote Ongoing Resilience Throughout COVID-19 for Healthcare Workers (SUPPORT COVID-19 Healthcare Workers)	1493	No medical treatment intervention
NCT04351789	Impact of a Minimal Psychoeducational Intervention on Anxiety Among Hospitalized COVID-19 Patients in Denmark	1494	No medical treatment intervention
DRKS00021153	get.calm and move.on ‚Äì tackling worries and finding strength for the crisis (COVID-19) in 10 steps	1495	No medical treatment intervention
EUCTR2020-001448-24-GB	Preventing Pulmonary Complications in Surgical Patients at Risk of COVID-19	1496	No medical treatment intervention
ChiCTR2000031888	A medical records based study for "Guangdong Pneumonia NO.1" in the Treatment of Novel Coronavirus Pneumonia (COVID-19)	1497	Observational
NCT04324736	COVID-19 and Diabetes Outcomes	1498	Observational
NCT04327804	A Longitudinal Study of COVID-19 Positive Patients Testing Nasal Swabs and Collecting Blood Samples for Research	1499	Observational
NCT04346043	To Access the Egyptian COVID-19 Whole Genome by NGS and Compare to the International Worldwide Database	1500	Observational
NCT04346212	Oropharyngeal Dysphagia in Patients With COVID-19	1501	Observational
NCT04346121	Non-COVID Patient-safety Incidents Reported by General Practitioners in Time of Lockdown: Protocol for a Cross-sectional Descriptive Study.cRisis	1502	Observational
NCT04346160	A Study to Assess the Virus, RNA, and miRNA Levels Related to Viral Infection, and Inflammatory Response in Tears of Patients Affected by COVID-19 Disease	1503	Observational
NCT04346186	COVID-19: Investigation of Transmission and Immunisation Among Hospital Staff	1504	Observational
NCT04348214	Screening & Risk Assessment of Healthcare Workers & Infection Control in University & COVID-19 Quarantine Hospitals	1505	Observational
NCT04346342	PRactice of VENTilation in COVID-19 Patients (PRoVENT-COVID)	1506	Observational
NCT04346056	Retrospective Study on Severe Respiratory Illness to Access the Presence of COVID-19	1507	Observational
NCT04348396	Clinical and Biological Predictors of COVID-19 Disease in Older Patients	1508	Observational
NCT04346810	Burnout Among Caregivers Facing COVID-19 Health Crisis at a Non-conventional Intensive Care Unit Compared to a Conventional Intensive Care Unit	1509	Observational
NCT04348422	Evaluating the Immune Response for COVID-19	1510	Observational
NCT04347070	Implementation of Physiotherapy on COVID-19 Patients in ICU	1511	Observational
NCT04346264	CoVid-19 - Infection and Antibody Formation in the Viennese Population	1512	Observational
NCT04347408	Rapid Diagnostics, Antibody Testing and Host Response in Children With COVID-19	1513	Observational
NCT04347369	A Retrospective Study of Neural Network Model to Dynamically Quantificate the Severity in COVID-19 Disease	1514	Observational
NCT04351646	Diagnostics of COVID-19/DARTS (Development and Assessment of Rapid Testing for SARS-CoV-2 Outbreak)	1515	Observational
NCT04351659	Novel Adoptive Cellular Therapy With SARS-CoV-2 Specific T Cells in Patients With Severe COVID-19	1516	Observational
NCT04347850	A Cohort of Patients With Possible or Confirmed SARS-CoV-2 (COVID-19)	1517	Observational
NCT04347876	Outcome of COVID-19 Cases Based on Tuberculin Test: Can Previous BCG Alter the Prognosis?	1518	Observational
NCT04347460	Establishing a COVID-19 Prospective Cohort for Identification of Secondary HLH	1519	Observational
NCT04347694	Study to Determine the Immunization Status Among Nephrological Health Care Personnel Against SARS-CoV-2 in a Single Center Over the Course of 12 Months	1520	Observational
NCT04347928	Respiratory and Hemodynamic Changes Induced in Mechanically Ventilated Patients for COVID-19	1521	Observational
NCT04347824	Predict Adverse Events by Covid-19 Nephritis	1522	Observational
NCT04348552	Non-COVID Patient-safety Incidents Reported by Primary Care Professionals in Time of Lockdown: Protocol for a Cross-sectional Descriptive Study	1523	Observational
NCT04348240	Transmissibility and Viral Load of SARS-CoV-2 in Oral Secretions	1524	Observational
NCT04348448	Observational Study, Use of Canakinumab Administered Subcutaneously in the Treatment COVID-19 Pneumonia	1525	Observational
ACTRN12620000493943	Study evaluating Front-line health care workers serial blood COVID-19 antibody results in relation to symptom presentation	1526	Observational
NCT04349202	Beaumont Health Large-scale Automated Serologic Testing for COVID-19	1527	Observational
ISRCTN18348009	Occupational health and safety among health care personnel in a COVID-19 pandemic	1528	Observational
NCT04350099	Emotional Burden of Healthcare Professionals and Covid Infection 19	1529	Observational
NCT04351399	Psychological Impact of Quarantine in Rheumatoid Arthritis Patient During COVID-19 Outbreak	1530	Observational
NCT04351711	Immunological Profiling of Patients With COVID-19 in Respiratory Distress	1531	Observational
ACTRN12620000486921	AUSTRALIAN CARDIOVASCULAR COVID-19 REGISTRY (AUS-COVID)	1532	Observational
ACTRN12620000474954	Airway Pressure Release Ventilation for mechanically ventilated patients with COVID-19 in Western Australian Intensive Care Units: an observational study	1533	Observational
NCT04351633	Psychological Impact of Quarantine in Osteoporosis Patient During COVID-19 Outbreak	1534	Observational
NCT04351139	Impact of the COVID-19 Pandemic in Gynecological Oncology	1535	Observational
ISRCTN51255782	Can the Ava fertility tracker device detect early signs of COVID-19?	1536	Observational
NCT04351802	CORonavirus (COVID-19) Diagnostic Lung UltraSound Study	1537	Observational
ACTRN12620000487910	Study evaluating Coronavirus (COVID-19) nasal/oral swab results compared to blood antibody results in COVID-19 clinic patients.	1538	Observational
ACTRN12620000479909	Mental health and wellbeing on general population during the COVID-19 outbreak	1539	Observational
ISRCTN80453162	The impact of the COVID-19 pandemic on the provision, practice, and outcomes of vascular surgery (COVER study)	1540	Observational
DRKS00021225	COVID-19 Registry of the LMU Hospital Munich	1541	Observational
DRKS00021220	Evaluation of the performance of novel rapid diagnostics for COVID-19 at point-of-care	1542	Observational
DRKS00021395	The effects of the COVID-19 pandemic on mental health	1543	Observational
DRKS00021166	Development of a test to measure the immune response in patients with Covid-19	1544	Observational
DRKS00021231	Accompanying monitoring of primary care in GP practices during the COVID-19 pandemic	1545	Observational
DRKS00021254	Investigation of the influence of the new coronavirus pandemic (COVID-19) on pain, mood and everyday life of patients with painful polyneuropathy	1546	Observational
DRKS00021206	COVID-19 Registry Freiburg	1547	Observational
DRKS00021161	Demographic and epidemiological characteristics of Covid-19 disease in Helios-Hospital and determinants of clinical course	1548	Observational
DRKS00021208	COVID-19 related obstetric and neonatal outcome study (CRONOS)	1549	Observational
DRKS00021164	Psychological distress of the COVID-19 pandemic: A comparison between cancer patients and healthy controls	1550	Observational
DRKS00021306	Covid-19 Case-Cluster-Study	1551	Observational
DRKS00021186	prospective observational study to evaluate transpulmonary pressure, end expiratory lung volume and intraabdominal pressure in mechanical ventilated patients with Covid-19	1552	Observational
DRKS00021145	Lean European Open Survey on	1553	Observational
CTRI/2020/04/024442	Screening for symptoms of COVID-19	1554	Observational
DRKS00021152	COVID-19 Contact Study - Immunoreaction after possible SARS-CoV-2 / COVID-19 contact	1555	Observational
DRKS00021289	Study on SARS-CoV-2-Serology (COVID-19) in Bremen's public sector	1556	Observational
DRKS00021268	VOICE: Online-Survey on distress and mental resources of medical health care workers during Covid-19-pandemic	1557	Observational
DRKS00021270	Prospective molecular biological and serological examination for the detection of SARS-CoV-2 (COVID-19) in employees of a hospital of regular care in Germany	1558	Observational
DRKS00021276	Dynamics of regional lung aeration non-invasive determined by electrical impedance tomography in patients with COVID 19	1559	Observational
CTRI/2020/04/024413	Knowledge, attitude and fear of COVID-19 in Bangladesh	1560	Observational
CTRI/2020/04/024636	Assessment of Objective clinical scoring system to rule out COVID-19 with high sensitivity	1561	Observational
CTRI/2020/04/024473	Viral Infection and Respiratory illness Universal Study	1562	Observational
LBCTR2020043459	The effect of confinement on the mental health of individuals during the COVID-19 pandemic: an online survey	1563	Observational
JPRN-UMIN000039997	Prevention of COVID-19 infection in a medical institution	1564	Observational
JPRN-UMIN000040162	Multicenter observational study to examine the efficacy of protection during endoscopy examination for Covid-19 infection	1565	Observational
JPRN-UMIN000040185	Multicenter prospective cohort study evaluating outcome of COVID-19 in patients with hepatobiliary-pancreatic disease	1566	Observational
NCT04325048	Clinical Evaluation of Cordio Application in Adult COVID-19 Virus Positive Patients	1567	Observational
NCT04347993	A Prospective "Universal" Observational Database for COVID-19	1568	Observational
NCT04347278	Observational Study of Follow-up of Hospitalized Patients Diagnosed With COVID-19 to Evaluate the Effectiveness of the Drug Treatment Used to Treat This Disease. COVID-19 Registry	1569	Observational
NCT04347798	IMPACT: IMPact of Antimalarials on Covid Infections in RAPPORT	1570	Observational
NCT04348227	How COVID-19 Virus Outbreak Affects Antimicrobial Resistance in a Low-middle-income Country's ICU?	1571	Observational
NCT04348942	A COVID-19 Symptom, Exposure and Immune Response Registry	1572	Observational
NCT04349982	ICU Trial in Critical Ill COVID-19 Patients	1573	Observational
NCT04351529	Austrian COVID-19 Registry	1574	Observational
ChiCTR2000032009	Clinical characteristics and risk factors of novel coronavirus pneumonia (COVID-19) patients with chronic liver disease	1575	Observational
ChiCTR2000031982	Clinical observation for the effect of Ke-Gan-Li-Yan oral liquid on the relief of laryngeal symptoms of novel coronavirus pneumonia (COVID-19) convalescence and suspected patients and other	1576	Observational
ChiCTR2000031896	Study for the influence of novel coronavirus pneumonia (COVID-19) on mental health of Chinese public hospital stuffs	1577	Observational
ChiCTR2000032095	Study for Healthy Behavior, Psychological Analysis and Psychological Reconstruction Strategies of Students Population Keeping in Home Under the Pandemic of Novel Coronavirus Pneumonia	1578	Observational
ChiCTR2000031928	Delineate the prevalence, risk factors, temporal distribution and epidemiological characteristics of hidden novel coronavirus (2019-nCoV) infection in the community	1579	Observational
ChiCTR2000031954	Medical Records Based Study for Maternal and Perinatal Outcomes of Women with novel coronavirus pneumonia (COVID-19)	1580	Observational
ChiCTR2000032040	A medical records based study for the curative effect of combined traditional Chinese and western medicine in the treatment of severe novel coronavirus pneumonia (COVID-19)	1581	Observational
ChiCTR2000031930	Analysis of clinical characteristics and therapeutic effect of 9 cases of novel coronavirus pneumonia (COVID-19)	1582	Observational
JPRN-UMIN000039986	Risk factors of death from coronavirus disease 2019	1583	Observational
JPRN-UMIN000040188	Chloroquine and hydroxychloroquine for treatment of COVID-19: systematic review of randomized and non-randomized controlled trial	1584	Observational
IRCT20180129038542N1	Early tracheostomy in COVID-19	1585	Clinical, not randomised
IRCT20200404046947N1	Effect of Methylprednisolone on treatment of COVID-19	1586	Clinical, not randomised
IRCT20200408046990N1	Evaluation of SinaCurcumin capsule efficacy as an supplement therapy for mild to moderate COVID-19 in Mashhad	1587	Clinical, not randomised
IRCT20200317046797N5	Treatment of COVID-19-induced cytokine storm by hemoperfusion	1588	Clinical, not randomised
IRCT20200406046968N1	The effect of IL-6 inhibitor (Tocilizumab) on the prognosis of covid-19 patients with acute respiratory failure hospitalized in Imam Reza Hospital in Tabriz	1589	Clinical, not randomised
IRCT20200411047030N1	The effect of melatonin on the quality of sleep in COVID-19 patients	1590	Clinical, not randomised
IRCT20190804044429N1	Interferon beta 1b in COVID-19	1591	Clinical, not randomised
IRCT20151228025732N51	Effect of Algomed, Menta longifolia, Chamomile, Althaea rosea, Malva sylvestris, Lepidium sativum supplements on the Severity and Consequences of Coronavirus 19 disease (COVID-19)	1592	Clinical, not randomised
NCT04350736	First in Human Study SAD and MAD Study of Inhaled TD-0903, a Potential Treatment for ALI Associated With COVID-19	1596	Not COVID-19 study
RBR-3cbs3w	Evaluation of Hydroxychloroquine for prevention of hospitalization and respiratory complications in patients with confirmed diagnosis or suspected infection by (COVID-19)	1604	Duplicate
RBR-9pgwfc	Psychological effects of social isolation in the coronavirus pandemic	1607	Observational
TCTR20200401002	Social, ethical and behavioural aspects of the novel coronavirus pandemic	1608	Observational
PACTR202004893013257	Preventing Pulmonary Complications in Surgical Patients at Risk of COVID-19	1610	Duplicate
researchregistry5535	Rapid Adoption of an Interdisciplinary Care Team Model for Surgical Residents Managing COVID-19: A Quality Improvement Study	1611	No medical treatment intervention
researchregistry5529	The Challenges Of Urgent Radical Sigmoid Colorectal Cancer Resection In A COVID-19 Patient; A Case Report	1612	Clinical, not randomised
RBR-658khm	Evaluation of patients treated by telemedicine in the Covid-19 pandemic in Brazil in a medical clinic in the supplementary health sector in São Paulo, Brazil	1613	No medical treatment intervention
ISRCTN17898730	Expressive writing for the reduction of psychological difficulties during the COVID-19 pandemic	1614	No medical treatment intervention
ISRCTN10666798	The COVID-19 Emergency Response Assessment Study: a survey of the psychological health of frontline doctors in the UK and Ireland during the coronavirus pandemic	1615	No medical treatment intervention
CTRI/2020/04/024804	Evaluation of safety and efficacy of convalescent plasma in COVID-19 patients	1618	Clinical, not randomised
CTRI/2020/04/024773	A clinical trial to study the effects of additional treatments for patients hospitalized	1619	Duplicate
CTRI/2020/04/024882	A study to assess the effectiveness of Ayurvedic formulation in addition to standard	1620	Cinical, not randomised
CTRI/2020/04/024846	A clinical trial to evaluate the safety and efficacy of Mycobacterium W in critically ill	1623	Duplicate
CTRI/2020/04/024706	Effect of convalescent plasma in COVID-19 patients	1624	Duplicate
CTRI/2020/04/024776	Novel Artificial Intelligence Algorithm to screen COVID-19 Patients from X-Ray , CTScan	1627	Cinical, not randomised
CTRI/2020/04/024857	Proving the efficacy of Homeopathic treatment in prevention and cure of COVID-19	1629	No medical treatment intervention
CTRI/2020/04/024858	To study the effectiveness of Ivermectin with standard of care treatment versus	1630	Cinical, not randomised
ChiCTR2000032456	A Randomized Controlled Trial for the Effects of Low-Oxygen Consumption Instruction on the Prognosis of Patients with Novel Coronavirus Pneumonia (COVID-19)	1631	Observational
NCT04352985	Evaluating the Use of Polymyxin B Cartridge Hemoperfusion for Patients With Septic Shock and COVID 19	1632	No medical treatment intervention
NCT04355494	SOLIRIS¬Æ (Eculizumab) Treatment of Participants With COVID-19	1633	No medical treatment intervention
NCT04355793	Expanded Access Program of Ruxolitinib for the Emergency Treatment of Cytokine Storm From COVID-19 Infection	1634	No medical treatment intervention
NCT04358211	Expanded Access to Convalescent Plasma to Treat and Prevent Pulmonary Complications Associated With COVID-19	1635	No medical treatment intervention
NCT04358588	Pulsed Inhaled Nitric Oxide for the Treatment of Patients With Mild or Moderate COVID-19	1636	No medical treatment intervention
NCT04360486	Treatment Of CORONAVIRUS DISEASE 2019 (COVID-19) With Anti-Sars-CoV-2 Convalescent Plasma (ASCoV2CP)	1637	No medical treatment intervention
ChiCTR2000032368	Investigation on the mental health status and intervention of the medical staff of the national rescue medical team in hubei province during the pandemic of novel coronavirus pneumonia (COVID-	1638	No medical treatment intervention
NCT04359251	Different PEEP Settings of COVID-19 Induced ARDS	1639	Cinical, not randomised
NCT04358614	Baricitinib Therapy in COVID-19	1640	Cinical, not randomised
NCT04356690	Etoposide in Patients With COVID-19 Infection	1641	Cinical, not randomised
NCT04352348	Cohort of Patients Infected With SARS-CoV2 (COVID-19) or Suspected of Being	1642	Cinical, not randomised
NCT04355351	Study of Immune Response During SARS-CoV-2 Infection - (COVID-19)	1643	Cinical, not randomised
NCT04357327	Rapid Salivary Test to Detect SARS-CoV-2 (COVID-19)	1644	Cinical, not randomised
NCT04360811	Assessment of Obstetric, Fetal and Neonatal Risks and Vertical SARS-CoV-2 Transmission During COVID-19 Pandemic	1645	Cinical, not randomised
NCT04354831	A Study Evaluating the Efficacy and Safety of High-Titer Anti-SARS-CoV-2 Plasma in Hospitalized Patients With COVID-19 Infection	1646	Cinical, not randomised
NCT04354870	COVID-19 PrEP HCW HCQ Study	1647	Cinical, not randomised

Registry entry ID	Title	Database ID (ID all)	Reason for exclusion
NCT04356508	COVID-19: A Pilot Study of Adaptive Immunity and Anti-PD1	1648	Cinical, not randomised
NCT04356677	Study to Evaluate the Safety and Efficacy of VIRAZOLE¬Æ in Hospitalized Adult Participants With Respiratory Distress Due to COVID-19	1649	Cinical, not randomised
NCT04352465	Efficacy and Safety of MTX-loaded Nanoparticles to Treat Severe COVID-19 Patients	1650	Cinical, not randomised
NCT04352803	Adipose Mesenchymal Cells for Abatement of SARS-CoV-2 Respiratory Compromise in COVID-19 Disease	1651	Cinical, not randomised
NCT04356833	Nebulised Rt-PA for ARDS Due to COVID-19	1652	Cinical, not randomised
NCT04355533	Immunity Against SARS-CoV2 in Children and Their Parents	1653	Cinical, not randomised
NCT04357782	Administration of Intravenous Vitamin C in Novel Coronavirus Infection (COVID-19) and Decreased Oxygenation	1654	Cinical, not randomised
NCT04359225	A Comparison of 3D and 2D Telemedicine During Covid 19	1655	No medical treatment intervention
NCT04359875	A Phone-call With a Student/General Practitioner Team to Impact Morbidity of Chronic Patients During COVID-19 Containment	1656	No medical treatment intervention
NCT04362358	Online Cognitive Behavioral Therapy (CBT) for Stress Disorders in Health Workers Involved in the Care of Patients During the Covid-19 Epidemic	1657	No medical treatment intervention
NCT04361838	The COVID-19 ICU PRAYER Study	1658	No medical treatment intervention
NCT04353674	Modulation of Hyperinflammation in COVID-19	1659	Cinical, not randomised
NCT04357730	STudy of Alteplase for Respiratory Failure in SARS-Cov2 (COVID-19)	1660	Cinical, not randomised
NCT04355052	Open Label Study to Compare Efficacy, Safety and Tolerability of Hydroxychloroquine Combined With Azithromycin Compared to Hydroxychloroquine Combined With Camostat Mesylate and to	1661	Clinical, not randomised
NCT04358406	Rhu-pGSN for Severe Covid-19 Pneumonia	1662	Clinical, not randomised
NCT04361474	Trial Evaluating the Efficacy of Local Budesonide Therapy in the Management of Hyposmia in COVID-19 Patients Without Signs of Severity	1663	Clinical, not randomised
ChiCTR2000032400	the efficacy and safety of high dose intravenous vitamin C in the treatment of novel coronavirus pneumonia (COVID-19): a prospective, randomize, controlled trial	1664	Cinical, not randomised
EUCTR2020-001640-26-GB	A clinical trial of nebulised recombinant tissue plasminogen activator (rt-PA) in patients with COVID-19 Acute Respiratory Distress Syndrome to decrease the duration of ventilatory support and	1665	Cinical, not randomised
EUCTR2020-001459-42-DK	Ruxolitinib Treatment in Patients with Severe COVID-19 Infection. A Danish Safety and Efficacy Study.	1666	Cinical, not randomised
EUCTR2020-001255-40-ES	Sarilumab Treatment In cytoKinE storm caused by infection with COVID-19.	1667	Cinical, not randomised
EUCTR2020-001536-98-ES	Prophylaxis of COVID-19 infection with hydroxychloroquine in healthcare	1668	Cinical, not randomised
EUCTR2017-001100-30-DE	Investigation of efficacy and tolerability of the healing water Staatl. Fachingen STILL in patients for symptomatic treatment of heartburn in comparison to placebo	1669	No medical treatment intervention
NCT04360837	PEEP Incremental and Decremental Alveolar Recruitment of Critically Ill Corona Virus Disease-19 (COVID-19) Patients	1670	Clinical, not randomised
NCT04361344	Neurodegeneration Markers and Neurological Course in Severe Covid-19 Infection	1671	Clinical, not randomised
NCT04361448	Performance of 3 Sampling Methods for the Detection of SARS-CoV-2 (COVID-19) With Real-time Reverse Transcriptase PCR	1672	Clinical, not randomised
NCT04361916	Home Care for Patients With COVID-19: Active Monitoring in Two Italian Health Units (Casale Monferrato and Torino)	1673	Clinical, not randomised
NCT04352751	Experimental Use of Convalescent Plasma for Passive Immunization in Current COVID-19 Pandemic in Pakistan in 2020	1674	Clinical, not randomised
NCT04355754	Ventil Device Usefulness in Mechanically Ventilated ICU Patients	1675	Clinical, not randomised
NCT04354610	Cardiovascular and Renal Biomarkers to Predict Acute Heart or Kidney Injury in Severe Covid-19 Infection	1676	Clinical, not randomised
NCT04361396	Assessment of the Presence of the SARS-COV-2 Virus in the Peritoneum of COVID-19 Patients	1677	Clinical, not randomised
NCT04354155	COVID-19 Anticoagulation in Children - Thromboprophlaxis (COVAC-TP) Trial	1678	Clinical, not randomised
NCT04355247	Prophylactic Corticosteroid to Prevent COVID-19 Cytokine Storm	1679	Clinical, not randomised
NCT04357366	suPAR-guided Anakinra Treatment for Validation of the Risk and Management of Respiratory Failure by COVID-19 (SAVE)	1680	Clinical, not randomised
NCT04359121	COVID-19 and the Developement of Phobic Fears of Disease	1681	Clinical, not randomised
NCT04354714	Ruxolitinib to Combat COVID-19	1682	Clinical, not randomised
NCT04353206	Convalescent Plasma in ICU Patients With COVID-19-induced Respiratory Failure	1683	Clinical, not randomised
NCT04356482	CONVALESCENT PLASMA FOR ILL PATIENTS BY COVID-19	1684	Clinical, not randomised
NCT04355897	CoVID-19 Plasma in Treatment of COVID-19 Patients	1685	Clinical, not randomised
NCT04358003	Plasma Adsorption in Patients With Confirmed COVID-19	1686	Clinical, not randomised
NCT04357106	COPLA Study: Treatment of Severe Forms of COronavirus Infection With Convalescent PLAsma	1687	Clinical, not randomised
NCT04358835	Keto-diet for Intubated Critical Care COVID-19	1688	Clinical, not randomised
NCT04359290	Ruxolitinib for Treatment of Covid-19 Induced Lung Injury ARDS	1689	Clinical, not randomised
NCT04361214	Leflunomide in Mild COVID-19 Patients	1690	Clinical, not randomised
ACTRN12620000516987	Testing a wellbeing app to support wellbeing during the Covid-19 pandemic	1691	No medical treatment intervention
ACTRN12620000500954	A reusable personalised ventilation hood: A phase 1 safety study of a new device (McMonty) in patients with suspected or confirmed COVID-19.	1692	No medical treatment intervention
NCT04351854	Registry for Clinical Presentation and Management of Patients With COVID-19 in the Emergency Room	1693	Observational
NCT04352491	An Online Survey to Understand Feelings Related to COVID-19 Among ILBS Patients With Liver Disease	1694	Observational
NCT04352699	Outcomes of Urological Surgery During Periods of Social COVID-19 Containemnt: is it Reasonable to Limit Access to Surgical Care for All?	1695	Observational
NCT04353154	The RAPID COVID TRIAGE Algorithm	1696	Observational
NCT04351503	A Systems Approach to Predict the Outcome of SARS-CoV-2 in the Population of a City; COVID-19	1697	Observational
NCT04353141	International Lung UltraSound Analysis (ILUSA) Study	1698	Observational
NCT04354272	Evaluation of Dental Emergency Treatments During COVID19 Crisis	1699	Observational
NCT04352517	Influence Physical Activity Psychological Responses COVID-19 Pandemic	1700	Observational
NCT04353323	COVID 19-Caregivers IGg Seroconversion	1701	Observational
NCT04352582	COVID-19 and Vaccination Attitudes	1702	Observational
NCT04352634	The Covid-19 HEalth caRe wOrkErS (HEROES) Study	1703	Observational
NCT04352842	Echocardiographic Manifestation in Patient With Coronavirus Disease 2019 (COVID-19)	1704	Observational
NCT04355338	Assessment of Seroprevalence and Incidence of SARS-CoV-2 Infection and COVID-19 in Brazil	1705	Observational
NCT04353011	Psychological Impact of Quarantine in Chronic Pain Patient During COVID-19 Outbreak	1706	Observational
NCT04354857	Qualitative and Quantitative Evaluation of Anosmia Over Time in Clinically Symptomatic Patients Tested for COVID-19 Infection	1707	Observational
NCT04355442	Evolution of Facial Trauma During COVID Containment Measures	1708	Observational
NCT04354753	AiM COVID for Covid 19 Tracking and Prediction	1709	Observational
NCT04354766	COVID-19 : Neutralizing Human Monoclonal Antibodies Against SARS-Cov-2	1710	Observational
NCT04353609	Covid-19 in Patients With Chronic Inflammatory Rheumatism, Auto-immune or Auto-inflammatory Rare and Non-rare Diseases	1711	Observational
NCT04355507	Computed Tomography for COVID-19 Diagnosis	1712	Observational
NCT04355611	Epidemiological Characteristics of COVID-19 in Patients With MS or NMO	1713	Observational
NCT04355702	Covid-19 in Lupus Patients	1714	Observational
NCT04354558	French Single Centre Experience of Critically Ill Patients With Covid 19	1715	Observational
NCT04355624	Kidney Involvement in COVID-19 Disease (COVKID)	1716	Observational
NCT04355884	Opportunistic Screening for Asymptomatic Left Ventricular Dysfunction in Coronavirus Disease 2019 (COVID19) Survivors	1717	Observational
NCT04355715	Combinaison of Chest Scanography and Nasal Viral Detection Test to Detect COVID-19 Positive Patients Before Surgical Intervention in a University Hospital During Coronavirus Pandemia	1718	Observational
NCT04355871	Characteristics and Outcomes of Hospitalized Patients With COVID-19 in Spain	1719	Observational
NCT04356443	Non-Invasive Monitoring of Respiratory Function in Spontaneously Breathing Patients With COVID-19 Infection	1720	Observational
NCT04355741	Gut Microbiota, "Spark and Flame" of COVID-19 Disease	1721	Observational
NCT04354584	Compartmental Inflammation in Mechanically Ventilated Patients With COVID-19	1722	Observational
NCT04356560	COVID-19 Surveillance of Patients and Healthcare Workers in a Hospital Department	1723	Observational
NCT04354792	Asymptomatic COVID-19 Infection Among Healthcare Workers	1724	Observational
NCT04356885	COVID-19 and Psychotic Symptoms in France	1725	Observational
NCT04356365	The Mental Health Status of the General Population During the COVID-19 Pandemic and Its Association With Adherence to Government-initiated Non-pharmacological Epidemiological	1726	Observational
NCT04356417	Long-term Use of Drugs That Could Prevent the Risk of Serious COVID-19 Infections or Make it Worse	1727	Observational
NCT04357418	Psychological Outcome of COVID-19 Lockdown on Psychiatric Hospital Staff and Close Relatives	1728	Observational
NCT04356950	Analysis of the Coagulopathy Developed by COVID-19 Infected Patients	1729	Observational
NCT04357574	Assessing the System for High-Intensity Evaluation During Radiotherapy During Changes in Response to COVID-19	1730	Observational
NCT04357535	Prognosis of Coronavirus Disease 2019 (COVID-19) Patients Receiving Receiving Antihypertensives	1731	Observational
NCT04357769	COVID-19 Pandemic Burden in Severe Mental Disease Patients	1732	Observational
NCT04355481	Dissemination of SARS-COV-2 (COVID-19) in the Environment of Infected Patients Admitted to Intensive Care Unit	1733	Observational
NCT04357041	COVID-19 Survey Study on Dietary Intake, Physical Activity and Overall Well-being	1734	Observational
NCT04357314	Myocardial Infarction Rates Overview During COVID-19 Pandemic In France: MODIF Study	1735	Observational
NCT04357834	WAVE. Wearable-based COVID-19 Markers for Prediction of Clinical Trajectories	1736	Observational
NCT04357431	COVID-19 Among Egyptian Health Care Providers	1737	Observational
NCT04358510	COVID-19 Mortality Prediction Model	1738	Observational
NCT04358029	Cardiac Arrhythmias In Patients With Coronavirus Disease (COVID-19)	1739	Observational
NCT04357496	COVID-19 Epidemic Response Study	1740	Observational
NCT04357886	Comparison of New Coronavirus (COVID-19) Awareness in Individuals of Different Age Groups: Analysis of Turkey	1741	Observational
NCT04358627	Dexmedetomidine to Improve Outcomes of ARDS in Critical Care COVID-19 Patients	1742	Observational
NCT04358536	Classification of COVID-19 Infection in Posteroanterior Chest X-rays	1743	Observational
NCT04357847	Assessment of Endothelial and Haemostatic Changes During Severe SARS-CoV-2 Infection	1744	Observational
NCT04359212	Increased Risk of Venous Thromboembolism and Higher Hypercoagulable State in Patients Recovered in Intensive Care Unit and in Medical Ward for Coronavirus Disease 2019 (COVID-19)	1745	Observational
NCT04359992	Study of Hemostasis in Case of Severe COVID-19	1746	Observational
NCT04358380	Liver Injury in Patients With COVID-19	1747	Observational
NCT04359459	Nasal Epithelial Genetic and Single Cell RNA COVID-19 Study	1748	Observational
NCT04358640	Anxiety and Work Resilience Among Tertiary University Hospital Workers During the COVID-19 Outbreak: An Online Survey	1749	Observational
NCT04359667	Serum IL-6 and Soluble IL-6 Receptor in Severe COVID-19 Pneumonia Treated With Tocilizumab	1750	Observational
NCT04359849	Sequencing and Tracking of Phylogeny in COVID-19 Study	1751	Observational
NCT04359836	A Study to Explore the Role of Gut Flora in COVID-19 Infection	1752	Observational
NCT04360850	Use of Telehealth Technology by Mental Health Care Professionals in Times of COVID-19 Pandemic	1753	Observational
NCT04360278	Plasma Collection From Convalescent and/or Immunized Donors for the Treatment of COVID-19	1754	Observational
NCT04360538	Long Term Outcomes of Patients With COVID-19	1755	Observational
NCT04361019	Physical Activity in University Students Around COVID-19 Confinement	1756	Observational
NCT04362150	Long-term Impact of Infection With Novel Coronavirus (COVID-19)	1757	Observational
NCT04360733	Precision Immunology to Determine the Immune Response in Patients With COVID-19	1758	Observational
NCT04362345	Clinical Features and Risk Factors Associated With Worse Outcome in Patients Hospitalized for Covid-19 Pneumonia	1759	Observational
NCT04359914	Neurocognitive Impairment in Patients With COVID-19	1760	Observational
NCT04360954	Evaluation of Antibody Tests for COVID-19	1761	Observational
NCT04360707	Solid Organ Transplant Recipients With Covid-19 French Registry	1762	Observational
NCT04361604	Clinical Characterisation Protocol for COVID-19 in People Living With HIV	1763	Observational
NCT04361786	Acral Cutaneous Thrombotic Vasculopathy and Covid-19 Infection	1764	Observational
NCT04361877	Cardiovascular Prevention During COVID-19 Pandemic Lockdown in Young Adults (COLA Trial)	1765	Observational
NCT04361123	Atrium COVID-19 Syndromic and Serologic Surveillance	1766	Observational
NCT04361903	Ruxolitinib for the Treatment of Acute Respiratory Distress Syndrome in Patients With COVID-19 Infection	1767	Observational
NL8529	General practice cooperative COVID-19 centre Urmond	1768	Observational
NL8535	Functional status and health status in COVID-19 patients pre and post rehabilitation	1769	Observational
NCT04361565	Frequency and Clinical Evolution of Olfactory and Taste Disorders in COVID-19 Patients	1770	Observational
ACTRN12620000498998	Retrospective Study on surgical outcomes among surgical patients during the COVID-19 (SARS-CoV-2) Pandemic	1771	Observational
NCT04362319	Burnout and Medical Errors in the Anaesthesiology Fraternity During Covid-19 Pandemic	1772	Observational
NL8551	COVID Immunosuppressive Treatment	1773	Observational
ACTRN12620000512921	SARS-CoV2 in children presenting to hospital: A repeating point prevalence study during the COVID-19 pandemic in South East Queensland	1774	Observational
NL8536	Neurological Manifestations of COVID-19 and Indications of an Association with Respiratory Failure	1775	Observational
NL8522	The Digital Doc in the Emergency Department: The solution for patient care in the Covid-19 pandemic?	1776	Observational
NL8528	Control of COVID-19 in hospitals	1777	Observational
NL8526	Neuroinvasion in COVID-19	1778	Observational
NL8543	COVID19 ultrasound triage	1779	Observational
NCT04092478	The Abdominal Crunch Position for COVID-19 Patients	1780	Observational
NCT04352764	ANTIBODY BASED TESTS FOR SARSCoV-2 COVID-19) - Evaluation of Patients and Healthcare Providers in the Confines of Healthcare Settings	1781	Observational
NCT04354519	The United Kingdom Multiple Sclerosis Register Covid-19 Substudy	1782	Observational
NCT04353583	Renal Outcome in Patients With COVID-19	1783	Observational
NCT04354701	COVID-19 and Cancer Consortium Registry	1784	Observational
NCT04354818	Coronavirus (COVID-19) Outcomes Registries in Immunocompromised Individuals Australia (CORIA)	1785	Observational
NCT04356586	Immune Response to Covid-19 in 300 Health Care Workers With Mild Symptoms	1786	Observational
NCT04357275	The RIsk Stratification in COVID-19 Patients in the ICU Registry	1787	Observational
NCT04358042	PSYCHIATRIC Disorders and Covid-19	1788	Observational
NCT04357639	Impact of Long-term Protease Inhibitors in Patients Living With HIV on the Incidence of COVID-19 (COVIP)	1789	Observational
NCT04359602	COVID-19 Recovered Volunteer Research Participant Pool Registry	1790	Observational
NCT04359927	Long-term Effects of Coronavirus Disease 2019 on the Cardiovascular System: CV COVID-19 Registry	1791	Observational
NCT04361500	Registry of COVID-19 Patients Treated With the Seraph 100 Microbind Affinity Blood Filter	1792	Observational
NCT04361591	COVID-19 in Liver Transplant Recipients	1793	Observational
NCT04361981	Influence of COVID-19 Infection in Thromboembolic Venous Disease: National Cohort Study	1794	Observational
ChiCTR2000032233	Clinical study for the potential impact of novel coronavirus pneumonia (COVID-19) on endocrine system	1795	Observational
ChiCTR2000032412	A medical records based retrospective study for the effectiveness and safety of Xi-Yan-Ping injection combined with conventional protocol in the treatment of common type novel coronavirus	1796	Observational
ChiCTR2000032239	A medical records based study for epidemiological characteristics of 88 patients with new coronavirus pneumonia (COVID-19) and its impact on severe disease	1797	Observational
ChiCTR2000032213	A medical records based study for the impact of wearing medical masks on oxygen saturation in adult surgical patients after general anaesthesia during novel coronavirus pneumonia (COVID-19)	1798	Observational
ACTRN12620000527965	Descriptive analysis and reporting of outcomes of neonates in COVID-19 confirmed infants or infants born to COVID-19 confirmed mothers.	1799	No medical treatment intervention
ACTRN12620000524998		1800	No medical treatment intervention
ChiCTR2000032161	A retrospective study for evolution and clinical outcomes study of novel coronavirus pneumonia (COVID-19) patients	1801	Observational

Registry entry ID	Title	Database ID (ID all)	Reason for exclusion
ChiCTR2000032162	A medical records based study for ECMO in the rescue therapy of extremely critical novel coronavirus pneumonia (COVID-19) patients	1802	Observational
ChiCTR2000032461		1803	Cinical, not randomised
ChiCTR2000032457	Application of pulmonary rehabilitation exercise in the treatment of novel coronavirus pneumonia (COVID-19) patients during convalescence period.	1804	Observational
ChiCTR2000032455	A study for the mental health status of novel coronavirus pneumonia (COVID-19) convalescent patients and first-line medical staff and Isolate residents and Hospital staffand the intervention	1805	No medical treatment intervention
ChiCTR2000032458	A medical records based retrospective study: Leukopenia is more common in asymptomatic patients less than 18 years old with novel coronavirus pneumonia (COVID-19) in convalescence	1806	Observational
ChiCTR2000032460	Early warning model and new biomarker of severe novel coronavirus pneumonia (COVID-19)	1807	No medical treatment intervention
NCT04363775	Comparison of VieScope vs. Macintosh Laryngoscope for Intubation in Level Cpersonal Protective Equipment Conditions	1808	No medical treatment intervention
NCT04366141	COVID-19 (Coronavirus Disease 2019) Intubation Barrier Box	1809	No medical treatment intervention
NCT04363541	Local Thermotherapy for Patients With Moderate Symptoms of COVID-19	1810	No medical treatment intervention
NCT04363177	Global Mental Health of Urban Mothers (MUM)	1811	No medical treatment intervention
NCT04346966	The Effectiveness of Video-Based Exercises in Young Adults	1812	No medical treatment intervention
NCT04346940	The Effectiveness of Telerehabilitation-Based Exercises in Elderly People	1813	No medical treatment intervention
NCT04346979	Telerehabilitation-based Yoga and Mindfulness Home Program in Postmenopausal Women Having Social Isolation	1814	No medical treatment intervention
NCT04363749	COVID-19 Infection: Exploration of Respiratory Control Center Abnormalities (CRC-COVID)	1815	Cinical, not randomised
NCT04363606	Chronic Fatigue Etiology and Recovery in Covid-19 Patients: the Role of Fatigability (FatCovid-19)	1816	Cinical, not randomised
NCT04363593	Serology COVID-19 From the Cornwall Hospital Union (ROCOCO)	1817	Cinical, not randomised
NCT04364802	COVID-19: Povidone-Iodine Intranasal Prophylaxis in Front-line Healthcare Personnel and Inpatients (PIIPPI)	1818	Cinical, not randomised
NCT04355234	Prevalence and Impact of COVID-19 Infection in Pregnant Women, Fetuses and Newborns (COVIPREG)	1819	Cinical, not randomised
NCT04364828	NGS Diagnostic in COVID-19 Hosts - Genetic Cause Relating to the Course of Disease Progression (COVID-19 NGS)	1820	Cinical, not randomised
NCT04363346	Study of FT516 for the Treatment of COVID-19 in Hospitalized Patients With Hypoxia	1821	Cinical, not randomised
NCT04365699	Cardiovascular Effects of COVID-19	1822	Cinical, not randomised
NCT04363034	Arkansas Expanded Access COVID-19 Convalescent Plasma Treatment Program	1823	No medical treatment intervention
NCT04364698	Observational Cohort of COVID-19 Patients at Raymond-Poincare (COVID-RPC)	1824	Observational
NCT04362865	Investigation of the B- and T-cell Repertoire and Immune Response in Patients With Acute and Resolved COVID-19 Infection	1825	Observational
NCT04362943	Clinical-epidemiological Characterization of COVID-19 Disease in Hospitalized Older Adults (COVID-AGE)	1826	Observational
NCT04365725	Multicenter, Retrospective Study of the Effects of Remdesivir in the Treatment of Severe Covid-19 Infections (REMDECO-19)	1827	Observational
NCT04365634	A Single-center Retrospective Study to Find Prediction Factors Related to Mortality of COVID-19 Patients.	1828	Observational
NCT04365816	NutriCovid30 : Nutritional Evaluation for Covid-19 Infection at D30	1829	Observational
NCT04363489	The CEDiD Study (COVID-19 Early Diagnosis in Doctors) (CEDiD)	1830	Observational
NCT04364984	ARB, ACEi, DRi in COVID-19 (BIRCOV)	1831	Observational
NCT04366154	Impact of the COVID-19 Infectious Epidemic on the Management of Oncology and Onco-hematology Patients and on the Psychological Consequences for Patients and Caregivers (COVIPACT)	1832	Observational
NCT04363008	COVID 19 Biomarkers	1833	Observational
NCT04366024	A Novel Nomogram to Predict Severity of COVID-19	1834	Observational
NCT04363099	Multicentre Observational Study on Management COVID 19 Positive Outpatients in a French Cluster. (COVID AMBU 60)	1835	Observational
NCT04349540	Role of Immune and Inflammatory Response in Recipients of Allogeneic Haematopoietic Stem Cell Transplantation (SCT) Affected by Severe COVID19 ((COVID19_BMT))	1836	Observational
NCT04364711	EuRopean Study of MAjor Infectious Disease Syndromes Related to COVID-19 (MERMAIDS 2)	1837	Observational
NCT04363671	Adolescents and Health Professionals Faced With the Necessity for Changing to Remote Care During the COVID-19 Outbreak Quarantine (AdoPro-Cov19) (AdoPro-Cov19)	1838	Observational
NCT04365959	The Prone Position in Covid-19 Affected Patients (PRON-COVID)	1839	Observational
NCT04362995	St. Jude Tracking of Viral and Host Factors Associated With COVID-19	1840	Observational
NCT04365764	Effect of Treatments in Patients Hospitalized for Severe COVID-19 Pneumonia: a Multicenter Cohort Study	1841	Observational
NCT04366206	Factors Associated With Clinical Outcomes in Patients Hospitalized for Covid-19 in GHT-93 Est	1842	Observational
NCT04362930	Cohort of Patients With Covid-19 Presenting Neurological or Psychiatric Disorders (CoCo-Neurosciences) (CoCo-Neuro)	1843	Observational
NCT04363268	ACCESS A Master Digital Surveillance Protocol for COVID-19 (ACCESS)	1844	Observational
NCT04362267	Longitudinal Study of Covid-19 Infection Among HCW in a French University Hospital (PRECOVIA)	1845	Observational
NCT04363632	Prospective Analysis of Morbi-mortality of Patients With Cancers in Active Phase of Treatment Suspected or Diagnosed of a SARS-CoV-2 Infection (ONCOVID-19)	1846	Observational
NCT04365595	SARS-CoV-2 Associated Respiratory Failure Recovery (COVID-19 CAir)	1847	Observational
NCT04362956	Clinical and Immunologic Impact of Perinatal SARS-CoV-2 (COVID-19) Infection	1848	Observational
NCT04365881	Loneliness During the NPIs for the COVID-19 Pandemic in Norway: Risk Factors and Associations With Psychopathology	1849	Observational
NCT04352556	COVID19-hematological Malignancies: the Italian Hematology Alliance	1850	Observational
NCT04359693	Impact of COVID-19 Infection on the Incidence of Ventilator-acquired Infections (COVAPID)	1851	Observational
NCT04359706	Bacterial and Fungal Microbiota of Patients With Severe Viral Pneumonia With COVID-19 (MICROVID)	1852	Observational
NCT04354779	Antibody Seroprevalence and Rate of Asymptomatic Infections With SARS-CoV-2 in Austrian Hospital Personnel	1853	Observational
NCT04357938	Risk Stratification With Chest CT to Rule-out Suspected SARS-CoV-2 Infections (SCout)	1854	Observational
NCT04360863	The Impact of COVID-19 Pandemic on the Smoking Behaviour of Young Smokers and the Youth Quitline Service in Hong Kong	1855	Observational
NCT04353401	WGS ANALYSIS OF SARS-COV-2 POSITIVE PATIENTS (CALYPSO)	1856	Observational
NCT04356378	Long Term Physical Functional Limitations in Daily Living in SARS-CoV2 Infected Patients Hospitalized in the Acute Phase Then Requiring Rehabilitation (CAPACoV19)	1857	Observational
NCT04365361	The Psychological Impact of the COVID-19 on Students.	1858	Observational
NCT04365348	The Psychological Impact of COVID-19 Outbreak on COVID-19 Survivors and Their Families	1859	Observational
NCT04362969	COVID-EC Study: Clinical Characteristics of Patients With COVID-19 in Guayaquil, Ecuador	1860	Observational
NCT04365335	Stress Biomarkers Leading to Professional Burnout Among People Involved in a Mobile Intensive Care Unit During the COVID-19 Pandemic (AUTONOMIC)	1861	Observational
NCT04366219	Impact of Confinement and Preventive Measures in Period of SARS-COV2 Infection on Clinical Features, Diagnostic and Therapeutic Management and Prognosis of Patients With Lung Cancer	1862	Observational
NCT04358952	Myocardial Involvement of Severe Acute Respiratory Syndrome-Cov-2 (Covid19) Infected Patients (COCARDE)	1863	Observational
NCT04365166	Study of Clinical and Immune Severity Profiles of Patients Infected With SARS-Cov2 (COVID-19) (REACOVIM)	1864	Observational
NCT04363333	Obstructive Sleep Apnea & Covid-19 Outcomes (OSACOVID-19)	1865	Observational
NCT04366167	Patient Recovery From Heart Surgery During the Covid-19 Pandemic (CardiacCovid)	1866	Observational
NCT04365530	Become of Patients Infected or Suspected of Being Infected by Covid-19 and Supported by the GHPSJ and the Establishments of the Paris Plaisance Hospital City	1867	Observational
NCT04349163	Resilience Evaluation of Caregivers During the SARS-CoV2 Epidemic Period : Prospective Cohort. (Resi-CoV)	1868	Observational
NCT04350710	Description of Respiratory Mechanics in Patients With SARS-CoV-2 Associated ARDS	1869	Observational
NCT04363788	Needle Stick Injuries in Emergency Medical Service Practice	1870	Observational
NCT04365322	Study of Specific Viral Immune Responses Induced by SARS-CoV-2 (COVID-19) (COV-CREM)	1871	Observational
NCT04363047	Immune Cells in Inflammatory Arthritis With Coronaviruses, Including COVID-19	1872	Observational
NCT04353245	Study of Biomarkers in the Long-term Impact of Coronavirus Infection in the Cardiorespiratory System (PostCOVID19)	1873	Observational
NCT04359407	Prone Positioning and Regional Ventilation in Mechanically Ventilated COVID-19 Patients (COVID-19_EIT)	1874	Observational
NCT04356144	Thrombomodulin-modified Thrombin Generation Assay (TGA-TM) in Patients With Critical Infections	1875	Observational
NCT04363203	VA Remote and Equitable Access to COVID-19 Healthcare Delivery (VA-REACH TRIAL) (VA-REACH)	1876	Clinical, not randomised
NCT04364594	COVID-19 Search in Conjunctival Cells (COVID-T)	1877	Clinical, not randomised
NCT04363385	Collection of Biological Samples With Clinical Characterization of Covid-19 Patients	1878	Clinical, not randomised
NCT04363528	Incidence of Deep Vein Thrombosis at Doppler Echo in Covid-19 Patients With SARS-Cov-2 Pneumopathy Hospitalized in ICU (COVIDOP-DVT)	1879	Clinical, not randomised
NCT04363853	Tocilizumab Treatment in Patients With COVID-19	1880	Clinical, not randomised
NCT04351919	Assessment of Efficacy and Safety of HCQ and Antibiotics Administrated to Patients COVID19(+) (COVID+PA)	1881	Clinical, not randomised
NCT04365439	Convalescent Plasma for COVID-19	1882	Clinical, not randomised
ChiCTR2000032165	A multicenter, randomized, double-blind, parallel-controlled trial for Qi-Mai-Fei-Luo-Ping Mixture in the improvement of lung function of novel coronavirus pneumonia (COVID-19) in the	1883	Observational
ChiCTR2000032205	A multicenter randomized, double-blind, placebo-controlled trial for Sheng-Mai-Yin for improvement of the pulmonary heart function related symptoms of convalescence patients of new	1884	Observational
ChiCTR2000032214	Screening of proprietary Chinese medicines in convalescent rehabilitation of novel coronavirus pneumonia (COVID-19) convalescent patients	1885	Observational
ChiCTR2000032237	A multicenter, randomized, double-blind, placebo-controlled trial for Xiang-Sha-Liu-Jun Pill in the treatment of novel coronavirus pneumonia (COVID-19) decline in digestive function during	1886	Observational
ChiCTR2000032313	Study for efficacy and safety of Jie-Xing-Jun-Zi granules in the Treatment of convalescent patients of novel coronavious pneumonia (COVID-19)	1887	Observational
ChiCTR2000032346	Lingnan Fire-Needle Therapy Improves the Quality of Life in General Population Under the Pandemic of novel coronavirus pneumonia (COVID-19): A Prospective, Randomized Controlled Trial	1888	No medical treatment intervention
ChiCTR2000032399	A multicenter, randomized, double-blind, placebo-controlled trial for Xiaoyao capsule in the improvement of sleep mood disorder of convalescence patients of novel coronavirus pneumonia	1889	Observational
EUCTR2018-004318-16-DK	STOP PIV - Phase III DAS181 Lower Tract PIV Infection in Immunocompromised Subjects	1890	Not COVID-19 study
NCT04351880		1891	No medical treatment intervention
NCT04357340	The Effects of Pulmonary Physiotherapy Treatments on Patients With COVID-19	1892	No medical treatment intervention
NCT04359264	Direct Income SupporT and Advice Negating Spread of Epidemic COVID-19: a Randomized Controlled Trial	1893	No medical treatment intervention
NL8538	Sleep position related to hospital length of stay in admitted COVID-19 patients	1894	No medical treatment intervention
NCT04365608		1895	No medical treatment intervention
NCT04365738		1896	No medical treatment intervention
NCT04366297		1897	No medical treatment intervention
DRKS00021522	Local and systemic markers of inflammation and circadian rhythmicity in COVID-19 disease and their role in the prediction of clinical outcome	1898	Observational
DRKS00021575	Covid-19 HOspitalized patients RegisTry	1899	Observational
JPRN-UMIN000040193	Evaluating the social function of people with dementia under the COVID-19 epidemic by the Japanese version of the social functioning in dementia scale	1900	Observational
JPRN-UMIN000040211	A Multicenter, Retrospective Study to Evaluate the Efficacy of Systemic Gluco-corticoid Against COVID-19	1901	Observational
JPRN-UMIN000040219	A prospective cohort study to follow the health status of medical staff engage in COVID-19	1902	Observational
JPRN-UMIN000040235	Examination of test accuracy of COVID-19 rapid antibody kit	1903	Observational
TCTR20200426002	Chula Covid-19 Rapid Test for Pandemic Control	1904	No medical treatment intervention
TCTR20200425001	Mental Health Circumstances Among Healthcare Workers and General Public Under the Pandemic Situation of COVID-19 (HOME-COVID-19)	1905	No medical treatment intervention
NL8520	SARS-CoV-2 Observational Study of patients with acute respiratory tract infection in European primary care	1906	Observational
NL8531	Humoral responses to SARS-CoV-2 infection in children	1907	Observational
NL8540	Lung ultrasound findings in patients with novel SARS CoV2	1908	Observational
NL8544	COVID19 ultrasound diagnosis	1909	Observational
IRCT20200406046968N2	Efficacy of convalescent plasma transfusion of COVID-19 survivors on the treatment of respiratory failure of these patients	1910	Clinical, not randomised
IRCT20200325046859N2	evaluation of the effects of the 2-drug diet (hydroxychloroquine + umifenovir(Arbidol)) compared with Hydroxy chlorquine on the of mortality rate in patients with moderate symptoms of COVID-	1911	Cinical, not randomised
IRCT20200416047099N1	Use of convalescent plasma in the treatment of patients with severe COVID-19 pneumonia	1912	Cinical, not randomised
IRCT20200414047072N1	Experimental Use of COVID-19 Convalescent Plasma for the Purpose Of Passive Immunization in Current COVID-19 Pandemic	1913	Cinical, not randomised
IRCT20190717044241N2	Cell therapy in patients with coronavirus19 using mesenchymal stem cells	1914	Cinical, not randomised
IRCT20181104041551N1	Investigating Effect of Convalescent Plasma on COVID-19 Patients Outcome: A Clinical Trial	1915	Cinical, not randomised
EUCTR2020-001722-66	Plasma turnover in patients with COVID-19 disease and invasive mechanical ventilation: a randomized study	1916	Clinical, not randomised
EUCTR2020-001364-29	Phase I/II clinical trial to evaluate the safety and efficacy of Allogenic Adipose Tissue-Derived Mesenchymal Stem Cells Expanded in patients with severe COVID-19 pneumonia	1917	Clinical, not randomised
EUCTR2020-001411-25	A Phase 2 Randomized Single-Blind Study to Evaluate the Activity and Safety of Low Dose Oral Selinexor (KPT-330) in Patients with Severe COVID-19 Infection	1918	Duplicate
EUCTR2020-001541-39	Pilot study of single-dose bevacizumab as a treatment for acute respiratory distress syndrome in patients with COVID-19	1919	Clinical, not randomised
EUCTR2020-001823-15	Evaluation of the concentration-effect relationship of enoxaparin for thromboembolic prevention in COVID-19 resuscitation patients. COV-ENOX study	1920	Clinical, not randomised
EUCTR2020-001370-30	Phase 3 multicenter, randomized, double-blind, placebo-controlled study to assess the efficacy and safety of canakinumab on cytokine release syndrome in patients with COVID-19-induced	1922	Duplicate
SNCTP000003732	Etude sur l'efficacité d'une prophylaxie pour les personnes exposées au SARS-CoV-2 en Suisse- l'étude COPEP	1923	Other
SNCTP000003733	Maladie causée par le nouveau coronavirus (COVID-19 ou COVID)	1924	Duplicate
SNCTP000003735	Riech- und Schmeckstörungen bei COVID-19 Patienten	1925	Other
SNCTP000003736	Traitement précoce par hydroxychloroquine pour réduire les hospitalisations secondaires et la transmission aux proches contacts à domicile de SARS-CoV-2	1926	Other
IRCT20200414047070N1	Determining the effect of skin test purified protein derivative (PPD) on the recovery process of patients with COVID 19 in patients with this disease	1927	No medical treatment intervention
EUCTR2020-001172-15-AT	Recombinant human angiotensin-converting enzyme 2 (rhACE2) as a treat-ment for patients with COVID-19	1936	Duplicate
EUCTR2020-001302-30-AT	A multicenter, randomized, active controlled, open label, platform trial on the efficacy and safety of experimental therapeutics for patients with a lung disease caused by coronavirus infection	1938	Duplicate
EUCTR2020-001327-13-AT	A SINGLE-BLINDED RANDOMIZED, PLACEBO-CONTROLLED PHASE II TRIAL OF PROPHYLACTIC TREATMENT WITH ORAL AZITHROMYCIN VERSUS PLACEBO IN	1939	Duplicate
EUCTR2020-001329-30-AT	Therapy with Inhaled Nitric Oxide for Patients Infected with Coronavirus Suffering form Lung Failure	1940	Duplicate
EUCTR2020-001363-85-DK	Medication for prevention of Corona-virus for Danish nursing home residents	1941	Cancelled
EUCTR2020-001366-11-IT	An international randomised trial of additional treatments for COVID-19 in hospitalised patients who are all receiving the local standard of care	1942	Duplicate
EUCTR2020-001469-35-PL	A multicenter, open, randomized, non-commercial study to evaluate the efficacy and safety of chloroquine phosphate in the outpatient treatment of COVID-19 in combination with telemedicine	1948	Duplicate
EUCTR2020-001531-27-ES	Clinical trial of Sarilumab in adults hospitalized with COVID-19	1950	Duplicate
NCT04347512		1958	Cancelled
NCT04353596	Stopping ACE-inhibitors in COVID-19	1978	Duplicate
NCT04354441	Effect of Hydroxychloroquine in COVID-19 Positive Pregnant Women	1982	Cancelled
NCT04361552	Tocilizumab for the Treatment of Cytokine Release Syndrome in Patients With COVID-19 (SARS-CoV-2 Infection)	2042	Cancelled
NCT04361942	Treatment of Severe COVID-19 Pneumonia With Allogeneic Mesenchymal Stromal Cells (COVID_MSV)	2044	Duplicate
NCT04362137		2049	Duplicate
NCT04362332	Chloroquine, Hydroxychloroquine or Only Supportive Care in Patients AdmItted With Moderate to Severe COVID-19	2052	Cancelled
NL8547	REDUCING HOSPITAL ADMISSION OF ELDERLY IN SARS-CoV-2 PANDEMIC VIA THE INDUCTION OF TRAINED IMMUNITY BY BACILLUS CALMETTE-GU√ãRIN	2091	Duplicate
IRCT20130812014333N147	Comparative assessment of the efficacy and safety of addon treatment with "Sofosbuvir-Daclatasvir", "Lithium",and "Trifluoprazine" to "standard of care in three groups of patients with	2104	Duplicate
EUCTR2020-001544-26	Effects of discontinuing renin-angiotensin system inhibitors in patients with COVID-19	2110	Duplicate
EUCTR2020-001734-36	Efficacy and safety of ANAkinra during Adult « COVID-19 » with Aggravating respiratory symptoms: a multicenter open-label controlled randomized trial	2111	Duplicate
EUCTR2020-001616-18	Treatment with inhaled corticoids in patients with COVID-19 admitted to hospital with pneumonia	2113	Duplicate
EUCTR2020-001505-22	Double-blind, randomized, parallel, placebo-controlled pilot clinical trial, nested in a prospective cohort observational study, for the evaluation of the efficacy and safetyof two doses of WJ-MSC	2115	Duplicate
ChiCTR2000032476	Analysis of clinical features of novel coronavirus pneumonia (COVID-19): a medical records based study	2116	Observational
ChiCTR2000032478	TCM Intervention study for novel coronavirus pneumonia (COVID-19) patient during home/designate-unit isolation based on community prevention and control	2117	Observational
ChiCTR2000032480	Study on data management and diagnosis-treatment mode of TCM intervention for novel coronavirus pneumonia (COVID-19) convalescence	2118	Observational
ChiCTR2000032514	Direct evidence of rectal cancer patient infected with novel coronavirus pneumonia (COVID-19)	2119	Observational
ChiCTR2000032527	Developing and validating of clinical prediction model for novel coronavirus pneumonia (COVID-19) patients progressing to critical type, acute respiratory distress syndrome and death: a medical	2120	Observational
ChiCTR2000032574	Diagnostic value of chest CT in outpatient for novel coronavirus pneumonia (COVID-19) compared to RT-PCR	2121	Observational
ChiCTR2000032632	The influence of COVID-19 pandemic on cardiovascular patients	2122	Cinical, not randomised
DRKS00021255	Point-of-care coagulation testing in patients suffering from COVID-19	2123	Observational

Registry entry ID	Title	Database ID (ID all)	Reason for exclusion
DRKS00021273	Effects of digital home exercise programs on physical and mental well-being during the COVID-19 pandemic: a multi-center trial	2124	No medical treatment intervention
DRKS00021399	Prospective gas chromatographic and spectrometric analysis of the exhalation of healthy test persons and of SARS-CoV2 infected patients as well as of patients suffering from COVID-19 at the	2125	Observational
DRKS00021416	SARS-CoV-2 in tears in patients with COVID-19	2126	Observational
DRKS00021439	COVID-19 associated brainstem encephalitis - is SARS-CoV-2 a neurotropic agent?	2127	Observational
DRKS00021443	Feasibility and usability of video-based physical therapy for older people in the context of the COVID-19 pandemic	2128	Observational
DRKS00021506	Registry of hospitalized pediatric Patients mit SARS-CoV-2 infection (COVID-19)	2129	Observational
DRKS00021514	Database for diagnosis and therapy of COVID-19 infected patients	2130	Observational
DRKS00021516	Dysosmia and Dysgeusia in COVID-19 patients	2131	Observational
DRKS00021521	Prevalence of COVID-19 in children aged 1-10 years in Baden-W√ºrttemberg	2132	Observational
DRKS00021523	The influence of proton pump inhibitor therapy on the clinical course of COVID-19	2133	Observational
DRKS00021527	Evaluation of online support during the coronavirus (COVID-19) pandemic: a group chat program in the psychiatric outpatient clinic of the Leipzig University Hospital	2134	Clinical, not randomised
DRKS00021537	Long term Analysis and Evaluation of morphologic and clinical Parameters in COVID-19 Patients: prospective development of a practical risk score	2135	Observational
DRKS00021573	Impact of pantoprazole on absorption and disposition of hydroxychloroquine, a drug used in Covid-19	2136	Not COVID-19 study
DRKS00021578	Validation of new detection methods for SARS CoV-2 in suspected COVID-19	2137	Observational
DRKS00021581	Alterations in inflammatory and coagulation variables in COVID-19 patients	2138	Observational
DRKS00021585	Lung Ultrasound in Covid-19 Pneumonia	2139	Cinical, not randomised
DRKS00021591	Identification of predictive biomarkers for severe progression of COVID-19	2140	Observational
DRKS00021605	COVID-19 in an academic teaching hospital in Konstanz/Germany - evaluation of the "double rule out - single rule in" concept (DURO-SIN)	2141	Observational
DRKS00021612	CORRECT: COvid (Covid-19) Registry RECHts der Isar intensive care Trial	2142	Observational
ISRCTN68026880	A global registry of women affected by COVID-19 in pregnancy and their babies, to guide treatment and prevention	2143	Observational
NCT03648372	A Study to Evaluate the Safety, Tolerability and Pharmacokinetics (PK) of TAK-981 in Adult Participants With Advanced or Metastatic Solid Tumors or Relapsed/Refractory Hematologic	2144	Clinical, not randomised
NCT04278404	Pharmacokinetics, Pharmacodynamics, and Safety Profile of Understudied Drugs Administered to Children Per Standard of Care (POPS)	2145	Observational
NCT04365972	Attention Bias Modification for Reducing Health Anxiety During the Coronavirus Pandemic	2146	Clinical, not randomised
NCT04366752	Thrombo Embolic Events in Critical Care Patients With Covid-19 Serious Acute Pneumopathy	2147	Observational
NCT04366765	COVID-19 Survival - The COVIVA Study	2148	Observational
NCT04366778	Thrombosis and Covid-19	2149	Observational
NCT04366791	Radiation Eliminates Storming Cytokines and Unchecked Edema as a 1-Day Treatment for COVID-19	2150	Clinical, not randomised
NCT04366817	Psychological Impact of the Lockdown on Patients Giving Birth During the COVID-19 Epidemic Short Title : Isolement and Childbirth: Psychological Impact	2151	Clinical, not randomised
NCT04366830	Intermediate-size Expanded Access Program (EAP), Mesenchymal Stromal Cells (MSC) for Acute Respiratory Distress Syndrome (ARDS) Due to COVID-19 Infection	2152	Observational
NCT04366882	Minimal Invasive Autopsies to Investigate Changes in Deceased COVID-19 Patients	2153	Clinical, not randomised
NCT04366921	European/Euro-ELSO Survey on Adult and Neonatal/ Pediatric COVID-19 Patients in ECMO	2154	Observational
NCT04366934	Study of the Pathogenesis of Olfactory Disorders in COVID-19	2155	Observational
NCT04366947	Intravascular Access in Suspected/Confirmed COVID-19 Patient	2156	No medical treatment intervention
NCT04366986	International Registry of Coronavirus (COVID-19) Exposure in Pregnancy	2157	Observational
NCT04367064	To Study the Clinical Characteristics and Treatment Outcome of COVID-19 Patients Admitted in Hospitals of Max Healthcare (SCOPe Study)	2158	Observational
NCT04367129	Electrocardiogram Analysis in COVID-19 Patients	2159	Observational
NCT04367142	Rapid Detection of COVID-19 by Portable and Connected Biosensor	2160	Observational
NCT04367207	African Covid-19 Critical Care Outcomes Study	2161	Observational
NCT04367337	Health Behavior Change During COVID-19 Pandemic	2162	Observational
NCT04367350	Prospective Registry of Corona Virus Disease 2019 (Covid-19) Patients With Neuromuscular Involvement	2163	Observational
NCT04367363	Social Media and COVID-19	2164	Observational
NCT04367402	COST (COvid STudio) ACTION: Study for the Evaluation of Specific Antibodies Anti Covid-19 Linked to Covid-19 Infection, Symptoms and Genetic Expression of ACE2 Polymorphisms in	2165	Observational
NCT04367545	Development of a Molecular Diagnostic Strategy for SARS-CoV2 Based on Saliva in the Context of the COVID-19 Pandemic	2166	Clinical, not randomised
NCT04367636	The Effects of Attention Training on Emotion Regulation and Stress Related Complaints During COVID-19	2167	No medical treatment intervention
NCT04367662	Study of the Vascular Compartment and Hypercoagulability During Coronavirus Infection COVID-19	2168	Clinical, not randomised
NCT04367714	Antibody Response Against SARS-CoV-2 in Dialysis Patients During COVID-19	2169	Observational
NCT04367740	ScreenNC, a Study to Determine the Number of Asymptomatic Individuals Who Have Antibodies to the Virus That Causes COVID-19	2170	Observational
NCT04367792	Cardiac Injury in COVID-19: a Pathology Study	2171	Observational
NCT04367805	COVID-19 Infection in Patients With Hepatocellular Carcinoma	2172	Observational
NCT04367857	ARMOR Study: COVID-19 Seroprevalence Among Healthcare Workers	2173	Observational
NCT04367870	COVID-19 Detection Test in Oncology	2174	Observational
NCT04367896	COVID-19 OUTBREAK AND SOLID ORGAN TRANSPLANTATION (SOT) : AN INTERNATIONAL WEB-BASED SURVEY	2175	Observational
NCT04368013	Host-pathogen Interactions, Immune Response, and Clinical Prognosis at COVID-19 - the CoVUm Trial	2176	Clinical, not randomised
NCT04368065	Registry to Study Factors That May Impact COVID-19 Occurrence and Severity	2177	Observational
NCT04368143	COVID-19 Immune Repertoire Sequencing	2178	Observational
NCT04368208	Impact of Giving Birth During the Covid 19 Pandemia on Postnatal Women's Depression	2179	Observational
NCT04368221	Characterization of Fungal Infections in COVID-19 Infected and Mechanically Ventilated Patients in ICU	2180	Observational
NCT04368234	Duke COVID-19 Shared Data and Specimen Repository	2181	Observational
NCT04368260	Clinical Validation of New Injection Molded Flocked Nasopharyngeal Swabs in Response to the COVID-19 Pandemic	2182	Cinical, not randomised
NCT04368299	Telemedicine for Follow-up of Systemic Lupus Erythematosus	2183	No medical treatment intervention
NCT04368312	Psychological Impairment Due to Covid-19	2184	Observational
NCT04368338	Lung Ultrasound to Diagnose COVID-19	2185	Observational
NCT04368351	Bacteriotherapy in the Treatment of COVID-19	2186	Observational
NCT04368377	Enhanced Platelet Inhibition in Critically Ill Patients With COVID-19	2187	Clinical, not randomised
NCT04368390	Neuroradiolological Analysis of COVID-19 Patients	2188	Observational
NCT04368637	Acute Cardiovascular Events Triggered by COVID-19-Related Stress	2189	Observational
NCT04368676	Breath Regulation and Yogic Exercise An Online Therapy for Calm and Happiness	2190	No medical treatment intervention
NCT04368845	Telerehabilitation in Patients With COVID-19 After Hospitalization. The ATHLOS Study	2191	No medical treatment intervention
NCT04368871	Radiographic Findings and Their Temporal Changes in COVID-19 Positive Changes Patients: A Prospective Study	2192	Observational
NCT04368884	Observational Study to Assess the Seroconversion Status of Health Care Workers and Suspected or Confirmed Cases of COVID-19 Patients	2193	Observational
NCT04368897	In-vitro Diagnostic Test to Predict COVID-19 Mortality and Disease Severity	2194	Observational
NCT04368923	Management of Covid-19 Patients During Home Isolation	2195	No medical treatment intervention
NCT04368975	ARDS Caused by COVID-19	2196	Observational
NCT04369066	COVID-19 Study of the Serological Response Against the Severe Acute Respiratory Syndrome (SARS) Coronavirus 2 (CoV-2) Virus in 2 Types of Employees, Hospital and Non-hospital, at	2197	Clinical, not randomised
NCT04369274	Repeated Measures Trial of Temporary Automated Manual Ventilation Versus Noninvasive Oxygenation or Conventional Vent	2198	Clinical, not randomised
NCT04369300	Anxiety And Depression During COVID-19 IN INDIA	2199	Observational
NCT04369378	Effectiveness of Using a Meditation App in Reducing Anxiety and Improving Well-being During the Covid-19 Pandemic	2200	Clinical, not randomised
NCT04369456	Blood Biomarkers as Predictors of COVID-19 Disease Progression in Recently Infected Kidney Transplant Patients	2201	Clinical, not randomised
NCT04369599	Trans Thoracic Manipulation of Ventilation/Perfusion: the V/Q Vest	2202	Clinical, not randomised
NCT04369690	The Psychological, Social, and Economic Impacts of COVID-19	2203	Observational
NCT04369807	Mapping Organ Health Following COVID-19 Disease Due to SARS-CoV-2 Infection	2204	Observational
NCT04369820	C5a Receptor Expression - COVID-19 (C5-COV)	2205	Cinical, not randomised
NCT04369859	Obstetric and Perinatal Outcomes of Women With COVID-19	2206	Observational
NCT04369989	Observational Study of COVID-19 Treatment Efficacy	2207	Observational
NCT04370171	Management of Diabetic Patients With Telemedicine in the Context of the Covid-19 Epidemic	2208	Observational
NCT04370197	Covid-19 Epidemic on Acute Stroke Management	2209	Observational
NCT04370210	Sleep Quality During COVID-19 Containment in Children Whether or Not Usually Followed in Child Psychiatry	2210	Observational
NCT04370249	Construction of a Composite Clinical-echo Score Predictive of a Risk of Short-term Aggravation of Respiratory Impairment in Patients Suspected of Covid-19	2211	Observational
NCT04370275	Accuracy of Lung Ultrasound in the Diagnosis of covid19 Pneumonia	2212	Observational
NCT04370678	Changes in Preference for Surgery of Patients Signed up for Arthroscopic Procedures	2213	Observational
NCT04370808	BREATH: Vitamin D Polymorphisms and Severity of COVID-19 Infection	2214	Observational
NCT04370821	COVID-19 CHAMPS Study of Healthcare, First Responder and Service Workers	2215	Observational
NCT04370834	Tocilizumab for Patients With Cancer and COVID-19 Disease	2216	Clinical, not randomised
NCT04370886	Recruit Blood Donors Via SMS During Epidemic of COVID-19	2217	No medical treatment intervention
NCT04370938	Provider Burnout During COVID-19	2218	No medical treatment intervention
NCT04371029	Impact of Neck Inspiratory Muscle Activation During Sleep in ICU Patients After a COVID 19 ARDS	2219	Clinical, not randomised
NCT04371289	Predictors of Cardiovascular Risk in Covid-19 Patients During Acute Disease and at Short Term Follow-up	2220	Observational
NCT04371302	Nursing Perspective on Burnout and Medical Errors in the Intensive Care Unit During Covid-19 Pandemic	2221	Observational
NCT04371315	Risk Factors, Clinical Characteristics and Outcomes of Acute Infection With Coronavirus 2019 (COVID-19) In Children	2222	Observational
NCT04371354	Outcomes of Covid Protective Measures in Endoscopy	2223	Observational
NCT04371419	COVID-19 Health Messaging to Underserved Communities	2224	No medical treatment intervention
NCT04371432	Genetics of COVID-19 Susceptibility and Manifestations	2225	Observational
NCT04371471	Covid-19 Pandemic Triage Score	2226	Observational
NCT04371510	Blood Biomarkers as Predictors of COVID-19 Disease Progression in Recently Infected Chronic Haemodialysis Patients	2227	Clinical, not randomised
NCT04371562	PREDICTING ICU ADMISSION AND DEATH FOR COVID-19 PATIENTS	2228	Observational
NCT04371679	Changes in Cardiac and Pulmonary Hemodynamics as Predictor of Outcome in Hospitalized COVID-19 Patients	2229	Observational
NCT04371692	Prevalence of COVID-19 Infection Among HCW in a French University Hospital	2230	Observational
NCT04371744	QT-Logs : Artificial Intelligence for QT Interval Analysis of ECG From Smartwatches in Patient Receiving Treatment for Covid-19	2231	Observational
NCT04371822	Efficacy of Sn-protoporphyrin IX (SnPPIX) and Sulfonated Tetranaphthyl Porphyrin Against Covid-19	2232	Cinical, not randomised
NCT04371835	COHIVE: Coronavirus (COVID-19) Outcomes in HIV Evaluation in Resource Limited Settings	2233	Observational
NCT04371848	Impact of the COVID-19 Pandemic on Diet Quality and Food Insecurity: a NutriQu√©bec Sub-study	2234	Observational
NCT04372004	Comparison of the Efficacy of Rapid Tests to Identify COVID-19 Infection (CATCh COVID-19)	2235	Cinical, not randomised
NCT04372030	Health Care Use During the Covid19 Crisis	2236	Clinical, not randomised
NCT04372043	Evaluation of the Sleep Hygiene in the Lebanese Population During the COVID-19 Confinement Period.	2237	Observational
NCT04372056	The COVID-ICU Healthcare Professional Study	2238	Observational
NCT04372069	London Ambulance Service Experience of COVID-19	2239	Observational
NCT04372199	SEVERITY SCORE FOR COVID-19 PNEUMONIA	2240	Observational
NL8562	Bioelectric Impedance Analysis in COVID-19 positive patients. A prospective prevalence study	2241	Observational
NL8563	Patient‚Äôs knowledge And behavior oN the COVID-19 disease and as DEterMInants of Contamination	2242	Observational
NL8570	COVID-19 infections in health care workers	2243	Observational
NL8572	Prevalence of asymptomatic deep vein thrombosis in admitted COVID-19 patients	2244	Observational
RPCEC00000308	Pharmacodynamics of the Nasalferon in healthy volunteers.	2245	Not COVID-19 study
ACTRN12620000457943	Hydroxychloroquine for the Community-Based Treatment of COVID-19	2246	Duplicate
EUCTR2020-001571-32	Low dose of IL-2 In Acute respiratory DistrEss syndrome related to COVID-19	2247	Duplicate
EUCTR2020-001198-55	Early Treatment to Patients with Coronavirus (COVID)	2248	Duplicate
EUCTR2020-001686-36	A double-blind, randomized study versus placebo of avdoralimab (IPH5401), an anti-C5aR antibody, in patients with COVID-19 induced pneumonia	2249	Duplicate
EUCTR2020-001723-13	A randomized trial of efficacy and safety of an early OUTpatient treatment of COVID-19 in patients with risk factor for poor outcome : a strategy to prevent hospitalization : OUTCOV Study	2250	Duplicate
NCT04366908	Prevention and Treatment With Calcifediol of COVID-19 Induced Acute Respiratory Syndrome	2251	Duplicate
NCT04369742	Treating COVID-19 With Hydroxychloroquine (TEACH)	2267	Cancelled
NCT04371926	Prophylactic Benefit of Hydroxychloroquine in COVID-19 Cases With Mild to Moderate Symptoms and in Healthcare Workers With High Exposure Risk	2281	Cancelled
NL8576	Effects of COVID-19 on quality of life and mental health in asthma	2288	Observational
NL8580	Metabolic syndrome And Severity of Covid-19	2289	Observational
NCT04377100	Impact on Anxiety and Motivation of COVID-19 and Predictors of Individual Responses	2290	Clinical, not randomised
NCT04376593	18F-av√ü6-binding-peptide PET/CT in Patients Post SARS CoV2 Infection	2291	Clinical, not randomised
NCT04373161	Utilizing Home Pulse Oximetry for Patients With COVID-19 To Monitor Disease Progression	2292	Clinical, not randomised
NCT04373005	A Research Platform to Screen Patients in Oncology and Assess Impact - RESPONSE	2293	Clinical, not randomised
NCT04377737	Incidence of Covid-19 in School Children During the Pandemic Period in Nice	2294	Clinical, not randomised
NCT04379154	Analysis of Volatile Organic Compounds by Electronic Noses in Hospitalised Patients for an Infection by SARS-CoV-2: Predictive Interest in Short-term Evolution	2295	Clinical, not randomised
NCT02765191	MR-Evaluation of Renal Function In Septic Patients	2296	Clinical, not randomised
NCT04377802	Safe Return to Regular Clinical Operation After COVID-19 Pandemic; (Oncology Center, Prospective Cohort)	2297	Clinical, not randomised
NCT04374786	Effects of a Mobile App on Health and Well-being During COVID-19 Pandemic in House Staff at Banner University Medical Center Phoenix	2298	Clinical, not randomised
NCT04379518	Phase 1/2A Study of Rintatolimod and IFN-Alpha Regimen in Cancer Patients With Mild or Moderate COVID-19 Infection	2299	Clinical, not randomised
NCT04379453	Robot Assisted Percutaneous Cardiovascular Intervention as a Strategy to Reduce or Risk of Intra-Procedure Contamination by COVID-19 and Other Respiratory Viruses	2300	Clinical, not randomised
NCT04374591	Inhalable Sodium Bicarbonate: A Possible Adjuvant Treatment of Patients With Novel COVID-19: A Feasibility Study	2301	Clinical, not randomised
NCT04377672	Safety and Pharmacokinetics of Human Convalescent Plasma in High Risk Children Exposed or Infected With SARS-CoV-2	2302	Clinical, not randomised
NCT04372602	A Pilot Study of Duvelisib to Combat COVID-19	2303	Clinical, not randomised
NCT04373044	An Open-Label, Phase II of Antiviral Therapy Combined With Baricitinib in Moderate and Severe Patients With COVID-19	2304	Clinical, not randomised
NCT04374565	Efficacy and Safety of High-Titer Anti-SARS-CoV-2 (COVID19) Convalescent Plasma for Hospitalized Patients With Infection Due to COVID-19 to Decrease Complications: A Phase II Trial	2305	Clinical, not randomised
NCT04377477	Pilot Study on the Feasibility of Low Dose Radiotherapy for SARS-Cov-2 Pneumonitis (COVID-19 Low Dose Radiotherapy - COLOR 19)	2306	Clinical, not randomised
NCT04380532	Clinical Trial of COVID-19 Therapeutic Vaccine Formulated as an Oral Pill	2307	Clinical, not randomised
NCT04378244	CORONA: A Phase 1/2 Study Using DeltaRex-G Gene Therapy for Symptomatic COVID-19	2308	Clinical, not randomised
NCT04379037	Vagus Nerve Stimulation ARDS Prevention Trial for COVID-19 Hospitalized Patients	2309	Clinical, not randomised
DRKS00021676	Communication in times of the corona virus pandemic (COVID-19) - KomCo	2310	Observational
DRKS00021675	Sorting the wheat form the chaff ‚Äì COVID-19 in the Emergency department - Spread COVID 19 ED	2311	Observational

Registry entry ID	Title	Database ID (ID all)	Reason for exclusion
NCT04379531	Low-dose Computed Tomography in COVID-19 Pneumonia: a Prospective Moscow Study	2312	Clinical, not randomised
NCT04373824	To Study the Effectiveness of Ivermectin With Standard of Care Treatment Versus Standard of Care Treatment for COVID 19 Cases. A Pilot Study	2313	Clinical, not randomised
NCT04372693	Unplanned Shifting the Traditional Classroom-Based to Online Distance Learning as a Result of (COVID-19) Social Distancing Measures: Nursing Students' Perception and Achievement	2314	Clinical, not randomised
NCT04373200	Human Ab Response & immunoMONItoring of COVID-19 Patients	2315	Clinical, not randomised
NCT04376476	Host-pathogen Interactions During Paediatric and Adult SARS-CoV-2 Infection (COVID-19)	2316	Clinical, not randomised
NCT04377789	The Possible Effect of Quercetin on Prophylaxis and Treatment of COVID-19	2317	Clinical, not randomised
NCT04379661	SUNLIGHT Study: Online Support Groups for Multiple Sclerosis (MS) to Address COVID-19	2318	Clinical, not randomised
NCT04380818	Low Dose Anti-inflammatory Radiotherapy for the Treatment of Pneumonia by COVID-19: Multi-central Prospective Study	2319	Clinical, not randomised
NCT04376814	Evaluation of Safety and Efficacy of Hydroxychloroquine Plus Favipiravir Drug Regimen in Comparison With Hydroxychloroquine Plus Kaletra on the Need for Intensive Care Unit Treatment in	2320	Clinical, not randomised
NCT04381338	Pulmonary and Motor Rehabilitation for People With COVID-19 in Intensive Care Units to Reduce Length of Stay in Hospital	2321	Clinical, not randomised
NCT04375124	Evaluation of the Possible Role of Angiotensin Peptide (1-7) on Treatment of COVID-19	2322	Clinical, not randomised
NCT04374461	Phase II Study of N-acetylcysteine in Severe or Critically Ill Patients With Refractory COVID-19 Infection	2323	Clinical, not randomised
NCT04377308	Fluoxetine to Reduce Intubation and Death After COVID19 Infection	2324	Clinical, not randomised
NCT04378712	Effects of Hydrogen/Oxygen Mixed Gas Inhalation for Patients With Coronavirus Disease 2019 (COVID-19) Who Had Dyspnea: A Multicenter, Open-label Clinical Trial	2325	Clinical, not randomised
NCT04381000	The Impact of Quarantine and the Effectiveness of Telerehabilitation in Patients With Chronic Pain During COVID-19 Pandemic.	2326	Clinical, not randomised
NCT04381013	Emergency Ventilator Splitting Between Two or More Patients Using a Single Ventilator to Address Critical Ventilator Shortages During a Pandemic	2327	Clinical, not randomised
NCT04374149	Interventional Study to Evaluate the Efficacy of Therapeutic Plasma Exchange (TPE) Alone or in Combination With Ruxolitinib in COVID-19 Positive Patients With PENN Grade 2, 3, 4 Cytokine	2328	Clinical, not randomised
NCT04375774	Verification of Alternative Do-it-yourself Equipment Respirators	2329	Clinical, not randomised
NCT04378920	A Study of Trans Crocetin in Patients With Acute Respiratory Distress Syndrome Due to COVID-19 Disease	2330	Clinical, not randomised
NCT04380376	Single-center, Prospective, Open-label, Comparator Study, Blind for Central Accessor to Access the Efficacy, Safety, and Tolerability of Inhalations of Low-doses of Melphalan in Patients With	2331	Clinical, not randomised
DRKS00021658	Incidence and risk factors for acute kidney injury in COVID-19	2332	Observational
DRKS00021226	PROTEGO: PRospective Observational cohort Trial Evaluating correlation of clinical findings, symptoms and disease development of German in- or Outpatients infected with SARS-CoV-2 - a	2333	Observational
DRKS00021297	Clinical and immunological predictors of SARS-CoV-2 infection and Covid-19 diseases in patients with renal diseases and healthy persons	2334	Observational
DRKS00021671	Neuroendocrine mechanisms Social isolation and psychobiological stress during the Covid-19 pandemic - SoDi Study	2335	Observational
DRKS00021630	Detection of a delayed Treatment of urgent ophthalmologic Treatment during the SARS-CoV-2 pandemic (COVID-19) - COVID-19 DETOUR: Delayed Treatment of urgent ophthalmologic	2336	Observational
DRKS00021588	Naturalistic evaluation of an Internet-based psychological training program for mental strain associated with the coronavirus / COVID-19 pandemic - HelloBetter Corona	2337	Clinical, not randomised
DRKS00021432	Limitations of social life in the context of the COVID-19 pandemic - Effects on physical performance and health-related quality of life of older people - CoNFINE	2338	Observational
DRKS00021295	Clinical characteristics of renal patients with COVID-19 infection	2339	Observational
ISRCTN28342533	The PRIEST study: Pandemic Respiratory Infection Emergency System Triage	2340	Observational
ChiCTR2000032716	High dose intravenous vitamin C might be used as an important rescue therapy for aggravation of severe and critical novel coronavirus pneumonia (COVID-19) patients	2341	Clinical, not randomised
EUCTR2020-001768-27-FR	"STUDY OF THE EFFICIENCY OF NORMAL HUMAN IMMUNOGLOBULINS (IVIG) IN PATIENTS AGED 75 YEARS AND OVER COVID-19 WITH SEVERE ACUTE RESPIRATORY	2342	Clinical, not randomised
EUCTR2020-001777-71-GB	Ruxolitinib therapy to Avoid Ventilation and improve outcome for deteriorating COVID-19 patiENts - RAVEN - RAVEN	2343	Clinical, not randomised
EUCTR2020-001882-36-GR	ANTI-INFLAMMATORY CLARITHROMYCIN TO IMPROVE SARS-CoV-2 (COVID-19) INFECTION EARLY: THE ACHIEVE OPEN-LABEL NON-RANDOMIZED CLINICAL TRIAL	2344	Clinical, not randomised
ChiCTR2000032770	Asymptomatic novel coronavirus pneumonia (COVID-19) patients Have Longer Treatment Cycle Than Mild and Moderate Patients	2345	Observational
ISRCTN20867558	Survive and Thrive: exploring the wellbeing of doctors in a post-COVID-19 era: a Core Outcome Set to measure wellbeing	2346	Observational
ISRCTN87832712	Characterization of oral microbiome in SARS-Cov-2 positive patients and asymptomatic subjects: CovOM study	2347	Observational
ISRCTN97041334	Enforced social isolation and mental health: an observational study of the psychosocial effects of quarantine during COVID-19	2348	Observational
ACTRN12620000554965	ADAPT - COVID-19 Study - Characterising pathophysiological, immunological and clinical outcomes relating to COVID-19 infection in the patient population of St Vincent‚Äôs Hospital Sydney.	2349	Observational
ChiCTR2000032686	Study for the application of novel coronavirus pneumonia (COVID-19) intestinal tract toxicity in diagnosis and its prognostic effect	2350	Observational
ChiCTR2000032767	The Clinical Efficacy and Safety of "clear lung detoxification soup" in the Treatment of Novel Coronavirus Pneumonia (COVID-19) at Different Time Points: A Retrospective Study	2351	Observational
ChiCTR2000032666	Follow-up study of pregnancy outcomes of novel coronavirus pneumonia (COVID-19) complicated in the first and second trimester	2352	Observational
ChiCTR2000032768	Correlation between novel coronavirus pneumonia (COVID-19) and mental health of breast cancer patients: a cross-sectional study in Hunan Province	2353	Observational
ChiCTR2000032743	The effectiveness of diaphragmatic breathing relaxation training for improving sleep quality among nursing staff during the novel coronavirus pneumonia (COVID-19) pandemic: a before and	2354	Observational
NCT04372368	Convalescent Plasma for the Treatment of Patients With COVID-19	2355	Clinical, not randomised
NCT04374370	Severe Acute Respiratory Syndrome Coronavirus 2 of the Genus Betacoronavirus (SARSCoV2) Convalescent Plasma (CP) Expanded Access Protocol (EAP)	2356	Clinical, not randomised
NCT04378582	Characteristics and Outcomes of Patients With COVID-19 Admitted to the ICU	2357	Observational
NCT04373109	Stroke Rehabilitation Outcome During COVID-19 Lockdown	2358	Observational
NCT04374422	Effect of SARS- COVID-19 Pandemic on Female Sexual Behavior	2359	Observational
NCT04374617	Risk of Venous Thromboembolism in Critically Ill Patients With Severe COVID-19	2360	Observational
NCT04374838	Effect of COVID-19 Pandemic on Pediatric Cancer Care	2361	Observational
NCT04375501	A Multicentre Study on the Effect of COVID-19 on 30-day Morbidity and Mortality Rates in Patients With Neck of Femur Fractures	2362	Observational
NCT04375709	Health-related Quality of Life (HRQOL) and Physical Performance in Individuals After COVID-19 Induced Hospitalisation and the Impact of a Standard Care Follow-up Program: a Longitudinal	2363	Observational
NCT04376580	Incidence and Implications With the Use of Nasal Cannulas During COVID-19	2364	Observational
NCT04376775	Assessment of the Impact of COVID-19 on Transplant Patients and on Patients Awaiting Transplantation	2365	Observational
NCT04376996	Slovenian National COVID-19 Prevalence Study	2366	Observational
NCT04377425	COVID-19 Prevalence, Morbidity and Long Term Cognitive Deficits in Consecutive Patients Presenting With Acute Neurological Symptoms	2367	Observational
NCT04377464	COVID-19 - Quality of Life After Infection	2368	Observational
NCT04380714	Changes in the Consumption of Psychoactive Substances by People Incarcerated at the Villeneuve-L√®s-Maguelone Prison During Containment Linked to COVID-19.	2369	Observational
NCT04380896	COVID-19 Staff Testing of Antibody Responses Study (CO-STARS)	2370	Observational
NCT04381351	The Role of Noncoding RNAs in COVID-19 and COVID-19 Associated Acute Kidney Injury (MiRCOVID)	2371	Observational
NCT04307459	Acute Respiratory Failure and Continuous Positive Airway Pressure Therapy in Patients With Severe Acute Respiratory Syndrome Coronavirus 2 (SARS-CoV-2) Infection: a Real Life Evaluation	2372	Observational
NCT04372472	SQuISH-COVID: A Pilot Study	2373	Observational
NCT04371250	Mental Health Implications During and After the COVID-19 Pandemic Lock-down Measures in Youth (18 - 25 Years Old) in French-Speaking Belgium and Italy: Resilience and Loneliness	2374	Observational
NCT04373148	Understanding Immunity to SARS-CoV-2, the Coronavirus Causing COVID-19	2375	Observational
NCT04373798	Triage Strategies Based on C-reactive Protein Levels Among Individuals With COVID-19: A Prospective Cohort Study	2376	Observational
NCT04372576	Epidemiology and Outcome of Ventilator-associated Pneumonia Among Critically Ill COVID-19 Patients	2377	Observational
NCT04373889	SARS-COV-2 Prevalence, Seroprevalence and Seroconversion Among Healthcare Workers in Belgium During the 2020 COVID-19 Outbreak	2378	Observational
NCT04374123	COVID-19 Serologic Screening and a Strict Protocol in the Management of a Referral Gastrointestinal Endoscopy Unit: a Prospective Study	2379	Observational
NCT04373213	Evaluation of the Fluid Response of SARS-CoV-2 (COVID-19) Patients in Intensive Care; Pleth Variability Index	2380	Observational
NCT04373486	Incidence of Acute Pulmonary Embolism in Covid-19 Patients on CT Angiography and Relationship to D-dimer Levels	2381	Observational
NCT04374058	Reduced Frequency Hemodialysis in Prevalent Patients Due to the Coronavirus Disease	2382	Observational
NCT04374656	Prevalence of SARS-CoV-2 in Conjunctival Swab Samples Among Patients Presenting With Conjunctivitis to the Ophthalmology Clinics During the COVID-19 Pandemic	2383	Observational
NCT04373811	Quality of Life and Functional Prognosis at One Year of Patients With COVID-19 Admitted in Post-ICU Setting	2384	Observational
NCT04374071	Early Short Course Corticosteroids in Hospitalized Patients With COVID-19	2385	Observational
NCT04375748	Hospital Registry of Acute Myocarditis: Evolution of the Proportion of Positive SARS-COV-2 Cases During the Covid-19 Pandemic, Case Characteristics and Prognoses	2386	Observational
NCT04374097	Level and Predictors of Trauma-symptoms Among Health Workers and Public Service Providers During the COVID-19 Outbreak	2387	Observational
NCT04374513	SURVEY of RETAIL PHARMACISTS KNOWLEDGE, ROLE & BEHAVIOR DURING COVID-19 VIRUS OUTBREAK	2388	Observational
NCT04374643	Descriptive Study of the PSYchological Impact of CONFinement Measures in Subjects With Mood Disorders : PsyConf	2389	Observational
NCT04374695	Association Between Hypertension, Renin-Angiotensin-Aldosterone System Inhibitors and COVID-19	2390	Observational
NCT04377074	Parenting in a Pandemic: Parental Stress During the COVID-19 and Its Association With Depression and Anxiety	2391	Observational
NCT04377490	Thrombo Embolic Events in Hospitalized Patients With Covid-19 Serious Acute Pneumopathy	2392	Observational
NCT04377581	COVID-19 Health Messaging Efficacy and Its Impact on Public Perception, Anxiety, and Behavior	2393	Observational
NCT04377607	An Important Index on Severity of Coronavirus Disease: Erythrocyte Haemoglobin Concentration	2394	Observational
NCT04375137	Correlation Between Oxidative Stress Markers and COVID-19 Severity Index	2395	Observational
NCT04375176	Study of Immune-mediated Mechanisms in Patients Tested Positive for SARS-CoV-2: Phenotypic and Functional Analysis of Monocytes and NK Cells in the Blood of Subjects Affected by Covid 19	2396	Observational
NCT04375644	Impact of the COVID-19 Pandemic on the Quality of Psychological Life of Healthcare Professionals	2397	Observational
NCT04377724	Longitudinal Serological Study on the Spread and Course of COVID-19 Infections	2398	Observational
NCT04377815	FORECAST Study: Finding Out if COVID-19 Infection Can be pREdicted by ChAnges in Smell and/or Taste	2399	Observational
NCT04375761	Human Epidemiology and Response to SARS-CoV-2 (DAIT-COVID-19-001)	2400	Observational
NCT04378881	Simulation of Risk of Adverse Drug Events Associated With the Initiation of Drugs Repurposed for the Treatment of COVID-19 in Adults With Polypharmacy Using Data From Large Medicare	2401	Observational
NCT04379232	Surgical Activity During the Covid-19 Pandemic: Results for 112 Patients in a French Tertiary Care Center: A Retrospective Observational Cohort Study	2402	Observational
NCT04376658	Assessment of Health-related Quality of Life and Patient-centered Outcomes After Hospitalization for COVID-19: A Multicenter Prospective Cohort Study	2403	Observational
NCT04376710	Surgical Telemedicine in the COVID-19 Pandemic Era	2404	Observational
NCT04376853	CoronaWatch - Early Detection of Cardiovascular Risks in COVID-19 Via SmartWatch	2405	Observational
NCT04376944	Evaluating Effectiveness of Barrier Measures to Limit Nosocomial Transmission: Covid-19 Prevalence Survey Among Caregivers With Various Exposures	2406	Observational
NCT04376879	Predicting the Need for Intubation in Hospitalised COVID-19 Patients (PRED ICU COVID19)	2407	Observational
NCT04376905	Assessment of Extra Vascular Lung Water and Pulmonary Permeability by Transpulmonary Thermodilution in Critically Ill Patients With Severe Acute Respiratory Syndrome Coronavirus 2	2408	Observational
NCT04377035	The ECHOVID-19 Study - A Prospective Cohort Study Investigating the Acute Effect of COVID-19 on the Heart and Lung by Ultrasound	2409	Observational
NCT04377412	Risk Factors for Anxiety and Depression Among Pregnant Women During the COVID-19 Pandemic - a Web-based Cross-sectional Survey	2410	Observational
NCT04379466	Effector and Regulatory T Cell Receptor Repertoire Analyses in Patients Affected by COVID-19	2411	Observational
NCT04377685	Prediction of Clinical Course in COVID19 Patients Using Unsupervised Classification Approaches of Clinical, Biological and the Multiparametric Signature of the Chest CT Scan Performed at	2412	Observational
NCT04381273	Oral Health and Psychosocial Factors During the Covid-19 Pandemic	2413	Observational
NCT04377763	Developement and Evaluation of Serological Assays for COVID-19	2414	Observational
NCT04378595	Assessing Pediatric Food Insecurity During the COVID-19 Pandemic in Austin Federally Qualified Health Centers	2415	Observational
NCT04377776	Observational Study to Assess the Health Status of Transplanted Patients (Kidney or Pancreas or Pancreatic Islet Transplantation) at Transplant Medicine Unit (MITRA) During the COVID-19	2416	Observational
NCT04378777	A Prospective Cohort Study to Assess Longitudinal Immune Responses in Hospitalized Patients With COVID-19 (DAIT-COVID-19-002)	2417	Observational
NCT04378829	Does the Platelet Count, Mean Platelet Volume and Platelet Distribution Width Change in COVID-19 Pandemic, ?	2418	Observational
NCT04378842	Impact of SARS-CoV-2 Infection on the Incidence of ICU Acquired Colonization Related to Multidrug-resistant Bacteria	2419	Observational
NCT04379063	COVID Pandemic Short Interval National Survey Gauging Psychological Distress Among Physicians (COPING Survey): A Longitudinal Survey	2420	Observational
NCT04379245	Study of the Attack Rate of COVID-19 Infection in Patients Infected With HIV and/or on Pre-exposure Prophylaxis (PrEP) and Possible Impact of Treatment With an HIV Protease Inhibitor	2421	Observational
NCT04379089	Neurologic Manifestations of COVID 19 in Children	2422	Observational
NCT04379297	COvid-19 National Survey for Assessing VIral Spread by Nonaffected CarriErs (CON-VINCE)	2423	Observational
NCT04379310	Evaluation of Demographic and Clinical Parameters on Admission and Medications Used for Comorbidities in Patients With Covid-19 Pneumonia: A Single Center Experience in Turkey	2424	Observational
NCT04380779	Prevalence of Severe Bleeding in COVID-19 Patients Treated With Higher Than Recommended Thromboprophylaxis Doses (BLEEDING Study)	2425	Observational
NCT04380870	Describing Chinese Herbal Medicine Telehealth Care for Symptoms Related to Infectious Diseases Such as COVID-19: A Descriptive, Longitudinal, Pragmatic Cohort Study	2426	Observational
NCT04380792	Prevalence of DVT or PE in Patients Infected With COVID-19 Admitted to Hospital (SCREENING Study)	2427	Observational
NCT04380987	Luxembourg Cohort of Positive Patients for COVID-19: a Stratification Study to Predict Patient Prognosis	2428	Observational
NCT04381286	Transpulmonary Driving Pressure in ARDS COVID19 Patients / TRANSPULMONARY-COVID19	2429	Observational
NCT04381312	COVID-19 Chez la Personne √¢g√©e de Plus de 70 Ans : Impact Direct et Indirect √† 3 Mois.	2430	Observational
NCT04373759	Cardiac Arrest Incidence and Outcome Among Patient With COVID-19 Pneumonia in French ICUs	2431	Observational
NCT04374045	Testing for Dysautonomia in Patients Hospitalized for SARS-CoV-2 Infection (COVID-19) : COVIDANS Study	2432	Observational
NCT04374110	Prospective Monitoring of Drug Safety and the Occurrence of Complications During Hospitalization in Patients With Cardiovascular Diseases With COVID-19	2433	Observational
NCT04376398	Survey on Patients With Suspected or Confirmed COVID-19 Requiring Anesthesia Care for Their Intervention	2434	Observational
NCT04379258	Epidemiological Analysis of the Mortality of Critically Ill Patients With the COVID-19 Admitted to the Intensive Care Unit: An Observational, Prospective and Multicenter Study	2435	Observational
NCT04379440	Geriatric Population COVID-19 Syndromic Characteristics and Clinical Outcomes: a Multi-setting, Multi-domain Observational Study (GEROCOVID Observational)	2436	Observational
NCT04380766	Effects of Covid-19 Pandemic on Pancreatic Surgery in Italy. A Multicenter Observational Clinical Study	2437	Observational
NCT04378452	Assessment of the Psycho-social Impact of COVID-19 Outbreak (COM-COVID)	2438	Observational
NCT04379544	Prognostic Value of Point of Care Cardiac and Lung Ultrasound in COVID-19	2439	Observational
NCT04380727	Almitrine and Severe COVID-19 Patients in ICU [Almitrine et Patients COVID-19 en Reanimation (French)]	2440	Observational
NL8577	Randomized controlled trial of Digital Cardiac Counseling in patients with delayed cardiac surgical treatment due to Covid-19 pandemic (DCC trial)	2441	No medical treatment intervention
NCT04373382	Peer Champion Support for Hospital Staff During and After the COVID-19 Pandemic	2442	No medical treatment intervention
NCT04378803	Mindfulness Training for Older Adults During the COVID-19 Pandemic	2443	No medical treatment intervention
NCT04373135	A Study to Evaluate Health Behavior and Access Impacts Due to COVID-19 and for Community Engagement of Stakeholders Surrounding Scarce Resource Allocation Policy.	2444	No medical treatment intervention
NCT04379375	Using Behavioral Nudges to Improve Preventive Health Behaviors That Limit COVID-19 Spread	2445	No medical treatment intervention
NCT04376515	HOPE Intervention for COVID-19	2446	No medical treatment intervention
NCT04378257	Efficacy of Therapist Guided e-Therapy Versus Self-Help Therapy on Psychological Distress Among Individuals in Oman During COVID-19 Pandemic: An Open-Label 12 - Weeks Randomized	2447	No medical treatment intervention
ISRCTN44152751	The School Opening in the Age of Pandemic (SOAP) study: a cluster-randomised re-introduction of school activities in Norway	2448	No medical treatment intervention
NL8578	Alkaline phosphatase for reducing systemic inflammatory response syndrome (SIRS) in patients with Sars-CoV-2 infection and acute respiratory insufficiency (COVID 19)	2449	Clinical, not randomised
ACTRN12620000555954	Randomised controlled trial of an app-based intervention, Anchored, to support the mental health of Australians recently unemployed due to COVID-19.	2450	No medical treatment intervention
EUCTR2020-001395-15-DK	Low dose hydrocortisone in patients with COVID-19 and severe hypoxia ,Äì the COVID STEROID trial	2451	Duplicate
NCT04374474	Olfactory Retraining Therapy and Budesonide Nasal Rinse for Anosmia Treatment in Patients Post-CoVID 19. A Randomized Controlled Trial	2452	No medical treatment intervention
NCT04373096	Development of a Novel Hood Shield to Enhance PPE Security and Minimize COVID-19 Transmission to Front-line Health Care Workers Performing High-risk Procedures	2453	No medical treatment intervention
ChiCTR2000032635	A randomized, double-blind, placebo, parallel controlled trial for Pien-Tze-Huang Capsules in treating convalescent novel coronavirus pneumonia (COVID-19) patient with SARS-CoV-2 positive.	2454	No medical treatment intervention
NCT04374084	Moxibustion Plus Cupping in Convalescent Patients With COVID-19: A Randomized Clinical Trial	2455	No medical treatment intervention
NCT04380909	Randomized Controlled Trial (RCT) to Evaluate the Efficacy of an Internet-based Self-help Program for People With Psychological Distress Due to Covid-19	2456	No medical treatment intervention
EUCTR2020-001909-22-FR	EFFICACY OF INTRAVENOUS ALMITRINE IN REDUCING THE NEED FOR MECHANICAL VENTILATION IN PATIENTS WITH HYPOXEMIC ACUTE RESPIRATORY FAILURE	2457	Duplicate
NCT04373291	Using BCG Vaccine to Enhance Non-specific Protection of Health Care Workers During the COVID-19 Pandemic. A Randomized Controlled Multi-center Trial	2458	Duplicate
NCT04374942	Protecting Frontline Health Care Workers From COVID-19 With Hydroxychloroquine Pre-exposure Prophylaxis: A Randomized, Placebo-controlled Multi-Site Trial in Toronto, Canada	2459	Duplicate
EUCTR2020-001700-42-FR	Efficacy of captopril nebulization in Covid-19 patients suffering of SARS-CoV-2 pneumonia.	2465	Duplicate
EUCTR2020-001684-89-GB	CATALYST - A randomised phase II proof of principle multi-arm multi-stage trial designed to guide the selection of interventions for phase III trials in hospitalised patients with COVID-19	2474	Duplicate
EUCTR2020-001825-29-ES	CLINICAL TRIAL OF THE USE OF ANAKINRA (ANTI IL-1) IN CYTOKINE STORM SYNDROME (CSS) SECONDARY TO COVID-19 - ANA-COVID-GEAS	2476	Duplicate
EUCTR2020-001474-29-ES	Pilot study to evaluate the potential of ivermectin to reduce COVID-19 transmission	2485	Duplicate
NCT04381962	A Multi-centre Open-label Two-arm Randomised Superiority Clinical Trial of Azithromycin Versus Usual Care In Ambulatory COVID-19 (ATOMIC2)	2538	Duplicate
NCT04381936	Randomized Evaluation of COVID-19 Therapy	2539	Duplicate

Registry entry ID	Title	Database ID (ID all)	Reason for exclusion
NCT04373733	A Randomised Controlled Trial of Early Intervention in COVID-19: Favipiravir Verses HydroxycholorquiNe & Azithromycin & Zinc vErsEs Standard CaRe	2540	Duplicate
NCT04373707	Weight-Adjusted vs Fixed Low Doses of Low Molecular Weight Heparin For Venous Thromboembolism Prevention in COVID-19	2541	Duplicate
NCT04382547	Treatment of Covid-19 Associated Pneumonia With Allogenic Pooled Olfactory Mucosa-derived Mesenchymal Stem Cells	2542	Cinical, not randomised
NCT04376034	Convalescent Plasma Collection and Treatment in Pediatrics and Adults	2543	Cinical, not randomised
NCT04380701	A Trial Investigating the Safety and Effects of Four BNT162 Vaccines Against COVID-2019 in Healthy Adults	2544	Cinical, not randomised
NCT04379986	Remote Blood Pressure Monitoring With the Wearable SENBIOSYS Photoplethysmographic Device	2545	Cinical, not randomised
NCT04382560	Coping Strategies and Responsiveness to a Brief Online Intervention During COVID-19 Pandemic	2546	No medical treatment intervention
NCT04382729	Neuromuscular Electrical Stimulation in Patients With COVID19 Associated Pneumonia	2547	No medical treatment intervention
NCT04377165	Innovative Tool to Limit Spread of SARS-CoV-2 in Residential Aged Care Facilities	2548	No medical treatment intervention
NCT04382794	Sitagliptin Treatment in Diabetic COVID-19 Positive Patients	2549	Observational
NCT04382469	Egyptian Initial Experience About 2019 Novel Corona Virus: Study of 48 Patients in Alexandria-Egypt Using Chest CT	2550	Observational
NCT04379284	Risks of COVID19 in the Pregnant Population	2551	Observational
NCT04375410	Consequences of Intensified Handwash and Hand Disinfection Among Children During the COVID19 Pandemic	2552	Observational
NCT04375670	COVID19-FOIE National Observatory	2553	Observational
NCT04370119	Screening for SARS-CoV-2-Infections and Monitoring of Serological Responses to SARS-CoV-2 in Healthcare Workers	2554	Observational
NCT04371328	Emergency Management in a Dedicated Respiratory Unit of Patients With a Possible COVID Infection (Unit "COVID Possible"¬∞	2555	Observational
NCT04382235	Non-invasive Ventilatory Support of Patients Affected by COVID-19	2556	Observational
NCT04382417	Observational Cohort Study of Critically Ill Patients With Covid-19 in Sweden	2557	Observational
NCT04382534	A Cross-sectional and Cohort Study of COVID-19 Patients With Post - Discharge Dysfunction	2558	Observational
NCT04382196	Impact of COVID-19 on Mental Health of Health Care Workers	2559	Observational
NCT04381819	Norwegian SARS-CoV-2 Study - Virological, Clinical and Immunological Characterisation of COVID-19	2560	Observational
NCT04378686	Serological Testing for COVID-19 (SARS-CoV-2) in ESKD	2561	Observational
NCT04380662	Risk Factors Worsening COVID19 for Out-patient With Home Monitoring	2562	Observational
NCT04382781	Immunosupressive Treatment in COVID-19 Patients	2563	Observational
NCT04370067	Hungarian COVID-19 Epidemiological Research	2564	Observational
NCT04368026	SARS-CoV-2 and Surgery	2565	Observational
NCT04382495	The Impact of COVID-19 Pandemic on Cancer Care	2566	Observational
NCT04367883	Influenza Vaccination, ACEI and ARB in the Evolution of SARS-Covid19 Infection	2567	Observational
NCT04368832	imPROving prenaTal carE During ConfinemenT	2568	Observational
NCT04382092	Co-infection Management in COVID-19 Critically Ill	2569	Clinical, not randomised
NCT04376840	Prevalence of Long-term Respiratory Complications of Severe SARS-CoV-2 Pneumonia	2570	Clinical, not randomised
NCT04378738	Social Media and Covid19 Pandemic	2571	Clinical, not randomised
NCT04382768	Inhaled Ibuprofen to Treat COVID-19	2572	Clinical, not randomised
ACTRN12620000547943		2573	Not COVID-19 study
ACTRN12620000551998		2574	Not COVID-19 study
NL8584		2575	No medical treatment intervention
NL8587		2576	No medical treatment intervention
NL8589		2577	No medical treatment intervention
NCT04382846	Novel Regimens in COVID-19 Treatment	2589	Duplicate
NCT04382950	Combination of Recombinant Bacterial ACE2 Receptors -Like Enzyme of B38-CAP and Isotretinoin Could be Promising COVID-19 Infection- and Lung Injury Preventing Drug Better Than	2592	Duplicate
DRKS00021677	COVID-19 associated neuropathy and myopathy in Germany.	2599	No medical treatment intervention
DRKS00021699	Influence of the COVID-19-pandemic on the treatment of ENT patients	2600	No medical treatment intervention
DRKS00021626	A Randomized, Controlled, Open Label, Multicentre Clinical Trial to explore Safety and Efficacy of Hyperbaric Oxygen for preventing	2601	Duplicate
DRKS00021638	Retrospective analysis of the relationship between invasive ventilation (considering COVID-19) and a Comprehensive Geriatric Assessment (CGA)	2602	Observational
IRCT20200413047062N1	Evaluating the efficacy of Atorvastatin in clinical improvement, prognosis and hospitalization course in patients with COVID-19 infection	2609	Cinical, not randomised
IRCT20130616013690N7	Comparison of interactive psycho-educational interventions via social networks with placebo on anxiety and self-efficacy of patients infected with COVID-19 and lived in home quarantine	2612	No medical treatment intervention
IRCT20200418047121N2	Investigation the adipose and placenta-derived mesenchymal stem cells effect on the respiratory distress syndrome in patients with COVID-19: a pilot study	2626	Cinical, not randomised
IRCT20200413047052N1	The effectiveness of educational-treatment package based on psychological flexibility in burnout, quality of working life, work-family conflict and mental health of the care team working in	2628	No medical treatment intervention
ChiCTR2000032842	Rapid Detection 2019-nCOV via Surface Plasamon Resonance	2632	No medical treatment intervention
RBR-4vm3yy	Effect of convalescent plasma in patients with severe COVI-19	2633	Cinical, not randomised
NL8609	Enhancing the BCG-induced trained immunity response by addition of bisphosphonate or MMR vaccine: a possible preventive approach against COVID-19 (BCG-PLUS)	2634	Cinical, not randomised
RPCEC00000311	Treatment of SARS CoV-2 pneumonia with the anti-CD6 monoclonal antibody itolizumab	2635	Cinical, not randomised
RPCEC00000313	CIGB-258 at COVID-19	2636	Cinical, not randomised
NCT04385017	Role of Inflammasomes in COVID-19 Disease	2637	Cinical, not randomised
NCT04387955	Analysis of Chilblains pROfile During coVID-19 Epidemic (ACROVID)	2638	Cinical, not randomised
NCT04383587	Seroprevalence of SARS CoV 2 Antibodies in Previously Undiagnosed Healthcare Workers	2639	Cinical, not randomised
NCT04389294	HSS Covid-19 Antibody Serology Among Surgeons & Anesthesiologists	2640	Cinical, not randomised
NCT04387292	Ocular Sequelae of Patients Hospitalized for Respiratory Failure During the COVID-19 Epidemic	2641	Cinical, not randomised
NCT04389385	COVID-19 Specific T Cell Derived Exosomes (CSTC-Exo)	2642	Cinical, not randomised
NCT04384497	Convalescent Plasma for Treatment of COVID-19: An Exploratory Dose Identifying Study	2643	Cinical, not randomised
NCT04384965	Accelerated iTBS for Depressed Patients During the COVID-19 Pandemic	2644	Cinical, not randomised
NCT04386239	Study on the Use of Sarilumab in Patients With COVID-19 Infection	2645	Cinical, not randomised
NCT04388527	COVID-19 Convalescent Plasma for Mechanically Ventilated Population	2646	Cinical, not randomised
NCT04389372	Effectiveness and Acceptability of MINDFULNESS by Smartphone, for Patients With Chronic Migraine and Medication Overuse	2647	Cinical, not randomised
NCT04389671	The Safety and Preliminary Efficacy of Lucinactant in Adults With COVID-19	2648	Cinical, not randomised
NCT04389710	Convalescent Plasma for the Treatment of COVID-19	2649	Cinical, not randomised
NCT04389944	Amotosalen-Ultraviolet A Pathogen-Inactivated Convalescent Plasma in Addition to Best Supportive Care and Antiviral Therapy on Clinical Deterioration in Adults Presenting With Moderate to	2650	Cinical, not randomised
NCT04390178	Convalescent Plasma as Treatment for Acute Coronavirus Disease (COVID-19)	2651	Cinical, not randomised
NCT04385108	Predictive Immune Biomarkers for COVID-19 Pathogenesis	2652	Cinical, not randomised
NCT04384458	COVID-19 Prophylaxis With Hydroxychloroquine Associated With Zinc For High-Risk Healthcare Workers	2653	Cinical, not randomised
NCT04385901	Long Term Functional Outcomes of COVID-19 Patients Treated by Rehabilitation Services viaTelehealth	2654	Cinical, not randomised
NCT04384588	COVID19-Convalescent Plasma for Treating Patients With Active Symptomatic COVID 19 Infection (FALP-COVID)	2655	Cinical, not randomised
DRKS00021706	Evaluation of online support during the coronavirus (COVID-19) pandemic: Online cognitive behavioral therapy for obsessive-compulsive disorder (OCD NET program)	2656	Cinical, not randomised
PACTR202004893013257	Preventing Pulmonary Complications in Surgical Patients at Risk of COVID-19	2657	Cinical, not randomised
CTRI/2020/05/025167	Evaluation of Efficacy and Safety of Thymoquinone compared to Best supportive care in Patients with COVID-19	2658	Cinical, not randomised
CTRI/2020/05/025013	Evaluation of BCG as potential therapy for COVID-19	2659	Cinical, not randomised
JPRN-JapicCTI-205270	A Study to Evaluate the Safety and Efficacy of Tocilizumab in Patients With Severe COVID-19 Pneumonia in Japan	2660	Cinical, not randomised
ACTRN12620000571976	Evaluation of Shift, a smartphone application for Junior Medical Officers in New South Wales during the COVID-19 epidemic.	2661	Cinical, not randomised
IRCT20200509047366N1	Radiotherapy in COVID-19	2662	Cinical, not randomised
JPRN-UMIN000040341	Immunological efficacy of Qing Fei Pai Du Tang for COVID-19	2663	Cinical, not randomised
JPRN-UMIN000040401	A feasibility study of remote rehabilitation using a wearable biological monitoring device in the patients with COVID-19	2664	Cinical, not randomised
JPRN-UMIN000040407	Immunological efficacy of Jinhua Qinggan Granule for COVID-19	2665	Cinical, not randomised
CTRI/2020/04/024747	EFFECTIVENESS TO MANAGE COVID 19 PANDEMIC BY TEACHING VENTILATORY MANAGEMENT TO NON-ANAESTHESIOLOGY RESIDENTS	2666	Cinical, not randomised
CTRI/2020/05/024967	Clinical trial on Immunity and antiviral for quarantine patients of COVID-19	2667	Cinical, not randomised
CTRI/2020/05/024983	Usage of topical lignocaine to decrease the gag reflex	2668	Cinical, not randomised
CTRI/2020/05/024986	HOMOEOPATHY IN PREVENTION OF COVID-19	2669	Cinical, not randomised
CTRI/2020/04/024731	Study to Evaluate Preventive Effect of Ayurveda and Homeopathy Treatment in COVID 19	2670	Cinical, not randomised
CTRI/2020/05/025069	Ayurvedic Interventions in prevention of COVID-19 infection-A survey study	2671	Cinical, not randomised
ISRCTN90906759	Investigating a vaccine against COVID-19	2672	Cinical, not randomised
ISRCTN85216856	Using blood plasma to develop passive immunity to coronavirus in Ecuador	2673	Cinical, not randomised
NL8627	Association of BMI with COVID-19 incidence and severity	2674	Observational
NL8613	Serial measurements in COVID-19-induced acute respiratory disease to unravel heterogeneity of the disease course: design of the Maastricht Intensive Care COVID cohort; MaastrICCht.	2675	Observational
NL8625	Hocus POCUS: a tool for increasing the efficiency of diagnosing venous thromboembolic disease in critically ill COVID-19 patients?	2676	Observational
RPCEC00000312	PrevengHo-Vir Homeoprohylaxis COVID-2019	2677	Observational
RPCEC00000310	Biomodulina T - IM- covid-19 - older adultos - Phase IV clinical trial	2678	Observational
DRKS00021611	Hypernatremia in critically ill patients with Covid-19	2679	Observational
DRKS00021740	CoViD-19 imaging register study Aachen	2680	Observational
DRKS00021664	Influence of COVID-19 Infection on sensory organs in othorynolaryngology	2681	Observational
DRKS00021688	Analysis of the Pathophysiology and Pathology of Corona Virus Disease 2019 (Pa-COVID-19)	2682	Observational
DRKS00021680	Quality assurance of cochlear implant rehabilitation in times of the COVID-19 pandemic (Corona)	2683	Observational
DRKS00021727	Incidence and long-term course of subclinical and clinical myocardial damage as well as pulmonary vascular and structural changes in patients with COVID-19	2684	Observational
JPRN-UMIN000040250	Changes of D-dimer in preventive period for COVID-19 for patients with chronic heart failure	2685	Observational
JPRN-UMIN000040278	Had COVID-19 Spread in Nagasaki Prefecture before the first confirmed case?	2686	Observational
JPRN-UMIN000040289	Simulation study of tracheal intubation through an aerosol-box assuming COVID-19	2687	Observational
JPRN-UMIN000040295	Prospective observational study on diagnosis and treatment of COVID-19	2688	Observational
JPRN-UMIN000040333	Fact-finding survey on subclinical infection of SARS-CoV-2 in Kanagawa Prefecture	2689	Observational
JPRN-UMIN000040335	Impacts of COVID-19 prevention life in community-dwelling older adults and support measures	2690	Observational
JPRN-UMIN000040353	Examination in COVID-19 pandemic; Whether endoscopies really generate aerosol particles?	2691	Observational
CTRI/2020/04/024772	Comparison of suspected or negative cases with confirmed cases of COVID-19 at Max Hospital	2692	Observational
CTRI/2020/04/024859	To Study the Clinical characteristics and treatment Outcome of COVID-19 Patients admitted in hospitals of Max Healthcare (SCOPe Study)	2693	Observational
CTRI/2020/05/025070	An Observational study to identify the issues and challenges in cancer patients on active treatment during the COVID-19 Pandemic and the resulting Lockdown	2694	Observational
CTRI/2020/05/025089	Effect of Hydroxychloroquine on QTc Interval	2695	Observational
CTRI/2020/04/024697	COVID-19 Data-bank Project	2696	Observational
CTRI/2020/04/024784	ANXIETY AND DEPRESSION DURING COVID-19 IN INDIA	2697	Observational
CTRI/2020/04/024805	Impact of Covid-19 pandemic on practice pattern of Indian urologists	2698	Observational
CTRI/2020/05/024984	How has the spread of COVID 19 disease affected the teaching and training of postgraduate students who are studying anaesthesiology	2699	Observational
CTRI/2020/05/024989	Mechanism of Covid 19 infection in Humans	2700	Observational
CTRI/2020/05/025010	Hydroxychloroquine prophylaxis in Covid 19 infection	2701	Observational
CTRI/2020/05/025041	Radiographic findings and their temporal changes in COVID-19 positive patient: A Prospective study	2702	Observational
CTRI/2020/05/025091	Knowledge status of public about COVID 19 disease prevention and control in Tamil Nadu	2703	Observational
CTRI/2020/05/025092	Outcomes of viral infection in hematology patients	2704	Observational
ACTRN12620000575932	Prevalence of the loss of smell in patients with COVID-19: The SMELLY Study	2705	Observational
JPRN-jRCT1032200025	new SARS-CoV-2 detection system	2706	Observational
CTRI/2020/04/024914	Characteristics of seriously ill COVID-19 patients admitted to a tertiary care centre	2707	Observational
CTRI/2020/05/024982	Effects of using hydroxychloroquine and azithromycin in the treatment of confirmed COVID-19 positive patients	2708	Observational
NCT04383444	Surveillance of Individuals Following SARS-CoV-2 Exposure	2709	Observational
NCT04383470	Collaborative Outcomes Study on Health and Functioning During Infection Times During COVID-19 Pandemic	2710	Observational
NCT04383626	Fear Among Dentists During COVID-19 Pandemic	2711	Observational
NCT04383652	Collection of Coronavirus COVID-19 Outbreak Samples in New South Wales	2712	Observational
NCT04383678	Outcome of COVID-19 Patients After Extracorporeal Membrane Oxygenation for Acute Respiratory Distress Syndrome	2713	Observational
NCT04383730	Inhaled Sedation in COVID-19-related Acute Respiratory Distress Syndrome (ISCA): an International Research Data Study in the Recent Context of Widespread Disease Resulting From the 2019	2714	Observational
NCT04383886	Evaluation of Emergency Department (ED) Staff Stress Level During COVID-19 Pandemic	2715	Observational
NCT04383899	Role of Ibuprofen and Other Medicines on Severity of Coronavirus Disease 2019	2716	Observational
NCT04384029	The Geneva Covid-19 CVD Study	2717	Observational
NCT04384042	Malaysian COVID-19 Anosmia Study (Phase 2) - A Nationwide Multicentre Case-Control Study	2718	Observational
NCT04384055	Predicting Outcomes for Covid-19 Using Sonography	2719	Observational
NCT04384250	Genetic Basis of COVID-19 Infection	2720	Observational
NCT04384406	COVID-19 Outbreak Consequences for Outpatients Followed in PRM	2721	Observational
NCT04384419	Death Number Perception in Depression, Anxiety, and Schizoypal Personnality in General Population (Covid-19 Pandemic)	2722	Observational
NCT04384471	Covid-19 Pandemic: Patients' Experience in T1D	2723	Observational
NCT04384887	"Investigation of the Relationship Between New Coronary Virus Disease (COVID-19) and Anxiety and Depressive Symptoms in Pregnant Women"	2724	Observational
NCT04384926	Outcomes of Elective Cancer Surgery During the COVID-19 Pandemic Crisis	2725	Observational
NCT04385004	Clinical, Biological and Histological Pulmonary and Renal Damage Associated With the SARS-CoV-2 Syndrome in Patients Admitted in ICU	2726	Observational
NCT04385069	COVID-19 Hyperinflammation Syndrome (COV-HI): Protocol for a Rapidly Executed Cohort Study	2727	Observational
NCT04385121	Psychological Impact of the Hospitalization of a Family Member in Intensive Care for Covid-19 Infection	2728	Observational
NCT04385147	Advanced Endoscopy During COVID-19	2729	Observational
NCT04385160	Myeloproliferative Neoplasms (MPN) and COVID-19	2730	Observational
NCT04385212	Outcomes in Hospitalized Older Patients With COVID-19	2731	Observational
NCT04385238	Health and Wellbeing of Pregnant and Post-Partum Women During the COVID-19 Pandemic	2732	Observational
NCT04385251	International SARS-CoV-2 (COVID-19) Infection Observational Study	2733	Observational
NCT04385823	Use of High Flow Nasal Cannula Oxygen and Covid-19 Acute Hypoxemic Respiratory Failure	2734	Observational
NCT04385914	Demographics and Outcomes of COVID 19 in Pregnancy at a Community Based Hospital	2735	Observational
NCT04386044	Investigating the Role of Vitamin D in the Morbidity of COVID-19 Patients	2736	Observational
NCT04386083	Neurologic Manifestations of COVID-19	2737	Observational

Registry entry ID	Title	Database ID (ID all)	Reason for exclusion
NCT04386109	Neonatal Complications of Coronavirus Disease (COVID-19)	2738	Observational
NCT04386369	Evaluation of Airway Pressure Release Ventilation in COVID-19 ARDS	2739	Observational
NCT04386395	Immune Changes in Severe COVID-19 Pulmonary Infections	2740	Observational
NCT04386460	Covid-19 and Prevention of Malnutrition After Confinement by Dentists	2741	Observational
NCT04386512	Clinical Epidemiology and Characteristics Of Covid-19 Cases Occurred In A Lymphoma Setting In The First Epidemic Phase (LymphoCov1)	2742	Observational
NCT04386551	Detection of COVID-19 in Saliva Collection	2743	Observational
NCT04386564	Kidney Injury Severity and COVID-19	2744	Observational
NCT04386720	Measurement of Airway Opening Pressure (AOP) in Patients With Acute Respiratory Distress Syndrom and With or Without Covid 19.	2745	Observational
NCT04386759	COVID-19 Infection in Healthcare Workers	2746	Observational
NCT04387214	COVID-19 Pandemic and Academic Performance of Veterinary Students	2747	Observational
NCT04387279	The Impact of COVID-19 on the Health Care Utilization of Inflammatory Bowel Disease	2748	Observational
NCT04387643	Protecting Health Care Workers During the COVID-19 Outbreak	2749	Observational
NCT04387656	NCI COVID-19 in Cancer Patients, NCCAPS Study	2750	Observational
NCT04387786	Dornase Alfa Administered to Patients With COVID-19 (DACOVID)	2751	Observational
NCT04387799	Negative-PCR for Covid- 19 Pneumonia (NPC-19)	2752	Observational
NCT04387838	Study of the SARS-COV-2 (COVID-19) Serological Profile of an Army Training Hospital Staff	2753	Observational
NCT04387890	Health Personnel Cohort for Surveillance of SARS-CoV-2 Infections (COVID-19)	2754	Observational
NCT04387968	Epidemiological Study of the Covid-19 Presto Test	2755	Observational
NCT04388579	Screening Patients for a Strategic Shift to Pulmonary Telerehabilitation Because of COVID-19	2756	Observational
NCT04388618	Investigating Anosmia and Ageusia in COVID-19 Adult Patients in Saudi Arabia	2757	Observational
NCT04388644	Survey of Satisfaction on Traditional Chinese Medicine Jing-Guan-Fang (JGF) for COVID-19 Prevention	2758	Observational
NCT04388657	COVID-19, bLOod Coagulation and Thrombosis	2759	Observational
NCT04388670	Epidemiological and Clinical Characteristics of Critically Ill Patients With COVID-19	2760	Observational
NCT04388813	Predictors of Severe COVID-19 Outcomes	2761	Observational
NCT04389320	Antimalarial and Covid 19 in Rheumatoid Arthritis	2762	Observational
NCT04389463	Patient Outcome of Cardiac Surgery During the COVID-19 Pandemic	2763	Observational
NCT04389476	The Impact and Coping Strategy of COVID-19 Among Taiwan Society and Medical and Nursing Institutes	2764	Observational
NCT04389489	Postpartum Sexual Function in Pregnant Women With COVID-19	2765	Observational
NCT04389515	Postoperative Recovery Index, Patient Expectations and Satisfaction With Prenatal Care Instrument in Pregnant Patients With COVID-19	2766	Observational
NCT04389567	Famotidine Outpatient COVID-19 Treatment Study	2767	Observational
NCT04389645	Interferon Gamma Induced Protein 10 (IP-10) in a Clinical Decision Support Protocol in COVID-19 Patients	2768	Observational
NCT04389658	Contagiousness and Immunogenicity Status of COVID-19 in Asymptomatic Population (COVID-19 CONDITION)	2769	Observational
NCT04389684	Clinical and Psycho-social Impact of COVID-19 Related Confinement on Patients With Digestive Tumors	2770	Observational
NCT04390074	COVID-19 in the Swedish ICU-cohort: Risk Factors of Critical Care Admission and Intensive Care Mortality	2771	Observational
NCT04390126	COVID-19 Related Lockdown Effects On Chronic Diseases	2772	Observational
NCT04390165	Malaysian COVID-19 Anosmia Study (Phase 1) - A Nationwide Multicentre Cross-Sectional Study	2773	Observational
NCT04390269	Immunogenetics Predictors With COVID-19	2774	Observational
NCT04390516	Respiratory Decompensation and Model for the Triage of COVID-19 Patients	2775	Observational
NCT04383457	Covid-19 Triage Using Camera-based AI	2776	Observational
NCT04384614	Covid-19 In Tunisia: An Observational Cross-Sectional Registry Study	2777	Observational
NCT04385797	Cognitive Outcomes During COVID-19 confiNemeNt in Elderly and Their Caregivers Using Technologies for DEMentia	2778	Observational
NCT04386265	Compassionate Use of Hyperbaric Oxygen Therapy	2779	Observational
NCT04387929	Detection of Anti-COVID-19 Antibody Levels in an Hospital Population	2780	Observational
NCT04388436	Post Covid-19 Cardiopulmonary and Immunological Changes	2781	Observational
NCT04388605	Assessing the Safety of Pregnancy In the CoRonavirus (COVID-19) pandEmic	2782	Observational
NCT04389554	D-dimer Levels in Pregnant With COVID-19	2783	Observational
NCT04389996	COVID-19 Pandemic Impact on Patients With Cancer - a Danish Survey	2784	Observational
NCT04390555	The Global PCHF-COVICAV Registry	2785	Observational
KCT0005005	Psychiatric Consultation for COVID-19 Patients	2786	Observational
ChiCTR2000032884	The duration and influencing factors of medical staff wearing disposable protective equipment in the isolation area during novel coronavirus pneumonia (COVID-19): an epidemiological study	2787	Observational
ChiCTR2000032895	Epidemiological, clinical and prognosticated features of novel coronavirus pneumonia (COVID-19) in Zhuhai	2788	Observational
ChiCTR2000033003	Novel coronavirus pneumonia (COVID-19) s-vax loaded autologous dendritic cell trial	2789	Observational
RBR-6w5tbk	Effects of tests for covid-19 on the preservation of the healthcare workforce during pandemia in Brazil	2790	No medical treatment intervention
DRKS00021582	A short, animated video to improve COVID-19 hygiene uptake: an online randomized experiment with 20,000 participants	2791	No medical treatment intervention
NCT04385576	Taiwan "Aerosol Box" Versus UMMC "Intubation Box"	2792	No medical treatment intervention
NCT04386070	Preventing Pulmonary Complications in Surgical Patients at Risk of COVID-19	2793	No medical treatment intervention
NCT04389333	Feasibility of Non-contact Magnetically-controlled Capsule Endoscopy During COVID-19 Pandemic	2794	No medical treatment intervention
NCT04386980	Study to Evaluate Resiniferatoxin in Patients With Knee Osteoarthritis Whose Total Knee Replacement Surgery is Delayed	2795	Not COVID-19 study
NCT04386291	Meditation and Kundalini Yoga for Heightened Anxiety Related to COVID-19	2796	No medical treatment intervention
ISRCTN88057279	Preventative treatment for patients at risk of COVID-19 infection (PROTECT)	2800	Duplicate
RPCEC00000309	Therapy for COVID-19-Phase I-II	2802	Cinical, not randomised
NCT04384549	Efficacy of BCG Vaccination in the Prevention of COVID19 Via the Strengthening of Innate Immunity in Health Care Workers	2807	Duplicate
NCT04385095	Trial of Inhaled Anti-viral (SNG001) for SARS-CoV-2 (COVID-19) Infection	2825	Duplicate
NCT04384731	Curosurf¬Æ in Adult Acute Respiratory Distress Syndrome Due to COVID-19	2838	Duplicate
JPRN-UMIN000040286	Study on risk perception and behavioral change related to COVID-19	2847	No medical treatment intervention
JPRN-jRCTs031200026	Combination therapy of Favipiravir and Nafamostat Mesilate in Patients with COVID-19 Pneumonia	2862	Duplicate
CTRI/2020/05/025171	Ayurveda protocol & COVID-19 exposed individuals: Clinical trial of an Ayurveda Intervention	2865	Duplicate
CTRI/2020/04/024915	A Phase II, Open Label, Randomized Controlled Trial to Assess the Safety and Efficacy of Convalescent Plasma to Limit COVID-19 Associated Complications	2870	Duplicate
CTRI/2020/05/025088	Study of GUDUCHI TABLET on healthy individuals to prevent covid 19.	2872	Duplicate
CTRI/2020/05/025166	Study of ASHWAGANDHA TABLET on healthy individuals to prevent covid 19	2873	Duplicate
CTRI/2020/05/025162	Effect of pranayama and meditation on psychological well-being of healthcare workers during COVID-19 pandemic.	2876	No medical treatment intervention
CTRI/2020/04/024775	Study to assess the efficacy and safety of convalescent plasma in moderate COVID-19 disease.	2882	Duplicate
NCT04391829	Detection of SARS-CoV-2 in Semen of COVID-19 Positive Males	2887	Clinical, not randomised
NCT04392323	Incidence of COVID-19 Test Conversion in Post-surgical Patients	2888	Clinical, not randomised
NCT04390412	Low Dose Radiotherapy in COVID-19 Pneumonia	2889	Clinical, not randomised
NCT04394182	Ultra Low Doses of Therapy With Radiation Applicated to COVID-19	2890	Clinical, not randomised
NCT04392453	Upper Limb Robotic Rehabilitation During COVID-19 Outbreak	2891	Clinical, not randomised
NCT04395911	Safety and Efficacy of SCD in AKI or ARDS Patients Associated With COVID-19 Infections	2892	Clinical, not randomised
NCT04394793	Low Dose Radiation Therapy for Covid-19 Pneumonia	2893	Clinical, not randomised
NCT04392232	A Study of COVID 19 Convalescent Plasma in High Risk Patients With COVID 19 Infection	2894	Clinical, not randomised
NCT04395716	A Study of ResCure‚Ñ¢ to Treat COVID-19 Infection	2895	Clinical, not randomised
NCT04393961	At-Home COVID-19 Antibody Test Usability Assessment & Behavior Change Study	2896	Cinical, not randomised
NCT04394169	Early Care Program for the Management of Post-ICU Syndrome and Chronic Pain After COVID-19 Infection.	2897	No medical treatment intervention
NCT04393818	App Intervention to Protect Mental Health in Healthcare Workers at Frontline Against COVID19: a Randomized Trial	2898	No medical treatment intervention
NCT04393636	Digital Cardiac Counseling Trial: DCC Trial	2899	No medical treatment intervention
NCT04394013	A Web-Based Gentle Body Stretching to Alleviate Loneliness and Improve Mental Fitness During COVID-19 Isolation Period	2900	No medical treatment intervention
NCT04394455	Cognitve Behaviorl Brief Therapy on Perceived Stress Level and Anxiety, Depression and Post Traumatic Stress Symptoms	2901	No medical treatment intervention
NCT04394403	Pilot Internet-based Self-Help Program for Managing Corona (COVID-19) Stress	2902	No medical treatment intervention
NCT04394039	Post-Pandemic Perception of Public Space in Singapore	2903	Cinical, not randomised
NCT04395508	An Expanded Access Study to Provide at Home Subcutaneous Administration of Pertuzumab and Trastuzumab Fixed-Dose Combination (PH FDC SC) for Patients With HER2-Positive Breast	2904	Cinical, not randomised
NCT04392245	Proctologic Practice and Covid-19	2905	Observational
NCT04393402	Lung Ultrasound Score in Covid 19 Infectious Disease in Critical Care (LUS-COVID19)	2906	Observational
NCT04393428	Urinary Biomarkers for AKI Diagnosis in Patients With SARS-CoV-2	2907	Observational
NCT04394078	Impact of Covid-19 Pandemic on Depression and Quality of Life	2908	Observational
NCT04394195	sFlt1: a Biomarker of Organ Dysfunction in Critically-ill Patients With COVID-19?	2909	Observational
NCT04396353	EXercise TRAining and Sedentary Lifestyle on Clinical Outcomes in Patients With COVID-19	2910	Observational
NCT04391738	Association Between BMI and COVID-19	2911	Observational
NCT04392388	Health, Perception, Practices, Relations and Social Inequalities in the General Population During the Covid-19 Crisis	2912	Observational
NCT04392401	COVID-19 - Study of the Kinetics of the Immune Response During the Intensive Care Unit Stay in Adult Patients Infected With SARS-CoV-2: Multicentric Non Interventional Study	2913	Observational
NCT04393233	Drug Management of Rheumatoid Arthritis in Covid-19 Context : Impact on Therapeutic De-escalation	2914	Observational
NCT04394104	COVID-19 Wellness Survey	2915	Observational
NCT04391166	Validity of at Home Visual Acuity Measurements for Telemedicine During Corona Virus (COVID-19) Pandemic	2916	Observational
NCT04395664	Arrhythmias in Patients With COVID-19	2917	Observational
NCT04391400	The Use of Peripheral Blood Specimens From Patients Suspected of Having SARS-CoV-2 Infections in Research Studies Intended to Support the Development of COVID-19 Detection Methods,	2918	Observational
NCT04391816	COVID-19 Pandemic Impact on Alcohol (PIA) - A Natural History Study	2919	Observational
NCT04395833	National Survey on the Lockdown of Children With Disabilities	2920	Observational
NCT04395859	Collateral Damage From the COVID-19 Pandemic Observed in Patients Treated With Intravitreal Injections (IVT) of Anti-angiogenic Agents	2921	Observational
NCT04393155	Longitudinal COVID-19 Cohort Study	2922	Observational
NCT04395872	Psychiatric Consultation for COVID-19 Patients	2923	Observational
NCT04395885	Sexual Function During COVID 19 Pandemic	2924	Observational
NCT04393558	Wearable Sensor to Monitor COVID-19 Like Signs and Symptoms	2925	Observational
NCT04393805	Heparins for Thromboprophylaxis in COVID Patients: HETHICO Study in Veneto	2926	Observational
NCT04394026	Imaging Feature of SARS-CoV2 Infection	2927	Observational
NCT04394429	Endotracheal Tube Obstruction in Covid-19 Patients Requiring Mechanical Ventilation	2928	Observational
NCT04394884	Pathogenesis of BTK-mediated Hyper-Inflammatory Responses in COVID-19 (RESPOND)	2929	Observational
NCT04395300	Sleep Quality in Healthcare Personnel During COVID-19	2930	Observational
NCT04395742	1,3,7-Trimethylxanthine as a Treatment of COVID-19: Results of a Controlled Study	2931	Observational
NCT04395755	Impact of COVID-19 on Psychological Status in Case of IVF Interrupted or Postponed	2932	Observational
NCT04396197	Physical Activity Levels in COVID-19 Patients Admitted to Intensive Care	2933	Observational
NCT04396210	Patients Perspectives on Discontinuation of Their Fertility Treatment Due to the COVID-19 Pandemic	2934	Observational
NCT04396600	The Professional Peer Resilience Initiative	2935	Observational
NCT04391881	Oral Manifestation of COVID 19 Patient: A Cross Sectional Study on Egyptian Population	2936	Observational
NCT04392089	Effects of Cardiovascular and Pulmonary Optimization on Cerebral Oxygenation in COVID-19 Patients With Severe ARDS	2937	Observational
NCT04393974	COVID-19 and Cancer Patients	2938	Observational
NCT04395391	Home Usability Study of the SARS-CoV-2 Test	2939	Observational
NCT04395482	Lung CT Scan Analysis of SARS-CoV2 Induced Lung Injury	2940	Observational
NCT04395924	Maternal-foetal Transmission of SARS-Cov-2	2941	Observational
NCT04388631	Detection Rate of SARS-CoV-2 in Male Genitourinary System and Its Impact on Male Reproductive Health.	2942	Observational
NCT04391686	Resting Energy Expenditure of the COronaVirus (COVID19) Patient in Reanimation Context	2943	Observational
NCT04391920	Registry of CytoSorb Therapy in COVID-19 ICU Patients	2944	Observational
NCT04395794	SARS-CoV-2 Disguise Study	2945	Observational
NCT04391946	Observatory of Patients With Chronic Lymphocytic Leukemia / Lymphocytic Lymphoma or Waldenstrom Disease Infected With COVID-19	2946	Observational
NCT04393142	Serologic Testing of Household Contacts of Confirmed Cases of COVID-19	2947	Observational
NCT04394000	Impact of an Intensified Thromboprofylaxis Protocol in COVID-19	2948	Observational
NCT04393324	The Critical Care Response to the COVID-19 Pandemic in Madrid	2949	Observational
NCT04394390	Do Vitamin D Levels Really Correlated With Disease Severity in COVID-19 Patients?	2950	Observational
NCT04395781	Pediatric Acute and Critical Care COVID-19 Registry of Asia	2951	Observational
NCT04393792	SINUS WASH Pilot Study in Adults Testing Positive for COVID-19	2971	Duplicate
NCT04396314	Effectiveness of Basic Body Awareness Therapy in Survivors of Covid-19 and Health Workers Regarding Post-traumatic Stress Disorders	2986	Clinical, not randomised
ChiCTR2000033104	Clinical study for rapid detection of novel coronavirus pneumonia (COVID-19) nucleic acid	2993	No medical treatment intervention
NCT04397835	Psychological Impact of COVID-19 Quarantine and Worsening of Cardiovascular Risk in the French General Population	2994	Observational
NCT04397900	Blood Collection Study From COVID-19 Convalescents to Identify Immunogenic Viral Epitopes	2995	Observational
NCT04399733	EMPOWER - BAME vs COVID	2996	Observational
NCT04397172	Intensive Care Associated Complications and Outcome of Acute Respiratory Distress Syndrome Due to COVID-19	2997	Observational
NCT04397380	COVID-19 Thales Thermography Triage : Thermal Camera Feasibility Study	2998	Observational
ACTRN12620000593932	Has the threat of SARS COVID-19 increased influenza vaccination rates in pregnant women?	2999	Observational
NCT04397237	Prevalence and Seroconversion of COVID-19 in Autoimmune Diseases in Europe	3000	Observational
NCT04397588	Extracorporeal Membrane Oxygenation (ECMO) and Coronavirus Disease (COVID) 19	3001	Observational
NCT04397614	Mobile Health Study and Enhanced Symptom Monitoring in COVID-19 Cancer Patients	3002	Observational
NCT04397848	Mental Health Outcomes in Healthcare Workers During COVID-19	3003	Observational
ISRCTN64783542	Investigating blood clotting changes in patients with COVID-19	3004	Observational
NCT04397822	COVID-19: Study of INFLAmmasome and PLAtelets Functions	3005	Observational
NCT04397939	Myocardial Injury and Major Adverse Outcomes in Patients With COVID-19	3006	Observational
NCT04399603	Mechanisms of Multi-organ Failure in COVID-19	3007	Observational

Registry entry ID	Title	Database ID (ID all)	Reason for exclusion
NCT04400461	Short-term Physical Function Outcomes in Severe COVID-19 Patients Admitted to ICU for Invasive Mechanical Ventilation	3008	Observational
ISRCTN10437835	Survival of patients suffering an out-of-hospital cardiac arrest treated by emergency services in Spain during the COVID-19 pandemic and 1 year later	3009	Observational
ISRCTN44324934	The symptoms, virology and immunity of COVID in healthcare workers	3010	Observational
NCT04400006	Ozone Therapy in the Prevention of COVID-19 Infection	3011	Observational
ACTRN12620000602921	Post-Intensive Care Nutrition Status in Patients with COVID-19	3012	Observational
ACTRN12620000609954	Covid-19 Biobank of clinical samples and data for use in research to help understand the immune characteristics of people who are diagnosed with COVID19.	3013	Observational
ACTRN12620000590965	Psychological impact of COVID-19 pandemic on perioperative staff at the Royal Melbourne Hospital ‚Äì a longitudinal study	3014	Observational
NCT04397575	The GCO-002 CACOVID-19 Cohort: a French Nationwide Multicenter Study of COVID-19 Infected Cancer Patients	3015	Observational
NCT04398264	Characteristics of COVID-19 Infection Among PREGnant Women	3016	Observational
NCT04400448	Acceptance of Telemedicine During the COVID-19 Pandemic	3017	Observational
ChiCTR2000033197	Investigation of Mental Status for in-patients with gastrointestinal malignancy during the COVID-19 Pandemic	3018	Observational
ChiCTR2000033108	Associations between suboptimal health status and TraditChinese Medicine constitution of medical staff fighting the disease, recovered COVID-19 patients and the general public under the	3019	Observational
ChiCTR2000033190	The research Of novel coronavirus pneumonia (COVID-19) patients' immune status and severity of disease	3020	Observational
ChiCTR2000033054	The investigation of psychosocial stress status in juveniles during the pandemic of the novel coronavirus pneumonia (COVID-19)	3021	Observational
ChiCTR2000033052	Acute Care Surgery during novel coronavirus pneumonia (COVID-19) pandemic in Spain	3022	Observational
ChiCTR2000033186	A follow-up study about clinically diagnosed cases of novel coronavirus pneumonia (COVID-19)	3023	Observational
ChiCTR2000033050	Effect of high dose intravenous vitamin C on moderate novel coronavirus pneumonia (COVID-19) patients: a medical records based retrospective study	3024	Observational
ChiCTR2000033200	The real-world study for the change of early breast cancer diagnosis and treatment mode under the pandemic of Coronavirus Disease 2019 (COVID-19)	3025	Observational
ChiCTR2000033198	Status of management of surgery in Beijing during COVID-19	3026	Observational
ChiCTR2000033107	Clinical characteristics and outcomes of novel coronavirus pneumonia (COVID-19) patients with pre-existing HBV infection	3027	Observational
ChiCTR2000033105	Follow up study of convalescent patients of novel coronavirus pneumonia (COVID-19)	3028	Observational
ChiCTR2000033055	Novel coronavirus pneumonia (COVID-19) and Symptomatic Venous Thromboembolism: a Multicenter Retrospective Study	3029	Observational
ChiCTR2000033053	Efficacy of Early Prone Positioning in Non-Mechanical Ventilation novel coronavirus pneumonia (COVID-19) Patients with Severe Hypoxia- a single-center prospective cohort study	3030	Observational
NCT04399005	The Efficacy Comparing Daily and After-each-case Room Disinfection.	3031	Cinical, not randomised
NCT04399109	Evaluation of a Remote Monitoring Smartphone Application and Care Model of COVID-19 Patients in the Community (ReCOVER)	3032	Cinical, not randomised
NCT04399746	Ivermectin-Azithromycin-Cholecalciferol (IvAzCol) Combination Therapy for COVID-19	3033	Cinical, not randomised
NCT04400032	Cellular Immuno-Therapy for COVID-19 Acute Respiratory Distress Syndrome - Vanguard	3034	Cinical, not randomised
EUCTR2020-001995-13-ES	Clinical trial to evaluate the efficacy and safety of tocilizumab for treating patients with COVID-19 pneumonia: the BREATH-19 Study	3035	Cinical, not randomised
EUCTR2020-002274-28-ES	Evaluation of the likely beneficial effects of vitamin D on infection with coronavirus.	3036	Cinical, not randomised
ISRCTN50212645	How effective are infection prevention and control measures that are informed by genetic analysis of coronavirus (rapid and standard) at preventing COVID-19 in NHS hospitals?	3037	Cinical, not randomised
ChiCTR2000033056	Convalescent Plasma in the treatment of 6 COVID-19 Patients	3038	Cinical, not randomised
ACTRN12620000579998	In a simulation environment, is there a difference in time taken for consultant anaesthetists to perform spinal anaesthesia as compared to general anaesthesia on women, with suspected or	3039	No medical treatment intervention
NCT04399681	The Utility of Bedside Lung Ultrasonography on Diagnosis of COVID-19	3040	Clinical, not randomised
NCT04397666	RT-PCR on Conjunctival Sample for the Detection of SARS-CoV-2 in Patients With Covid-19	3041	Clinical, not randomised
NCT04397705	Remote Monitoring of Cancer Patients With Suspected Covid-19	3042	Clinical, not randomised
NCT04398004	Anti-inflammatory Clarithromycin for Improving COVID-19 Infection Early	3043	Clinical, not randomised
NCT04397523	Efficacy and Safety of COVID-19 Convalescent Plasma	3044	Clinical, not randomised
NCT04399798	Baricitinib for coRona Virus pnEumonia (COVID-19): a THerapeutic Trial	3045	Clinical, not randomised
NCT04398277	A Daily Coping Toolkit for Medical Personnel and First-Responders During COVID-19 Pandemic	3046	Clinical, not randomised
NCT04399889	hCT-MSCs for COVID19 ARDS	3047	Clinical, not randomised
ChiCTR2000033049	A clinical study for the efficacy and safety of artemisinin-pipequine tablets in the treatment of the untreated mild or common type novel coronavirus pneumonia (COVID-19) patients	3048	Clinical, not randomised
ISRCTN13447477	A pilot study of the ability of povidone-iodine (PVP-I) 0.5% aqueous solution oral/nasal spray and mouthwash to kill the SARS-CoV-2 virus in people with COVID-19	3049	Clinical, not randomised
NCT04399967	Building Capacity and Promoting Smoking Cessation in the Community Via "Quit to Win" Contest 2020	3054	No medical treatment intervention
NCT04400305	"Increasing Physical Activity in Canadian Adults Who Have Been Affected by COVID-19 Social Distancing Restrictions: A Feasibility Trial of an Online Intervention"	3055	No medical treatment intervention
NCT04400279	The COVID-19 Pandemic and Exercise Study	3056	No medical treatment intervention
EUCTR2020-001759-42-GB	Investigating otilimab in patients with severe coronavirus related lung disease.	3071	Duplicate
EUCTR2020-001886-35-GB	A Clinical Trial of Nebulized Bovactant (Alveofact ¬Æ) for the Treatment of Moderate to Severe COVID-19	3073	Duplicate
ISRCTN15254871	Online parent training for reducing parenting stress during the coronavirus pandemic	3075	No medical treatment intervention
ChiCTR2000033057	A randomized controlled trial for determination of the clinical effect of Internet-assisted pulmonary rehabilitation on patients with novel coronavirus pneumonia (COVID-19)	3076	No medical treatment intervention
ChiCTR2000033133	A randomized, open-label, blank-controlled, multicenter trial for Shuang-Huang-Lian oral solution in the treatment of novel coronavirus pneumonia (COVID-19)	3077	Duplicate
NCT04401644	COVID-19: Laser Interferometry for Rapid Single Coronavirus Detection	3082	Cinical, not randomised
NCT04402918	Study of Viral Load and Maternal-fetal Serology in the Interpretation of the Vertical Transmission of SARS Cov-2 (COVID-19) During Pregnancy	3083	Cinical, not randomised
NCT04403906	Somerset and South Essex Coronavirus Antigen Testing	3084	Cinical, not randomised
NCT04401046	COVID-19 and Reorganisation of Cancer Patient Care: Self- Evaluation of Potential Post-traumatic Stress Disorder	3085	Cinical, not randomised
NCT04401410	Anti-SARS Cov-2 T Cell Infusions for COVID 19	3086	Cinical, not randomised
NCT04402840	Stellate Ganglion Block (SGB) for COVID-19 Acute Respiratory Distress Syndrome (ARDS)	3087	Cinical, not randomised
NCT04404192	PH94B in the Treatment of Adjustment Disorder With Anxiety	3088	Cinical, not randomised
NCT04403269	NORMAL HUMAN IMMUNOGLOBULINS (IVIG) IN PATIENTS AGED 75 YEARS AND OVER, COVID-19 WITH SEVERE ACUTE RESPIRATORY FAILURE	3089	Cinical, not randomised
NCT04402892	COVID-19: SARS-CoV-2 Specific Memory B and T-CD4+ Cells	3090	Cinical, not randomised
NCT04392869	The Effects of Mindfulness-based Training in Undergraduate Students of Translation and Interpreting	3091	No medical treatment intervention
NCT04402983	The Effectiveness of Telerehabilitation-Based Physiotherapy in COVID 19 Patients	3092	No medical treatment intervention
NCT04400812	Attitude and Perception Towards COVID-19 Pandemic	3093	Observational
NCT04401111	One-year Outcomes in Survivors of the Severe COVID-19 Pneumonia (CO-Qo-ICU)	3094	Observational
NCT04401124	Status of Management of Surgery in Beijing During COVID-19	3095	Observational
NCT04401228	Predictive Models for Intensive Care Admission and Death of COVID-19	3096	Observational
NCT04401254	Recovery of Patients From COVID-19 After Critical Illness	3097	Observational
NCT04401371	Distance Learning for Dental Student During COVID-19 Pandemic	3098	Observational
NCT04401241	How Routine Biomarkers and Blood Leucocytes Count Can Assist Diagnosis of COVID-19 in Emergency Department	3099	Observational
NCT04401449	Cardiopulmonary Inflammation and Multi-System Imaging During the Clinical Course of COVID-19 Infection in Asymptomatic and Symptomatic Persons	3100	Observational
NCT04401436	COVID-19 Associated Lymphopenia Pathogenesis Study in Blood (CALYPSO)	3101	Observational
NCT04400877	Prevalence and Severity of Venous Thromboembolism in a General Population During the COVID-19 Pandemic	3102	Observational
NCT04401072	Attitude of Egyptian Population Toward COVID 19 in Ramadan	3103	Observational
NCT04402229	Psychological Impact of the Covid-19 Pandemic on Student Nurses	3104	Observational
NCT04401085	COVID-19: Herd Immunity Study in the Czech Republic	3105	Observational
NCT04401098	Changing Of Egyptian Population Behaviour Towards COVID 19 Outbreak	3106	Observational
NCT04403061	Th1/Th2/Th17/TREG and TLRs Activation/KIR for COVID 19 Prediction of Outcome	3107	Observational
NCT04402905	Observational Study in Diagnosed Patients COVID-19, Supported on an Outpatient Basis. (COVID-PSL)	3108	Observational
NCT04402814	IgG/IgM Antibody Test in Patients Who Have Tested Negative or Positive for COVID-19 With Nasopharyngeal PCR Testing.	3109	Observational
NCT04402827	Different Susceptibility Among Health Care Workers Highly Exposed to Patients With COVID-19 Diagnosis.	3110	Observational
NCT04403386	Prospective Natural History Study of Smoking, Immune Cell Profiles, Epigenetics and COVID-19	3111	Observational
NCT04403438	Familial Mediterranean Fever and Beh√ßet: Analysis Before and After Covid19 Pandemic	3112	Observational
NCT04403009	The Clinical Difference Between the Nonfatal Patients and the Fatal Patients With Severe COVID-19	3113	Observational
NCT04403607	Cardiac Imaging in SARS-CoV-2 (COVID-19)	3114	Observational
NCT04403828	Impact of COVID-19 on Personal Protection Among Dentist in Egypt	3115	Observational
NCT04403581	Egyptian Doctors Stresses, Knowledge and Attitude During COVID-19 Pandemic	3116	Observational
NCT04403932	Increased Risk of Severe COVID-19 Infection in Patients With Vitamin D Deficiency	3117	Observational
NCT04404062	Validation of COVID-19 Tests	3118	Observational
NCT04404179	Setting up a COVID Care Facility at a Prison in Pakistan	3119	Observational
NCT04404270	Study of SARS-CoV2 Virus (COVID-19) Seroprevalence in the Population of Creil Air Force Base (BA110)	3120	Observational
NCT04403035	Comparison of the ID NOW and Accula Point-of-Care Assays for Detection of COVID-19	3121	Observational
NCT04404244	Extraordinary Measures for Egyptian Children With Hemato-Oncological Disorders During COVID-19 Pandemic	3122	Observational
NCT04403880	Characterizing SARS-CoV-2-specific Immunity in Individuals Who Have Recovered From COVID-19	3123	Observational
NCT04401540	Characteristics of Neonatal Covid-19 in Turkey	3124	Observational
NCT04403672	Performance Evaluation of RealDetect‚Ñ¢ COVID-19 RT-PCR Kit for the Detection of SARS-CoV-2 Virus	3125	Observational
NCT04402866	TD-0903 for ALI Associated With COVID-19	3145	Duplicate
RPCEC00000314	VA-MENGOC-BC to stimulate the innate response against SARS-CoV-2	3153	Cinical, not randomised
NCT04417270	Reliability of the Freestyle Libre CGM in the Inpatient Setting During the COVID-19 Surge	3154	Cinical, not randomised
NCT04405492	Evaluation of Rapid Diagnostic Solutions, Serological and Molecular Tests for COVID-19	3155	Cinical, not randomised
NCT04405544	Determination of Acute Encephalopathy Predictors in Patients With COVID-19	3156	Cinical, not randomised
NCT04408066	COVID-19: A POC Test Under Research & Evaluation	3157	Cinical, not randomised
NCT04420338	SeroCOVIDial Study: Assessment of COVID-19 Seroconversion in Patients on Chronic Hemodialysis and in Their Caregivers, a Cohort Study	3158	Cinical, not randomised
NCT04423991	Identification of a Responsive Subpopulation to Hydroxychloroquine in COVID-19 Patients Using Machine Learning	3159	Cinical, not randomised
NCT04416308	COVID 19 : Seroprevalence Study of Anti SARS-CoV-2 Antibodies in GHT Employees in Haute Bretagne (AntiCoV-HB)	3160	Cinical, not randomised
NCT04408456	Efficacy of Hydroxychloroquine (HCQ) as Post Exposure Prophylaxis (PEP) for Prevention of COVID-19	3161	Cinical, not randomised
NCT04435808	Efficacy of Hydroxychloroquine Prophylaxis for Health Care Workers at High Risk for COVID-19	3162	Cinical, not randomised
NCT04436471	An Open Study of the Safety, Tolerability and Immunogenicity of the Drug "Gam-COVID-Vac" Vaccine Against COVID-19	3163	Cinical, not randomised
NCT04437875	An Open Study of the Safety, Tolerability and Immunogenicity of "Gam-COVID-Vac Lyo" Vaccine Against COVID-19	3164	Cinical, not randomised
NCT04441684	Seroprevalence of SARS-CoV-2 in Strasbourg University Hospital, Strasbourg, France (COVID-19)	3165	Cinical, not randomised
NCT04406181	Psychological Well-being of Patients Awaiting for Cardiac Surgery During the COVID-19 Pandemic	3166	Cinical, not randomised
NCT04422353	Video Dance Class and Unsupervised Physical Activity During Covid-19 Pandemic in People With Parkinson's Disease	3167	Cinical, not randomised
NCT04408040	Use of Convalescent Plasma for COVID-19	3168	Cinical, not randomised
NCT04416139	Mesenchymal Stem Cell for Acute Respiratory Distress Syndrome Due for COVID-19	3169	Cinical, not randomised
NCT04427098	Enoxaparin in COVID-19 Moderate to Severe Hospitalized Patients	3170	Cinical, not randomised
NCT04432103	Treatment of Severe and Critical COVID-19 Pneumonia With Convalescent Plasma	3171	Cinical, not randomised
NCT04432272	ANTIBODY-LEVEL BASED ANALYSIS OF COVID CONVALESCENT SERUM (ABACCuS)	3172	Cinical, not randomised
NCT04422509	Lanadelumab for Treatment of COVID-19 Disease	3173	Cinical, not randomised
NCT04428073	Therapeutic Vaccine Trial of COVID-19 for Severe Acute Respiratory Syndrome Coronavirus 2 (SARS-CoV-2) Infection	3174	Cinical, not randomised
NCT04423042	Tocilizumab in Coronavirus-19 Positive Patients	3175	Cinical, not randomised
ChiCTR2000033372	Efficacy and safety of leflunomide for Refractory novel coronavirus pneumonia (COVID-19): A non-Randomized Controlled Study	3176	Cinical, not randomised
ACTRN12620000635965	Open label, prospective study for the Biofourmis Everion armband telemonitoring solution for patients during COVID-19 home isolation within South Western Sydney	3177	Cinical, not randomised
ACTRN12620000636954	COACHING FOR COVID-19: A Pilot Study Investigating the Effectiveness of Coaching on Psychological Outcomes in Hospital-Based Senior Doctors	3178	Cinical, not randomised
ACTRN12620000640909	A phase II, open label non-randomised clinical trial of the safety and efficacy of the CovidCare app to support self-monitoring for COVID-19 symptoms in self-isolation and to determine the	3179	Cinical, not randomised
IRCT20140428017469N1	Polio Vaccine and COVID-19	3180	Cinical, not randomised
IRCT20170211032494N3	Low dose whole lung irradiation for treatment of COVID-19 pneumonia	3181	Cinical, not randomised
IRCT20150303021315N19	Pembrolizumab (CinnaGen Co, Iran) in COVID-19	3182	Cinical, not randomised
IRCT20150107020592N25	Prone position effectiveness in Non invasive ventilation of covid-19 patients undergoing respiratory failure	3183	Cinical, not randomised
IRCT20150107020592N23	Plasmapheresis and plasma exchanges;usefulness in covid-19 critically ill patients	3184	Cinical, not randomised
IRCT20150107020592N22	Cyclosporin for the Prevention and Treatment of COVID-19 Disease	3185	Cinical, not randomised
IRCT20150107020592N24	Evaluation the effects of Oxycodone administration on pain control in patients with COVID-19	3186	Cinical, not randomised
IRCT20200517047485N1	Comparison of combination therapies of COVID_19	3187	Cinical, not randomised
IRCT20200519047510N1	Evaluation the anti-inflammatory effects of curcumin in the treatment of patients with COVID-19	3188	Cinical, not randomised
IRCT20200525047562N1	Treatment of Covid-19 patients with convalescent plasma	3189	Cinical, not randomised
IRCT20200523047550N1	Effect of Umifenovir in treatment of covid-19	3190	Cinical, not randomised
ChiCTR2000033936	Psychological Preparedness towards Novel Coronavirus Precautionary Measures ‚Äì an online survey	3191	Cinical, not randomised
ChiCTR2000033899	Impact of COVID-19 on Laboratory Professionals-Early Evidences from a Survey at a Clinical Chemistry Laboratory in a Developing Country	3192	No medical treatment intervention
NCT04420988	Investigational COVID-19 Convalescent Plasma Infusion for Severely or Life-threateningly Ill COVID-19 Patients	3193	No medical treatment intervention
ACTRN12620000634976	Testing an Online Coping Skills Intervention for People Living with Chronic Illness During the Covid-19 Pandemic	3194	No medical treatment intervention
RBR-43hbks	Evaluation with ultrasound on the bedside in serious patients with COVID-19	3195	Observational
RPCEC00000319	Biomodulina T - IM - covid-19 - older adult - Confirmatory study	3196	Observational
RPCEC00000318	Heberon Alfa R in COVID-19	3197	Observational
DRKS00022057	Traumatological emergency consultations during the COVID-19 pandemic 2020	3198	Observational
DRKS00021823	Survival and risk factors in Covid-19 and influenza, a retrospective analysis	3199	Observational
DRKS00021729	A prospective trial on the prevalence and the variation over time of SARS-CoV-2 antibodies in hospital health care workers (COVID-19)	3200	Observational
DRKS00021815	Investigation of the immune status against SARS-CoV-2 of medical professionals in Doctor's Practices in Thuringia (COVID-19 research project)	3201	Observational
DRKS00021424	Explorative Study of the Association of Oropharyngeal Microbiota Profiles with SARS-CoV-2 Infection and COVID-19 disease	3202	Observational
DRKS00021810	The role of immune-reactions in patients with COVID-19	3203	Observational
DRKS00021709	Serial cross-sectional seroprevalence study of SARS-CoV-2 infection during and after the COVID-19 epidemic in Heidelberg and the Rhein-Neckar region	3204	Observational
DRKS00021468	Exploring immunological control mechanisms of SARS-CoV-2 (COVID-19)	3205	Observational
DRKS00021757	Impact of the COVID-19 Pandemic on Symptoms of Obessive Compulsive Disorder	3206	Observational
DRKS00021698	Prospective COVID-19 Cohort Munich	3207	Observational
DRKS00021772	Covid-19 related Obstetric Anaesthesia Longitudinal Assessment-Registry	3208	Observational
DRKS00021763	Influence of the Corona regulation on the physical activity of hospital staff - COVID-19	3209	Observational
DRKS00021574	Investigation of the predictive value of coagulation parameters for the course of disease in COVID-19 patients	3210	Observational
DRKS00022054	CovidCare 1 ‚Äì Surveillance and care for confirmed and suspected patients with COVID-19 in general practice: an observational study	3211	Observational

Registry entry ID	Title	Database ID (ID all)	Reason for exclusion
DRKS00022088	Prevalence of SARS-CoV-2 (COVID-19) in pregnancy and at delivery in Franconia, Germany	3212	Observational
DRKS00021653	Retrospective examination of the course of a Covid-19 infection	3213	Observational
DRKS00022071	Psychosocial functioning and stress factors of psychiatric patients during the COVID-19 pandemic: Telephone survey of patients in the psychiatric outpatient clinic of the Leipzig University	3214	Observational
DRKS00021752	Study platform ArcHIV - Substudy on SARS-CoV-2 (COVID-19) IgG antibody prevalence in an HIV cohort	3215	Observational
DRKS00021791	The Impact of quarantine and social distancing on mental health in the context of the Covid-19 pandemic	3216	Observational
DRKS00021775	COVID-19 treatment outcomes at the University Medical Center Freiburg	3217	Observational
ChiCTR2000033719	Hip Fracture in elderly people during COVID-19 State of Alarm in Spain: reports from a specialized Center	3218	Observational
ChiCTR2000033919	A medical records based study for the influence of novel coronavirus pneumonia (COVID-19) on the symptoms of patients with achalasia	3219	Observational
ChiCTR2000033580	Analysis of Characteristics of recurrent positive RT-PCR findings of recovered patients from novel coronavirus pneumonia (COVID-19) in Wuhan, China: a medical records based study	3220	Observational
ChiCTR2000033980	A follow-up study of novel coronavirus pneumonia (COVID-19) patients in Wanzhou District, Chongqing	3221	Observational
ChiCTR2000033517	Effectiveness of day care service on improving the physical and psychological well-being of people with dementia and easing the caregiving burden in COVID-19 pandemic	3222	Observational
ChiCTR2000033364	A correlation study between food intake and risk factors of PTSD after the COVID-19 of shift medical staff	3223	Observational
ChiCTR2000033346	Erythrocyte Sedimentation Rate in novel coronavirus pneumonia (COVID-19) patients	3224	Observational
ChiCTR2000033609	A medical records based study for nano short peptide nutrition intervention on the prognosis of patients with novel coronavirus pneumonia (COVID-19)	3225	Observational
ChiCTR2000033830	Development and Application of a Low-Cost Ear-Contactless Stethoscope Powered by Raspberry Pi and Python	3226	Observational
ChiCTR2000033798	The efficacy and safety of convalescent plasma therapy in novel coronavirus pneumonia (COVID-19): A medical records based retrospective cohort study	3227	Observational
ChiCTR2000033705	A medical records based study for the preliminary efficacy of Tocilizumab in the treatment of the patients with novel coronavirus pneumonia (COVID-19)	3228	Observational
ChiCTR2000033960	A multicenter clinical study: transmission of COVID-19 and impact on health and developmental outcomes among newborns of mothers with confirmed/suspected COVID-19	3229	Observational
ISRCTN55886926	Investigation of COVID-19 symptoms and potential immunity in the general population	3230	Observational
ISRCTN15571919	Investigating the impact of COVID-19 and its response, on people with severe mental illness in South Asia	3231	Observational
TCTR20200527001	Endotoxin and circulating bacteriome in severe COVID-19 patients	3232	Observational
ISRCTN14447421	COVID-19 in care homes (VIVALDI)	3233	Observational
JPRN-UMIN000040405	International survey to investigate the implementation of ICU daily care for COVID-19	3234	Observational
JPRN-UMIN000040453	Corona virus disease 2019 (COVID-19) antibody seroprevalence of the medical staff in the hospital with cluster of COVID-19.	3235	Observational
JPRN-UMIN000040516	Usefulness of PCR testing for COVID-19 before gastrointestinal endoscopy and hospitalization	3236	Observational
JPRN-UMIN000040520	Examination in COVID-19 pandemic; Whether endoscopies really generate aerosol particles?	3237	Observational
JPRN-UMIN000040563	Prospective observational study on cytokine removal effect of continuous hemodiafiltration in COVID-19	3238	Observational
JPRN-UMIN000040587	The analysis of COVID-19 in saliva, gastric fluid and intestinal fluid on gastrointestinal endoscopy	3239	Observational
JPRN-UMIN000040598	Clinical Outcomes of COVID-19 Infection in Hospitalized Patients with Cardiovascular Diseases and/or Risk Factors: CLAVIS-COVID	3240	Observational
JPRN-UMIN000040614	A retrospective study on the therapeutic effect of tocilizumab on COVID-19	3241	Observational
JPRN-UMIN000040616	Retrospective study on clinical and pathological aspects of COVID-19	3242	Observational
JPRN-UMIN000040617	A retrospective study on serum markers in the diagnosis and differentiation of COVID-19	3243	Observational
JPRN-UMIN000040679	prospective cohort study of COVID-19 antibody for medical stuff in hospital	3244	Observational
JPRN-UMIN000040683	Longitudinal study on work style change, health and well-being due to pandemic of novel coronavirus infection (COVID-19)	3245	Observational
JPRN-UMIN000040451	COVID 19 - What Families Think and Do? A Survey for Families Visiting the Pediatric Emergency Department	3246	Observational
ISRCTN12394803	DIAMONDS-SEARCH study: Designing new tests that can diagnose the causes of fever, including COVID-19	3247	Observational
ISRCTN60400862	Evaluation of a COVID-19 antibody test: What is the performance of the Panbio‚Ñ¢ COVID-19 IgG/IgM rapid test device in fingerstick blood, venous whole blood, serum and plasma in adult	3248	Observational
ISRCTN15324611	A study to assess the usefulness of combination of Vitamin D, magnesium and Vitamin B12 in older COVID-19 patients	3249	Observational
ISRCTN69546370	Best available treatment study for inflammatory conditions associated with COVID-19	3250	Observational
ISRCTN13471603	Management of patients with heart failure with preserved ejection fraction: what do patients and providers need and want?	3251	Observational
ISRCTN93266696	Studying the immune response and long-term morbidity of COVID-19 infection in pregnancy	3252	Observational
ISRCTN11952463	What‚Äôs the STORY: serum testing of representative youngsters including SARS-CoV-2 (COVID-19), diphtheria and meningitis C	3253	Observational
ISRCTN59048638	Sulodexide in the treatment of COVID-19	3254	Observational
ISRCTN18274006	Understanding cognitive decline after stroke and the impact of COVID-19	3255	Observational
RBR-4qjzh7	Study of vascular complications in critically ill patients with coronavirus	3256	Observational
ACTRN12620000620921	Angiotensin II Infusion in COVID-19-Associated Vasodilatory Shock: A multinational, multicentre registry	3257	Observational
ACTRN12620000641998	Ventricular Arrhythmias in Implantable Cardioverter Defibrillator Patients During the COVID-19 Pandemic	3258	Observational
ACTRN12620000692932	Atrial Fibrillation in Patients with an Implantable Cardiac Device during the COVID-19 Pandemic	3259	Observational
RBR-62y3h7	Acute impairment of renal function in COVID-19: study on incidence, risk factors and mortality	3260	Observational
ISRCTN15475176	A Saudi Arabia based study into the impact of coronavirus infection on the nervous system	3261	Observational
ChiCTR2000033347	Epidemiological and Clinical Characteristics of 20 Re-positive novel coronavirus pneumonia (COVID-19) Patients	3262	Observational
ChiCTR2000033345	Study for the effect of Traditional Chinese medicine in the prevention of novel coronavirus pneumonia (COVID-1) in the high risk area	3263	Observational
ChiCTR2000033342	The comprehensive evaluation of therapeutic drugs for pediatric novel coronavirus pneumonia (COVID-19)	3264	Observational
ChiCTR2000033319	A follow up research of exercise, cardiopulmonary and quality of Life for patients of novel coronavirus pneumonia (COVID-19)	3265	Observational
ChiCTR2000033317	Psychiatric admissions during the COVID-19 pandemic	3266	Observational
ChiCTR2000033514	A Survey Among Collage Students for Changes and impact factors of Lifestyle, Psychology and Weight During Keep Stay at Home for four months in the Pandemic of Novel Coronavirus	3267	Observational
ChiCTR2000033872	Serum protein marker in patients with novel coronavirus pneumonia (COVID-19)	3268	Observational
ChiCTR2000033782	A medical records based retrospective study of novel coronavirus pneumonia (COVID-19) patients	3269	Observational
ChiCTR2000033781	A medical records based study for predictors of novel coronavirus pneumonia (COVID-19) elderly patients	3270	Observational
ChiCTR2000033780	A medical records based study for analysis of Tongue Features of novel coronavirus pneumonia (COVID-19) Patients	3271	Observational
ChiCTR2000033745	Clinical study on the effect of Clarenicol oral liquid on the relief of pharyngeal symptoms of novel coronavirus pneumonia (COVID-19) in convalescence, suspected patients and other susceptible	3272	Observational
ChiCTR2000033723	A study for long-term prognosis in hospitalized novel coronavirus pneumonia (COVID-19) patients	3273	Observational
ChiCTR2000033720	Clinical observation of the relieving effect of Clarenicol oral liquid on the throat symptoms of novel coronavirus pneumonia (COVID-19) in convalescence, suspected patients and other	3274	Observational
ISRCTN55201674	B-cells in stroke-associated infection	3275	Observational
ACTRN12620000617965	Multi-site audit of COVID-19 airway interventions	3276	Observational
NCT04405362	Consequences of the QUARANTINE Relating to the COvid-19 Epidemic on the Mental Health of the Patients Followed in PSYchiatry	3277	Observational
NCT04405869	Thromboembolic Events in Severe COVID-19 Patients	3278	Observational
NCT04406038	Study of the Spread of COVID-19 in Saint Petersburg, Russia	3279	Observational
NCT04406090	Endovascular Thrombectomy in COVID-19 Infected Patients	3280	Observational
NCT04406233	Muscle Mass and Strength as Predictors of Time to Discharge in Patients With COVID-19	3281	Observational
NCT04406324	COVID-19: Respiratory and Sleep Follow-up	3282	Observational
NCT04406571	Reorganization of the Healthcare System During COVID-19 Pandemic: Impact on Management of Patients With Exocrine Pancreatic Cancer	3283	Observational
NCT04406688	Prediction of Acute Kidney Injury in Patients With COVID-19	3284	Observational
NCT04406844	An Observational Study to Identify the Issues and Challenges in Cancer Patients on Active Treatment During the COVID-19 Pandemic and the Resulting Lockdown	3285	Observational
NCT04407221	COVID-19 Research in Organ Transplant Recipients	3286	Observational
NCT04407273	STATIN THERAPY AND COVID-19 INFECTION	3287	Observational
NCT04407299	COVID-19 Impact on Rheumatic Patients	3288	Observational
NCT04407429	Vienna Versus SARS-CoV-2 Virus Study	3289	Observational
NCT04407468	Awake Prone Positioning and Oxygen Therapy in Patients With COVID-19	3290	Observational
NCT04407533	Dietary Intake and Eating Habits During the COVID-19 Pandemic	3291	Observational
NCT04407572	Evaluation of the Relationship Between Zinc Vitamin D and b12 Levels in the Covid-19 Positive Pregnant Women	3292	Observational
NCT04407585	Testing the Accuracy of a Digital Test to Diagnose Covid-19	3293	Observational
NCT04407598	COPD in the Time of COVID-19	3294	Observational
NCT04408014	Seroepidemiological Study of SARS-CoV-2 (COVID-19) Infection in Population Subgroups in the State of S√£o Paulo	3295	Observational
NCT04408222	Awake Proning in COVID-19 Patients With Hypoxemic Respiratory Failure	3296	Observational
NCT04408365	Endothelial Function, Inflammation and Organ Dysfunction in COVID-19	3297	Observational
NCT04408391	Brain MRI Imaging in Patients With SARS-Cov2 (COVID-19) Infection With or Without Anosmia	3298	Observational
NCT04405973	Clinical Scores for Outcome Prediction in Patients With Severe COVID-19 Pneumonia Requiring ECMO	3299	Observational
NCT04406545	Microvascular Flow and Reactivity in Patients Presenting in the Acute Phase of COVID-19.	3300	Observational
NCT04407143	Study of the Immunity of Patients With Lung Cancer and COVID-19 Infection	3301	Observational
NCT04407169	Prevalence of Critical Forms of CoVid-19 in Patients With Chronic Respiratory Disease	3302	Observational
NCT04407195	COVID-19 and Mental Health	3303	Observational
NCT04407260	The Use of Oxygen Hoods as Compared to Conventional High-flow Oxygen Delivery Systems, the Effects on Oxygenation, Intubation and Mortality Rates in Hypoxic Patients With COVID-19.	3304	Observational
NCT04407494	Diagnostic Value of Patient - Reported and Clinically Verified Olfactory Disorders (COVID-19)	3305	Observational
NCT04407559	COVID-SErology in Rheumatoid Arthritis (COVID-19)	3306	Observational
NCT04407663	Outpatient Clinic in Obesity Care During COVID-19 Outbreak: Physically Far, Virtually Near	3307	Observational
NCT04407923	Drug Management of Juvenile Idiopathic Arthritis in Covid-19 Context : Impact on Therapeutical Managment	3308	Observational
NCT04408170	Facilitating AcceLerated Clinical Validation Of Novel Diagnostics for COVID-19 (FALCON-C19)	3309	Observational
NCT04408196	QoL and the Emotional-affective Sphere o in Rehabilitation Setting During COVID-19 Quarantine	3310	Observational
NCT04408378	Change in the Ratio of Mean Platelet Volume (MPV) to Platelet(PLT) in Covid-19 Pneumonia Patients	3311	Observational
NCT04409184	Whole Blood Collection From Individuals in the Convalescent Phase of SARS-CoV-2 Infection	3312	Observational
NCT04409197	Evaluation of Changes in Weight, Sleep, and Other Psycho-behavioural Parameters During Covid-19 Confinement in Subjects Monitored by the RNPC Network	3313	Observational
NCT04409535	Effects of the COVID-19 Health Emergency on Biopsychosocial Health	3314	Observational
NCT04409847	COVID-19 Blood Pressure Endothelium Interaction Study (OBELIX)	3315	Observational
NCT04409899	The Utility of Enhanced Pre and Post-surgical Work-out to Prevent the Spread of COVID-19 in a Large Urology Department	3316	Observational
NCT04409951	Pharmacy Education During Covid-19 Outbreak	3317	Observational
NCT04410107	Lung Function, Exercise Capacity and Health-Related Quality of Life After Severe COVID-19	3318	Observational
NCT04410263	Microbiota in COVID-19 Patients for Future Therapeutic and Preventive Approaches	3319	Observational
NCT04410718	Glycaemia and Cardiac Function in Patients With COVID-19	3320	Observational
NCT04410939	COVID-19 PCR Test Results in Asymptomatic Pregnants	3321	Observational
NCT04411511	COVID-19, Obesity and Lifestyle in Children	3322	Observational
NCT04412226	Effect of Using Barrier Devices for Intubation in COVID-19 Patients	3323	Observational
NCT04412369	Multi-modality Imaging & Immunophenotyping of COVID-19 Related Myocardial Injury	3324	Observational
NCT04412460	Echocardiography in Patients With Covid-19	3325	Observational
NCT04412473	COVID-19 Respiratory Distress and Antithrombotic Drugs in Subject's Habits	3326	Observational
NCT04413058	Menstrual Cycle Characteristics of Healthcare Professionals	3327	Observational
NCT04408326	Efficacy and Safety of Angiotensin II Use in Coronavirus Disease(COVID)-19 Patients With Acute Respiratory Distress Syndrome	3328	Observational
NCT04409574	COVID 19 Viral Clearance Among the Infected Healthcare Workers In Assiut University Hospitals	3329	Observational
NCT04410484	PREPARE-IBD: Physician Responses to Disease Flares and Patient Adaptation in Relation to Events in Inflammatory Bowel Disease During COVID-19 Pandemic	3330	Observational
NCT04411459	Risk Factors for Prolonged Invasive Mechanical Ventilation in COVID-19 Acute Respiratory Distress Syndrome	3331	Observational
NCT04411576	Current and Past SARS-CoV-2 Infection and COVID-19 in Healthcare	3332	Observational
NCT04411784	PROTECT-ASUC: Covid-19 Pandemic Response Of assessmenT, EndosCopy and Treatment in Acute Severe Ulcerative Colitis	3333	Observational
NCT04412031	Prediction of Unfavourable Outcome in Newly Covid-19 Hospitalized Patient	3334	Observational
NCT04412746	Covid-19 and Diabetes in West of Algeria	3335	Observational
NCT04413045	Novel COVID-19, A National Analysis	3336	Observational
NCT04414059	Protein Electrophoresis as a Tool for Complications Prediction in COVID-19 Hospitalised Patients	3337	Observational
NCT04414410	Echocardiography in Critically-ill Patients With COVID-19 Pneumonia	3338	Observational
NCT04414904	Determining the Reproductive Health of Men Post-COVID-19 Infection	3339	Observational
NCT04416061	The Role of a Private Hospital in Hong Kong Amid COVID-19 Pandemic	3340	Observational
NCT04416100	Development of Interstitial Lung Disease (ILD) in Patients With Severe SARS-CoV-2 Infection (COVID-19)	3341	Observational
NCT04416256	COVID-19 Pandemic and Worldwide Organ Procurement	3342	Observational
NCT04416373	COVID-19 and Pregnancy Outcomes	3343	Observational
NCT04416438	COVID-19 Epidemic and Patients With Myeloproliferative Neoplasias	3344	Observational
NCT04418544	Vocal Feature Analysis Algorithm	3345	Observational
NCT04408001	Study of Kinetics and Efficacy of the Immune Response Against COVID-19 Among Hospital Staff	3346	Observational
NCT04412265	Frailty in Elderly Patients With COVID-19	3347	Observational
NCT04417374	Caracteristics of the Patients Hospitalized With Unvonluntary Commitment Procedure, in the Context COVID-19	3348	Observational
NCT04418193	Patient Preference Trial for COVID-19 (PPT-COVID)	3349	Observational
NCT04418245	CT Biomarkers Identification by Artificial Intelligence for COVID-19 Prognosis	3350	Observational
NCT04418375	Establishment of a Biological Collection During COVID-19 Serological Screening in APHP Professionals (COVID-HOP)	3351	Observational
NCT04418609	Neuro-COVID-19: Neurological Complications of COVID-19	3352	Observational
NCT04419571	Peri-Operative NLR in Emergency General Surgery During COVID-19 Pandemic	3353	Observational
NCT04420286	Study of the Increase in ICU Beds Capacity During COVID-19 Pandemic in France	3354	Observational
NCT04420312	Cohort Comparing the Prevalence and Characteristics of Pulmonary Embolism on COVID-19 Patients Presenting Respiratory Symptoms	3355	Observational
NCT04420416	Mental Health and Academic Performance in COVID-19	3356	Observational
NCT04420468	Descriptive and Retrospective Analysis of Acute Myocarditis Associated With Pandemic COVID-19 in Children	3357	Observational
NCT04420637	Impact of the Covid-19 Pandemic on Gastrointestinal and Liver Diseases	3358	Observational
NCT04422379	COVID-19 and Liver Injury in Patients With or Without Underlying Liver Disease: A Multi-centre Retrospective-prospective Observational Study	3359	Observational
NCT04422418	Impact of Burnout on Cardiovascular and Immune Biomarkers in Healthcare Professionals - Covid-19 Pandemic in Abu Dhabi	3360	Observational
NCT04422535	Effects on the Qt Interval of COVID-19 Coronavirus Infection	3361	Observational
NCT04422691	Ultrasonography in Norwegian Emergency Department Patients With Suspected Covid-19 Infection	3362	Observational
NCT04422769	Needs of Persons With Spinal Cord Injury (SCI)	3363	Observational
NCT04423315	Thrombosis Risk Assessment and Clinical Presentation of Covid-19 Pneumonia	3364	Observational
NCT04423692	Knowledge About Covid-19 Infection	3365	Observational

Registry entry ID	Title	Database ID (ID all)	Reason for exclusion
NCT04405466	A Study to Assess the Feasibility of Screening for SARS-CoV-2 Among Healthy Workers	3366	Observational
NCT04407117	Appendicitis During the National Lockdown During the COVID-19 Pandemic	3367	Observational
NCT04407546	Role of Children in Transmission of COVID-19 to Immunocompromised Patients	3368	Observational
NCT04408248	AKI Biomarkers in Coronavirus(COVID)-19	3369	Observational
NCT04409249	Prognosis in Pregnant With COVID-19	3370	Observational
NCT04410835	Psychological Impact of the Corona Virus (SARS-CoV-2) and COVID-19 Pandemic in Individuals With Psychiatric Disorders	3371	Observational
NCT04411147	A Longitudinal Study of COVID-19 Sequelae and Immunity	3372	Observational
NCT04412278	Impact of COVID-19 Pandemic on Perceived Exercise Benefits and Barriers	3373	Observational
NCT04412304	Anticoagulant Therapy and 28-days Mortality in Critically Ill COVID-19 Patients	3374	Observational
NCT04412655	Primary Angioplasty for STEMI During COVID-19 Pandemic (ISACS-STEMI COVID-19) Registry	3375	Observational
NCT04413071	Cardiac COVID-19 Health Care Workers	3376	Observational
NCT04413435	Clinical Characteristics of Critically Ill Patients With COVID-19	3377	Observational
NCT04415359	Evaluation of the Evolution of Pregnancies in the First Trimester Following MAR Management During a COVD-19 Pandemic Period	3378	Observational
NCT04416347	COVID19 Clinical Predictors and Outcome	3379	Observational
NCT04416464	Quality of Life and Patient-centered Outcomes After ICU Admission for COVID-19	3380	Observational
NCT04417673	Living With Sickle Cell Disease in the COVID-19 Pandemic	3381	Observational
NCT04418284	Online Learning of Veterinary Anatomy During COVID-19 Pandemic	3382	Observational
NCT04422340	Senior-COVID-Rea Multicentric Survey	3383	Observational
NCT04422522	Fibromyalgia During the COVID-19 Pandemic	3384	Observational
NCT04422574	COVID 19 Serology in Pulmonologists	3385	Observational
NCT04422626	Data Analysis of the Cytokine Adsorption Treatment on Coronavirus Disease-19 (COVID-19) Patients With Respiratory Failure	3386	Observational
NCT04422873	The Impact of COVID-19 on Dialysis Users	3387	Observational
NCT04423640	Analysis of the Inflammatory Response and the Development of Humoral and Cellular Immunity in Patients With COVID-19	3388	Observational
NCT04423770	COVID-19 Related Health and Infection Control Practices Among Dentists	3389	Observational
NCT04423978	Implications of Covid-19 on the Lifestyle Changes	3390	Observational
NCT04424004	MURDOCK Cabarrus County COVID-19 Prevalence and Immunity (C3PI) Study	3391	Observational
NCT04424017	Sero-prevalence of Coronavirus Disease 2019 (COVID-19) in Healthcare Workers	3392	Observational
NCT04424355	Magnetic Resonance Imaging to Detect Signs of Viral Pneumonia in Patients With Coronavirus Infection.	3393	Observational
NCT04424446	Saliva/Nares as Source of Detection for SARS-CoV-2 at NIH	3394	Observational
NCT04424771	Post-traumatic Stress Disease in Health Workers During COVID-19 Pandemia	3395	Observational
NCT04424836	HFNC Treatment in COVID-19 Pneumonia	3396	Observational
NCT04424849	Thorax Computed Tomography Severity Score and Outcome in COVID-19 Patients	3397	Observational
NCT04424992	The Natural History of Hospitalized COVID-19 Patients	3398	Observational
NCT04425044	AiM Covid Self Monitoring	3399	Observational
NCT04425213	Obesity and Mortality of Critically Ill Patients With COVID-19	3400	Observational
NCT04425382	Darunavir/Cobicistat vs. Lopinavir/Ritonavir in COVID-19 Pneumonia in Qatar	3401	Observational
NCT04425850	USEFULNESS of Topic Ivermectin and Carrageenan to Prevent Contagion of Covid 19	3402	Observational
NCT04425863	IVERMECTIN Aspirin Dexametasone and Enoxaparin as Treatment of Covid 19	3403	Observational
NCT04425889	COVID-19 Antibodies Among Healthcare Workers	3404	Observational
NCT04426071	The Impact of a Societal Lockdown on Those With Spinal Cord and Brain Injuries	3405	Observational
NCT04426253	Identification of Genetic Factors Determining Disease Course in the New Type of Coronavirus Infection, COVID-19	3406	Observational
NCT04426266	Psychological Effects of the COVID-19 Pandemic on the Hungarian Adult Population	3407	Observational
NCT04426279	Morbimortality of Covid-19 in Patients With Chronic Inflammatory Rheumatism Treated With Immunosuppressants	3408	Observational
NCT04427176	Evaluation of ARFC Masks Equipped With CF5 Filter in the Care Unit to Allow a Wider Distribution of FFP2 Masks (Covid-19).	3409	Observational
NCT04427267	Assessment of Thermal Condition of Health Workers Who Use Personal Protective Equipment From Biological Hazards During Their Work With COVID-19 Patients	3410	Observational
NCT04427280	Cancer: Rapid Diagnostics and Immune Assessment for SARS-CoV-2 (COVID-19)	3411	Observational
NCT04427332	Smell and Taste Disorders in COVID-19 Patients	3412	Observational
NCT04427345	Predictive Factors COVID-19 Patients	3413	Observational
NCT04427358	RT-PCR SARS-CoV-2 at 1 Month of COVID-19 Infection in the Geriatric Population	3414	Observational
NCT04427735	Effect of Covid-19 Epidemic on Primary PCI in Patients With STEMI	3415	Observational
NCT04429815	Impact of Smoking and Nicotine on the Risk of Being Infected With COVID-19	3416	Observational
NCT04430049	Impact of Covid-19 Restrictive Measure on Anxiety, Depression and PTSD for Relatives of ICU Patients	3417	Observational
NCT04430062	Short-term Outcome of Covid-19 Surgical Patients: Case-series of a High-risk Area Community Hospital	3418	Observational
NCT04430322	Covid-19 at the Regional Medical Center Metz-Thionville: a Descriptive Study	3419	Observational
NCT04431284	Impact of COVID-19 Lockdown on Obesity and Eating Behaviors	3420	Observational
NCT04431310	Seroconversion Among Staff at a Large Acute Care Hospital in Denmark During the COVID-19 Pandemic	3421	Observational
NCT04431414	A Study of Immune Responses to the Virus That Causes COVID-19	3422	Observational
NCT04431908	Validation of a Rapid Quantitative Test for Loss of Smell in COVID-19 Subjects	3423	Observational
NCT04432779	Impact of COVID-19 Infection During Pregnancy on Newborns and Young Children	3424	Observational
NCT04432870	Patients' Preferences About Rescheduling Colonoscopies Delayed Due to COVID-19: Cross Sectional Study	3425	Observational
NCT04432922	Perceptions, Representations and Experiences of Septic Isolation of Hospitalized Patients for COVID-19 Infection	3426	Observational
NCT04432948	Maternal Psychological Implications of Covid-19 Pandemic and Possible Effect on Anaesthetic Management.	3427	Observational
NCT04432961	Natural Language Processing (NLP) Analysis of Free Text Notes to Investigate Coronavirus (COVID-19)	3428	Observational
NCT04433000	Reliability of a Pocket Sized Ultrasound Scanner in the Evaluation Covid-19 Pneumonia	3429	Observational
NCT04433026	BNP, Serum Troponin and D-dimer as Risk Factors in Patients With COVID-19	3430	Observational
NCT04433039	COVID19 Versus H1N1: Radiological Challenge During Next Influenza Season Rising	3431	Observational
NCT04433260	COVID19 and Physical and Emotional Wellbeing of HCP	3432	Observational
NCT04433286	COVID-19 - Implications on Surgeons' Burnout and Career Satisfaction	3433	Observational
NCT04433325	COVID-19 Critically Ill Patients' Evolution After Medical Transport by Train From Paris Intensive Care Units to West of France Intensive Care Units	3434	Observational
NCT04433754	The Development of Pancreatic Injury in the Course of Severe Acute Respiratory Syndrome Coronavirus 2 Infection	3435	Observational
NCT04434118	Hydroxychloroquine May Reduce Risk of COVID-19 Infection in Rheumatoid Arthritis Patients:	3436	Observational
NCT04434157	Association of the Neutrophil/Lymphocyte Ratio With Clinical Complications and Mortality in COVID-19 Patients	3437	Observational
NCT04434261	Oncological Surgery in Times of COVID-19: Effectiveness of Preoperative Screening for Sars-Cov-2	3438	Observational
NCT04434417	Validation of an Immunochromatographic Assay for IgG/IgM Antibodies to 2019- nCoV	3439	Observational
NCT04435028	Ketotifen: as a Cardioprotective Agent in Breast Cancer Patients Receiving Anthracycline-containing Chemotherapy	3440	Observational
NCT04435080	Physical Rehabilitation in ICU in ARDS Patients With COVID-19	3441	Observational
NCT04435106	Compassionate Use of Opaganib in Patients With Coronavirus Disease 2019 (COVID-19)	3442	Observational
NCT04435119	Covid-19 and Vitamin D in Nursing-home	3443	Observational
NCT04435223	Lipid Metabolism in COVID-19 Severe Pneumonia Compared With Severe Pneumonia Caused by Other Pathogen	3444	Observational
NCT04435327	Lung Damage Caused by SARS-CoV-2 Pneumonia (COVID-19)	3445	Observational
NCT04436484	Immune Biomarkers of Outcome From COVID-19	3446	Observational
NCT04437121	Dietary and Lifestyle Habits During the Pandemic of COVID-19 in Greece	3447	Observational
NCT04437342	Postpartum Depression in the Covid-19 Pandemic and the Impact of Anaesthesia	3448	Observational
NCT04437706	Network-Targeted Strategies for Efficient Community SARS-CoV-2 (COVID-19) Sampling	3449	Observational
NCT04437940	Evaluation of Vaginal Fluid for Covid-19 Positivity in Women With Positive Nasofarengeal Covid-19 Test	3450	Observational
NCT04438239	Rehabilitation Needs After COVID-19 Hospital Treatment	3451	Observational
NCT04438629	Evaluation of Immune Response in COVID-19 Patients	3452	Observational
NCT04438863	Daily Home Spirometry for Early Detection of Pulmonary Complications in Patients With COVID19	3453	Observational
NCT04438941	Contribution of a Prone Team in Intensive Care During the Covid-19 Epidemic	3454	Observational
NCT04438954	Investigation of Fatigue, Physical Activity, Sleep Quality and Anxiety Levels	3455	Observational
NCT04438967	Brain Health in the Time of COVID-19	3456	Observational
NCT04438993	The COVID-19 Disease and CARdiac Events Study	3457	Observational
NCT04439084	COVID-19 in Patients With Chronic Liver Diseases	3458	Observational
NCT04440098	Isolated During COVID-19: Effects of COVID-19's Social Restrictions on Loneliness and Psychosocial Symptomatology	3459	Observational
NCT04441489	Assessment of Lung Inflammation With FDG PET/CT in COVID-19	3460	Observational
NCT04441502	Identification of Predictors for the Evolution of COVID-19 Related Pneumonia by Transcriptomic and Seroproteomic	3461	Observational
NCT04441814	FOUND - Ancillary Study to Smile Protocol NCT03654105	3462	Observational
NCT04442165	Characterising Transmission of SARS-CoV-2 in a Peri-urban Population in Mozambique	3463	Observational
NCT04442204	Predictors and Mechanisms of Depression and Anxiety During the COVID-19 Pandemic	3464	Observational
NCT04442217	Factors Associated With Adherence to Hygiene-related Behaviors and Viral Mitigation Protocols During the COVID-19 Pandemic	3465	Observational
NCT04442243	PTSD Symptoms Among Health Workers and Public Service Providers After the COVID-19 Outbreak: A 3 Month Follow up	3466	Observational
NCT04442282	Difficulties in Emotion-regulation and Interpersonal Problems During and After the COVID-19 Pandemic	3467	Observational
NCT04442308	Parental Stress After the Covid-19 Pandemic	3468	Observational
NCT04442360	The Impact of Information Sources on Mental Health During the COVID-19 Pandemic	3469	Observational
NCT04442386	Parental Burnout During the COVID-19 Pandemic: Risk Factors and Predictors	3470	Observational
NCT04410471	Humoral Immunity Against SARS-CoV-2 in Liver Transplanted Patients After COVID-19 in Comparison With Immunocompetent Patients	3471	Observational
NCT04411563	Epigenetic Tools as Prognostic Predictors in Covid19	3472	Observational
NCT04412382	Covid-19: Possible Role of Neutrophil Extracellular Traps	3473	Observational
NCT04413877	Implementation of the Integrated Care of Older People App and ICOPE Monitor in Primary Care (ICOPE)	3474	Observational
NCT04412551	Lung Ultrasound for Assessment of Patients With Moderate to Severe Covid-19	3475	Observational
NCT04413955	Registry of Seraph¬Æ-100 Microbind¬Æ Affinity Blood Filter for COVID-19 Under EUA	3476	Observational
NCT04416009	Extracellular Water in Covid 19 Pneumonia	3477	Observational
NCT04418557	COVID-19 and Obstetric Transmission	3478	Observational
NCT04408339	COVID-19 in Cancer Patients: Evaluation of Clinical Course and Impact on Oncological Care Including Biobanking	3479	Observational
NCT04412317	Use of PCR-Sars-CoV-2 in Children	3480	Observational
NCT04422470	Observational Prospective Cohort Study - Registry of Patients With Hematologic Disease and COVID-19 in Russia	3481	Observational
NCT04422587	Evaluation of the COVID-19 Infection Response in Patients Admitted to the Emergency Department for Dyspnea	3482	Observational
NCT04423003	Endoscopic Interventions in Patients With COVID-19	3483	Observational
NCT04424940	COVID-19 Lessons Learned: the Outcome of TOMEKA¬Æ Project is to Teach	3484	Observational
NCT04425759	Prevalence and Risk Factors of SARS-CoV-2 Antibody Responses (COVID-19)	3485	Observational
NCT04426084	Cardiovascular Risk Factors and Severe COVID-19. A Nationwide Registry-based Case-Control Study	3486	Observational
NCT04429477	Cerebral Compliance Impairment in COVID-19	3487	Observational
NCT04430374	Registry for a Cardiovascular Patient Who COVID-19 Infection	3488	Observational
NCT04431869	In-Utero Vascular Accidents in Neonates From COVID-19 Infected Mothers	3489	Observational
NCT04433312	Impact of Cancellation of Non-urgent Surgical Cases on Patients During the COVID-19 Pandemic	3490	Observational
NCT04433364	COPE - COVID-19 in Pregnancy and Early Childhood	3491	Observational
NCT04433871	COVID-19 in Pediatric Oncology and Hematology Centers in France	3492	Observational
NCT04435041	Remote-by-Default Care in the COVID-19 Pandemic	3493	Observational
NCT04435457	Cardiovascular Implications of COVID-19	3494	Observational
NCT04437901	COVIDAR - Arrhythmias in COVID-19	3495	Observational
NCT04406493	Monitoring COVID-19 Patients' Lung Fluid Using Impedance Technique	3496	Clinical, not randomised
NCT04408131	Morbi-mortality by COVID-19 Among Homeless People in Marseille: a Cohort Study	3497	Clinical, not randomised
NCT04410549	Pulmonary Optical Coherence Tomography in COVID-19 Patients	3498	Clinical, not randomised
NCT04413968	COVID-19 Infection and Transmission in Exposed, Confined and Community-based Infants	3499	Clinical, not randomised
NCT04422613	Characterization of Persistent Pulmonary Abnormalities Following COVID-19 Pneumonia	3500	Clinical, not randomised
NCT04425317	Detection of SARS-CoV-2 in Follicular Fluid and Cumulus-oocyte-complexes in COVID-19 Patients	3501	Clinical, not randomised
NCT04432805	Descriptive and Evaluation Study of the Use of Pulmonary Ultrasound in the Initial Management of Pregnant Women in the Context of COVID-19	3502	Clinical, not randomised
NCT04405986	Exploring Brain Damages After COVID-19 Infection	3503	Clinical, not randomised
NCT04407208	Convalescent Plasma Therapy in Patients With COVID-19	3504	Clinical, not randomised
NCT04426292	SARS-COV-2 Seroprevalence and Seroconversion Among Employees of the Universitair Ziekenhuis Brussel During the 2020 COVID-19 Outbreak	3505	Clinical, not randomised
NCT04412239	To Assess the Efficiency and Satisfaction of Pharmacist Giving Patient-centred Advice on Telephone Consultation in TB Patients: Experience of the COVID-19 Pandemic	3506	Clinical, not randomised
NCT04413864	Immunomodulatory Profile of Dexmedetomidine Sedation in Patients Recovering After ARDS Covid-19	3507	Clinical, not randomised
NCT04416360	Autism Spectrum Disorder or Attention Deficit Hyperactivity Disorder in Covid-19 Outbreak	3508	Clinical, not randomised
NCT04410016	Evaluation of Staff COVID-19 Wellbeing Centres in a Healthcare Workplace: COVID-WELL Study	3509	Clinical, not randomised
NCT04427878	Is Adipose Tissue a Reservoir for SARS-Cov2 Spread in Covid-19 Patients?	3510	Clinical, not randomised
NCT04437719	Prospective Cohort Study to Describe the Clinical Characteristics of COVID-19, the Acquired Immune Response and the Biological and Clinical Parameters of Patients Followed in Oncology by	3511	Clinical, not randomised
NCT04416919	Assessment of N-95 Facemask for Use in COVID-19 Pandemic in Case of Shortage of Personal Protective Equipment	3512	Clinical, not randomised
NCT04418206	Validating an ELISpot for Early Detection of an Active Immune Response Against COVID-19	3513	Clinical, not randomised
NCT04426006	Serological Response to SARS-Cov-2 Virus in Personnel of the Institut Bergoni√© in the Context of the COVID-19 Pandemic - PRO-SERO-COV	3514	Clinical, not randomised
NCT04429724	Health Professional Exposure Assessment to Covid-19	3515	Clinical, not randomised
NCT04434131	Treatment With Investigational Convalescent Plasma and Measure Antibody Levels in Patients Hospitalized With COVID-19	3516	Clinical, not randomised
NCT04421625	Patients and Health Staff of Cancer Centres During the Covid-19 Pandemic	3517	Clinical, not randomised
NCT04409821	Tele-based Psychological Emotional Support for Informal CARegivers of COVID-19 Patients in Intensive Care	3518	Clinical, not randomised
NCT04413747	Yoga Pranayama Adjuvant to Treat Burden COVID -19	3519	Clinical, not randomised

Registry entry ID	Title	Database ID (ID all)	Reason for exclusion
NCT04424082	External Dead Space in Ventilated COVID-19 Patients	3520	Clinical, not randomised
NCT04429828	COVID-19 Psychological Wellbeing for Healthcare Students	3521	Clinical, not randomised
NCT04431297	Virtual Mindfulness Rounds in the Time of COVID-19 Pandemic	3522	Clinical, not randomised
NCT04406064	Viral Specific T-cells for Treatment of COVID-19	3523	Clinical, not randomised
NCT04406246	Prevention of Coronavirus Disease (COVID-19) Outbreaks With Nitazoxanide	3524	Clinical, not randomised
NCT04407286	Vitamin D Testing and Treatment for COVID 19	3525	Clinical, not randomised
NCT04408027	Mental Health Support for SickKids Children and Families During COVID-19 Using Established eHealth Interventions	3526	Clinical, not randomised
NCT04408209	Convalescent Plasma for the Treatment of Patients With Severe COVID-19 Infection	3527	Clinical, not randomised
NCT04408287	Improving Activity Engagement Among Persons With SCI During COVID-19	3528	Clinical, not randomised
NCT04410536	Home Detox in Medication Overuse Headache (MOH) During Covid-19 Emergency	3529	Clinical, not randomised
NCT04411602	Feasibility Study of Anti-SARS-CoV-2 Plasma Transfusions in COVID-19 Patients With SRD	3530	Clinical, not randomised
NCT04412486	COVID-19 Convalescent Plasma (CCP) Transfusion	3531	Clinical, not randomised
NCT04412785	Cyclosporine in Patients With Moderate COVID-19	3532	Clinical, not randomised
NCT04413006	Self-compassion for Chronic Pain Virtual Group Treatment Program	3533	Clinical, not randomised
NCT04414098	Ruxolitinib in the Treatment of Covid-19	3534	Clinical, not randomised
NCT04418531	Convalescent Antibodies Infusion in COVID 19 Patients	3535	Clinical, not randomised
NCT04420182	COVID-19 Virtual Care at Home	3536	Clinical, not randomised
NCT04420390	Low Dose Radiotherapy for COVID-19 Pneumonitis	3537	Clinical, not randomised
NCT04421391	QuadraMune(TM) for Prevention of COVID-19	3538	Clinical, not randomised
NCT04425616	SafeFit Trial: Virtual Clinics to Deliver Universal Interventions in People With Cancer	3539	Clinical, not randomised
NCT04409925	DISmantling COvid iNduced Neutrophil ExtraCellular Traps (DISCONNECT-1)	3540	Clinical, not randomised
NCT04412330	Optimizing Outcomes With Physical Therapy Treatment for IndividuALs Surviving an ICU Admission for Covid-19	3541	Clinical, not randomised
NCT04414293	Low Dose Pulmonary Irradiation in Patients With COVID-19 Infection of Bad Prognosis	3542	Clinical, not randomised
NCT04425538	A Phase 2 Trial of Infliximab in Coronavirus Disease 2019 (COVID-19).	3543	Clinical, not randomised
NCT04427137	Accelerated LFR for Bipolar Patients During the COVID-19 Pandemic	3544	Clinical, not randomised
NCT04427566	Low Dose Whole Lung Radiation Therapy for Patients With COVID-19 and Respiratory Compromise	3545	Clinical, not randomised
NCT04427631	Exploring Interventions for Glue Ear During Covid-19	3546	Clinical, not randomised
NCT04431453	Study to Evaluate the Safety, Tolerability, Pharmacokinetics, and Efficacy of Remdesivir (GS-5734,Ñ¢) in Participants From Birth to < 18 Years of Age With Coronavirus Disease 2019 (COVID-	3547	Clinical, not randomised
NCT04441632	Effect of Positive Attitudes on Behavior and Wellness	3548	Clinical, not randomised
NCT04419610	RAS and Coagulopathy in COVID19	3549	Clinical, not randomised
NCT04424901	Trial of Open Label Dipyridamole- In Hospitalized Patients With COVID-19	3550	Clinical, not randomised
NCT04435717	Efficacy of Tocilizumab in Modifying the Inflammatory Parameters of Patients With COVID-19 (COVITOZ-01)	3551	Clinical, not randomised
NCT04438694	Use of Convalescent Plasma for Treatment of Patients With COVID-19 Infection	3552	Clinical, not randomised
NCT04419623	A Study of TL-895 With Standard Available Treatment Versus Standard Available Treatment for the Treatment of COVID-19 in Patients With Cancer	3553	Clinical, not randomised
DRKS00022067	An intensive longitudinal study on habit formation of hand washing behavior during the COVID-19 pandemic	3554	Clinical, not randomised
RBR-876qb5	Treatment of severe cases of Covid-19 with Eculizumab	3555	Clinical, not randomised
EUCTR2020-001481-11-DE	Treatment with Ruxolitinib in Covid-19 patients with severe symptoms	3556	Clinical, not randomised
EUCTR2020-001546-19-BE	A First-in-Human Study in Adult Subjects Hospitalized with Covid-19 to Investigate a new product called ARGX-117	3557	Clinical, not randomised
EUCTR2020-001732-10-DE	Ruxolitinib for treatment of Covid-19 Patienten with acute respiratory	3558	Clinical, not randomised
EUCTR2020-001038-36-DE	Study to investigate the safety and effects of vaccines in healthy adults.	3559	Clinical, not randomised
JPRN-UMIN000040479	Immunological efficacy of lactic acid bacteria for COVID-19	3560	Clinical, not randomised
JPRN-UMIN000040694	SUrveillance of SARS-CoV-2 Virus Antibody in Health CarE and CloSe-Contact WorkerS	3561	Clinical, not randomised
JPRN-jRCTs032200044	Clinical safety and usefulness of the simple ventilator	3562	Clinical, not randomised
ISRCTN89951424	A phase III study to investigate a vaccine against COVID-19	3563	Clinical, not randomised
ISRCTN37530113	Enhanced cognitive behavioral therapy for children and adolescents with obsessive compulsive disorder: an open trial	3564	Clinical, not randomised
RBR-2dxyx4	Mechanical ventilator for use in patients with respiratory failure due to Covid-19 (VExCO)	3565	Clinical, not randomised
IRCT20200223046586N2	web- based communication between nurse and family member	3566	No medical treatment intervention
ISRCTN94091479	Behavioural Activation in Social IsoLation (BASIL): Benefiting from activities to improve your mood while you are socially isolating	3567	No medical treatment intervention
DRKS00022083	Evaluation of three commercially available rapid assays for detection of IgG and/or IGM antibodies to SARS-CoV-2 (COVID-19) for assessment of contact persons,Äô immune status and	3568	No medical treatment intervention
NCT04415879	Effects of a N95 Respirator vs Cloth Mask on Exercise Capacity During Treadmill Exercise.	3569	No medical treatment intervention
NCT04435275	Virtual Assistant for COVID-19 PPE	3570	No medical treatment intervention
NCT04414371	Tools for Wellbeing COVID-19 National Study of Undergraduate Students	3571	No medical treatment intervention
NCT04410458	Recruit Blood Donors Via SMS During Epidemic of COVID-19 (Repeat Trial)	3572	No medical treatment intervention
NCT04431180	Recruit Blood Donors Via SMS During Epidemic of COVID-19 #Second Repeat Trial#	3573	No medical treatment intervention
NCT04412343	The Seniors COvid-19 Pandemic and Exercise Study	3574	No medical treatment intervention
NCT04442152	Impact of COVID-19 on Family Dynamics on Bandebereho Study Participants	3575	No medical treatment intervention
NCT04409743	Brief Telehealth CBT-I Intervention in the Context of the COVID-19 Pandemic	3576	No medical treatment intervention
NCT04420273	Targeted Melanoma Detection With Skin Self-Examination During COVID-19 Restricted Physician Access	3577	Not COVID-19 study
NCT04387409	Study to Assess VPM1002 in Reducing Healthcare Professionals' Absenteeism in COVID-19 Pandemic	3578	No medical treatment intervention
NCT04435379	Study to Assess VPM1002 in Reducing Hospital Admissions and/or Severe Respiratory Infectious Diseases in Elderly in COVID-19 Pandemic	3579	No medical treatment intervention
NCT04423289	Telematic Monitoring Through a Mobile Application of Patients With COVID-19	3580	No medical treatment intervention
NCT04425720	Use of Remote Monitoring for COVID-19 Patient	3581	No medical treatment intervention
NCT04426305	Community Health Workers Against COVID19	3582	No medical treatment intervention
NCT04406532	Home-Based Covid-19 Rehabilitation Program	3583	No medical treatment intervention
NCT04409496	Chat-based Support for Preveting Smoking Relapse	3584	No medical treatment intervention
NCT04420806	Effects of COVID-19 Lockdown in Exercising Early Postmenopausal Women	3585	No medical treatment intervention
NCT04425005	Home-based Exercise Training During Covid-19 Pandemic in Post-bariatric	3586	No medical treatment intervention
NCT04428099	Healthy Lifestyle Promotion as Adjunctive Teletherapy for Treatment-resistant Major Depression	3587	No medical treatment intervention
NCT04416113	Photodynamic Therapy for the Treatment of COVID-19	3588	No medical treatment intervention
ChiCTR2000033261	Usefulness of abdominal massage and ST-36 (Zusanli) acupressure in improving appetite of novel coronavirus pneumonia (COVID-19) patients treated in makeshift hospital	3589	No medical treatment intervention
ISRCTN12107048	Supporting weight management during COVID-19	3590	No medical treatment intervention
IRCT20150716023235N15	sex hormones in coronavirus	3591	No medical treatment intervention
NCT04433910	A Clinical Trial of Convalescent Plasma Compared to Best Supportive Care for Treatment of Patients With Severe COVID-19	3613	Duplicate
NCT04418947	Project Resurgence Communication Trial	3614	No medical treatment intervention
NCT04410692	Can the Prediction Market Improve Predictions of COVID-19?	3619	No medical treatment intervention
NCT04430608	Remote Continues Glucose Monitoring During the COVID-19 Pandemic in Quarantined Hospitalized Patients	3621	Not COVID-19 study
NCT04425499	A Gamified Network for Surgical Education During COVID-19: A Randomized Controlled Trial	3622	No medical treatment intervention
NCT04432051	The Use of Lung Ultrasonography in COVID-19 Patients	3623	No medical treatment intervention
NCT04416334	PREEMPTIVE THERAPY WITH COLCHICINE IN PATIENTS OLDER THAN 70 YEARS WITH HIGH RISK OF SEVERE PNEUMONIAE DUE TO CORONAVIRUS	3629	Duplicate
NCT04431856	CO-PARENT: COVID-19 - Parent Action in Response to Emotions and Needs for Treatment	3632	No medical treatment intervention
NCT04417335	Reducing COVID-19 Related Hospital Admission in Elderly by BCG Vaccination	3641	Duplicate
NCT04438070	COVID Screening Strategies in Homeless Shelters	3643	No medical treatment intervention
NCT04429529	Safety of TY027, a Treatment for COVID-19, in Humans	3661	Not COVID-19 study
NCT04438980	Glucocorticoids in COVID-19 (CORTIVID)	3664	Duplicate
NCT04408157	A Research Study to Evaluate the Benefits of a Self-management Booklet to Promote Wellbeing During the COVID-19 Pandemic	3669	No medical treatment intervention
NCT04409483	Evaluation of Additional Treatments for COVID-19: a Randomized Trial in Niger	3670	Cancelled
NCT04418128	Clinical Efficacy of Nafamostat Mesylate for COVID-19 Pneumonia	3682	Duplicate
NCT04418505	Vielight RX Plus for the Treatment of COVID-19 Respiratory Symptoms	3683	No medical treatment intervention
NCT04418518	A Trial of CONvalescent Plasma for Hospitalized Adults With Acute COVID-19 Respiratory Illness	3684	Duplicate
NCT04425707	Ivermectin In Treatment of COVID 19 Patients	3692	Cinical, not randomised
NCT04432324	Study to Evaluate the Safety and Efficacy of High Dose IVIG in Hospitalized Participants With Coronavirus Disease (COVID-19)	3695	Duplicate
NCT04410562	Hydroxychloroquine Efficacy and Safety in Preventing SARS-CoV-2 Infection and COVID-19 Disease Severity During Pregnancy	3709	Duplicate
NCT04420741	Infusion of Prostacyclin (Iloprost) vs Placebo for 72-hours in COVID-19 Patients With Respiratory Failure	3719	Duplicate
NCT04423861	Efficacy and Safety of Nitazoxanide 600 mg TID for the Treatment of Hospitalized Patients With COVID-19	3720	Duplicate
NCT04425629	Safety, Tolerability, and Efficacy of Anti-Spike (S) SARS-CoV-2 Monoclonal Antibodies for the Treatment of Ambulatory Adult Patients With COVID-19	3721	Duplicate
NCT04425772	A Clinical Trial for Azvudine in the Treatment of Novel Coronavirus Pneumonia (COVID-19)	3722	Duplicate
NCT04426201	InterLeukin-7 to Improve Clinical Outcomes in Lymphopenic pAtients With COVID-19 Infection (ILIAD-7-US-O)	3723	Duplicate
NCT04442178	InterLeukin-7 to Improve Clinical Outcomes in Lymphopenic pAtients With COVID-19 Infection (ILIAD-7-US-I)	3734	Duplicate
NCT04426318	COVID-19 and the Healthy Minds Program for Educators	3737	No medical treatment intervention
RBR-9y32yy	Acute and chronic cardiovascular and respiratory changes in COVID-19 hospitalized patients and the effect of physical rehabilitation supervised by telecommunication	3767	No medical treatment intervention
EUCTR2020-001264-28-HU	A study to investigate whether the experimental medication IMU-838 is safe and effective when it is given to Covid-19 patients in addition to their normal treatment	3769	Duplicate
EUCTR2020-002287-31-ES	COVID 19 prevention in nursing homes	3771	Duplicate
EUCTR2020-002448-21-GR	BCG VACCINATION TO PREVENT COVID-19	3772	Duplicate
EUCTR2020-001573-78-FR	Recombinant InterLeukin-7 (CYT107) to Improve clinical outcomes in lymphopenic pAtients with COVID-19 infection	3774	Duplicate
EUCTR2020-001673-75-FR	Phase 2/3 study of ABX464, once daily oral capsule, in high risk patients infected by SARS-CoV-2 prior to COVID-19 associated respiratory distress.	3775	Duplicate
EUCTR2020-001623-13-GR	Triiodothyronine for the treatment of critically ill patients with COVID-19 infection (Thy-Support Study)	3776	Duplicate
EUCTR2020-001597-30-GB	A study to test the effectiveness and safety of bacteria called Bifidobacterium breve in patients with COVID-19 infections	3777	Duplicate
EUCTR2020-001512-26-DE	Hydroxychloroquine for the treatment of mild COVID-19 disease	3780	Duplicate
EUCTR2020-001264-28-GR	A study to investigate whether the experimental medication IMU-838 is safe and effective when it is given to Covid-19 patients in addition to their normal treatment	3782	Duplicate
EUCTR2020-001264-28-BG	A study to investigate whether the experimental medication IMU-838 is safe and effective when it is given to Covid-19 patients in addition to their normal treatment	3783	Duplicate
EUCTR2020-001870-32-DK	Study to assess efficacy and safety of DFV890 in patients with COVID-19 pneumonia and impaired respiratory function	3787	Duplicate
EUCTR2020-001951-42-PL	Amiodarone or Verapamil in COVID-19 hospitalized patients with symptoms: Randomized clinical trial	3788	Duplicate
EUCTR2020-001644-25-FR	A Phase 2 Randomized Study of the Efficacy and Safety of Acalabrutinib with Best Supportive Care Versus Best Supportive Care in Subjects Hospitalized with COVID-19	3794	Duplicate
EUCTR2020-001754-21-FR	An open prospective randomized therapeutic trial using ANAKINRA or TOCILIZUMAB alone or in combination with RUXOLITINIB in severe stage 2b and 3 COVID-19 disease	3795	Duplicate
EUCTR2020-001963-10-FR	Interleukin-1 (IL-1) and Interferon gamma (IFNg) inhibition during COVID 19 inflammation: Randomized, controlled study assessing efficacy and safety of Anakinra and Ruxolitinib	3796	Duplicate
EUCTR2020-001497-30-FR	ravulizumab for the treatment of patients with Coronavirus Disease 2019	3797	Duplicate
EUCTR2020-002130-33-BE	Zilucoplan¬Æ in patients with acute hypoxic respiratory failure due to COVID-19	3800	Duplicate
EUCTR2020-001889-10-GB	Treating COVID-19 infections with inhaled corticosteroids	3801	Duplicate
EUCTR2020-001644-25-DE	A Phase 2, Open-Label, Randomized Study of the Efficacy and Safety of Acalabrutinib with Best Supportive Care Versus Best Supportive Care in Subjects Hospitalized with COVID-19	3803	Duplicate
EUCTR2020-001928-34-SE	Treatment with inhalation of Ciclesonide for patients with coronavirus disease: A randomised open study.	3804	Duplicate
EUCTR2020-001497-30-GB	ravulizumab for the treatment of patients with Coronavirus Disease 2019	3805	Duplicate
ISRCTN99916292	CROWN CORONATION: Chloroquine repurposing to healthworkers for novel coronavirus mitigation	3809	Duplicate
NCT04405934	COG-UK Project Hospital-Onset COVID-19 Infections Study	3810	Cinical, not randomised
ISRCTN30564012	STOP-COVID19: Superiority Trial Of Protease inhibition in COVID-19 (coronavirus infection)	3812	Duplicate
ISRCTN11384058	A collaborative pilot study to monitor and optimise access to urgent surgery during the period of reduced resources in Genoa, Italy caused by the COVID-19 pandemic, using a bespoke referral	3813	Cinical, not randomised
ChiCTR2000033433	Study for the Effect of Internet-based Cognitive Behavior Intervention on Perinatal Depression and Anxiety during the novel coronavirus pneumonia (COVID-19) pandemic	3815	No medical treatment intervention
ChiCTR2000033320	Study for Traditional Chinese Medicine in the treatment of novel coronavirus pneumonia (COVID-19)	3816	Cinical, not randomised
ChiCTR2000033318	Efficacy of Liu-zi-jue in Patients with 2019 Novel Coronavirus Pneumonia (COVID-19): a randomized controlled trial	3817	Duplicate
ChiCTR2000033284	Study for the improvement of the quality of life of severe patients of novel coronavirus pneumonia (COVID-19) by the integrated Chinese medicine and Western medicine therapy	3819	No medical treatment intervention
JPRN-UMIN000040602	Homeopathy for Covid-19 in primary care	3822	No medical treatment intervention
PACTR202006717277937	Effect of Interferential Therapy on SARS-CoV-2. COVID-19	3824	No medical treatment intervention
ISRCTN99248812	Peer support training for adolescents during the COVID-19 outbreak	3825	No medical treatment intervention
KCT0005105	A Trial of Ciclesonide in Adults with Mild COVID-19	3826	Duplicate
ACTRN12620000688987	Comparing the user seal check and fit test between two types of N95 respirators - the Halyard N95 Particulate Filter Respirators and the ProShield¬Æ N-95 masks	3828	No medical treatment intervention
ISRCTN11188345	A therapeutic study in pre-ICU patients admitted with coronavirus using repurposed drugs	3832	Duplicate
ISRCTN16483855	Inhale work package 3 ,Äì rapid versus standard testing to diagnose lung infections in intensive care, including COVID-19 observational sub-study	3833	No medical treatment intervention
IRCT20180608040007N2	Effect of Auriculotherapy in treatment of Covid 19	3846	No medical treatment intervention
IRCT20131129015584N3	The effect of hot cupping on corona symptoms	3847	No medical treatment intervention
IRCT20200115046144N2	Effects of nokhod-ab on fatigue	3857	No medical treatment intervention
IRCT20200509047360N1	The effect of cognitive-behavioral intervention on improving COVID-19 fear symptoms, improving quality of life and improving mental health in patients with corona	3860	No medical treatment intervention
TCTR20200601002	A Randomized Controlled Trial on Effectiveness of Mental Health & Psychosocial Support Module (MHPSS) in Reduction of Depression, Anxiety and Stress among Healthcare Workers during	3867	No medical treatment intervention
ChiCTR2000033323	Severe Reluctant COVID-19 Patients Responding to Convalescent Plasma; A case series	3870	Cinical, not randomised
NCT04444310	Antibody Responses in Contacts of COVID-19 Patients	3870	Observational
ChiCTR2000033491	Oral Favipiravir for Patients with Delayed SARS-Cov-2 viral RNA Clearance	3871	Cinical, not randomised
NCT04443270	Chloroquine Phosphate Prophylactic Use in Health Personnel Exposed to COVID-19 Patients	3871	Cinical, not randomised
NCT04444115	Loneliness During Strict and Lifted Social Distancing Protocols Against the COVID-19 Pandemic	3872	Observational
NCT04443803	Characterizing the Perioperative Epidemiology of SARS-CoV-2 (COVID-19) Spread for Quality Improvement of Perioperative Infection Control Program	3873	Observational
NCT04444596	Conjunctival Swab vs Nasopharyngeal Swab in Patients With COVID-19	3874	Clinical, not randomised
NCT04444531	Clinical Improvement and COVID-19 Pneumonia	3875	Observational
NCT04427254	Study of the Sars-Cov2 Neuroinvasiveness - COVID19	3876	Clinical, not randomised
NCT04444401	Registry on NEN Patients and COVID-19	3877	Observational
NCT04383548	Clinical Study for Efficacy of Anti-Corona VS2 Immunoglobulins Prepared From COVID19 Convalescent Plasma Prepared by VIPS Mini-Pool IVIG Medical Devices in Prevention of SARS-CoV-	3878	Clinical, not randomised
NCT04416893	Part 2 on the Study Prevalence of SARS -Cov2 Carriage in Asymptomatic and Mildly-symptomatic Children (COVILLE2)	3879	Observational
NCT04385810	Description of Ophthalmologic Injuries in Intensive Care During the SARS-CoV2 Epidemic - COVID19	3880	Clinical, not randomised

Registry entry ID	Title	Database ID (ID all)	Reason for exclusion
NCT04443140	Preeclampsia-like Syndrome Induced by COVID-19: Is it a Real Public Health Issue in Pregnancy During the Pandemic?	3881	Observational
NCT04443712	Screening for Patients Admitted to Ain-Shams University Hospitals for SARS-COV-2 (COVID19)	3882	Observational
NCT04444609	PROSAIC-19 - Prospective Longitudinal Assessment in a COVID-19 Infected Cohort	3883	Observational
NCT04422444	Post Intensive Care Syndrome in COVID19 Patients	3884	Observational
NCT04405726	Prognostic Factors of COVID19	3885	Observational
NCT04401163	Long-term Pulmonary Outcomes After Infection With Sars-CoV-2	3886	Observational
NCT04429620	Prospective Assay for SARS-CoV-2 (COVID-19) Antibody Detection Indirectly by Immunofluorescence: SARS-CoV2 IIF Method	3887	Observational
NCT04434144	A Comparative Study on Ivermectin and Hydroxychloroquine on the COVID19 Patients in Bangladesh	3888	Observational
NCT04442087	Study of Seroprevalence of Anti-SARS-CoV2 Among Children of Hospital Workers in AP-HP	3889	Clinical, not randomised
NCT04443257	REassessement After Hospitalization for Sars-COV-2 disordER	3890	Observational
NCT04414657	USZ BioResource COVID	3891	Observational
NCT04402853	SARS Cov-2 in Conjunctival Secretion	3892	Observational
NCT04414319	Severe Acute Respiratory Syndrome-Coronavirus-2 and Loss of Autonomy in the Elderly	3893	Observational
NCT04422977	Serological Study of the Exposure of Personnel to Sars-cov-2	3894	Clinical, not randomised
NCT04395599	Risk of Air Contamination During Visceral Surgery in COVID19 Patients	3895	Clinical, not randomised
NCT04441372	Prognostication of Oxygen Requirement in Non-severe SARS-CoV-2 Infection	3896	Observational
NCT04410432	Immune Response and Risk of Serious Infection to SARS-Cov2	3897	Observational
NCT04401033	Increased Risk of SARS-CoronaVirus-2 (SARS-CoV-2) Infection Associated With Endoscopy (DECORE Study)	3898	Observational
NCT04402970	Dornase Alfa for ARDS in Patients With SARS-CoV-2	3899	Clinical, not randomised
NCT04443361	Insomnia After the COVID-19 Pandemic	3900	Observational
NCT04443699	Predictors of Mental Well-being During the COVID-19 Pandemic	3901	Observational
NCT04444661	Effects of COVID-19 Induced Deconditioning After Long-term High Intensity Resistance Exercise	3902	Observational
NCT04443764	Status and Predictors of Mental Health Symptoms Among Migrants and Refugees During the COVID-19 Pandemic	3903	Observational
NCT04444336	Information Sources and Their Relationship to Depressive and Anxiety Symptoms During the COVID-19 Pandemic: A Network Study	3904	Observational
NCT04404608	Etiology of Lymphopenia in Covid19 Infection	3905	Observational
NCT04405232	Coagulopathy in COVID19 - A Multi-Centre Observational Study in UK	3906	Observational
NCT04444557	Effect of COvid-19 on Mental Health in Syrian and Turkish Maintenance HemoDialysis Patients: COST-HD Study	3907	Observational
NCT04407156	Epidemiology of Acute Kidney Injury in COVID19 Disease in United Kingdom	3908	Observational
NCT04419870	Acute Infection in Mitochondrial Disease: Metabolism, Infection and Immunity During the COVID19 Pandemic	3909	Observational
NCT04397471	A Study to Collect Bone Marrow for Process Development and Production of BM-MSC to Treat Severe COVID19 Pneumonitis	3910	Observational
NCT04384705	HDL Target of CoViD19 ? Analysis on the Caregivers of the Reunion University Hospital	3911	Observational
NCT04432350	Assessment of Mortality Rates in SARS-CoV-2 Infected Populations Treated With Repurposed Medications	3912	Observational
NCT04441476	Ethical and Psychological Support for Health Care Professions in Intensive Care Units in the COVID19 Pandemic Context: Adequacy With Needs and Psychological Impact Crisis and Post-crisis	3913	Observational
NCT04444687	COVID 19: Is SARS-CoV-2 Contaminating Surgical Aerosols From Laparoscopy and Open Abdominal Surgery?	3914	Observational
NCT04398043	SARS-CoV 2 in Personal Protective Equipment	3915	Observational
NCT04441710	Caregiver Serological Monitoring Extended Secondarily to Patients With the SARS-CoV-2 Coronavirus	3916	Observational
NCT04435522	Maraviroc in SARS-CoV-2	3917	Clinical, not randomised
NCT04442789	Sequelae of Sars-CoV-2 Infections	3918	Observational
NCT04409275	LUNG FIBROTIC CHANGES ASSOCIATED WITH SARS-CoV-2 INFECTION	3919	Observational
NCT04429594	Study of the Incidence of SARS-CoV-2 Infection	3920	Clinical, not randomised
NCT04383483	CoV-ICU Score, Intensive Care Unit, SARS-CoV-2	3921	Observational
NCT04444713	Do Network Centrality Predict Overall Depression Symptom Reduction With Lifting of Social Distancing Protocols?	3922	Observational
NCT04443179	Brain Imaging in Babies Study	3923	Observational
NCT04406558	Psychological Impact of the Health Measures Generated by the COVID19 in Adolescents	3924	Observational
NCT04443075	Alterations of Gut Microbiome in the Frontline Medical Staff Under the Stress	3925	Observational
NCT04387253	Study of Late Fetal Loss in Poitou-Charentes. GYNE-COVID	3926	Clinical, not randomised
NCT04441047	Universal Anti-Viral Vaccine for Healthy Elderly Adults	3927	Clinical, not randomised
NCT04387708	Care in Patients Hospitalized for Anorexia Nervosa at the Time of the Coronavirus-19 Epidemic (QUALICOVID)	3928	Observational
NCT04432038	Healthy Lifestyle or Pro-health Obsession During the Pandemic	3929	Observational
SNCTP000003906	Die Coronavirus-Krankheit 2019 (Covid-19) hat mittlerweile Pandemiestatus erreicht und betrifft Länder überall auf der Welt. Aufgrund der Covid-19 Pandemie stehen die Gesundheitssysteme	3930	Duplicate
SNCTP000003938	Infection COVID-19 grave (virus SARS-Cov-2)	3931	Duplicate
SNCTP000003706	Infezione da COVID-19 grave	3932	Duplicate
SNCTP000003750	Akutes Lungenfunktionsstörung (ARDS) durch eine Infektion mit dem SARS-CoV-2 Virus. (COVID-19 ARDS)	3933	Duplicate
NCT04414826	Preliminary Efficacy of a One-Session Mindfulness Telehealth Intervention for Loneliness	3934	No medical treatment intervention
NCT04392115	The PREPARE for COVID Trial	3935	No medical treatment intervention
NCT04424212	ICBT for Mental Health Problems Related to the Coronavirus Pandemic	3936	No medical treatment intervention
NL8668	Lifestyle of pregnant women during COVID-19 pandemic	3937	No medical treatment intervention
NL8685	COVID-19 in professional soccer players (COPROS study)	3938	No medical treatment intervention
NL8705	Quality of life in patients with suspected COVID-19	3939	No medical treatment intervention
NL8714	The impact of COVID-19 among illiterate people and people with a mild intellectual disabilities: preventative measures, (mental) health and support needs	3940	No medical treatment intervention
RBR-3zdynp	Clinical Characterisation Protocol for Severe Emerging Infections ISARIC/WHO: COVID-19	3941	No medical treatment intervention
researchregistry5627	Experiences from the Epicenter—An Observational Report on Converting a Post Anesthesia Care Unit to a COVID-19 Intensive Care Unit	3942	No medical treatment intervention
researchregistry5628	CORONA VIRUS TRANSMISSION AND CONTROLS CHALLENGE MYSTERY FOR DENTISTS (Systematic review)	3943	No medical treatment intervention
researchregistry5631	Prevalence of Positive COVID-19 among Asymptomatic Health Care Workers who take care of Patients Infected with the Novel Coronavirus	3944	No medical treatment intervention
researchregistry5644	The use of Telemedicine in the Management of Continuous Positive Airway Pressure for the Treatment of Obstructive Sleep Apnoea, A Randomised Controlled Trial	3945	No medical treatment intervention
researchregistry5662	A Personalised Risk Predictor for Covid-19 Outcome: Development and Validation of a Rapid Front Door Triage Tool	3946	No medical treatment intervention
researchregistry5671	Clinical characteristics and effect on outcome in Covid-19 patients during hospital admission at a District General Hospital: a retrospective cohort study	3947	Cinical, not randomised
researchregistry5675	Surgical initial best practice for the coronavirus covid-19 pandemic: A protocol for a rapid systematic review of the literature, rapid collaborative evaluation and consensus process.	3948	No medical treatment intervention
researchregistry5701	Impact of COVID-19 Pandemic on Residency and Fellowship Training Programs in Saudi Arabia: A Nationwide Cross-Sectional Study	3949	No medical treatment intervention
researchregistry5707	Data-driven Insights and Analysis to Better Estimate The Effect of Sugar in COVID-19: A retrospective observational study of outcomes and survival in those with diabetes and COVID-19	3950	No medical treatment intervention
researchregistry5709	The Impact of COVID-19 pandemic on pediatric surgery practice: a cross-sectional study	3951	No medical treatment intervention
researchregistry5716	Comprehensive assessment of immune-mediated response; CoVOD-19 infection, from virus exposure to the infection outcomes regarding different treatment protocols	3952	No medical treatment intervention
researchregistry5720	Impact of the COVID-19 pandemic on surgical practice in the Netherlands: a multicenter retrospective cohort study	3953	No medical treatment intervention
researchregistry5726	Change in the spectrum of orthopedic trauma: Effects of COVID-19 Pandemic in a developing nation during the upsurge	3954	No medical treatment intervention
researchregistry5728	COVID-19 outbreak in Jordan: epidemiological features, clinical characteristics, and laboratory findings	3955	No medical treatment intervention
researchregistry5729	COVID-19 pandemic has significantly influenced the pathway of the management of acute appendicitis. An experience from a tertiary care centre	3956	No medical treatment intervention
NCT04393077	Emotional Freedom Technique (EFT) Effect on Nurses	3957	No medical treatment intervention
NCT04406194	Bioequivalence Study of Favipiravir 200 mg Film Tablet (ATABAY, Turkey) Under Fasting Conditions	3958	Not COVID-19 study
NCT04400682	Bioequivalence Study of Favipiravir 200 mg Film Tablet (Novelfarma, Turkey) Under Fasting Conditions	3959	Not COVID-19 study
NCT04407000	Bioequivalence Study of Favipiravir 200 mg Film Tablet (World Medicine, Turkey) Under Fasting Conditions	3960	Not COVID-19 study
NCT04386876	Bioequivalence Study of Lopinavir/Ritonavir 200/50 mg Film Tablet (World Medicine Ilac, Turkey) Under Fasting Conditions	3961	Not COVID-19 study
ChiCTR2000033699	The mechanism of PECAM-1 in mediating pulmonary vascular endothelial permeability through Wnt/β-catenin signaling pathway in SARS-CoV-2-related ARDS	3962	Cinical, not randomised
ChiCTR2000034076	Novel coronavirus pneumonia (COVID-19) loaded autologous dendritic cell trial	3963	Cinical, not randomised
ACTRN12620000698976	Maintaining the health of people with and without COVID-19 during isolation	3964	No medical treatment intervention
NL8694	Prospective diagnosis of Covid-19 ïnfection using exhaled breath analysis by electronic nose	3965	No medical treatment intervention
RPCEC00000322	Treatment of lung lesions with autologous stem cells in patients recovered from COVID-19 (COVID-19)	3966	No medical treatment intervention
DRKS00021248	Cytokine adsorption in patients with severe COVID-19	3967	No medical treatment intervention
DRKS00022166	Scientific guidance for risk-control and prevention of	3968	No medical treatment intervention
DRKS00022082	Effects of COVID-19 on lung function and gas	3969	No medical treatment intervention
DRKS00021614	Prospective study to evaluate immunity to SARS-CoV2	3970	No medical treatment intervention
DRKS00022188	Influence of the Covid-19 pandemic on the patient mix	3971	No medical treatment intervention
DRKS00022185	Data evaluation of an online symptom and risk	3972	No medical treatment intervention
DRKS00022224	Ambulatory healthcare models and processes during	3973	No medical treatment intervention
UMIN000040816	Relationship between age and viral load in novel coronavirus patients	3974	No medical treatment intervention
NL8645	Serologic surveillance of SARS-CoV-2 during the 2020 pandemic in exposed and unexposed healthcare workers in an academic hospital in Amsterdam	3975	No medical treatment intervention
NL8710	COVID-19 Follow-up care paths and Long-term Outcomes Within the Dutch health care system: a combined rehabilitation, pulmonary, and intensive care perspective	3976	No medical treatment intervention
NCT04386668	Let It Out (LIO) and COVID19: Testing an Online Emotional Disclosure-based Intervention During the COVID19 Pandemic	3977	No medical treatment intervention
eudract002230-32	A MULTISTAGE, PHASE II, STUDY EVALUATING THE SAFETY AND EFFICACY OF COBIMETINIB IN COMBINATION WITH PACLITAXEL AS FIRST-LINE TREATMENT FOR	3994	Cinical, not randomised
eudract002229-27		3996	Duplicate
eudract001517-21	A Randomized, Double-Blind, Placebo-Controlled, Parallel-Group Phase 3 Study of Baricitinib in Patients with COVID-19 Infection	3998	Duplicate
eudract001357-52	Randomized, Controlled, Double-blind Clinical Trial Comparing the Efficacy and Safety of Chemoprophylaxis With Hydroxychloroquine in Patients Under Biological Treatment	3999	Duplicate
eudract001319-26	Multicenter, randomized, controlled, open-label clinical trial to assess the prognostic implications of rosuvastatin treatment in patients discharged after hospitalization for COVID-19 Positive	4000	Not COVID-19 study
eudract001803-17	A Phase 2/3 Single-Arm, Open-Label Study to Evaluate the Safety, Tolerability, Pharmacokinetics, and Efficacy of Remdesivir (GS-5734™) in Participants from Birth to < 18 Years of Age with	4002	Clinical, not randomised
eudract002472-12	Lanadelumab for treatment of COVID-19 disease	4004	Cinical, not randomised
IRCT20150107020592N27	The effect of pentaglobin in the treatment of critically ill patients with COVID-19	4007	Cinical, not randomised
IRCT20150107020592N29	Efficacy of Blood Purification Techniques on Acute Respiratory Distress Syndrome (ARDS) in COVID-19 Pneumonia	4009	Cinical, not randomised
IRCT20150107020592N28	Effectiveness of Borage based solutions in cytokine's levels in covid-19 ARDS patients with elevated Extravascular Lung Water(EVLW)	4013	Cinical, not randomised
IRCT20200508047345N1	The Effectiveness of Combined Extract of 8 Medicinal Plants on the Control of Mild to Moderate Symptoms of Covid-19 Disease: A Clinical Trial	4014	Cinical, not randomised
IRCT20170922036314N4	Evaluating the effects of self-care mobile application on psychologic parameters of admitted patients with Covid-19: a randomized controlled trial	4015	No medical treatment intervention
UMIN000040733	A study on the prevalence of infection in dental institutions by measuring the antibody titer to COVID-19 of the staff members of Kanagawa Dental College Medical Center	4018	No medical treatment intervention
UMIN000040744	Clinical study to evaluate the efficiency of specific antibody against SARS-CoV-2 for the purpose of its social implementation	4019	Cinical, not randomised
JapicCTI-205326	Phase 1 study of FOY-305(FOY-305-02)(COVID-19)	4020	Cinical, not randomised
NL8706	Extracorporeal membrane oxygenation in patients with coronavirus disease 2019	4022	Cinical, not randomised
RPCEC00000323	TRANSFUSION WITH CONVALING PLASMA IMMUNIZES THE ASYMPTOMATIC PATIENT WITH COVID INFECTION 19 THAT FOR MORE THAN 30 DAYS IS POSITIVE SARS	4023	Clinical, not randomised
PACTR202006473370201	Aspirin, losartan and simvastatin in hospitalised COVID-19 patients: a multinational randomised open-label factorial trial	4024	Duplicate
PACTR202006922165132	COVID-19: An Adaptive Phase I/II Randomized Placebo-controlled Trial to Determine Safety, Immunogenicity and Efficacy of Non-replicating ChAdOx1 SARSCoV-2 Vaccine in South African	4025	Duplicate
CTRI/2020/05/025346	A Clinical Trial to Assess the Safety and Efficacy of Convalescent Plasma in Severe Covid-19 patients	4028	No medical treatment intervention
CTRI/2020/05/025299	Convalescent Plasma treatment trial in COVID 19 patients	4030	Duplicate
CTRI/2020/06/025766	A study of 254nm UVC ray therapy in pharynx for asymptomatic to mild cases of Corona Virus Disease	4033	No medical treatment intervention
CTRI/2020/06/026119	Efficacy of Arsenic Album 30C and 200C potency in mild case of COVID-19 positive . (Isolation ward of Sir T General Hospital, Bhavnagar) patients: A randomized controlled study	4035	No medical treatment intervention
