# Supplementary material for: A systematic review of trial registry entries for randomized clinical trials investigating COVID-19 medical prevention and treatment
Source: PLoS One. 2020 Aug 20;15(8):e0237903. doi: 10.1371/journal.pone.0237903 (PMC7444584; doi:10.1371/journal.pone.0237903)
Supplement: S3 Appendix — (PDF) [file pone.0237903.s003.pdf]

Appendix 3: Included trial registry entries

Registry entry ID	Included ID	Intervention	Title	Webpage
NCT04246242	1	Umifenovir	A Randomized Multicenter Controlled Clinical Trial of Arbidol in Patients With 2019 Novel Coronavirus (2019-nCoV)	https://clinicaltrials.gov/show/NCT04246242
NCT04252885	2	Ritonavir/Lopinavir/Umifenovir	The Efficacy of Lopinavir Plus Ritonavir and Arbidol Against Novel Coronavirus Infection	https://clinicaltrials.gov/show/NCT04252885
NCT04251871	3	TCM	Treatment and Prevention of Traditional Chinese Medicines (TCMs) on 2019-nCoV Infection	https://clinicaltrials.gov/show/NCT04251871
NCT04260594	4	Umifenovir	Clinical Study of Arbidol Hydrochloride Tablets in the Treatment of Pneumonia Caused by Novel Coronavirus	https://clinicaltrials.gov/show/NCT04260594
NCT04261426	5	Immunoglobulin	The Efficacy of Intravenous Immunoglobulin Therapy for Severe 2019-nCoV Infected Pneumonia	https://clinicaltrials.gov/show/NCT04261426
NCT04261907	6	Ritonavir/Lopinavir/ASC09	Evaluating and Comparing the Safety and Efficiency of ASC09/Ritonavir and Lopinavir/Ritonavir for Novel Coronavirus Infection	https://clinicaltrials.gov/show/NCT04261907
NCT04244591	7	Glucocorticoid	Glucocorticoid Therapy for Novel CoronavirusCritically Ill Patients With Severe Acute Respiratory Failure	https://clinicaltrials.gov/show/NCT04244591
NCT04268537	8	PD-1 blocking antibody/Thymosin	Immunoregulatory Therapy for 2019-nCoV	https://clinicaltrials.gov/show/NCT04268537
NCT04273529	9	Thalidomide	The Efficacy and Safety of Thalidomide in the Adjuvant Treatment of Moderate New Coronavirus (COVID-19) Pneumonia	https://clinicaltrials.gov/show/NCT04273529
NCT04273581	10	Thalidomide	The Efficacy and Safety of Thalidomide Combined With Low-dose Hormones in the Treatment of Severe COVID-19	https://clinicaltrials.gov/show/NCT04273581
NCT04273646	11	Stem cells	Study of Human Umbilical Cord Mesenchymal Stem Cells in the Treatment of Novel Coronavirus Severe Pneumonia	https://clinicaltrials.gov/show/NCT04273646
NCT04275388	12	TCM	Xiyanping Injection for the Treatment of New Coronavirus Infected Pneumonia	https://clinicaltrials.gov/show/NCT04275388
NCT04279197	13	TCM	Treatment of Pulmonary Fibrosis Due to 2019-nCoV Pneumonia With Fuzheng Huayu	https://clinicaltrials.gov/show/NCT04279197
NCT04280224	14	NK cells	NK Cells Treatment for Novel Coronavirus Pneumonia	https://clinicaltrials.gov/show/NCT04280224
NCT04276688	15	Ritonavir/Lopinavir/Interferon Beta-1B/Umifenovir	Lopinavir/ Ritonavir, Ribavirin and IFN-beta Combination for nCoV Treatment	https://clinicaltrials.gov/show/NCT04276688
NCT04282902	16	Pirfenidone	A Study to Evaluate the Efficacy and Safety of Pirfenidone With Novel Coronavirus Infection	https://clinicaltrials.gov/show/NCT04282902
NCT04286503	17	Claritromycin/lopinavir/Umifenovir	The Clinical Study of Carrimycin on Treatment Patients With COVID-19	https://clinicaltrials.gov/show/NCT04286503
NCT04295551	18	TCM	Multicenter Clinical Study on the Efficacy and Safety of Xiyanping Injection in the Treatment of New Coronavirus Infection Pneumonia (General and Severe)	https://clinicaltrials.gov/show/NCT04295551
NCT04293887	19	INF-Beta	Efficacy and Safety of IFN-a2ß in the Treatment of Novel Coronavirus Patients	https://clinicaltrials.gov/show/NCT04293887
NCT04252274	20	Darunavir/Cobicistat	Efficacy and Safety of Darunavir and Cobicistat for Treatment of Pneumonia Caused by 2019-nCoV	https://clinicaltrials.gov/show/NCT04252274
NCT04264533	21	Vitamin C	Vitamin C Infusion for the Treatment of Severe 2019-nCoV Infected Pneumonia	https://clinicaltrials.gov/show/NCT04264533
IRCT20100228003449N28	22	INF-Beta	Interferon in treatment of COVID-19	http://en.irct.ir/trial/46538
IRCT20200316046792N1	23	TCM	Effect of herbal syrup and solution in treatment of COVID-19	http://en.irct.ir/trial/46554
JPRN-jRCTs041190120	24	Favipiravir	Favipiravir for SARS-CoV-infected patients	https://jrct.niph.go.jp/latest-detail/jRCTs041190120
IRCT20100228003449N29	25	Sofosbuvir/ledipasvir	Effect of sofosbuvir/ ledipasvir on COVID-19	http://en.irct.ir/trial/46567
IRCT20200128046294N2	26	Sofosbuvir/Daclatasvir	Study to Evaluate the Safety and Efficacy of Sofosbuvir/Daclatasvir in Participants with Moderate to Severe Coronavirus Disease (COVID-19)	http://en.irct.ir/trial/46463
JPRN-jRCTs031190227	27	Oseltamivir	Triple combination therapy in patients infected with COVID-19	https://jrct.niph.go.jp/latest-detail/jRCTs031190227
NCT04254874	28	INF atomization	A Prospective/Retrospective,Randomized Controlled Clinical Study of Interferon Atomization in the 2019-nCoV Pneumonia	https://clinicaltrials.gov/show/NCT04254874
NCT04255017	29	Ritonavir/Lopinavir/Umifenovir/Oseltamivir	A Prospective/Retrospective,Randomized Controlled Clinical Study of Antiviral Therapy in the 2019-nCoV Pneumonia	https://clinicaltrials.gov/show/NCT04255017
NCT04263402	30	Glucocorticoid	The Efficacy of Different Hormone Doses in 2019-nCoV Severe Pneumonia	https://clinicaltrials.gov/show/NCT04263402
NCT04273763	31	Bromhexine	Evaluating the Efficacy and Safety of Bromhexine Hydrochloride Tablets Combined With Standard Treatment/ Standard Treatment in Patients With Suspected and Mild Novel Coronavirus Pneumonia (COVID-19)	https://clinicaltrials.gov/show/NCT04273763
NCT04288102	32	Stem cells	Treatment With Mesenchymal Stem Cells for Severe Corona Virus Disease 2019(COVID-19)	https://clinicaltrials.gov/show/NCT04288102
NCT04291053	33	TCM	The Efficacy and Safety of Huai er in the Adjuvant Treatment of COVID-19	https://clinicaltrials.gov/show/NCT04291053
NCT04261270	34	Ritonavir/ASC09F	A Randomized,Open,Controlled Clinical Study to Evaluate the Efficacy of ASC09F and Ritonavir for 2019-nCoV Pneumonia	https://clinicaltrials.gov/show/NCT04261270
NCT04308317	35	Tetrandrine	Tetrandrine Tablets Used in the Treatment of COVID-19	https://clinicaltrials.gov/show/NCT04308317
NCT04310228	36	Favipiravir/Tocilizumab	Favipiravir Combined With Tocilizumab in the Treatment of Corona Virus Disease 2019	https://clinicaltrials.gov/show/NCT04310228
NCT04318015	37	Chloroquine	Hydroxychloroquine Chemoprophylaxis in Healthcare Personnel in Contact With COVID-19 Patients (PHYDRA Trial)	https://clinicaltrials.gov/show/NCT04318015
NCT04315896	38	Chloroquine	Hydroxychloroquine Treatment for Severe COVID-19 Pulmonary Infection (HYDRA Trial)	https://clinicaltrials.gov/show/NCT04315896
NCT04317040	39	CD24Fc	CD24Fc as a Non-antiviral Immunomodulator in COVID-19 Treatment	https://clinicaltrials.gov/show/NCT04317040
NCT04292899	40	Remdesivir	Study to Evaluate the Safety and Antiviral Activity of Remdesivir (GS-5734™) in Participants With Severe Coronavirus Disease (COVID-19)	https://clinicaltrials.gov/show/NCT04292899
NCT04303507	41	Chloroquine	Chloroquine/ Hydroxychloroquine Prevention of Coronavirus Disease (COVID-19) in the Healthcare Setting	https://clinicaltrials.gov/show/NCT04303507
NCT04306393	42	Nitric Oxide	Nitric Oxide Gas Inhalation in Severe Acute Respiratory Syndrome in COVID-19	https://clinicaltrials.gov/show/NCT04306393
NCT04298060	43	DAS181	DAS181 for Patients With Severe Hospitalized Flu and SAD-RVs (COVID-19)	https://clinicaltrials.gov/show/NCT04298060
NCT04304053	44	Chloroquine + other	Treatment of COVID-19 Cases and Chemoprophylaxis of Contacts as Prevention	https://clinicaltrials.gov/show/NCT04304053
NCT04308668	45	Chloroquine	Post-exposure Prophylaxis / Preemptive Therapy for SARS-Coronavirus-2	https://clinicaltrials.gov/show/NCT04308668
NCT04280705	46	Remdesivir	Adaptive COVID-19 Treatment Trial (ACTT)	https://clinicaltrials.gov/show/NCT04280705
NCT04305457	47	Nitric Oxide	Nitric Oxide Gas Inhalation Therapy for Mild/Moderate COVID-19	https://clinicaltrials.gov/show/NCT04305457
NCT04312243	48	Nitric Oxide	NO Prevention of COVID-19 for Healthcare Providers	https://clinicaltrials.gov/show/NCT04312243
NCT04313023	49	PUL-042	The Use PUL-042 Inhalation Solution to Prevent COVID-19 in Adults Exposed to SARS-CoV-2	https://clinicaltrials.gov/show/NCT04313023
NCT04315298	50	Sarilumab	Evaluation of the Efficacy and Safety of Sarilumab in Hospitalized Patients With COVID-19	https://clinicaltrials.gov/show/NCT04315298
NCT04311697	51	Aviptadil	Intravenous Aviptadil for COVID-19 Associated Acute Respiratory Distress	https://clinicaltrials.gov/show/NCT04311697
NCT04312009	52	Losartan	Losartan for Patients With COVID-19 Requiring Hospitalization	https://clinicaltrials.gov/show/NCT04312009
NCT04316377	53	Chloroquine	Norwegian Coronavirus Disease 2019 Study	https://clinicaltrials.gov/show/NCT04316377
NCT04318444	54	Chloroquine	Hydroxychloroquine Post Exposure Prophylaxis for Coronavirus Disease (COVID-19)	https://clinicaltrials.gov/show/NCT04318444
NCT04311177	55	Losartan	Losartan for Patients With COVID-19 Not Requiring Hospitalization	https://clinicaltrials.gov/show/NCT04311177
NCT04312997	56	PUL-042	The Use of PUL-042 Inhalation Solution to Reduce the Severity of COVID-19 in Adults Positive for SARS-CoV-2 Infection	https://clinicaltrials.gov/show/NCT04312997
NCT04315948	57	Chloroquine + other	Trial of Treatments for COVID-19 in Hospitalized Adults	https://clinicaltrials.gov/show/NCT04315948
NCT04320056	58	Automated oxygen administration	Closed-Loop Oxygen to Verify That Healthcare Workers Interventions Decrease During Pneumonia	https://clinicaltrials.gov/show/NCT04320056
NCT04319900	59	Chloroquine	Clinical Trial of Favipiravir Tablets Combine With Chloroquine Phosphate in the Treatment of Novel Coronavirus Pneumonia	https://clinicaltrials.gov/show/NCT04319900
NCT04322123	60	Chloroquine / Azithromycin	Safety and Efficacy of Hydroxychloroquine Associated With Azythromycin in SARS-Cov-2 Virus	https://clinicaltrials.gov/show/NCT04322123
NCT04324073	61	Sarilumab	Cohort Multiple Randomized Controlled Trials Open-label of Immune Modulatory Drugs and Other Treatments in COVID-19 Patients - Sarilumab Trial - CORIMUNO-19 - SARI	https://clinicaltrials.gov/show/NCT04324073
NCT04321174	62	Ritonavir/Lopinavir	COVID-19 Ring-based Prevention Trial With Lopinavir/Ritonavir	https://clinicaltrials.gov/show/NCT04321174
NCT04322565	63	Colchicine	Colchicine Efficacy in COVID-19 Pneumonia	https://clinicaltrials.gov/show/NCT04322565
NCT04322773	64	Tocilizumab/Sarilumab	Anti-il6 Treatment of Serious COVID-19 Disease With Threatening Respiratory Failure	https://clinicaltrials.gov/show/NCT04322773
NCT04261517	65	Chloroquine	Efficacy and Safety of Hydroxychloroquine for Treatment of Pneumonia Caused by 2019-nCoV (HC-nCoV)	https://clinicaltrials.gov/show/NCT04261517
NCT04292730	66	Remdesivir	Study to Evaluate the Safety and Antiviral Activity of Remdesivir (GS-5734™) in Participants With Moderate Coronavirus Disease (COVID-19) Compared to Standard of Care Treatment	https://clinicaltrials.gov/show/NCT04292730
NCT04324606	67	Vaccine COV001	A Study of a Candidate COVID-19 Vaccine (COV001)	https://clinicaltrials.gov/show/NCT04324606
NCT04325906	68	Prone position	Early PP With HFNC Versus HFNC in COVID-19 Induced Moderate to Severe ARDS	https://clinicaltrials.gov/show/NCT04325906
NCT04321616	69	Chloroquine + other	The Efficacy of Different Anti-viral Drugs in (Severe Acute Respiratory Syndrome-Corona Virus-2) SARS-CoV-2	https://clinicaltrials.gov/show/NCT04321616
NCT04323631	70	Chloroquine	Hydroxychloroquine for the Treatment of Patients With Mild to Moderate COVID-19 to Prevent Progression to Severe Infection or Death	https://clinicaltrials.gov/show/NCT04323631
NCT04324528	71	Cytokine adsorption	Cytokine Adsorption in Severe COVID-19 Pneumonia Requiring Extracorporeal Membrane Oxygenation	https://clinicaltrials.gov/show/NCT04324528
NCT04305106	72	Bevacizumab	Bevacizumab in Severe or Critically Severe Patients With COVID-19 Pneumonia-RCT	https://clinicaltrials.gov/show/NCT04305106
NCT04320615	73	Tocilizumab	A Study to Evaluate the Safety and Efficacy of Tocilizumab in Patients With Severe COVID-19 Pneumonia	https://clinicaltrials.gov/show/NCT04320615
NCT04326790	74	Colchicine	The GReek Study in the Effects of Colchicine in Covid-19 cOmplications Prevention	https://clinicaltrials.gov/show/NCT04326790
NCT04327206	75	BCG vaccine	BCG Vaccination to Protect Healthcare Workers Against COVID-19	https://clinicaltrials.gov/show/NCT04327206
NCT04322682	76	Colchicine	Colchicine Coronavirus SARS-CoV2 Trial (COLCORONA)	https://clinicaltrials.gov/show/NCT04322682
NCT04323345	77	TCM	Efficacy of Natural Honey Treatment in Patients With Novel Coronavirus	https://clinicaltrials.gov/show/NCT04323345
NCT04321096	78	Camostat Mesilate	The Impact of Camostat Mesilate on COVID-19 Infection	https://clinicaltrials.gov/show/NCT04321096
NCT04321278	79	Chloroquine / Azithromycin	Safety and Efficacy of Hydroxychloroquine Associated With Azithromycin in SARS-CoV2 Virus (Coalition Covid-19 Brasil II)	https://clinicaltrials.gov/show/NCT04321278
NCT04328441	80	BCG Vaccine	Reducing Health Care Workers Absenteeism in Covid-19 Pandemic Through BCG Vaccine	https://clinicaltrials.gov/show/NCT04328441
NCT04325061	81	Glucocorticoid	Efficacy of Glucocorticoide Treatment for Patients With ARDS Caused by COVID-19	https://clinicaltrials.gov/show/NCT04325061
NCT04325893	82	Chloroquine	Hydroxychloroquine Versus Placebo in Patients Presenting COVID-19 Infection and at Risk of Secondary Complication: a Prospective, Multicentre, Randomised, Double-blind Study	https://clinicaltrials.gov/show/NCT04325893
NCT04323800	83	Convalescent Plasma	Efficacy and Safety Human Coronavirus Immune Plasma (HCIP) vs. Control (SARS-CoV-2 Non-immune Plasma) Among Adults Exposed to COVID-19	https://clinicaltrials.gov/show/NCT04323800
NCT04324021	84	Emapalumab/Anakinra	Efficacy and Safety of Emapalumab and Anakinra in Reducing Hyperinflammation and Respiratory Distress in Patients With COVID-19 Infection.	https://clinicaltrials.gov/show/NCT04324021
NCT04322396	85	Chloroquine / Azithromycin	Proactive Prophylaxis With Azithromycin and Chloroquine in Hospitalized Patients With COVID-19	https://clinicaltrials.gov/show/NCT04322396
NCT04323228	86	Oral nutrition supplement (ONS) enriched in eicosapentaenoic acid, gamma-linolenic acid and antioxidants	Anti-inflammatory/Antioxidant Oral Nutrition Supplementation in COVID-19	https://clinicaltrials.gov/show/NCT04323228
NCT04330144	87	Chloroquine	Hydroxychloroquine as Post Exposure Prophylaxis for SARS-CoV-2(HOPE Trial)	https://clinicaltrials.gov/show/NCT04330144
NCT04330495	88	Chloroquine	Randomized, Controlled, Double-blind Clinical Trial Comparing the Efficacy and Safety of Chemoprophylaxis With Hydroxychloroquine in Patients Under Biological Treatment and / or JAK Inhibitors in the Prevention of SARS-CoV-2 Infection	https://clinicaltrials.gov/show/NCT04330495

Registry entry ID	Included ID	Intervention	Title	Webpage
NCT04326920	89	Sargramostim	Sargramostim in Patients With Acute Hypoxic Respiratory Failure Due to COVID-19 (SARPAC)	https://clinicaltrials.gov/show/NCT04326920
NCT04328285	90	Chloroquine + other	Chemoprophylaxis of SARS-CoV-2 Infection (COVID-19) in Exposed Healthcare Workers	https://clinicaltrials.gov/show/NCT04328285
NCT04324463	91	Chloroquine / Azithromycin	Anti-Coronavirus Therapies to Prevent Progression of Coronavirus Disease 2019 (COVID-19) Trial	https://clinicaltrials.gov/show/NCT04324463
NCT04326426	92	Tradipitant	COVIDL1: A Study to Investigate the Efficacy of Tradipitant in Treating Severe or Critical COVID-19 Infection	https://clinicaltrials.gov/show/NCT04326426
NCT04324996	93	NK cells	A Phase I/II Study of Universal Off-the-shelf NKG2D-ACE2 CAR-NK Cells for Therapy of COVID-19	https://clinicaltrials.gov/show/NCT04324996
NCT04325633	94	Naproxen	Efficacy of Addition of Naproxen in the Treatment of Critically Ill Patients Hospitalized for COVID-19 Infection	https://clinicaltrials.gov/show/NCT04325633
NCT04330586	95	Chloroquine + other	A Trial of Ciclesonide in Adults With Mild COVID-19	https://clinicaltrials.gov/show/NCT04330586
NCT04331054	96	Budesonide / Formoterol	Protective Role of Inhaled Steroids for Covid-19 Infection	https://clinicaltrials.gov/show/NCT04331054
NCT04331470	97	Levamisole/Budesonide/Formoterol	Evaluation of Efficacy of Levamisole and Formoterol+Budesonide in Treatment of COVID-19	https://clinicaltrials.gov/show/NCT04331470
NCT04328961	98	Chloroquine	Hydroxychloroquine for COVID-19 PEP	https://clinicaltrials.gov/show/NCT04328961
NCT04329650	99	Siltuximab/Glucocorticoid	Efficacy and Safety of Siltuximab vs. Corticosteroids in Hospitalized Patients With COVID19 Pneumonia	https://clinicaltrials.gov/show/NCT04329650
NCT04326075	100	CPAP	Early CPAP in COVID Patients With Respiratory Failure.	https://clinicaltrials.gov/show/NCT04326075
NCT04327505	101	Hyperbaric oxygen	Safety and Efficacy of Hyperbaric Oxygen for ARDS in Patients With COVID-19	https://clinicaltrials.gov/show/NCT04327505
NCT04329923	102	Chloroquine	The PATCH Trial (Prevention And Treatment of COVID-19 With Hydroxychloroquine)	https://clinicaltrials.gov/show/NCT04329923
NCT04333420	103	FX-1	OPEN LABEL, RANDOMIZED PHASE II/III STUDY OF IFX-1 IN PATIENTS WITH SEVERE COVID-19 PNEUMONIA	https://clinicaltrials.gov/show/NCT04333420
NCT04333628	104	Chloroquine	Chloroquine for Mild Symptomatic and Asymptomatic COVID-19	https://clinicaltrials.gov/show/NCT04333628
NCT04329832	105	Chloroquine / Azithromycin	Hydroxychloroquine vs. Azithromycin for Hospitalized Patients With Suspected or Confirmed COVID-19	https://clinicaltrials.gov/show/NCT04329832
NCT04330300	106	Thiazide/Calcium Channel Blockers/ACE inhibitor/Angiotensin receptor blocker	Coronavirus ACEi/ARB Investigation	https://clinicaltrials.gov/show/NCT04330300
NCT04330690	107	Ritonavir/Lopinavir	Treatments for COVID-19: Canadian Arm of the SOLIDARITY Trial	https://clinicaltrials.gov/show/NCT04330690
NCT04327388	108	Sarilumab	Sarilumab COVID-19	https://clinicaltrials.gov/show/NCT04327388
NCT04333654	109	Chloroquine	Hydroxychloroquine in Outpatient Adults With COVID-19	https://clinicaltrials.gov/show/NCT04333654
ISRCTN50189673	110	Chloroquine + other	A randomised trial of treatments to prevent death in patients hospitalised with COVID-19 (coronavirus)	http://isrctn.com/ISRCTN50189673
NCT04331834	111	Chloroquine	Pre-Exposure Prophylaxis With Hydroxychloroquine for High-Risk Healthcare Workers During the COVID-19 Pandemic	https://clinicaltrials.gov/show/NCT04331834
NCT04327401	112	Glucocorticoid	COVID-19-associated ARDS Treated With Glucocorticoide: Alliance Covid-19 Brasil III	https://clinicaltrials.gov/show/NCT04327401
NCT04328012	113	Chloroquine + other	Comparison Of Therapeutics for Hospitalized Patients Infected With SARS-CoV-2 In a Pragmatic aDaptive randoMizED Clinical Trial During the COVID-19 Pandemic (COVID MED Trial)	https://clinicaltrials.gov/show/NCT04328012
NCT04328272	114	Chloroquine / Azithromycin	Effectiveness of Hydroxychloroquine in Covid-19 Patients	https://clinicaltrials.gov/show/NCT04328272
NCT04331600	115	Chloroquine	ChloroQUine As antiviRal treAtmeNT In coroNavirus infEction 2020	https://clinicaltrials.gov/show/NCT04331600
NCT04332094	116	Chloroquine + other	Clinical Trial of Combined Use of Hydroxychloroquine, Azithromycin, and Tocilizumab for the Treatment of COVID-19	https://clinicaltrials.gov/show/NCT04332094
NCT04333589	117	Favipiravir	A Prospective Study on Corona Virus Disease 2019 Patients Whose Nucleic Acids Changed From Negative to Positive	https://clinicaltrials.gov/show/NCT04333589
NCT04328467	118	Chloroquine	Pre-exposure Prophylaxis for SARS-Coronavirus-2	https://clinicaltrials.gov/show/NCT04328467
NCT04328480	119	Colchicine	The ECLA PHRI COLCOVID Trial	https://clinicaltrials.gov/show/NCT04328480
NCT04328493	120	Chloroquine	The Vietnam Chloroquine Treatment on COVID-19	https://clinicaltrials.gov/show/NCT04328493
NCT04331808	121	Tocilizumab	Cohort Multiple Randomized Controlled Trials Open-label of Immune Modulatory Drugs and Other Treatments in COVID-19 Patients - Tocilizumab Trial - CORIMUNO-19 - TOCI (CORIMUNO-TOCI)	https://clinicaltrials.gov/show/NCT04331808
NCT04331899	122	Peginterferon Lambda-1a	Mild COVID-19 Peginterferon Lambda	https://clinicaltrials.gov/show/NCT04331899
NCT04332107	123	Azithromycin	Azithromycin for COVID-19 Treatment in Outpatients Nationwide	https://clinicaltrials.gov/show/NCT04332107
NCT04334005	124	Vitamin D	Vitamin D on Prevention and Treatment of COVID-19	https://clinicaltrials.gov/show/NCT04334005
NCT04332666	125	Angiotensin 1-7	Angiotensin-(1,7) Treatment in COVID-19: the ATCO Trial	https://clinicaltrials.gov/show/NCT04332666
NCT04332991	126	Chloroquine	Outcomes Related to COVID-19 Treated With Hydroxychloroquine Among In-patients With Symptomatic Disease	https://clinicaltrials.gov/show/NCT04332991
NCT04330638	127	Anakinra/Siltuximab/Tocilizumab	Treatment of COVID-19 Patients With Anti-interleukin Drugs	https://clinicaltrials.gov/show/NCT04330638
NCT04332835	128	Convalescent Plasma	Convalescent Plasma for Patients With COVID-19: A Randomized, Open Label, Parallel, Controlled Clinical Study	https://clinicaltrials.gov/show/NCT04332835
NCT04333251	129	Convalescent Plasma	Study Testing Convalescent Plasma vs Best Supportive Care	https://clinicaltrials.gov/show/NCT04333251
NCT04333407	130	Aspirin/Clopidogrel/Rivaroxaban/Atorvastatin/Omeprazole	Preventing Cardiac Complication of COVID-19 Disease With Early Acute Coronary Syndrome Therapy: A Randomised Controlled Trial.	https://clinicaltrials.gov/show/NCT04333407
NCT04333550	131	Deferoxamine	Application of Desferal to Treat COVID-19	https://clinicaltrials.gov/show/NCT04333550
NCT04333732	132	Chloroquine	CROWN CORONATION: Chloroquine RepurpOsing to healthWorkers for Novel CORONAvirus mitigaTION	https://clinicaltrials.gov/show/NCT04333732
ISRCTN16912075	133	CPAP/high-flow nasal oxygen	RECOVERY Respiratory Support: Respiratory Strategies in patients with coronavirus COVID-19 – CPAP, high-flow nasal oxygen, and standard care	http://isrctn.com/ISRCTN16912075
ISRCTN83971151	134	Chloroquine + other	Public health emergency SOLIDARITY trial of treatments for COVID-19 infection in hospitalized patients	http://isrctn.com/ISRCTN83971151
NCT04333472	135	Piclidenoson	Piclidenoson for Treatment of COVID-19	https://clinicaltrials.gov/show/NCT04333472
NCT04333914	136	Chloroquine + other	Prospective Study in Patients With Advanced or Metastatic Cancer and SARS-CoV-2 (COVID-19) Infection	https://clinicaltrials.gov/show/NCT04333914
ACTRN12620000445976	137	Chloroquine + other	To compare the effectiveness of two drugs (hydroxychloroquine and lopinavir/ritonavir alone or combined in treating hospitalized patients with confirmed COVID-19 compared to standard of care	https://anzctr.org.au/ACTRN12620000445976.aspx
ACTRN12620000417987	138	Chloroquine	Chloroquine Chemorophylaxis Countermeasure against COVID-19	https://anzctr.org.au/ACTRN12620000417987.aspx
EUCTR2020-001243-15-BE	139	Itraconazole	Covid-19: A randomized, open-label, adaptive, proof-of- concept clinical trial of new antiviral drug candidates against SARS-CoV-2.	https://www.clinicaltrialsregister.eu/ctr-search/search?query=eudract_number:2020-001243-15
EUCTR2020-000919-69-NL	140	BCG vaccine	Reducing health care workers absenteeism in Coronavirus 2019 pandemic nu enhancement of the immune system through vaccination with Bacillus Calmette-Guérin	https://www.clinicaltrialsregister.eu/ctr-search/search?query=eudract_number:2020-000919-69
EUCTR2020-000982-18-NO	141	Chloroquine	A clinical study to evaluate the efficacy of different anti-viral drugs in SARS-CoV-2 infected patients (COVID-19)	https://www.clinicaltrialsregister.eu/ctr-search/search?query=eudract_number:2020-000982-18
EUCTR2020-001023-14-GB	142	INF-Beta	a trial of an inhaled antiviral drug to treat or prevent severe respiratory difficulties in patients with COVID-19	https://www.clinicaltrialsregister.eu/ctr-search/search?query=eudract_number:2020-001023-14
EUCTR2020-001236-10-NL	143	Imatinib	Countering Lung Damage in COVID-19 infection: the CounterCovid Study	https://www.clinicaltrialsregister.eu/ctr-search/search?query=eudract_number:2020-001236-10
ChiCTR2000029941	144	TCM	A randomized controlled trial for Traditional Chinese Medicine in the treatment for Novel Coronavirus Pneumonia (COVID-19)	http://www.chictr.org.cn/showproj.aspx?proj=49596
ChiCTR2000029939	145	Chloroquine	A Single-blind, Randomized, Controlled Clinical Trial for Chloroquine Phosphate in the treatment of Novel Coronavirus Pneumonia 2019 (COVID-19)	http://www.chictr.org.cn/showproj.aspx?proj=49612
ChiCTR2000029947	146	TCM	A Randomized Controlled Trial for Qingyi No. 4 Compound in the treatment of Convalescence Patients of Novel Coronavirus Pneumonia (COVID-19)	http://www.chictr.org.cn/showproj.aspx?proj=49599
ChiCTR2000029869	147	TCM	A multicenter, randomized, controlled trial for integrated Chinese and western medicine in the treatment of ordinary novel coronavirus pneumonia (COVID-19) based on the ' Internal and External Relieving -Truncated Torsion' strategy	http://www.chictr.org.cn/showproj.aspx?proj=49486
ChiCTR2000029811	148	TCM	Clinical Study for Anti-aging Active Freeze-dried Powder Granules in the Treatment of Acute Novel Coronavirus Pneumonia (COVID-19)	http://www.chictr.org.cn/showproj.aspx?proj=49355
ChiCTR2000029867	149	Ritonavir/Lopinavir/Carrimycin	The efficacy and safety of carrimycin treatment in patients with novel coronavirus pneumonia (COVID-19): a multicenter, randomized, open-label, controlled trial	http://www.chictr.org.cn/showproj.aspx?proj=49514
ChiCTR2000029849	150	Regulating intestinal flora	Application of Regulating Intestinal Flora in the Treatment of Severe Novel Coronavirus Pneumonia (COVID-19)	http://www.chictr.org.cn/showproj.aspx?proj=49530
ChiCTR2000029855	151	TCM	A randomized, open and controlled clinical trial for traditional Chinese medicine in the treatment of novel coronavirus pneumonia (COVID-19)	http://www.chictr.org.cn/showproj.aspx?proj=49543
ChiCTR2000029789	152	TCM	Randomized controlled trial for TCM syndrome differentiation treatment impacting quality of life of post-discharge patients with novel coronavirus pneumonia (COVID-19)	http://www.chictr.org.cn/showproj.aspx?proj=49348
ChiCTR2000029822	153	TCM	A randomized controlled trial for honeysuckle decoction in the treatment of patients with novel coronavirus (COVID-19) infection	http://www.chictr.org.cn/showproj.aspx?proj=49502
ChiCTR2000029853	154	Azvudine	A randomized, open-label, controlled clinical trial for azvudine in the treatment of novel coronavirus pneumonia (COVID-19)	http://www.chictr.org.cn/showproj.aspx?proj=49532
ChiCTR2000029813	155	TCM	Clinical Trial for Tanreqing Capsules in the Treatment of Novel Coronavirus Pneumonia (COVID-19)	http://www.chictr.org.cn/showproj.aspx?proj=49425
ChiCTR2000029806	156	Thymosin/Camrelizumab	Immunomodulatory Therapy for Severe Novel Coronavirus Pneumonia (COVID-19)	http://www.chictr.org.cn/showproj.aspx?proj=49161
ChiCTR2000029777	157	TCM	A multicenter, randomized, controlled trial for integrated chinese and western medicine in the treatment of novel coronavirus pneumonia (COVID-19) based on the 'Truncated Torsion' strategy	http://www.chictr.org.cn/showproj.aspx?proj=49380
ChiCTR2000029776	158	Polyinosinic-Polycytidylic Acid	A randomized, open-label, blank-controlled, multicenter trial for Polyinosinic-Polycytidylic Acid Injection in the treatment of novel coronavirus pneumonia (COVID-19)	http://www.chictr.org.cn/showproj.aspx?proj=49342
ChiCTR2000029790	159	TCM	Clinical study for the integration of traditional Chinese and western medicine in the treatment of novel coronavirus pneumonia (COVID-19)	http://www.chictr.org.cn/showproj.aspx?proj=49453
ChiCTR2000029788	160	TCM	Traditional Chinese medicine cooperative therapy for patients with novel coronavirus pneumonia (COVID-19): a randomized controlled trial	http://www.chictr.org.cn/showproj.aspx?proj=49452
ChiCTR2000029769	161	TCM	Babaodan Capsule used for the adjuvant treatment of severe novel coronavirus pneumonia (COVID-19)	http://www.chictr.org.cn/showproj.aspx?proj=49415
ChiCTR2000029765	162	Tocilizumab	A multicenter, randomized controlled trial for the efficacy and safety of tocilizumab in the treatment of new coronavirus pneumonia (COVID-19)	http://www.chictr.org.cn/showproj.aspx?proj=49409
ChiCTR2000029763	163	TCM	The efficacy of traditional chinese medicine on Novel Coronavirus Pneumonia (COVID-19) patients treated in square cabin hospital: a prospective, randomized controlled trial	http://www.chictr.org.cn/showproj.aspx?proj=49408
ChiCTR2000029656	164	Glucocorticoid	A randomized, open-label study to evaluate the efficacy and safety of low-dose corticosteroids in hospitalized patients with novel coronavirus pneumonia (COVID-19)	http://www.chictr.org.cn/showproj.aspx?proj=49086
ChiCTR2000029768	165	TCM	A randomized, open, controlled trial for diammonium glycyrrhizinate enteric-coated capsules combined with vitamin C tablets in the treatment of common novel coronavirus pneumonia (COVID-19) in the basic of clinical standard antiviral treatment to evaluate the safety and efficiency	http://www.chictr.org.cn/showproj.aspx?proj=49131
ChiCTR2000029756	166	TCM	Clinical study of nebulized Xiyanping injection in the treatment of novel coronavirus pneumonia (COVID-19)	http://www.chictr.org.cn/showproj.aspx?proj=49222
ChiCTR2000029742	167	TCM	A randomized, parallel controlled trial for the efficacy and safety of Sodium Aescinate Injection in the treatment of patients with pneumonia (COVID-19)	http://www.chictr.org.cn/showproj.aspx?proj=49297
ChiCTR2000029741	168	Chloroquine + other	Efficacy of Chloroquine and Lopinavir/ Ritonavir in mild/general novel coronavirus (CoVID-19) infections: a prospective, open-label, multicenter randomized controlled clinical study	http://www.chictr.org.cn/showproj.aspx?proj=49263
ChiCTR2000029747	169	TCM	Effect evaluation and prognosis of Chinese medicine based on Novel Coronavirus Pneumonia (COVID-19)	http://www.chictr.org.cn/showproj.aspx?proj=49287
ChiCTR2000029739	170	Hydrogen-Oxygen Nebulizer/Oxygen concentrator	A Multicenter, Randomized, Parallel Controlled Clinical Study of Hydrogen-Oxygen Nebulizer to Improve the Symptoms of Patients With Novel Coronavirus Pneumonia (COVID-19)	http://www.chictr.org.cn/showproj.aspx?proj=49283
ChiCTR2000029601	171	TCM	Community based prevention and control for Chinese medicine in the treatment of novel coronavirus pneumonia (COVID-19) in the isolate suspected and confirmed population	http://www.chictr.org.cn/showproj.aspx?proj=48988
ChiCTR2000029569	172	Stem Cells	Safety and efficacy of umbilical cord blood mononuclear cells conditioned medium in the treatment of severe and critically novel coronavirus pneumonia (COVID-19): a randomized controlled trial	http://www.chictr.org.cn/showproj.aspx?proj=49062
ChiCTR2000029602	173	TCM	Clinical study for community based prevention and control strategy of novel coronavirus pneumonia (COVID-19) in the isolate suspected and confirmed population	http://www.chictr.org.cn/showproj.aspx?proj=48985
ChiCTR2000029606	174	Stem Cells	Clinical Study for Human Menstrual Blood-Derived Stem Cells in the Treatment of Acute Novel Coronavirus Pneumonia (COVID-19)	http://www.chictr.org.cn/showproj.aspx?proj=49146
ChiCTR2000029539	175	Ritonavir/Lopinavir	A randomized, open-label study to evaluate the efficacy and safety of Lopinavir-Ritonavir in patients with mild novel coronavirus pneumonia (COVID-19)	http://www.chictr.org.cn/showproj.aspx?proj=48991
ChiCTR2000029572	176	Stem Cells	Safety and efficacy of umbilical cord blood mononuclear cells in the treatment of severe and critically novel coronavirus pneumonia(COVID-19): a randomized controlled clinical trial	http://www.chictr.org.cn/showproj.aspx?proj=41760
ChiCTR2000029559	177	Chloroquine	Therapeutic effect of hydroxychloroquine on novel coronavirus pneumonia (COVID-19)	http://www.chictr.org.cn/showproj.aspx?proj=48880
ChiCTR2000029605	178	TCM	A randomized, open-label, blank-controlled, multicenter trial for Shuang-Huang-Lian oral solution in the treatment of ovel coronavirus pneumonia (COVID-19)	http://www.chictr.org.cn/showproj.aspx?proj=49051

Registry entry ID	Included ID	Intervention	Title	Webpage
ChiCTR2000029544	179	Favipiravir/Baloxavir Marboxil	A randomized controlled trial for the efficacy and safety of Baloxavir Marboxil, Favipiravir tablets in novel coronavirus pneumonia (COVID-19) patients who are still positive on virus detection under the current antiviral therapy	http://www.chictr.org.cn/showproj.aspx?proj=49013
ChiCTR2000029541	180	Ritonavir/Lopinavir/darunavir/cobicistat/thymosin-a1	A randomised, open, controlled trial for darunavir/cobicistat or Lopinavir/ritonavir combined with thymosin a1 in the treatment of novel coronavirus pneumonia (COVID-19)	http://www.chictr.org.cn/showproj.aspx?proj=48992
ChiCTR2000029438	181	TCM	A randomized controlled trial of integrated TCM and Western Medicine in the treatment of severe novel coronavirus pneumonia (COVID-19)	http://www.chictr.org.cn/showproj.aspx?proj=48911
ChiCTR2000029548	182	Ritonavir/Lopinavir/Favipiravir/Baloxavir Marboxil	Randomized, open-label, controlled trial for evaluating of the efficacy and safety of Baloxavir Marboxil, Favipiravir, and Lopinavir-Ritonavir in the treatment of novel coronavirus pneumonia (COVID-19) patients	http://www.chictr.org.cn/showproj.aspx?proj=49015
ChiCTR2000029518	183	TCM	Chinese medicine prevention and treatment program for novel coronavirus pneumonia (COVID-19): a perspective, double-blind, placebo, randomised controlled trial	http://www.chictr.org.cn/showproj.aspx?proj=48860
ChiCTR2000029517	184	TCM	Chinese medicine prevention and treatment program for suspected novel coronavirus pneumonia (COVID-19): a perspective, double-blind, placebo, randomised controlled trial	http://www.chictr.org.cn/showproj.aspx?proj=48861
ChiCTR2000029435	185	TCM	Randomized controlled trial for traditional Chinese medicine in the prevention of novel coronavirus pneumonia (COVID-19) in high risk population	http://www.chictr.org.cn/showproj.aspx?proj=48827
ChiCTR2000029461	186	TCM	A Randomized Controlled Trial for Integrated Traditional Chinese Medicine and Western Medicine in the Treatment of Common Type Novel Coronavirus Pneumonia (COVID-19)	http://www.chictr.org.cn/showproj.aspx?proj=48927
ChiCTR2000029418	187	TCM	Chinese Herbal medicine for Severe nevel coronavirus pneumonia (COVID-19): a Randomized Controlled Trial	http://www.chictr.org.cn/showproj.aspx?proj=48886
ChiCTR2000029495	188	TCM	Traditional Chinese Medicine, Psychological Intervention and Investigation of Mental Health for Patients With Novel Coronavirus Pneumonia (COVID-19) in Convalescent Period	http://www.chictr.org.cn/showproj.aspx?proj=48971
ChiCTR2000029493	189	TCM	Traditional Chinese Medicine for Pulmonary Fibrosis, Pulmonary Function and Quality of Life in Patients With Novel Coronavirus Pneumonia (COVID-19) in Convalescent Period: a Randomized Controlled Trial	http://www.chictr.org.cn/showproj.aspx?proj=48931
ChiCTR2000029439	190	TCM	Combination of traditional Chinese medicine and western medicine in the treatment of common type novel coronavirus pneumonia (COVID-19)	http://www.chictr.org.cn/showproj.aspx?proj=48904
ChiCTR2000029431	191	M1 macrophages	Clinical study for the remedy of M1 macrophages target in the treatment of novel coronavirus pneumonia (COVID-19)	http://www.chictr.org.cn/showproj.aspx?proj=48907
ChiCTR2000030179	192	Convalescent Plasma	Experimental study of novel coronavirus pneumonia rehabilitation plasma therapy severe novel coronavirus pneumonia (COVID-19)	http://www.chictr.org.cn/showproj.aspx?proj=50059
ChiCTR2000030173	193	Stem Cells	Key techniques of umbilical cord mesenchymal stem cells for the treatment of novel coronavirus pneumonia (COVID-19) and clinical application demonstration	http://www.chictr.org.cn/showproj.aspx?proj=49229
ChiCTR2000030166	194	TCM	Randomized, parallel control, open trial for Qing-Wen Bai-Du-Yin combined with antiviral therapy in the treatment of novel coronavirus pneumonia (COVID-19)	http://www.chictr.org.cn/showproj.aspx?proj=49696
ChiCTR2000030188	195	TCM	Clinical Study on Syndrome Differentiation of TCM in Treating Severe and Critical novel coronavirus pneumonia (COVID-19)	http://www.chictr.org.cn/showproj.aspx?proj=50025
ChiCTR2000030167	196	Interleukin-2	Clinical Trial for Recombinant Human Interleukin-2 in the Treatment of Novel Coronavirus Pneumonia (COVID-19)	http://www.chictr.org.cn/showproj.aspx?proj=49567
ChiCTR2000030113	197	Favipiravir	Randomized controlled trial for safety and efficacy of Favipiravir in the treatment of novel coronavirus pneumonia (COVID-19) with poorly responsive ritonavir/ritonavir	http://www.chictr.org.cn/showproj.aspx?proj=49988
ChiCTR2000030138	198	Stem Cells	Clinical Trial for Human Mesenchymal Stem Cells in the Treatment of Severe Novel Coronavirus Pneumonia (COVID-19)	http://www.chictr.org.cn/showproj.aspx?proj=50004
ChiCTR2000030118	199	TCM	A clinical study for "Huo-Shen" particles in the treatment of novel coronavirus pneumonia (COVID-19)	http://www.chictr.org.cn/showproj.aspx?proj=49594
ChiCTR2000030116	200	Stem Cells	Safety and effectiveness of human umbilical cord mesenchymal stem cells in the treatment of acute respiratory distress syndrome of severe novel coronavirus pneumonia (COVID-19)	http://www.chictr.org.cn/showproj.aspx?proj=49901
ChiCTR2000030102	201	ozone autohemotherapy	A multicenter randomized controlled trial for ozone autohemotherapy in the treatment of novel coronavirus pneumonia (COVID-19)	http://www.chictr.org.cn/showproj.aspx?proj=49747
ChiCTR2000030058	202	leflunomide	A multicenter, randomized, double-blind, controlled clinical trial for leflunomide in the treatment of novel coronavirus pneumonia (COVID-19)	http://www.chictr.org.cn/showproj.aspx?proj=49831
ChiCTR2000030043	203	TCM	Shen-Fu injection in the treatment of severe novel coronavirus pneumonia (COVID-19): a multicenter, randomized, open-label, controlled trial	http://www.chictr.org.cn/showproj.aspx?proj=49866
ChiCTR2000030089	204	Adalimumab	A clinical study for the efficacy and safety of Adalimumab Injection in the treatment of patients with severe novel coronavirus pneumonia (COVID-19)	http://www.chictr.org.cn/showproj.aspx?proj=49889
ChiCTR2000030088	205	Stem Cells	Umbilical cord Wharton's Jelly derived mesenchymal stem cells in the treatment of severe novel coronavirus pneumonia (COVID-19)	http://www.chictr.org.cn/showproj.aspx?proj=49902
ChiCTR2000030034	206	TCM	Traditional Chinese Medicine in the treatment of novel coronavirus pneumonia (COVID-19): a multicentre, randomized controlled trial	http://www.chictr.org.cn/showproj.aspx?proj=49647
ChiCTR2000030033	207	TCM	A study for the intervention of Xiangxue antiviral oral solution and Wu-Zhi-Fang-Guan-Fang on close contacts of novel coronavirus pneumonia (COVID-19)	http://www.chictr.org.cn/showproj.aspx?proj=49703
ChiCTR2000030054	208	Chloroquine	A prospective, open label, randomized, control trial for chloroquine or hydroxychloroquine in patients with mild and common novel coronavirus pulmonary (COVIP-19)	http://www.chictr.org.cn/showproj.aspx?proj=49869
ChiCTR2000030016	209	Vaccine mycobacterium w	Basic and clinical study of inhalation of inactivated mycobacterium vaccine in the treatment of Novel coronavirus pneumonia (COVID-19)	http://www.chictr.org.cn/showproj.aspx?proj=49799
ChiCTR2000030007	210	Filgrastim (rhG-CSF)	Multicenter randomized controlled trial for rhG-CSF in the treatment of novel coronavirus pneumonia (COVID-19)	http://www.chictr.org.cn/showproj.aspx?proj=49619
ChiCTR2000030010	211	Convalescent Plasma	A randomized, double-blind, parallel-controlled, trial to evaluate the efficacy and safety of anti-SARS-CoV-2 virus inactivated plasma in the treatment of severe novel coronavirus pneumonia patients (COVID-19)	http://www.chictr.org.cn/showproj.aspx?proj=49777
ChiCTR2000030006	212	ozone autohemotherapy	A randomized controlled trial for the efficacy of ozonated autohemotherapy in the treatment of Novel Coronavirus Pneumonia (COVID-19)	http://www.chictr.org.cn/showproj.aspx?proj=49737
ChiCTR2000030001	213	Triazavirin	The efficacy and safety of Triazavirin for 2019 novel coronary pneumonia (COVID-19): a multicenter, randomized, double blinded, placebo-controlled trial	http://www.chictr.org.cn/showproj.aspx?proj=49723
ChiCTR2000029956	214	TCM	Chinese Medicine Promotes Rehabilitation Recommendations after 2019 Novel Coronavirus Infection (COVID-19)	http://www.chictr.org.cn/showproj.aspx?proj=49618
ChiCTR2000029996	215	Favipiravir	A randomized, open-label, controlled trial for the efficacy and safety of Farpiravir Tablets in the treatment of patients with novel coronavirus pneumonia (COVID-19)	http://www.chictr.org.cn/showproj.aspx?proj=49510
ChiCTR2000029992	216	Chloroquine	A prospective, randomized, open label, controlled trial for chloroquine and hydroxychloroquine in patients with severe novel coronavirus pneumonia (COVID-19)	http://www.chictr.org.cn/showproj.aspx?proj=49574
ChiCTR2000030003	217	TCM	Optimization Protocal of Integrated Traditional Chinese and Western Medicine in the Treatment for Novel Coronavirus Pneumonia (COVID-19)	http://www.chictr.org.cn/showproj.aspx?proj=49770
ChiCTR2000030002	218	Tranilast	Clinical study of novel NLRP Inflammasome inhibitor (Tranilast) in the treatment of novel coronavirus pneumonia (COVID-19)	http://www.chictr.org.cn/showproj.aspx?proj=49738
ChiCTR2000029994	219	TCM	Liu-Zi-Jue Qigong and Acupressure Therapy for Pulmonary Function and Quality of Life in Patient with Severe novel coronavirus pneumonia (COVID-19): A Randomized Controlled Trial	http://www.chictr.org.cn/showproj.aspx?proj=49309
ChiCTR2000029978	220	TCM	A randomized controlled trial for the efficacy of Dao-Yin in the prevention and controlling novel coronavirus pneumonia (COVID-19)	http://www.chictr.org.cn/showproj.aspx?proj=49702
ChiCTR2000029989	221	INF-Beta	A randomized controlled Trial for therapeutic efficacy of Recombinant Human Interferon alpha 1b Eye Drops in the treatment of elderly with novel coronavirus pneumonia (COVID-19)	http://www.chictr.org.cn/showproj.aspx?proj=49720
ChiCTR2000029988	222	Chloroquine	Clinical Study of Chloroquine Phosphate in the Treatment of Severe Novel Coronavirus Pneumonia (COVID-19)	http://www.chictr.org.cn/showproj.aspx?proj=49218
ChiCTR2000029781	223	TCM	A multicenter, randomized, open and controlled trial for the efficacy and safety of Kang-Bing-Du granules in the treatment of novel coronavirus pneumonia (COVID-19)	http://www.chictr.org.cn/showproj.aspx?proj=49138
ChiCTR2000029740	224	Chloroquine	Efficacy of therapeutic effects of hydroxycholoroquine in novel coronavirus pneumonia (COVID-19) patients(randomized open-label control clinical trial)	http://www.chictr.org.cn/showproj.aspx?proj=49317
ChiCTR2000029990	225	Stem Cells	Clinical trials of mesenchymal stem cells for the treatment of pneumonitis caused by novel coronavirus pneumonia (COVID-19)	http://www.chictr.org.cn/showproj.aspx?proj=49674
ChiCTR2000029972	226	Ultra Short Wave Electrotherapy	A randomized controlled trial for the Efficacy of Ultra Short Wave Electrotherapy in the treatment of Novel Coronavirus Pneumonia (COVID-19)	http://www.chictr.org.cn/showproj.aspx?proj=49664
ChiCTR2000029974	227	Probiotic	A prospective, multicenter, open-label, randomized, parallel-controlled trial for probiotics to evaluate efficacy and safety in patients infected with 2019 novel coronavirus pneumonia (COVID-19)	http://www.chictr.org.cn/showproj.aspx?proj=49321
ChiCTR2000029803	228	Chloroquine	A prospective, randomized, open-label, controlled clinical study to evaluate the preventive effect of hydroxychloroquine on close contacts after exposure to the Novel Coronavirus Pneumonia (COVID-19)	http://www.chictr.org.cn/showproj.aspx?proj=49428
ChiCTR2000029780	229	TCM	A multicenter, randomized, open, controlled trial for the efficacy and safety of Shen-Qi Fu-Zheng injection in the treatment of novel coronavirus pneumonia (COVID-19)	http://www.chictr.org.cn/showproj.aspx?proj=49220
ChiCTR2000029851	230	Alpha lipoic acid	A multicenter, randomized controlled trial for the efficacy and safety of Alpha lipoic acid (iv) in the treatment of patients of severe novel coronavirus pneumonia (COVID-19)	http://www.chictr.org.cn/showproj.aspx?proj=49534
ChiCTR2000030418	231	TCM	Application of rehabilitation lung exercise eight-segment exercise in patients with novel coronavirus pneumonia (COVID-19)	http://www.chictr.org.cn/showproj.aspx?proj=50341
ChiCTR2000030398	232	bismuth potassium citrate	A randomized, double-blind, placebo-controlled trial for evaluation of the efficacy and safety of bismuth potassium citrate capsules in the treatment of patients with novel coronavirus pneumonia (COVID-19).	http://www.chictr.org.cn/showproj.aspx?proj=50173
ChiCTR2000030333	233	Pirfenidone	A randomized, open-label controlled trial for the efficacy and safety of Pirfenidone in patients with severe and critical novel coronavirus pneumonia (COVID-19)	http://www.chictr.org.cn/showproj.aspx?proj=48801
ChiCTR2000030328	234	Acetylcysteine	Clinical application of inhaled acetylcysteine solution in the treatment of novel coronavirus pneumonia (COVID-19)	http://www.chictr.org.cn/showproj.aspx?proj=50241
ChiCTR2000030262	235	IFN-κ + TFF2	Clinical study for combination of anti-viral drugs and type I interferon and inflammation inhibitor TFF2 in the treatment of novel coronavirus pneumonia (COVID-19)	http://www.chictr.org.cn/showproj.aspx?proj=50136
ChiCTR2000030260	236	TCM	Clinical study for individualized nutritional assessment and supportive treatment of novel coronavirus pneumonia (COVID-19) patients in Tibetan Plateau	http://www.chictr.org.cn/showproj.aspx?proj=50130
ChiCTR2000030265	237	CRRT	Study for continuous renal replacement therapy with adsorption filter in the treatment of the novel coronavirus pneumonia (COVID-19)	http://www.chictr.org.cn/showproj.aspx?proj=49691
ChiCTR2000030215	238	TCM	Study for the efficacy of Kangguan No. 1-3 prescription in the treatment of novel coronavirus pneumonia (COVID19)	http://www.chictr.org.cn/showproj.aspx?proj=50107
ChiCTR2000030261	239	Aerosol inhalation of exosomes	A study for the key technology of mesenchymal stem cells exosomes atomization in the treatment of novel coronavirus pneumonia (COVID-19)	http://www.chictr.org.cn/showproj.aspx?proj=49963
ChiCTR2000030255	240	TCM	Efficacy and Safety of Jing-Yin Granule in the treatment of novel coronavirus pneumonia (COVID-19) wind-heat syndrome	http://www.chictr.org.cn/showproj.aspx?proj=50089
ChiCTR2000030259	241	Ritonavir/Danorevir	Evaluation Danorevir sodium tablets combined with ritonavir in the treatment of novel coronavirus pneumonia (COVID-19): a randomized, open and controlled trial	http://www.chictr.org.cn/showproj.aspx?proj=49918
ChiCTR2000030022	242	TCM	A parallel, randomized controlled clinical trial for the efficacy and safety of Pediatric Huatanzhike granules (containing ipecacuanha tincture) in the treatment of mild and moderate novel coronavirus pneumonia (COVID-19)	http://www.chictr.org.cn/showproj.aspx?proj=49797
ChiCTR2000030218	243	TCM	Study of Pinavir / Ritonavir Tablets (Trade Name: Kelizhi) Combined with Xiyanping Injection for Novel Coronavirus Pneumonia (COVID-19)	http://www.chictr.org.cn/showproj.aspx?proj=50115
ChiCTR2000029991	244	TCM	A randomized, open-label, controlled trial for the safety and efficiency of Kesuting syrup and Keqing capsule in the treatment of mild and moderate novel coronavirus pneumonia (COVID-19)	http://www.chictr.org.cn/showproj.aspx?proj=49666
ChiCTR2000029755	245	TCM	A randomized, open, parallel-controlled clinical trial on the efficacy and safety of Jingyebaidu granules in treating novel coronavirus pneumonia (COVID-19)	http://www.chictr.org.cn/showproj.aspx?proj=49301
ChiCTR2000030225	246	TCM	Clinical Reseach of Acupuncture in the treatment of Novel Coronavirus Pneumonia (COVID-19)	http://www.chictr.org.cn/showproj.aspx?proj=49945
ChiCTR2000029387	247	Ribavirin/interferon-alpha/lopinavir/ritonavir/LPV-r	Comparative effectiveness and safety of ribavirin plus interferon-alpha, lopinavir/ritonavir plus interferon-alpha and ribavirin plus lopinavir/ritonavir plus interferon-alphain in patients with mild to moderate novel coronavirus pneumonia	http://www.chictr.org.cn/showproj.aspx?proj=48782
ChiCTR2000029386	248	Glucocorticoid	Effectiveness of glucocorticoid therapy in patients with severe novel coronavirus pneumonia: a randomized controlled trial	http://www.chictr.org.cn/showproj.aspx?proj=48777
ChiCTR2000029899	249	Chloroquine	Evaluation the Efficacy and Safety of Hydroxychloroquine Sulfate in Comparison with Phosphate Chloroquine in Mild and Commen Patients with Novel Coronavirus Pneumonia (COVID-19): a Randomized, Open-label, Parallel, Controlled Trial	http://www.chictr.org.cn/showproj.aspx?proj=49536
ChiCTR2000029898	250	Chloroquine	Evaluation the Efficacy and Safety of Hydroxychloroquine Sulfate in Comparison with Phosphate Chloroquine in Severe Patients with Novel Coronavirus Pneumonia (COVID-19): a Randomized, Open-Label, Parallel, Controlled Trial	http://www.chictr.org.cn/showproj.aspx?proj=49482
ChiCTR2000030535	251	Ebastine	Multi-Center Clinical Study on the Treatment of Patients with Novel Coronavirus Pneumonia (COVID-19) by Ebastine	http://www.chictr.org.cn/showproj.aspx?proj=49790
ChiCTR2000030522	252	TCM	Efficacy and safety of Ma-Xing-Gan-Shi decoction in the treatment of novel coronavirus pneumonia (COVID-19)	http://www.chictr.org.cn/showproj.aspx?proj=44213
ChiCTR2000030540	253	CRRT dialysis	Research for the mechanism of improvement of novel coronavirus pneumonia (COVID-19) patients' pulmonary exudation by continuous renal replacement therapy	http://www.chictr.org.cn/showproj.aspx?proj=50658
ChiCTR2000030627	254	Convalescent Plasma	Study for using the healed novel coronavirus pneumonia (COVID-19) patients plasma in the treatment of severe critical cases	http://www.chictr.org.cn/showproj.aspx?proj=50727
ChiCTR2000030518	255	TCM	Zedoary Turmeric Oil for Injection in the treatment of Novel Coronavirus Pneumonia (COVID-19): a randomized, open, controlled trial	http://www.chictr.org.cn/showproj.aspx?proj=50586
ChiCTR2000030578	256	Pulmonary rehabilitation	Clinical Prediction and Intervention of Pulmonary Function Impairment in Patients with Novel Coronavirus Pneumonia (COVID-19)	http://www.chictr.org.cn/showproj.aspx?proj=50690
ChiCTR2000030471	257	Lipoic acid	Efficacy and safety of lipoic acid injection in reducing the risk of progression in common patients with novel coronavirus pneumonia (COVID-19)	http://www.chictr.org.cn/showproj.aspx?proj=50421
ChiCTR2000030484	258	Stem Cells	HUMSCs and Exosomes Treating Patients with Lung Injury following Novel Coronavirus Pneumonia (COVID-19)	http://www.chictr.org.cn/showproj.aspx?proj=50263
ChiCTR2000030481	259	Glucocorticoid	The clinical value of corticosteroid therapy timing in the treatment of novel coronavirus pneumonia (COVID-19): a prospective randomized controlled trial	http://www.chictr.org.cn/showproj.aspx?proj=50453
ChiCTR2000030467	260	TCM	A Randomized Controlled Trial for the Influence of TCM Psychotherapy on Negative Emotion of Patients with Novel Coronavirus Pneumonia (COVID-19) Based on Network Platform	http://www.chictr.org.cn/showproj.aspx?proj=50378
ChiCTR2000030469	261	TCM	A randomized parallel controlled trial for LIUSHENWAN in Treatment of Novel Coronavirus Pneumonia (COVID-19)	http://www.chictr.org.cn/showproj.aspx?proj=50082
ChiCTR2000030480	262	INF-Alpha	Randomized, open, blank controlled trial for the efficacy and safety of recombinant human interferon alpha 1beta in the treatment of Wuhan patients with novel coronavirus pneumonia (COVID-19)	http://www.chictr.org.cn/showproj.aspx?proj=50470
ChiCTR2000030479	263	TCM	Study for the Effectiveness and Safety of Yi-Qi Hua-shi Jie-Du-Fang in the Treatment of the Novel Coronavirus Pneumonia (COVID-19)	http://www.chictr.org.cn/showproj.aspx?proj=50450
ChiCTR2000029993	264	TCM	A pilot study for Integrated Chinese and Western Medicine in the treatment of non-critical novel coronavirus pneumonia (COVID-19)	http://www.chictr.org.cn/showproj.aspx?proj=49435
ChiCTR2000029638	265	INF (rSIFN-co)	A Multicenter, Randomized, Controlled trial for Recombinant Super-Compound Interferon (rSIFN-co) in the Treatment of 2019 Novel Coronavirus (2019-nCoV) Infected Pneumonia	http://www.chictr.org.cn/showproj.aspx?proj=49224
ChiCTR2000029580	266	Ruxolitinib/stem cell	Severe novel coronavirus pneumonia (COVID-19) patients treated with ruxolitinib in combination with mesenchymal stem cells: a prospective, single blind, randomized controlled clinical trial	http://www.chictr.org.cn/showproj.aspx?proj=49088
ChiCTR2000029434	267	TCM	A randomized, open-label, blank-controlled trial for Lian-Hua Qing-Wen Capsule /Granule in the treatment of novel coronavirus pneumonia (COVID-19)	http://www.chictr.org.cn/showproj.aspx?proj=48889
ChiCTR2000029757	268	Convalescent Plasma	Convalescent plasma for the treatment of severe and critical novel coronavirus pneumonia (COVID-19): a prospective randomized controlled trial	http://www.chictr.org.cn/showproj.aspx?proj=49081

Registry entry ID	Included ID	Intervention	Title	Webpage
ChiCTR2000029549	269	TCM	Recommendations of Integrated Traditional Chinese and Western Medicine for Novel Coronavirus Pneumonia (COVID-19)	http://www.chictr.org.cn/showproj.aspx?proj=49014
ChiCTR2000029479	270	TCM	Research for Traditional Chinese Medicine Technology Prevention and Control of Novel Coronavirus Pneumonia (COVID-19) in the Community Population	http://www.chictr.org.cn/showproj.aspx?proj=48773
ChiCTR2000029308	271	Ritonavir/Lopinavir	A randomized, controlled open-label trial to evaluate the efficacy and safety of lopinavir-ritonavir in hospitalized patients with novel coronavirus pneumonia (COVID-19)	http://www.chictr.org.cn/showproj.aspx?proj=48684
ChiCTR2000030897	272	Probiotic	Evaluation of the effect of taking Newgen beta-gluten probiotic composite powder to nutrition intervention of patients with novel coronavirus pneumonia (COVID-19)	http://www.chictr.org.cn/showproj.aspx?proj=50462
ChiCTR2000030892	273	Pirfenidone	Efficacy and Safety of Pirfenidone in the Treatment of Severe Post-Novel Coronavirus Pneumonia (COVID-19) Fibrosis: a prospective exploratory experimental medical study	http://www.chictr.org.cn/showproj.aspx?proj=51118
ChiCTR2000030853	274	Dexmedetomidine	Evaluation of the protective effect of dexmedetomidine on patients with severe novel coronavirus pneumonia (COVID-19)	http://www.chictr.org.cn/showproj.aspx?proj=51081
ChiCTR2000030804	275	TCM	Exocarpium Citri Grandis Relieves Symptoms of Novel Coronavirus Pneumonia (COVID-19): a Randomized Controlled Clinical Trial	http://www.chictr.org.cn/showproj.aspx?proj=51018
ChiCTR2000030779	276	Ulinastatin	A clinical trial for Ulinastatin Injection in the treatment of patients with severe novel coronavirus pneumonia (COVID-19)	http://www.chictr.org.cn/showproj.aspx?proj=50973
ChiCTR2000030759	277	TCM	Study for the therapeutic effect and mechanism of traditional Chinese medicine in the treatment of novel coronavirus pneumonia (COVID-19)	http://www.chictr.org.cn/showproj.aspx?proj=49284
ChiCTR2000030750	278	Vaccine	A clinical study for effectiveness and safety evaluation for recombinant chimeric COVID-19 epitope DC vaccine in the treatment of novel coronavirus pneumonia	http://www.chictr.org.cn/showproj.aspx?proj=50928
ChiCTR2000030720	279	TCM	Prognosis Investigation and Intervention Study on Patients with novel coronavirus pneumonia (COVID-19) in recovery period Based on Community Health Management	http://www.chictr.org.cn/showproj.aspx?proj=50793
ChiCTR2000030701	280	Enoxaparin	A randomized, parallel controlled open-label trial for the efficacy and safety of Prolongin (Enoxaparin Sodium Injection) in the treatment of adult patients with novel coronavirus pneumonia (COVID-19)	http://www.chictr.org.cn/showproj.aspx?proj=50795
ChiCTR2000030702	281	Convalescent Plasma	Plasma of the convalescent in the treatment of novel coronavirus pneumonia (COVID-19) common patient: a prospective clinical trial	http://www.chictr.org.cn/showproj.aspx?proj=50537
ChiCTR2000030700	282	Enoxaparin	Study for the efficacy and safety of Prolongin (Enoxaparin Sodium Injection) in treatment of novel coronavirus pneumonia (COVID-19) adult common patients	http://www.chictr.org.cn/showproj.aspx?proj=50786
ChiCTR2000030703	283	Ixekizumab	A randomized, blinded, controlled, multicenter clinical trial to evaluate the efficacy and safety of Ixekizumab combined with conventional antiviral drugs in patients with novel coronavirus pneumonia (COVID-19)	http://www.chictr.org.cn/showproj.aspx?proj=50251
ChiCTR2000030545	284	TCM	Efficacy and safety of honeysuckle oral liquid in the treatment of novel coronavirus pneumonia (COVID-19): a multicenter, randomized, controlled, open clinical trial	http://www.chictr.org.cn/showproj.aspx?proj=50126
ChiCTR2000030580	285	Tocilizumab/adamumab	Efficacy and safety of tozumab combined with adamumab(Qletli) in severe and critical patients with novel coronavirus pneumonia (COVID-19)	http://www.chictr.org.cn/showproj.aspx?proj=50693
ChiCTR2000030388	286	TCM	Efficacy and safety of Xue-Bi-Jing injection in the treatment of severe cases of novel coronavirus pneumonia (COVID-19)	http://www.chictr.org.cn/showproj.aspx?proj=50306
ChiCTR2000030187	287	Ritonavir/Lopinavir	Clinical study for Lopinavir and Ritonavir in the treatment of novel coronavirus pneumonia (COVID-19)	http://www.chictr.org.cn/showproj.aspx?proj=50057
ChiCTR2000031138	288	Pirfenidone	Prospective exploratory clinical research on the efficacy and safety of pirfenidone capsules in the treatment of severe novel coronavirus pneumonia (COVID-19)	http://www.chictr.org.cn/showproj.aspx?proj=51416
ChiCTR2000030987	289	Chloroquine	A Randomized Controlled Trial for Favipiravir Tablets Combine With Chloroquine Phosphate in the Treatment of Novel Coronavirus Pneumonia (COVID-19)	http://www.chictr.org.cn/showproj.aspx?proj=51329
ChiCTR2000030996	290	TCM	Effect of Auricular point pressing on insomnia of novel coronavirus pneumonia (COVID-19) patients: a randomized controlled trial.	http://www.chictr.org.cn/showproj.aspx?proj=51349
ChiCTR2000030933	291	TCM	Effectiveness of "Liu-Zi-Jue" combined with respiratory muscle training for respiratory function in novel coronavirus pneumonia (COVID-19) patients: a randomized controlled trial	http://www.chictr.org.cn/showproj.aspx?proj=51184
ChiCTR2000030944	292	Stem Cells	Clinical study of human NK cells and MSCs transplantation for severe novel coronavirus pneumonia (COVID-19)	http://www.chictr.org.cn/showproj.aspx?proj=50199
ChiCTR2000030929	293	Convalescent Plasma	A randomized, double-blind, parallel-controlled trial to evaluate the efficacy and safety of anti-SARS-CoV-2 virus inactivated plasma in the treatment of severe novel coronavirus pneumonia (COVID-19)	http://www.chictr.org.cn/showproj.aspx?proj=50696
ChiCTR2000029868	294	Chloroquine	Hydroxychloroquine treating novel coronavirus pneumonia (COVID-19): a randomized controlled, open label, multicenter trial	http://www.chictr.org.cn/showproj.aspx?proj=49524
ChiCTR2000030922	295	Interferon A+ribavirin/Umifenovir+ribavirin	Prospective, open-label, controlled, multicenter cohort study of long-acting interferon plus ribavirin in patients with novel coronavirus pneumonia (COVID-19)	http://www.chictr.org.cn/showproj.aspx?proj=51141
ChiCTR2000030254	296	Favipiravir/Umifenovir	the Efficacy and Safety of Favipiravir for novel coronavirus–infected pneumonia: A multicenter, randomized, open, positive, parallel-controlled clinical study	http://www.chictr.org.cn/showproj.aspx?proj=50137
ChiCTR2000029658	297	high-flow therapy by nasal cannulae (HFNC)	Nasal high-fow preoxygenation assisted fibre-optic bronchoscope intubation in patients with critical novel coronavirus pneumonia (COVID-19)	http://www.chictr.org.cn/showproj.aspx?proj=49074
ChiCTR2000029573	298	Novaferon atomization/TCM?	A multicenter, randomized, open, controlled clinical study to evaluate the efficacy and safety of recombinant cytokine gene-derived protein injection in combination with standard therapy in patients with novel coronavirus infection	http://www.chictr.org.cn/showproj.aspx?proj=49065
ChiCTR2000029496	299	Novaferon	A randomized, open label, parallel controlled trial of evaluating the efficacy of recombinant cytokine gene-derived protein injection for clearing novel coronavirus in patients with novel coronavirus pneumonia (COVID-19)	http://www.chictr.org.cn/showproj.aspx?proj=48809
ChiCTR2000031319	300	Stem Cells	Safety and Efficacy Study of Allogeneic Human Dental Pulp Mesenchymal Stem Cells to Treat Severe novel coronavirus pneumonia (COVID-19) patients	http://www.chictr.org.cn/showproj.aspx?proj=51647
ChiCTR2000031271	301	Nutrtion/Amino Acids	Metabolic abnormalities and rearrangement reversal treatment of patient with novel coronavirus pneumonia (COVID-19)	http://www.chictr.org.cn/showproj.aspx?proj=51194
ChiCTR2000031203	302	TCM	Study for the effect of Moxibustion Guidance and Intervention based on internet for the discharged patients with novel coronavirus pneumonia (COVID-19)	http://www.chictr.org.cn/showproj.aspx?proj=51030
ChiCTR2000031204	303	Chloroquine	A multicenter, single-blind, randomized controlled clinical trial for chloroquine phosphate in the treatment of novel coronavirus pneumonia (COVID-19)	http://www.chictr.org.cn/showproj.aspx?proj=49420
ChiCTR2000030937	304	TCM	A randomized, open-label, controlled trial for Gu-Shen Ding-Chuan-Wan in the treatment of patients with novel coronavirus pneumonia (COVID-19) at recovery phase with Fei-Pi-Qi-Xu Zhen	http://www.chictr.org.cn/showproj.aspx?proj=51240
ChiCTR2000030988	305	TCM	A RCT for Hua-Shi Bai-Du granules in patients with novel coronavirus pneumonia (COVID-19)	http://www.chictr.org.cn/showproj.aspx?proj=51317
ChiCTR2000030923	306	TCM	The treatment and diagnosis plan of integrated traditional Chinese and Western medicine for novel coronavirus pneumonia (COVID-19)	http://www.chictr.org.cn/showproj.aspx?proj=51139
ChiCTR2000030288	307	TCM	Efficacy of Traditional Chinese Medicine in the Treatment of Novel Coronavirus Pneumonia (COVID-19): a Randomized Controlled Trial	http://www.chictr.org.cn/showproj.aspx?proj=50202
ChiCTR2000030704	308	TCM	Observation Of Clinical Efficacy And Safety Of Bufonis Venenum Injection In The Treatment Of Severe Novel Coronavirus Pneumonia (COVID-19)	http://www.chictr.org.cn/showproj.aspx?proj=50778
ChiCTR2000030028	309	PD-1 mAb(Monoclonal antibodies)	Clinical comparative study of PD-1 mAb in the treatment of severe and critical patients with novel coronavirus pneumonia (COVID-19)	http://www.chictr.org.cn/showproj.aspx?proj=49840
ChiCTR2000030198	310	Pulmonary rehabilitation	Clinical research of pulmonary rehabilitation in survivors due to severe or critial novel coronavirus pneumonia (COVID-19)	http://www.chictr.org.cn/showproj.aspx?proj=50066
ChiCTR2000029976	311	TCM	The effect of Gymnastic Qigong Yangfeifang on functional recovery and quality of life in patients with mild novel coronavirus pneumonia (COVID-19)	http://www.chictr.org.cn/showproj.aspx?proj=49631
ChiCTR2000031453	312	Nintedanib (tyrosine kinase)	Efficacy and safety of Nintedanib esilate soft capsules in the treatment of pulmonary fibrosis in healed moderate to severe patients of novel coronavirus pneumonia (COVID-19): a single-center, randomized, placebo-controlled trial	http://www.chictr.org.cn/showproj.aspx?proj=51859
ChiCTR2000031454	313	Chloroquine + other	Clinical study for prevention and treatment of digestive tract lesions caused by novel coronavirus pneumonia (COVID-19)	http://www.chictr.org.cn/showproj.aspx?proj=51693
ChiCTR2000031672	314	TCM	Development and application of TCM body regulating protection scheme for the convalescent population of novel coronavirus pneumonia (COVID-19)	http://www.chictr.org.cn/historyversionpuben.aspx?regno=ChiCTR2000031672
ChiCTR2000031174	315	Chloroquine	Effectiveness and safety of hydroxychloroquine sulfate in the preventive treatment of novel coronavirus pneumonia (COVID-19)	http://www.chictr.org.cn/historyversionpuben.aspx?regno=ChiCTR2000031174
NCT04333368	316	Stem cells	Cell Therapy Using Umbilical Cord-derived Mesenchymal Stromal Cells in SARS-CoV-2-related ARDS	https://clinicaltrials.gov/ct2/show/NCT04333368
NCT04329195	317	ACE inhibitor	ACE Inhibitors or ARBs Discontinuation in Context of SARS-CoV-2 Pandemic	https://clinicaltrials.gov/ct2/show/NCT04329195
NCT04323527	318	Chloroquine	Chloroquine Diphosphate for the Treatment of Severe Acute Respiratory Syndrome Secondary to SARS-CoV2	https://clinicaltrials.gov/ct2/show/NCT04323527
NCT04303299	319	Chloroquine + other	Various Combination of Protease Inhibitors, Oseltamivir, Favipiravir, and Hydroxychloroquine for Treatment of COVID19 : A Randomized Control Trial	https://clinicaltrials.gov/ct2/show/NCT04303299
NCT04299152	320	Stem cells	Stem Cell Educator Therapy Treat the Viral Inflammation Caused by Severe Acute Respiratory Syndrome Coronavirus 2	https://clinicaltrials.gov/ct2/show/NCT04299152
DRKS00021214	321	Vitamin B3	Improvement of the nutritional status regarding nicotinamide (vitamin B3) and the course of COVID-19 disease	https://www.drks.de/drks_web/navigate.do?navigationId=trial.HTML&TRIAL_ID=DRKS00021214
CTRI024402	322	Chloroquine	Hydroxy Chloroquine, in open labelled, Randomised intervention for prevention of new infection and adverse outcomes following COVID-19 infection -A Tertiary Hospital based study	http://ctri.nic.in/Clinicaltrials/pmaindet2.php?trialid=42638&EncHid=&userName=024402
CTRI024479	323	Chloroquine	Study of the effect of Chloroquine in addition to standard therapy in COVID-19 patients	http://ctri.nic.in/Clinicaltrials/pmaindet2.php?trialid=42713&EncHid=&userName=024479
IRCT20160310026998N11	324	Metformin	Study of the metformin effect on the survival and recovery rate of COVID-19 patients	https://www.irct.ir/trial/46690
IRCT20180923041093N3	325	TCM	Efficacy of the myrtle (Myrtus Communis) syrup in the treatment of suspected novel coronavirus pneumonia (COVID-19)	https://www.irct.ir/trial/46721
IRCT20180802040678N4	326	Losartan	Evaluation of the effects of Losartan in patients with corona virus disease 2019	https://www.irct.ir/trial/46824
IRCT20200323046841N1	327	TCM	Efficacy evaluation of an herbal compound “Fluherb” on clinical symptoms and paraclinical parameters in highly suspected COVID-19 patients: a controlled randomized clinical trial.	https://www.irct.ir/trial/46715
IRCT20120826010664N6	328	Chloroquine	Effect of hydroxychloroquine on prevention of covid-19 virus infection among treatment staff in Arash hospital-A double-blind clinical trial	https://www.irct.ir/trial/46603
IRCT20200324046850N3	329	Naproxen	The effect of naproxen on the healing process of patients with COVID-19	https://www.irct.ir/trial/46718
IRCT20151113025025N3	330	ACE inhibitor	Clinical Trial of renin-angiotensin-aldosterone system inhibitors with halting their administration and the effect on clinical outcomes of patients with corona virus disease-2019 (COVID-19) referring to Sina Hospital in 2020	https://www.irct.ir/trial/46531
IRCT20101219005416N2	331	TCM	Evaluation of the effect of Traditional medicine product incorporated in the base of Astragalus gossypinus with food spice on improvement of clinical menifestation of COVID -19 patients with conventional protocol	https://www.irct.ir/trial/46775
IRCT20180923041093N4	332	TCM	Efficacy of the barley-based remedy in the treatment of suspected novel coronavirus (COVID-19) pneumonia	https://www.irct.ir/trial/46728
IRCT20081019001369N2	333	TCM	Clinical trial of berberine against COVID-19	https://www.irct.ir/trial/46868
IRCT20180610040049N4	334	TCM	The comparison of effectiveness of Tanacetum parthenium (L) Sch. syrups on clinical and paraclinical features of inpatients infected with 2019 novel coronavirus COVID-19, Parallel randomized trial	https://www.irct.ir/trial/46850
IRCT20180610040049N3	335	TCM	The effect of Trachyspermum copticum-based traditional medicine product on clinical manifestations and Para clinical features in patients with COVID-19: A Randomized clinical trial	https://www.irct.ir/trial/46697
IRCT20140528017891N8	336	Stem Cells	Evaluation of the efficacy and safety of cord-derived mesenchymal stem cell transplantation in the treatment of COVID-19	https://www.irct.ir/trial/46623
IRCT20130917014693N10	337	Chloroquine	Evaluation the efficacy and safety of Hydroxychloroquine administration for COVID-19 post exposure prophylaxis	https://www.irct.ir/trial/46849
IRCT20200324046850N5	338	Vitamin C	Study of the effect of Vitamin C on duration of hospital stay in patients with Covid-19	https://www.irct.ir/trial/46751
IRCT20200317046797N2	339	fingolimod	Effect of Fingolimod for treatment of COVID-19-induced cytokine storm	https://www.irct.ir/trial/46786
IRCT20190727044343N1	340	febuxostat	The effect of febuxostat against lung injury induced by COVID-19 virus in infected patients: A clinical trial study	https://www.irct.ir/trial/46629
IRCT20140617018126N2	341	TCM	The Effect of Persian Medicine Products on the Clinical and Laboratory Symptoms of Patients With or Suspected of Covid-19	https://www.irct.ir/trial/46637
IRCT20200317046797N1	342	Camostat mesilate	Effect of Camostate mesylate on clinical improvement and outcome of Coronavirus (COVID-19)-induced pneumonia	https://www.irct.ir/trial/46573
IRCT20150914024017N1	343	INF-Beta	The efficacy of Interferon beta 1 a (ReciGen) in treatment of patients with Covid-19 in Sina Hospital Emergency Department	https://www.irct.ir/trial/46665
IRCT20200310046736N1	344	Convalescent Plasma	Comparison of The Therapeutic Effect of Convalescent Plasma and Plasma-derived Immunoglobulin-enriched solution on COVID-19 Patients: A Clinical Trial Study	https://www.irct.ir/trial/46424
IRCT20160316027081N1	345	TCM	Comparison of the Effectiveness of Standard Treatment with Standard Treatment plus licorice extract in Improving Respiratory Symptoms and Survival Rate in COVID-19 Patients	https://www.irct.ir/trial/46678
IRCT20200322046833N1	346	Chloroquine + other	Evaluation of the efficacy and safety of Umifenovir in the treatment of hospitalized patients with covid-19: A randomized clinical trial	https://www.irct.ir/trial/46731
IRCT20190917044805N2	347	Vitamin C	Effects of High-dose Vitamin C on Treatment, Clinical Symptoms and Laboratory Signs of Iranian COVID-19 Patients: A Double Blind Randomized Clinical Trial	https://www.irct.ir/trial/46804
IRCT20200319046819N1	348	Vitamin B/A/D/E/C	Impact of vitamin B, A, D, E, C supplementation on improvement and mortality rate in patients with COVID-19 admitted in intensive care unit	https://www.irct.ir/trial/46838
IRCT20200318046812N1	349	Chloroquine + other	Evaluation of safety and efficacy of hydroxychloroquine plus favipiravir drug regimen in comparison with hydroxychloroquine plus kaletra on the need for intensive care unit treatment in patients with COVID-19; a randomized, multicenter, parallel groups, open label study	https://www.irct.ir/trial/46783
IRCT20140907019073N4	350	TCM	Double blind clinical trial study on Evaluation of the effectiveness of Ipecac homeopathic remedy in control of Clinical manifestations of COVID-19	https://www.irct.ir/trial/46736
IRCT20200324046850N1	351	D3 and N-acetylcysteine	Comparison of vitamin D3 and N-acetylcysteine prescription in COVID19 patients and their effect on recovery process	https://www.irct.ir/trial/46732
IRCT20200405046953N1	352	Chloroquine + other	Randomzied trial of additional treatments for COVID-19 in hospitalized patients who are all receiving the local standard of care- Iranian SOLIDARITY multicentre trial	https://www.irct.ir/trial/46930
IRCT20200312046749N1	353	Altebrel	Evaluation the efficacy of Tumor Necrosis Factor alpha inhibitor in COVID-19 outcomes: a prospective clinical trial study.	https://www.irct.ir/trial/46551
IRCT20180712040449N2	354	TCM	Formulation and evaluation of their efficacy of herbal capsule and decoction in patients with COVID-19 via a randomized clinical trial.	https://www.irct.ir/trial/46782
IRCT20120225009124N4	355	Glucocorticoid / IV-IG / INF-beta	Efficacy of different methods of administeration of combination regiemn including Glucocorticoide, IV-IG and Interferone beta for treatment of patients with severe COVID-19: a randomized controlled trial	https://www.irct.ir/trial/46810
IRCT20130812014333N145	356	Sofosbuvir and Velpatasvir	Comparative assessment of the efficacy and safety of add ontreatment with “Sofosbuvir plus Velpatasvir” to “standard of care therapeutic regimen” in patients with COVID-19	https://www.irct.ir/trial/46790
IRCT20200318046812N2	357	Chloroquine + other	Safety and efficacy of “Hydroxychloroquine + Azithromycin + Naproxen + Prednisolone” and “Hydroxychloroquine + Azithromycin + Naproxen” regimens in comparison with “Hydroxychloroquine + kaletra” on the need for intensive care unit treatment in patients with COVID-19; a randomized, multicenter, parallel	https://www.irct.ir/trial/46968
IRCT20200328046882N1	358	Sofosbuvir	Efficacy and safety of Sofosbuvir in the treatment of SARS-CoV-2: An Open Label phase II Trial	https://www.irct.ir/trial/46784

Registry entry ID	Included ID	Intervention	Title	Webpage
IRCT20170117032004N3	359	Vitamin A	Evaluation the effect of vitamin A on respiratory signs and hospitalization in patients with COVID-19	https://www.irct.ir/trial/46564
EUCTR2020-001194-69	360	Mefloquine	Pilot study to evaluate the efficacy and safety of mefloquine as prophylaxis in people exposed to the disease caused by the SARS-CoV-2 coronavirus (COVID-19)	https://www.clinicaltrialsregister.eu/ctr-search/trial/2020-001194-69/ES
EUCTR2020-001421-31	361	Chloroquine	Clinical trial randomized, unblinded and controled for evaluation of efficacy and safety of hydroxychloroquine chemoprophylaxis against SARS-CoV-2 (COVID-19) infection in healthcare professionals.	https://www.clinicaltrialsregister.eu/ctr-search/trial/2020-001421-31/ES
EUCTR2020-001445-39	362	Glucocorticoid/Tacrolimus	PRAGMATIC, CONTROLLED, OPEN, SINGLE CENTER, RANDOMIZED, PHASE II CLINICAL TRIAL TO EVALUATE METHYLPREDNISOLONE PULSES AND TACROLIMUS IN HOSPITALIZED PATIENTS WITH SEVERE PNEUMONIA SECONDARY TO COVID-19 (TACROVID)	https://www.clinicaltrialsregister.eu/ctr-search/trial/2020-001445-39/ES
EUCTR2020-001606-33	363	Chloroquine + other	Randomized clinical trial to evaluate the efficacy of hydroxychloroquine associated or not with azithromycin as a treatment for COVID-19 infection.	https://www.clinicaltrialsregister.eu/ctr-search/trial/2020-001606-33/ES
EUCTR2020-001246-18	364	Chloroquine + other	Cohort Multiple randomized controlled trials open-label of immune modulatory drugs and other treatments in COVID-19 patients	https://www.clinicaltrialsregister.eu/ctr-search/trial/2020-001246-18/FR
EUCTR2020-001366-11	365	Chloroquine + other	An international randomised trial of additional treatments for COVID-19 in hospitalised patients who are all receiving the local standard of care	https://www.clinicaltrialsregister.eu/ctr-search/trial/2020-001366-11/ES
EUCTR2020-001113-21	366	Chloroquine/Lopinavir/ritonavir/azithromycin	Randomised Evaluation of COVID-19 Therapy (RECOVERY)	https://www.clinicaltrialsregister.eu/ctr-search/trial/2020-001113-21/GB
EUCTR2020-001310-38	367	Convalescent Plasma	A randomized, prospective, open label clinical trial on the use of convalescent plasma compared to best supportive care in patients with severe COVID-19	https://www.clinicaltrialsregister.eu/ctr-search/trial/2020-001310-38/DE
EUCTR2020-001307-16	368	Glucocorticoid	Efficacy and Safety of corticoids in patients with adult respiratory distress syndrome (ARDS) secondary to COVID-19.	https://www.clinicaltrialsregister.eu/ctr-search/trial/2020-001307-16/ES
ChiCTR2000030055	369	Dipyridamole	Multicenter study for the treatment of Dipyridamole with novel coronavirus pneumonia (COVID-19)	http://www.chictr.org.cn/showproj.aspx?proj=49864
IRCT20200328046886N1	370	Sovodak		https://www.irct.ir/trial/46885
IRCT20180520039738N2	371	Vitamin A		https://www.irct.ir/trial/46974
IRCT20200404046934N1	372	Zufa syrup		https://www.irct.ir/trial/46913
IRCT20200404046933N1	373	Licorice		https://www.irct.ir/trial/46893
IRCT20151227025726N14	374	Favipiravir		https://www.irct.ir/trial/46942
IRCT20200317046797N3	375	IVIG		https://www.irct.ir/trial/47014
IRCT20200409047007N1	376	Convalescent Plasma		https://www.irct.ir/trial/47058
IRCT20200403046926N1	377	Sovodak		https://www.irct.ir/trial/46926
IRCT20161204031229N3	378	Teicoplanin		https://www.irct.ir/trial/46814
IRCT20200402046923N1	379	Fenugreek syrup		https://www.irct.ir/trial/46879
IRCT20081019001369N3	380	crocetin		https://www.irct.ir/trial/46978
IRCT20150808023559N20	381	Chloroquine + other		https://www.irct.ir/trial/47022
IRCT20200406046965N1	382	Elderberry		https://www.irct.ir/trial/46958
IRCT20081019001369N4	383	Minocycline		https://www.irct.ir/trial/46977
IRCT20080901001157N16	384	IMFLUNA herbal compound		https://www.irct.ir/trial/46907
IRCT20151228025732N52	385	Vitamin C/melatonin/zinc		https://www.irct.ir/trial/46963
EUCTR2020-001290-74	386	Sarilumab		https://www.clinicaltrialsregister.eu/ctr-search/trial/2020-001290-74/ES
EUCTR2020-001273-73	387	Zitromax		https://www.clinicaltrialsregister.eu/ctr-search/trial/2020-001273-73/FR
EUCTR2020-001156-18	388	Chloroquine / Azithromycin		https://www.clinicaltrialsregister.eu/ctr-search/trial/2020-001156-18/ES
EUCTR2020-001160-28	389	Tocilizumab+pembrolizumab		https://www.clinicaltrialsregister.eu/ctr-search/trial/2020-001160-28/ES
EUCTR2020-001333-13	390	Chloroquine		https://www.clinicaltrialsregister.eu/ctr-search/trial/2020-001333-13/FR
EUCTR2020-001209-22	391	Chloroquine		https://www.clinicaltrialsregister.eu/ctr-search/trial/2020-001209-22/GB
ChiCTR2000031735	392	NK cells	Clinical study for natural killer (NK) cells from umbilical cord blood in the treatment of novel coronavirus pneumonia (COVID-19)	http://www.chictr.org.cn/historyversionpuben.aspx?regno=ChiCTR2000031735
ChiCTR2000031809	393	Inactivated novel coronavirus vaccine (Vero cells)	A randomized, double-blind, placebo parallel-controlled phase I/II clinical trial for inactivated COVID-19 vaccine (Vero cells)	http://www.chictr.org.cn/historyversionpuben.aspx?regno=ChiCTR2000031809
NCT04334148	394	Chloroquine	Healthcare Worker Exposure Response and Outcomes of Hydroxychloroquine	https://ClinicalTrials.gov/show/NCT04334148
NCT04334265	395	TCM	Efficacy and Safety of Anluohuaxian in the Treatment of Rehabilitation Patients With Corona Virus Disease 2019	https://ClinicalTrials.gov/show/NCT04334265
NCT04334382	396	Chloroquine + other	Hydroxychloroquine vs. Azithromycin for Outpatients in Utah With COVID-19	https://ClinicalTrials.gov/show/NCT04334382
NCT04334460	397	BLD-2660	Safety and Antiviral Activity of BLD-2660 in COVID-19 Hospitalized Subjects	https://ClinicalTrials.gov/show/NCT04334460
NCT04334629	398	Ibuprofen	LIBA Trial in COVID-19	https://ClinicalTrials.gov/show/NCT04334629
NCT04334928	399	Chloroquine + other	Randomized Clinical Trial for the Prevention of SARS-CoV-2 Infection (COVID-19) in Healthcare Personnel	https://ClinicalTrials.gov/show/NCT04334928
NCT04334980	400	Bacterial medium bacTRL-Spike	Evaluating the Safety, Tolerability and Immunogenicity of bacTRL-Spike Vaccine for Prevention of COVID-19	https://ClinicalTrials.gov/show/NCT04334980
NCT04335071	401	Tocilizumab	Tocilizumab in the Treatment of Coronavirus Induced Disease (COVID-19)	https://ClinicalTrials.gov/show/NCT04335071
NCT04335136	402	rhACE2	Recombinant Human Angiotensin-converting Enzyme 2 (rhACE2) as a Treatment for Patients With COVID-19	https://ClinicalTrials.gov/show/NCT04335136
NCT04335305	403	Tocilizumab/Pembrolizumab	Checkpoint Blockade in COVID-19 Pandemic	https://ClinicalTrials.gov/show/NCT04335305
NCT04335552	404	Chloroquine / Azithromycin	Pragmatic Factorial Trial of Hydroxychloroquine, Azithromycin, or Both for Treatment of Severe SARS-CoV-2 Infection	https://ClinicalTrials.gov/show/NCT04335552
NCT04335786	405	Valsartan	Valsartan for Prevention of Acute Respiratory Distress Syndrome in Hospitalized Patients With SARS-COV-2 (COVID-19) Infection Disease	https://ClinicalTrials.gov/show/NCT04335786
NCT04336332	406	Chloroquine / Azithromycin	Randomized Comparison of Combination Azithromycin and Hydroxychloroquine vs. Hydroxychloroquine Alone for the Treatment of Confirmed COVID-19	https://ClinicalTrials.gov/show/NCT04336332
NCT04336462	407	Oxygen nebulizer	Hydrogen-Oxygen Generator With Nebulizer in the Improvement of Symptoms in Patients Infected With COVID-19	https://ClinicalTrials.gov/show/NCT04336462
NCT04336748	408	Chloroquine	HCQ for Primary Prophylaxis Against COVID19 in Health-care Workers	https://ClinicalTrials.gov/show/NCT04336748
NCT04336904	409	Favipiravir	Clinical Study To Evaluate The Performance And Safety Of Favipiravir in COVID-19	https://ClinicalTrials.gov/show/NCT04336904
NCT04338074	410	Tranexamic acid	TXA and Corona Virus 2019 (COVID19) in Outpatients	https://ClinicalTrials.gov/show/NCT04338074
NCT04338126	411	Tranexamic acid	Tranexamic Acid (TXA) and Corona Virus 2019 (COVID19) in Inpatients	https://ClinicalTrials.gov/show/NCT04338126
NCT04338698	412	Chloroquine + other	Hydroxychloroquine, Oseltamivir and Azithromycin for the Treatment of COVID-19 Infection: An RCT	https://ClinicalTrials.gov/show/NCT04338698
NCT04338802	413	Nintedanib	Efficacy and Safety of Nintedanib in the Treatment of Pulmonary Fibrosis in Patients With Moderate to Severe COVID -19	https://ClinicalTrials.gov/show/NCT04338802
NCT04338828	414	Nitric Oxide	Nitric Oxide Inhalation Therapy for COVID-19 Infections in the ED	https://ClinicalTrials.gov/show/NCT04338828
NCT04338906	415	Chloroquine + other	Combination Therapy With Camostat Mesilate + Hydroxychloroquine for COVID-19	https://ClinicalTrials.gov/show/NCT04338906
NCT04339660	416	Stem cells	Clinical Research of Human Mesenchymal Stem Cells in the Treatment of COVID-19 Pneumonia	https://ClinicalTrials.gov/show/NCT04339660
NCT04339816	417	Chloroquine / Azithromycin	Azithromycin Added to Hydrochloroquine in Patients Admitted to Intensive Care With COVID-19: Randomised Controlled Trial	https://ClinicalTrials.gov/show/NCT04339816
NCT04340349	418	Chloroquine	Low-dose Hydroxychloroquine and Bromhexine: a Novel Regimen for COVID-19 Prophylaxis in Healthcare Professionals	https://ClinicalTrials.gov/show/NCT04340349
NCT04340544	419	Chloroquine	Hydroxychloroquine for the Treatment of Mild COVID-19 Disease	https://ClinicalTrials.gov/show/NCT04340544
NCT04340557	420	Losartan	Do Angiotensin Receptor Blockers Mitigate Progression to Acute Respiratory Distress Syndrome With SARS-CoV-2 Infection	https://ClinicalTrials.gov/show/NCT04340557
NCT04341116	421	TJ003234	Study of TJ003234 (Anti-GM-CSF Monoclonal Antibody) in Subjects With Severe Coronavirus Disease 2019 (COVID-19)	https://ClinicalTrials.gov/show/NCT04341116
NCT04341285	422	ECMO	Early Versus Late ECMO Therapy in COVID-19 Induced ARDS (ECMO-VID)	https://ClinicalTrials.gov/show/NCT04341285
NCT04341389	423	Vaccine Adenovirus	A Phase II Clinical Trial to Evaluate the Recombinant Novel Coronavirus Vaccine (Adenovirus Vector)	https://ClinicalTrials.gov/show/NCT04341389
NCT04341441	424	Chloroquine	Will Hydroxychloroquine Impede or Prevent COVID-19	https://ClinicalTrials.gov/show/NCT04341441
NCT04341584	425	Anakinra	CORIMUNO-ANA: Trial Evaluating Efficacy Of Anakinra In Patients With Covid-19 Infection	https://ClinicalTrials.gov/show/NCT04341584
NCT04341675	426	Sirolimus	Sirolimus Treatment in Hospitalized Patients With COVID-19 Pneumonia	https://ClinicalTrials.gov/show/NCT04341675
NCT04341727	427	Chloroquine / Azithromycin	Hydroxychloroquine,Hydroxychloroquine,Azithromycin in the Treatment of SARS CoV-2 Infection	https://ClinicalTrials.gov/show/NCT04341727
NCT04341870	428	Chloroquine + other	Study of Immune Modulatory Drugs and Other Treatments in COVID-19 Patients: Sarilumab, Azithromycin, Hydroxychloroquine Trial - CORIMUNO-19 - VIRO	https://ClinicalTrials.gov/show/NCT04341870
NCT04341935	429	Linagliptin/insulin	Effects of DPP4 Inhibition on COVID-19	https://ClinicalTrials.gov/show/NCT04341935
NCT04342156	430	Chloroquine	Safety And Efficacy Of Hydroxychloroquine As COVID-19 Prophylaxis For At-Risk Population (SHARP): A Cluster Randomized Controlled Trial	https://ClinicalTrials.gov/show/NCT04342156
NCT04342169	431	Chloroquine	University of Utah COVID-19 Hydrochloroquine Trial	https://ClinicalTrials.gov/show/NCT04342169
NCT04342221	432	Chloroquine	Hydroxychloroquine for COVID-19	https://ClinicalTrials.gov/show/NCT04342221
NCT04342650	433	Chloroquine	Chloroquine Diphosphate in the Prevention of SARS in Covid-19 Infection	https://ClinicalTrials.gov/show/NCT04342650
NCT04342663	434	Fluvoxamine	A Double-blind, Placebo-controlled Clinical Trial of Fluvoxamine for Symptomatic Individuals With COVID-19 Infection	https://ClinicalTrials.gov/show/NCT04342663
NCT04342689	435	Bobs Red Mill	The Role of Resistant Potato Starch in COVID-19 Infection	https://ClinicalTrials.gov/show/NCT04342689
NCT04342897	436	LY3127804	A Study of LY3127804 in Participants With COVID-19	https://ClinicalTrials.gov/show/NCT04342897
NCT04343001	437	Losartan/aspirin/simvastatin	Coronavirus Response - Active Support for Hospitalised Covid-19 Patients	https://ClinicalTrials.gov/show/NCT04343001
NCT04343092	438	Ivermectin	Ivermectin Adjuvant to Hydroxychloroquin in COVID19 Patients	https://ClinicalTrials.gov/show/NCT04343092
NCT04343144	439	Nivolumab	Trial Evaluating Efficacy and Safety of Nivolumab (Optivo¬Æ) in Patients With COVID-19 Infection, Nested in the Corimmuno-19 Cohort.	https://ClinicalTrials.gov/show/NCT04343144
NCT04343248	440	Nitazoxanide	A Randomized, Double-Blind, Placebo Controlled, Trial to Evaluate the Efficacy and Safety of Nitazoxanide (NTZ) for Post-Exposure Prophylaxis of COVID-19 and Other Viral Respiratory Illnesses in Elderly Residents of Long-Term Care Facilities (LTCF)	https://ClinicalTrials.gov/show/NCT04343248
NCT04343651	441	Leronlimab	Study to Evaluate the Efficacy and Safety of Leronlimab for Mild to Moderate COVID-19	https://ClinicalTrials.gov/show/NCT04343651
NCT04343677	442	Chloroquine	Military COVID-19 Hydroxychloroquine Pre-exposure and Post-exposure Prophylaxis Study	https://ClinicalTrials.gov/show/NCT04343677
NCT04343729	443	Glucocorticoid	Methylprednisolone in the Treatment of Patients With Signs of Severe Acute Respiratory Syndrome in Covid-19	https://ClinicalTrials.gov/show/NCT04343729
NCT04343768	444	Chloroquine + other	An Investigation Into Beneficial Effects of Interferon Beta 1a, Compared to Interferon Beta 1b And The Base Therapeutic Regiment in Moderate to Severe COVID-19: A Randomized Clinical Trial	https://ClinicalTrials.gov/show/NCT04343768
NCT04343794	445	Biovitals	Using Biovitals¬Æ Sentinel to Monitor Disease Progression in Subjects Quarantined for Suspected COVID-19	https://ClinicalTrials.gov/show/NCT04343794
NCT04343963	446	Pyridostigmine	Pyridostigmine in Severe SARS-CoV-2 Infection	https://ClinicalTrials.gov/show/NCT04343963
NCT04343976	447	INF-Lambda	Pegylated Interferon Lambda Treatment for COVID-19	https://ClinicalTrials.gov/show/NCT04343976
NCT04343989	448	Clazakizumab	A Randomized Placebo-controlled Safety and Dose-finding Study for the Use of the IL-6 Inhibitor Clazakizumab in Patients With Life-threatening COVID-19 Infection	https://ClinicalTrials.gov/show/NCT04343989

Registry entry ID	Included ID	Intervention	Title	Webpage
NCT04344041	449	Vitamin D	COvid-19 and Vitamin D Supplementation: a Multicenter Randomized Controlled Trial of High Dose Versus Standard Dose Vitamin D3 in High-risk COVID-19 Patients (CoVitTrial)	https://ClinicalTrials.gov/show/NCT04344041
NCT04344080	450	Cyto-sorb	Effect of CytoSorb Adsorber on Hemodynamic and Immunological Parameters in Critical Ill Patients With COVID-19	https://ClinicalTrials.gov/show/NCT04344080
NCT04344184	451	Vitamin C	Early Infusion of Vitamin C for Treatment of Novel COVID-19 Acute Lung Injury (EVICT-CORONA-ALI)	https://ClinicalTrials.gov/show/NCT04344184
NCT04344288	452	Prednisolone	Corticosteroids During Covid-19 Viral Pneumonia Related to SARS-Cov-2 Infection	https://ClinicalTrials.gov/show/NCT04344288
NCT04344444	453	Chloroquine / Azithromycin	Treatment in Patients With Suspected or Confirmed COVID-19 With Early Moderate or Severe Disease	https://ClinicalTrials.gov/show/NCT04344444
NCT04344535	454	Convalescent Plasma	Convalescent Plasma vs. Standard Plasma for COVID-19	https://ClinicalTrials.gov/show/NCT04344535
NCT04344561	455	Prone position	Incline Positioning in COVID-19 Patients for Improvement in Oxygen Saturation	https://ClinicalTrials.gov/show/NCT04344561
NCT04344730	456	Dexamethasone/CPAP/Mechanical ventilation	Dexamethasone and Oxygen Support Strategies in ICU Patients With Covid-19 Pneumonia	https://ClinicalTrials.gov/show/NCT04344730
NCT04344756	457	Tinzaparin/heparin	Trial Evaluating Efficacy and Safety of Anticoagulation in Patients With COVID-19 Infection, Nested in the Corimmuno-19 Cohort	https://ClinicalTrials.gov/show/NCT04344756
NCT04344782	458	Bevacizumab	Trial Evaluating Efficacy and Safety of Bevacizumab (Avastin¬Æ/Zeribev¬Æ) in Patients With COVID-19 Infection, Nested in the Corimmuno-19 Cohort	https://ClinicalTrials.gov/show/NCT04344782
NCT04345289	459	Chloroquine + other	Efficacy and Safety of Novel Treatment Options for Adults With COVID-19 Pneumonia	https://ClinicalTrials.gov/show/NCT04345289
NCT04345406	460	ACE inhibitor	Angiotensin Converting Enzyme Inhibitors in Treatment of Covid 19	https://ClinicalTrials.gov/show/NCT04345406
NCT04345445	461	Methylprednisolone/tocilizumab	Study to Evaluate the Efficacy and Safety of Tocilizumab Versus Corticosteroids in Hospitalised COVID-19 Patients With High Risk of Progression	https://ClinicalTrials.gov/show/NCT04345445
NCT04345523	462	Transfusion	Convalescent Plasma Therapy vs. SOC for the Treatment of COVID19 in Hospitalized Patients	https://ClinicalTrials.gov/show/NCT04345523
NCT04345614	463	Calcium-channel inhibitor	A Study of CM4620-Injectable Emulsion (IE) in Patients With Severe COVID-19 Pneumonia	https://ClinicalTrials.gov/show/NCT04345614
NCT04345692	464	Chloroquine	A Randomized Controlled Clinical Trial: Hydroxychloroquine for the Treatment of COVID-19 in Hospitalized Patients	https://ClinicalTrials.gov/show/NCT04345692
TCTR20200409006	465	HA330	Efficacy of HA330 Hemoperfusion in Critically Ill Patients with Severe COVID-19 (HA-COVID19)	https://www.clinicaltrials.in.th/index.php?tp=regtrials&menu=trialsearch&smenu=fulltext&task=search&task2=view1&id=6016
TCTR20200404004	466	Chloroquine + other	Comparative effectiveness of Chloroquine and Vitamin C prophylaxis in household contacts of confirmed COVID-19 patients (ProphylaxisCOVID19)	https://www.clinicaltrials.in.th/index.php?tp=regtrials&menu=trialsearch&smenu=fulltext&task=search&task2=view1&id=5995
jRCTs031190269	467	Ciclesonide	A multicenter, open-label, randomized controlled trial to evaluate the efficacy and safety of inhaled ciclesonide for asymptomatic and mild patients with COVID-19	https://rctportal.niph.go.jp/en/detail?trial_id=jRCTs031190269
RBR-3cbs3w	468	Chloroquine	Evaluation of Hydroxychloroquine for prevention of hospitalization and respiratory complications in patients with confirmed diagnosis or suspected infection by (COVID-19)	http://www.ensaiosclinicos.gov.br/rg/RBR-3cbs3w/
ACTRN12620000457943p	469	Chloroquine	Hydroxychloroquine for the Community-Based Treatment of COVID-19	https://www.anzctr.org.au/Trial/Registration/TrialReview.aspx?id=379574&isReview=true
ACTRN12620000454976	470	Zinc	High-dose intravenous zinc (HDIVZn) as adjunctive therapy in COVID-19 positive critically ill patients: A pilot randomized controlled trial	https://www.anzctr.org.au/Trial/Registration/TrialReview.aspx?id=379579&isReview=true
NCT04350320	471	Colchicine	Randomized Open-blind Controlled Trial to Study the Benefit of Colchicine in Patients With COVID-19	https://clinicaltrials.gov/show/NCT04350320
NCT04345991	472	Convalescent Plasma	Efficacy of Convalescent Plasma to Treat COVID-19 Patients, a Nested Trial in the CORIMUNO-19 Cohort	https://clinicaltrials.gov/show/NCT04345991
NCT04346147	473	Chloroquine vs Lopinavir/ritonavir vs Imatinib tablets vs Baricitinib	Clinical Trial to Evaluate Efficacy of 3 Types of Treatment in Patients With Pneumonia by COVID-19	https://clinicaltrials.gov/show/NCT04346147
NCT04346199	474	Acalabrutinib	Acalabrutinib Study With Best Supportive Care Versus Best Supportive Care in Subjects Hospitalized With COVID-19. CALAVI (Calquence Against the Virus)	https://clinicaltrials.gov/show/NCT04346199
NCT04346329	475	Chloroquine	Immune Monitoring of Prophylactic Effect of Hydroxychloroquine in Healthcare Providers Highly Exposed to COVID-19	https://clinicaltrials.gov/show/NCT04346329
NCT04346446	476	Convalescent Plasma	Efficacy of Convalescent Plasma Therapy in Severely Sick COVID-19 Patients	https://clinicaltrials.gov/show/NCT04346446
NCT04346615	477	Vazegepant	Safety and Efficacy Trial of Vazegepant Intranasal for Hospitalized Patients With COVID-19 Requiring Supplemental Oxygen	https://clinicaltrials.gov/show/NCT04346615
NCT04346628	478	Favipiravir	Oral Favipiravir Compared to Standard Supportive Care in Subjects With Mild COVID-19	https://clinicaltrials.gov/show/NCT04346628
NCT04346667	479	Chloroquine	Post-Exposure Prophylaxis for Asymptomatic SARS-CoV-2 COVID-19 Patients With choloroquinE Compounds	https://clinicaltrials.gov/show/NCT04346667
NCT04346693	480	Dalargin	An Open Randomized Study of Dalargin Effectiveness in Patients With Severe and Critical Manifestations of SARS-COVID-19	https://clinicaltrials.gov/show/NCT04346693
NCT04346797	481	Eculizumab	CORIMUNO19-ECU: Trial Evaluating Efficacy and Safety of Eculizumab (Soliris) in Patients With COVID-19 Infection, Nested in the CORIMUNO-19 Cohort	https://clinicaltrials.gov/show/NCT04346797
NCT04347174	482	Vaccine Mycobacterium w	A Clinical Trial of Mycobacterium w in Critically Ill COVID 19 Patients	https://clinicaltrials.gov/show/NCT04347174
NCT04347226	483	Anti-IL-8	Anti-Interleukin-8 (Anti-IL-8) for Cancer Patients With COVID-19	https://clinicaltrials.gov/show/NCT04347226
NCT04347239	484	Leronlimab	Study to Evaluate the Efficacy and Safety of Leronlimab for Patients With Severe or Critical Coronavirus Disease 2019 (COVID-19)	https://clinicaltrials.gov/show/NCT04347239
NCT04347382	485	Nigella Sativa and Natural Honey	Efficacy of Nigella Sativa and Natural Honey Against COVID-19: an RCT	https://clinicaltrials.gov/show/NCT04347382
NCT04347889	486	Chloroquine vs Vitamin C	Preventing COVID-19 in Healthcare Workers With HCQ: A RCT	https://clinicaltrials.gov/show/NCT04347889
NCT04347915	487	Chloroquine / Clevudine	The Phase 2 Study to Evaluate the Safety and Efficacy of Clevudine in Patients With Moderate COVID-19	https://clinicaltrials.gov/show/NCT04347915
NCT04347941	488	Prone position	Awake Prone Positioning to Reduce Invasive VEntilation in COVID-19 Induced Acute Respiratory failurE	https://clinicaltrials.gov/show/NCT04347941
NCT04347954	489	Povidone Iodine	PVP-I Nasal Sprays and SARS-CoV-2 Nasopharyngeal Titers (for COVID-19)	https://clinicaltrials.gov/show/NCT04347954
NCT04348383	490	Defibrotide	Defibrotide as Prevention and Treatment of Respiratory Distress and Cytokine Release Syndrome of Covid 19.	https://clinicaltrials.gov/show/NCT04348383
NCT04348409	491	Nitazoxanide	Efficacy and Safety of Nitazoxanide for the Treatment of Hospitalized Patients With Moderate COVID-19	https://clinicaltrials.gov/show/NCT04348409
NCT04348435	492	Stem cells	Study of Allogeneic HB-adMSCs to Provide Immune Support Against COVID-19	https://clinicaltrials.gov/show/NCT04348435
NCT04348656	493	Convalescent Plasma	CONvalescent Plasma for Hospitalized Adults With COVID-19 Respiratory Illness (CONCOR-1)	https://clinicaltrials.gov/show/NCT04348656
NCT04348695	494	Ruxolitinib / Simvastatin	Study of Ruxolitinib Plus Simvastatin in the Prevention and Treatment of Respiratory Failure of COVID-19.	https://clinicaltrials.gov/show/NCT04348695
NCT04349241	495	Favipiravir	Efficacy and Safety of Favipiravir in Management of COVID-19	https://clinicaltrials.gov/show/NCT04349241
NCT04349371	496	Chloroquine	Saved From COVID-19	https://clinicaltrials.gov/show/NCT04349371
NCT04349618	497	Protective ventilation	Ultraprotective Ventilation Without Extracorporeal Circulation During COVID 19 Pneumonia	https://clinicaltrials.gov/show/NCT04349618
NCT04350281	498	Chloroquine / IFN-B	Double Therapy With IFN-beta 1b and Hydroxychloroquine	https://clinicaltrials.gov/show/NCT04350281
NCT04350593	499	Dapagliflozin	Dapagliflozin in Respiratory Failure in Patients With COVID-19	https://clinicaltrials.gov/show/NCT04350593
NCT04350931	500	BCG vaccine	Application of BCG Vaccine for Immune-prophylaxis Among Egyptian Healthcare Workers During the Pandemic of COVID-19	https://clinicaltrials.gov/show/NCT04350931
NCT04351152	501	Lenzilumab	Phase 3 Study to Evaluate Efficacy and Safety of Lenzilumab in Hospitalized Patients With COVID-19 Pneumonia	https://clinicaltrials.gov/show/NCT04351152
NCT04351295	502	Favipiravir	Efficacy of Faviprevir in COVID-19 Treatment	https://clinicaltrials.gov/show/NCT04351295
NCT04351347	503	Chloroquine / Nitazoxanide / Ivermectin	The Efficacy of Ivermectin and Nitazoxanide in COVID-19 Treatment	https://clinicaltrials.gov/show/NCT04351347
NCT04351724	504	Chloroquine / Lopinavir/Ritonavir / Rivaroxaban / Candesartan / non-RAS blocking	Austrian CoronaVirus Adaptive Clinical Trial	https://clinicaltrials.gov/show/NCT04351724
ACTRN12620000473965	505	Broncho-Vaxom	Reducing acute severe respiratory events in health care workers during the Covid-19 pandemic with OM85	https://anzctr.org.au/ACTRN12620000473965.aspx
ACTRN12620000478910	506	Stem cells	Cord Blood Therapy to prevent progression of COVID-19 related pneumonia	https://anzctr.org.au/ACTRN12620000478910.aspx
ChiCTR2000031944	507	Chinese Herbal Tea	Efficacy of Chinese Herbal Tea in the Prevention of Novel Coronavirus Pneumonia (COVID-19): a Randomized Controlled Trial	http://www.chictr.org.cn/showproj.aspx?proj=52414
ChiCTR2000032011	508	Hyperbaric Oxygen	Effecacy and Safety of Hyperbaric Oxygen Therapy to patients with novel coronavirus pneumonia (COVID-19)	http://www.chictr.org.cn/showproj.aspx?proj=52142
EUCTR2019-002688-89-ES	509	Stem cells	Clinical study to assess the safety and preliminary efficacy of HCR040, a drug based on mesenchymal stem cells, in patients with acute respiratory distress syndrome. (included patients COVID-19)	https://www.clinicaltrialsregister.eu/ctr-search/search?query=eudract_number:2019-002688-89
EUCTR2020-001257-51-DK	510	Chloroquine	Hydroxychloroquine for prevention of COVID-19 in patients on chronic dialysis	https://www.clinicaltrialsregister.eu/ctr-search/search?query=eudract_number:2020-001257-51
EUCTR2020-001266-11-ES	511	Stem cells	Clinical trial of administration of MSC to patients with respiratory distress type COVID-19	https://www.clinicaltrialsregister.eu/ctr-search/search?query=eudract_number:2020-001266-11
EUCTR2020-001406-27-FR	512	Azithromycin	efficacy and safety of a bitherapy by hydroxychloroquine + azythromycin vs monotherapy by hydroxychoroquine in adult patients with documentated infection by covid19	https://www.clinicaltrialsregister.eu/ctr-search/search?query=eudract_number:2020-001406-27
EUCTR2020-001420-34-DK	513	Senicapoc	Senicapoc treatment of COVID-19 positive patients in intensive care	https://www.clinicaltrialsregister.eu/ctr-search/search?query=eudract_number:2020-001420-34
EUCTR2020-001437-12-ES	514	Ciclosporin /Tocilizumab	Random, controlled, open, one-site clinical trial in adult patients with COVID-19 severe pneumonia treated with immunomodulatory drugs	https://www.clinicaltrialsregister.eu/ctr-search/search?query=eudract_number:2020-001437-12
EUCTR2020-001570-30-FR	515	Clairyg	ICAR (IgIV in Covid-related ARds)	https://www.clinicaltrialsregister.eu/ctr-search/search?query=eudract_number:2020-001570-30
EUCTR2020-001587-29-ES	516	Chloroquine	Preventing SARS-CoV-2 virus infection and severity of COVID-19 diseases during pregnancy with hydroxychloroquine	https://www.clinicaltrialsregister.eu/ctr-search/search?query=eudract_number:2020-001587-29
EUCTR2020-001682-36-ES	517	Stem cells	Treatment of COVID-19 with allogeneic mesenchymal cells (MSV¬Æ).	https://www.clinicaltrialsregister.eu/ctr-search/search?query=eudract_number:2020-001682-36
EUCTR2020-001689-12-ES	518	Colchicine	COLCHICINA TEST IN THE SARS-COV2 CORONAVIRUS (COLCORONA-COVID-19)	https://www.clinicaltrialsregister.eu/ctr-search/search?query=eudract_number:2020-001689-12
IRCT20080901001165N46	519	Umifenovir	Investigating the efficacy and safety of Umifenovir in controlling the symptoms of patients with COVID-19	http://en.irct.ir/trial/47087
IRCT20130306012728N8	520	Chloroquine	Post-exposure hydroxycloroquine prophylaxis for Covid 19	http://en.irct.ir/trial/46932
IRCT20160313027033N2	521	MCT Oil	The effect of MCT Oil on COVID-19	http://en.irct.ir/trial/46762
IRCT20180725040596N2	522	Umifenovir	Effect of Arbidol in treatment of COVID-19	http://en.irct.ir/trial/46897
IRCT20190122042450N4	523	Chloroquine	The effect of hydroxychloroquine to prevent coronavirus disease	http://en.irct.ir/trial/47090
IRCT20191125045492N2	524	ozone autohemotherapy	Effect of Ozone therapy in treatment of COVID-19	http://en.irct.ir/trial/47149
IRCT20200217046526N2	525	Stem cells	Mesenchymal Stem Cell Therapy for Acute Respiratory Distress Syndrome in Coronavirus Infection	http://en.irct.ir/trial/47073
IRCT20200314046764N1	526	Pirfenidone	Pirfenidine in SARS-COV2	http://en.irct.ir/trial/46533
IRCT20200317046797N4	527	Bromhexine	Effect of Bromhexine Hydrochloride on patients with COVID-19	http://en.irct.ir/trial/46969
IRCT20200404046935N1	528	Herbal medicine	Effect of herbal medicine containing Saatar, Hofarigon and Fennel on COVID-19 patients	http://en.irct.ir/trial/46940
IRCT20200404046937N1	529	Marshmallow and Licorice	Evaluating the effect of Marshmallow and Licorice on COVID 19 patients	http://en.irct.ir/trial/46903
IRCT20200404046937N2	530	Phyllanthus Emblica	Evaluating the effect of Phyllanthus Emblica on COVID 19 patients	http://en.irct.ir/trial/47016
IRCT20200404046948N1	531	Convalescent Plasma	Efficacy and safety of convalescent plasma in the treatment of COVID-19	http://en.irct.ir/trial/46973
IRCT20200405046958N1	532	Chloroquine	Prevention of Novel Coronavirus Disease by Hydroxychloroquine in cancer patients.	http://en.irct.ir/trial/46946
IRCT20200408046992N1	533	Trifluoperazine	Effect of Trifluoperazine in treatment of COVID-19	http://en.irct.ir/trial/47006
IRCT20200411047025N1	534	Vitamin C	Effect of Intravenous vitamin c in Patients with COVID-19	http://en.irct.ir/trial/47078
IRCT20200413047053N1	535	Asafoetida	Evaluation the effect of asafoetida in the treatment of coronavirus infection	http://en.irct.ir/trial/47139
IRCT20200413047056N1	536	Immunoglobulin and plasma	Comparison between the efficacy of intravenous immunoglobulin and convalescent plasma in COVID-19	http://en.irct.ir/trial/47212
IRCT20200415047089N1	537	Herbal medicine	The effect of herbal drugs (Echinacea and Ginger) on Improvement of clinical symptoms and hospitalizations in suspected COVID-19 outpatient cases	http://en.irct.ir/trial/47234
IRCT20200415047092N1	538	Azithromycin	The effect of azithromycin in treatment of COVID19	http://en.irct.ir/trial/47244

Registry entry ID	Included ID	Intervention	Title	Webpage
ISRCTN14326006	539	Chloroquine	Does taking hydroxychloroquine before and during exposure to patients protect frontline healthcare workers from coronavirus?	http://isrctn.com/ISRCTN14326006
JPRN-JapicCTI-205238	540	Favipiravir	Phase 3 Clinical Study of Favipiravir in Patients with COVID-19 Non-Severe Pneumonia	https://www.clinicaltrials.jp/user/showCteDetailE.jsp?japicId=JapicCTI-205238
JPRN-jRCT2031190264	541	Remdesivir	A Multicenter, Adaptive, Randomized Blinded Controlled Trial of the Safety and Efficacy of Investigational Therapeutics for the Treatment of COVID-19 in Hospitalized Adults	https://jrct.niph.go.jp/latest-detail/jRCT2031190264
NCT04345848	542	Enoxaparin	Preventing COVID-19 Complications With Low- and High-dose Anticoagulation	https://clinicaltrials.gov/show/NCT04345848
NCT04345887	543	Spironolactone	Spironolactone in Covid-19 Induced ARDS	https://clinicaltrials.gov/show/NCT04345887
NCT04346368	544	Stem cells	Bone Marrow-Derived Mesenchymal Stem Cell Treatment for Severe Patients With Coronavirus Disease 2019 (COVID-19)	https://clinicaltrials.gov/show/NCT04346368
NCT04347980	545	Dexamethasone	Dexamethasone Treatment for Severe Acute Respiratory Distress Syndrome Induced by COVID-19	https://clinicaltrials.gov/show/NCT04347980
NCT04348305	546	Hydrocortisone	Hydrocortisone for COVID-19 and Severe Hypoxia	https://clinicaltrials.gov/show/NCT04348305
NCT04348461	547	Stem cells	BAttLe Against COVID-19 Using MesenchYmal Stromal Cells	https://clinicaltrials.gov/show/NCT04348461
NCT04348500	548	Clazakizumab	Clazakizumab (Anti-IL- 6 Monoclonal) Compared to Placebo for COVID19 Disease	https://clinicaltrials.gov/show/NCT04348500
NCT04348513	549	Triiodothyronine	Triiodothyronine for the Treatment of Critically Ill Patients With COVID-19 Infection	https://clinicaltrials.gov/show/NCT04348513
NCT04349098	550	Selinexor	Evaluation of Activity and Safety of Oral Selinexor in Participants With Severe COVID-19 Infection	https://clinicaltrials.gov/show/NCT04349098
NCT04349410	551	Chloroquine, Azithromycin, Clindamycin, Primaquine, Remdesivir, Tocilizumab,	The Fleming [FMTVDM] Directed CoVid-19 Treatment Protocol	https://clinicaltrials.gov/show/NCT04349410
NCT04350671	552	INF-Beta	Interferon Beta 1a in COVID-19: A Randomized, Double-Blind, Placebo-Controlled, Clinical Trial	https://clinicaltrials.gov/show/NCT04350671
NCT04350684	553	Umifenovir Interferon-a Lopinavir/Ritonavir Chloroquine SoC	Umifenovir in COVID-19: A Randomized, Double-Blind, Placebo-Controlled, Clinical Trial	https://clinicaltrials.gov/show/NCT04350684
NCT04351191	554	Chloroquine	PRophylaxis of Exposed COVID-19 Individuals With Mild Symptoms Using choloroquinE Compounds	https://clinicaltrials.gov/show/NCT04351191
NCT04351581	555	Discontinuing ACEi/ARB	Effects of Discontinuing Renin-angiotensin System Inhibitors in Patients With COVID-19	https://clinicaltrials.gov/show/NCT04351581
NCT04351763	556	Amiodarone or Verapamil	Amiodarone or Verapamil in COVID-19 Hospitalized Patients With Symptoms	https://clinicaltrials.gov/show/NCT04351763
PACTR202004693933092	557	Measles Vaccine	Effectiveness of Measles Vaccine in Health Care Professionals During COVID-19 Outbreak (Randomized Controlled Trial)	https://pactr.samrc.ac.za/TrialDisplay.aspx?TrialID=10953
PACTR202004761408382	558	IHP Detox Tea	Efficacy and safety of IHP Detox Tea (a special blend of Andrographis paniculata, Garcinia kola and Psidium guajava) for treatment of Corona virus disease 2019 (COVID-19): a pilot placebo-controlled randomized trial	https://pactr.samrc.ac.za/TrialDisplay.aspx?TrialID=10957
PACTR202004801273802	559	Chloroquine	Lagos COVID-19 Chloroquine Treatment Trial (LACCTT)	https://pactr.samrc.ac.za/TrialDisplay.aspx?TrialID=10928
PER-010-20	560	Chloroquine / Lopinavir/ritonavir / INF-B (SOLIDARITY)	SOLIDARITY	https://www.ins.gob.pe/ensayosclinicos/rpec/recuperarECPBNuevoEN.asp?numec=010-20
RBR-8969zg	561	N-Acetyl Cysteine	Treatment of 2019-nCoV Pneumonia with N-acetylcysteine	http://www.ensaiosclinicos.gov.br/rg/RBR-8969zg/
RPCEC00000306	562	Vaccine CIGB 2020	CIGB 2020 in contacts and SARS-CoV-2 infection suspects	http://rpcec.sld.cu/en/trials/RPCEC00000306-En
RPCEC00000307	563	HeberFERON versus Heberon alfa	Evaluation of the effect and safety of HeberFERON versus Heberon alfa in patients infected with the SARS-CoV-2 coronavirus.	http://registroclinico.sld.cu/en/trials/RPCEC00000307-En
ISRCTN13311119	564	Siddha treatment	START (Siddha Treatment Accelerating Recovery from SARS-CoV-2 Test) trial of Siddha treatment for patients with novel coronavirus infectious disease (COVID-19)	https://www.isrctn.com/ISRCTN13311119?q=COVID-19&filters=&sort=&offset=1&totalResults=20&page=1
CTRI/2020/04/024806	565	Imatinib	Efficacy of Imatinib in mild SARS CoV2 infection	
CTRI/2020/04/024729	566	Chloroquine	Topical Chloroquine Nasal Drops in Early Stage Covid 19- Impact on Viral load and cure rates	
CTRI/2020/04/024749	567	BCG vaccine	Study to Evaluate the Efficacy of Recombinant BCG VPM1002 in Reducing Infection Incidence and Disease Severity of SARS-COV-2/COVID-19 Among High-Risk	
CTRI/2020/04/024833	568	BCG vaccine	BCG-Denmark versus no-BCG for COVID 19 prevention	
CTRI/2020/04/02477	569	Convalescent Plasma	Study to assess the efficacy and safety of convalescent plasma in moderate COVID-19 disease	
CTRI/2020/04/024883	570	Ayurveda	Clinical research on safety and efficacy of ZingiVir-H as an add on therapy in COVID-19 patients.	
EUCTR2020-001618-39	571	vafidemstat	A Phase II, randomized, open–label study to evaluate the efficacy and tolerability of treatment with vafidemstat in combination with standard of care treatment to prevent Acute Respiratory Distress Syndrome (ARDS) in adult severely ill patients with COVID-19.	https://www.clinicaltrialsregister.eu/ctr-search/trial/2020-001618-39/ES
ACTRN12620000501943	572	Chloroquine	COVID-19 prophylaxis with hydroxychloroquine in Front-line Health and Allied-Health Care Workers: The COVID-SHIELD Trial	https://anzctr.org.au/ACTRN12620000501943.aspx
ACTRN12620000517976	573	Heparin	COVID-19: Can Nebulised HepArin Reduce Time to Extubation in SARS-CoV-2 patients requiring mechanical ventilation (CHARTER Study)	https://anzctr.org.au/ACTRN12620000517976.aspx
ChiCTR2000032135	574	Ulinastatin	Efficacy and Safety of ulinastatin in the Treatment of novel coronavirus pneumonia (COVID-19)	http://www.chictr.org.cn/showproj.aspx?proj=52506
ChiCTR2000032242	575	Kolimycin	A multicenter, randomized, open, controlled trial for the efficacy and safety of oral kolimycin in the treatment of patients with new coronavirus pneumonia (CoVID-19)	http://www.chictr.org.cn/showproj.aspx?proj=52824
ChiCTR2000032314	576	Aliskiren and Nifedipine	A randomized clinical trial for the efficacy and safety of Aliskiren and Nifedipine in novel coronavirus pneumonia (COVID-19) patients with hypertension	http://www.chictr.org.cn/showproj.aspx?proj=52867
ChiCTR2000032367	577	TCM	Efficacy of Liu-zi-jue in Patients with 2019 Novel Coronavirus Pneumonia (COVID-19): a randomized controlled trial	http://www.chictr.org.cn/showproj.aspx?proj=52972
ChiCTR2000032459	578	Inactivated novel coronavirus vaccine (Vero cells)	A phase I/II clinical trial for inactivated novel coronavirus (2019-CoV) vaccine (Vero cells)	http://www.chictr.org.cn/historyversionpuben.aspx?regno=ChiCTR2000032459
EUCTR2020-001072-15-GB	579	Vaccine COV001	A phase I/II to determine efficacy, safety and immunogenicity of a candidate COVID-19 vaccine	https://www.clinicaltrialsregister.eu/ctr-search/search?query=eudract_number:2020-001072-15
EUCTR2020-001244-26-AT	580	Solnatide	COVID-19: Efficacy of solnatide to treat pulmonary permeability oedema in SARS-Cov-2 positive patients with moderate-to-severe ARDS	https://www.clinicaltrialsregister.eu/ctr-search/search?query=eudract_number:2020-001244-26
EUCTR2020-001375-32-NL	581	Tocilizumab	Early intervention with Tocilizumab in patients with COVID-19 and hypoxia	https://www.clinicaltrialsregister.eu/ctr-search/search?query=eudract_number:2020-001375-32
EUCTR2020-001391-15-IE	582	Plasma-purified alpha-1 antitrypsin	A randomized double-blind placebo-controlled trial of intravenous plasma-purified alpha-1 antitrypsin for severe COVID-19 illness.	https://www.clinicaltrialsregister.eu/ctr-search/search?query=eudract_number:2020-001391-15
EUCTR2020-001408-41-DE	583	Tocilizumab	Evaluation of efficacy and safety of an tocilizumab treatment in patients with severe COVID-19 pneumonia	https://www.clinicaltrialsregister.eu/ctr-search/search?query=eudract_number:2020-001408-41
EUCTR2020-001417-21-BE	584	Chloroquine	An open label single center randomized controlled trial to evaluate the effect of hydroxychloroquine on viral shedding in mild COVID-19	https://www.clinicaltrialsregister.eu/ctr-search/search?query=eudract_number:2020-001417-21
EUCTR2020-001440-26-ES	585	Chloroquine	Study for the prevention of COVID-19 infection in healthcare personnel	https://www.clinicaltrialsregister.eu/ctr-search/search?query=eudract_number:2020-001440-26
EUCTR2020-001527-14-NL	586	Azithromycin	COVID-19: addition of azithromycin to chloroquine treatment	https://www.clinicaltrialsregister.eu/ctr-search/search?query=eudract_number:2020-001527-14
EUCTR2020-001591-15-NL	587	BCG vaccine	Reducing hospital admission of elderly in Coronavirus pandemic by boosting the immune system through vaccination with bacillus Calmette-Gu√©rin (BCG).	https://www.clinicaltrialsregister.eu/ctr-search/search?query=eudract_number:2020-001591-15
EUCTR2020-001614-38-BE	588	Azithromycin	Covid-19: A randomized, open-label, adaptive, proof-of- concept clinical trial of new antiviral drug candidates against SARS-CoV-2.	https://www.clinicaltrialsregister.eu/ctr-search/search?query=eudract_number:2020-001614-38
EUCTR2020-001622-64-ES	589	Prednisolone	OUTPATIENT TREATMENT OF COVID-19 WITH EARLY PULMONARY CORTICOSTEROIDS AS AN OPPORTUNITY TO MODIFY THE COURSE OF THE DISEASE	https://www.clinicaltrialsregister.eu/ctr-search/search?query=eudract_number:2020-001622-64
EUCTR2020-001659-42-ES	590	Antithrombin	Study of antithrombin as prophylaxis of acute respiratory disease in patients with COVID-19	https://www.clinicaltrialsregister.eu/ctr-search/search?query=eudract_number:2020-001659-42
EUCTR2020-001662-11-DE	591	Ruxolitinib	RUXCOVID	https://www.clinicaltrialsregister.eu/ctr-search/search?query=eudract_number:2020-001662-11
EUCTR2020-001770-30-BE	592	Tocilizumab	Experimental use of tocilizumab in COVID-19 related pneumonia.	https://www.clinicaltrialsregister.eu/ctr-search/search?query=eudract_number:2020-001770-30
NCT04347031	593	Mefloquine		https://ClinicalTrials.gov/show/NCT04347031
NCT04348370	594	BCG vaccine	BCG Vaccine for Health Care Workers as Defense Against COVID 19 (BADAS)	https://ClinicalTrials.gov/show/NCT04348370
NCT04349228	595	Chloroquine		https://ClinicalTrials.gov/show/NCT04349228
NCT04349592	596	Chloroquine / Azithromycin		https://ClinicalTrials.gov/show/NCT04349592
NCT04350580	597	Immunoglobulin	Polyvalent Immunoglobulin in COVID-19 Related ARds	https://clinicaltrials.gov/show/NCT04350580
NCT04351243	598	Gimsilumab	A Study to Assess the Efficacy and Safety of of Gimsilumab in Subjects With Lung Injury or Acute Respiratory Distress Syndrome Secondary to COVID-19.	https://clinicaltrials.gov/show/NCT04351243
NCT04351490	599	Vitamin D / Zinc	Impact of Zinc and Vitamin D3 Supplementation on the Survival of Aged Patients Infected With COVID-19	https://clinicaltrials.gov/show/NCT04351490
NCT04351516	600	Chloroquine		https://ClinicalTrials.gov/show/NCT04351516
NCT04352400	601	Nafamostat Mesilate	Efficacy of Nafamostat in Covid-19 Patients (RACONA Study)	https://clinicaltrials.gov/show/NCT04352400
NCT04352608	602	Inactivated novel coronavirus vaccine	Safety and Immunogenicity Study of 2019-nCoV Vaccine (Inactivated) for Prophylaxis SARS CoV-2 Infection (COVID-19)	https://clinicaltrials.gov/show/NCT04352608
NCT04352933	603	Chloroquine	PROLIFIC ChemoprophylaxisTrial (COVID-19)	https://clinicaltrials.gov/show/NCT04352933
NCT04352946	604	Chloroquine	HEalth Care Worker pROphylaxis Against COVID-19: The HERO Trial	https://clinicaltrials.gov/show/NCT04352946
NCT04352959	605	Mouthwash	COVID-19: Nasal and Salivary Detection of the SARS-CoV-2 Virus After Antiviral Mouthrinses	https://clinicaltrials.gov/show/NCT04352959
NCT04353037	606	Chloroquine	PATCH 2&3:Prevention & Treatment of COVID-19 (Severe Acute Respiratory Syndrome Coronavirus 2) With Hydroxychloroquine	https://clinicaltrials.gov/show/NCT04353037
NCT04353128	607	Melatonin	Efficacy of Melatonin in the Prophylaxis of Coronavirus Disease 2019 (COVID-19) Among Healthcare Workers.	https://clinicaltrials.gov/show/NCT04353128
NCT04353180	608	Vitamin A	Assessment the Activity Value of 13- Cis-Retinoic Acid(Isotretinoin) in the Treatment of COVID-19	https://clinicaltrials.gov/show/NCT04353180
NCT04353271	609	Chloroquine	Trial of Hydroxychloroquine In Covid-19 Kinetics	https://clinicaltrials.gov/show/NCT04353271
NCT04353284	610	Camostat mesilate	Camostat Mesylate in COVID-19 Outpatients	https://clinicaltrials.gov/show/NCT04353284
NCT04353336	611	Chloroquine	Efficacay of Chloroquine in COVID-19 Treatment	https://clinicaltrials.gov/show/NCT04353336
NCT04353518	612	Vaccine mycobacterium w	Clinical Trial to Evaluate Mycobacterium w in Preventing COVID-19 in Subjects at Risk of Getting Infected With COVID-19	https://clinicaltrials.gov/show/NCT04353518
NCT04354259	613	INF-Lambda	Interferon Lambda for Immediate Antiviral Therapy at Diagnosis	https://clinicaltrials.gov/show/NCT04354259
NCT04354389	614	DAS181	DAS181 for STOP COVID-19	https://clinicaltrials.gov/show/NCT04354389
NCT04354428	615	Chloroquine / Azithromycin / Ascorbic Acid / Folic Acid	Treatment for SARS-CoV-2 in High-Risk Adult Outpatients	https://clinicaltrials.gov/show/NCT04354428
NCT04354597	616	Chloroquine / Azithromycin	Hydroxychloroquine and Azithromycin as Prophylaxis for Healthcare Workers Dealing With COVID19 Patients	https://clinicaltrials.gov/show/NCT04354597
NCT04354805	617	Chloropromazine	Administration of Chloropromazine as a Treatment for COVID-19	https://clinicaltrials.gov/show/NCT04354805
NCT04355026	618	Chloroquine / Bromhexine	Use of Bromhexine and Hydroxychloroquine for Treatment of COVID-19 Pneumonia	https://clinicaltrials.gov/show/NCT04355026
NCT04355143	619	Colchicine	Colchicine to Reduce Myocardial Injury in COVID-19 (COLHEART-19)	https://clinicaltrials.gov/show/NCT04355143
NCT04355364	620	Pulmozyme	Efficacy and Safety of Aerosolized Intra-tracheal Dornase Alpha Administration in Patients With COVID19-induced ARDS (COVIDORNASE)	https://clinicaltrials.gov/show/NCT04355364
NCT04355429	621	Captopril	Efficacy of Captopril in Covid-19 Patients With Severe Acute Respiratory Syndrome (SARS) CoV-2 Pneumonia (CAPTOCOVID)	https://clinicaltrials.gov/show/NCT04355429
NCT04355637	622	Budesonide	Inhaled Corticosteroid Treatment of COVID19 Patients With Pneumonia	https://clinicaltrials.gov/show/NCT04355637
NCT04355676	623	Selinexor	Evaluation of Activity and Safety of Two Regimens of Low Dose Oral Selinexor in Participants With Moderate or Severe COVID-19	https://clinicaltrials.gov/show/NCT04355676
NCT04355728	624	Stem cells	Use of UC-MSCs for COVID-19 Patients	https://clinicaltrials.gov/show/NCT04355728
NCT04355767	625	Convalescent Plasma	Convalescent Plasma vs. Placebo in Emergency Room Patients With COVID-19	https://clinicaltrials.gov/show/NCT04355767
NCT04355936	626	Telmisartan	Telmisartan for Treatment of COVID-19 Patients	https://clinicaltrials.gov/show/NCT04355936
NCT04355962	627	Sevoflurane	Sevoflurane in COVID-19 ARDS (SevCov)	https://clinicaltrials.gov/show/NCT04355962
NCT04356495	628	Chloroquine / Imatinib / Favipiravir / Telmisartan / Vitamins	Treatments to Decrease the Risk of Hospitalization or Death in Elderly Outpatients With Symptomatic SARS-CoV-2 Infection (COVID-19)	https://clinicaltrials.gov/show/NCT04356495

Registry entry ID	Included ID	Intervention	Title	Webpage
NCT04356534	629	Convalescent Plasma	Convalescent Plasma Trial in COVID -19 Patients	https://clinicaltrials.gov/show/NCT04356534
NCT04356937	630	Tocilizumab	Efficacy of Tocilizumab on Patients With COVID-19	https://clinicaltrials.gov/show/NCT04356937
NCT04357028	631	Measles Vaccine	Measles Vaccine in HCW	https://clinicaltrials.gov/show/NCT04357028
NCT04357444	632	IL-2	Low Dose of IL-2 In Acute Respiratory DistrEss Syndrome Related to COVID-19	https://clinicaltrials.gov/show/NCT04357444
NCT04357457	633	Almitrine	Efficacy of Intravenous Almitrine in Reducing the Need for Mechanical Ventilation in Patients With Hypoxemic Acute Respiratory Failure Due to Covid-19-related Pneumonia	https://clinicaltrials.gov/show/NCT04357457
NCT04357613	634	Imatinib	IMATINIB IN COVID-19 DISEASE IN AGED PATIENTS.	https://clinicaltrials.gov/show/NCT04357613
NCT04357808	635	Sarilumab	Efficacy of Subcutaneous Sarilumab in Hospitalised Patients With Moderate-severe COVID-19 Infection (SARCOVID)	https://clinicaltrials.gov/show/NCT04357808
NCT04357860	636	Sarilumab	Clinical Trial of Sarilumab in Adults With COVID-19	https://clinicaltrials.gov/show/NCT04357860
NCT04357990	637	Kerecis Oral and Nasal Spray	Kerecis Oral and Nasal Spray for Treating the Symptoms of COVID-19	https://clinicaltrials.gov/show/NCT04357990
NCT04358068	638	Chloroquine / Azithromycin	Evaluating the Efficacy of Hydroxychloroquine and Azithromycin to Prevent Hospitalization or Death in Persons With COVID-19	https://clinicaltrials.gov/show/NCT04358068
NCT04358081	639	Azithromycin	Hydroxychloroquine Monotherapy and in Combination With Azithromycin in Patients With Moderate and Severe COVID-19 Disease	https://clinicaltrials.gov/show/NCT04358081
NCT04358549	640	Favipiravir	Study of the Use of Favipiravir in Hospitalized Subjects With COVID-19	https://clinicaltrials.gov/show/NCT04358549
NCT04358783	641	Convalescent Plasma	Convalescent Plasma Compared to the Best Available Therapy for the Treatment of SARS-CoV-2 Pneumonia	https://clinicaltrials.gov/show/NCT04358783
NCT04358809	642	Vaccine mycobacterium w	Clinical Trial of Mycobacterium w in COVID-19 Positive Patients, Hospitalized But Not Critically Ill	https://clinicaltrials.gov/show/NCT04358809
NCT04358926	643	Hyperbaric oxygen	Hyperbaric Oxygen Therapy Effect in COVID-19 RCT (HBOTCOVID19)	https://clinicaltrials.gov/show/NCT04358926
NCT04358939	644	Prone position	Prone Position in Patients on High-flow Nasal Oxygen Therapy for COVID-19 (HIGH-PRONE-COVID-19)	https://clinicaltrials.gov/show/NCT04358939
NCT04359095	645	Chloroquine / Lopinavir/Ritonavir / Azithromycin	Effectiveness and Safety of Medical Treatment for SARS-CoV-2 (COVID-19) in Colombia	https://clinicaltrials.gov/show/NCT04359095
NCT04359277	646	Enoxaparin	A Randomized Trial of Anticoagulation Strategies in COVID-19	https://clinicaltrials.gov/show/NCT04359277
NCT04359303	647	ozone autohemotherapy		https://ClinicalTrials.gov/show/NCT04359303
NCT04359316	648	Chloroquine / Azithromycin	Azithromycin in Hospitalized COVID-19 Patients	https://clinicaltrials.gov/show/NCT04359316
NCT04359329	649	Estradiol	Estrogen Patch for COVID-19 Symptoms	https://clinicaltrials.gov/show/NCT04359329
NCT04359511	650	Prednisolone	Efficacy and Safety of Corticosteroids in Oxygen-dependent Patients With COVID-19 Pneumonia	https://clinicaltrials.gov/show/NCT04359511
NCT04359537	651	Chloroquine	Efficacy of Various Doses of Hydroxychloroquine in Pre-Exposure Prophylaxis for COVID 19	https://clinicaltrials.gov/show/NCT04359537
NCT04359615	652	Chloroquine / favipiravir	Favipiravir in Hospitalized COVID-19 Patients	https://clinicaltrials.gov/show/NCT04359615
NCT04359654	653	Pulmozyme	Nebulised Dornase Alfa for Treatment of COVID-19	https://clinicaltrials.gov/show/NCT04359654
NCT04359680	654	Nitazoxanide	Trial to Evaluate the Efficacy and Safety of Nitazoxanide (NTZ) for Pre- or Post Exposure Prophylaxis of COVID-19 and Other Viral Respiratory Illnesses (VRI) in Healthcare Workers	https://clinicaltrials.gov/show/NCT04359680
NCT04359797	655	Prone position	COVID-19 Patient Positioning Pragmatic Trial	https://clinicaltrials.gov/show/NCT04359797
NCT04359810	656	Convalescent Plasma	Plasma Therapy of COVID-19 in Critically Ill Patients	https://clinicaltrials.gov/show/NCT04359810
NCT04359862	657	Sevoflurane / propofol		https://ClinicalTrials.gov/show/NCT04359862
NCT04359901	658	Sarilumab	Sarilumab for Patients With Moderate COVID-19 Disease: A Randomized Controlled Trial With a Play-The-Winner Design	https://clinicaltrials.gov/show/NCT04359901
NCT04359953	659	Chloroquine / Azithromycin / Telmisartan	Efficacy of Hydroxychloroquine, Telmisartan and Azithromycin on the Survival of Hospitalized Elderly Patients With COVID-19	https://clinicaltrials.gov/show/NCT04359953
NCT04360096	660	Aviptadil	Inhaled Aviptadil for the Treatment of Non-Acute Lung Injury in COVID-19	https://clinicaltrials.gov/show/NCT04360096
NCT04360122	661	Levamisole / Isoprinosine	Levamisole and Isoprinosine in Immune-prophylaxis of Egyptian Healthcare Workers Facing COVID-19	https://clinicaltrials.gov/show/NCT04360122
NCT04360356	662	Ivermectin / nitazoxanide	Ivermectin and Nitazoxanide Combination Therapy for COVID-19	https://clinicaltrials.gov/show/NCT04360356
NCT04360551	663	Telmisartan	Pilot Clinical Trial of the Safety and Efficacy of Telmisartan for the Mitigation of Pulmonary and Cardiac Complications in COVID-19 Patients	https://clinicaltrials.gov/show/NCT04360551
NCT04360759	664	Chloroquine	Chloroquine Outpatient Treatment Evaluation for HIV-Covid-19	https://clinicaltrials.gov/show/NCT04360759
NCT04360824	665	Enoxaparin	Covid-19 Associated Coagulopathy	https://clinicaltrials.gov/show/NCT04360824
NCT04360876	666	Dexamethasone	Targeted Steroids for ARDS Due to COVID-19 Pneumonia: A Pilot Randomized Clinical Trial	https://clinicaltrials.gov/show/NCT04360876
NCT04360980	667	Colchicine	The Effects of Standard Protocol With or Without Colchicine in Covid-19 Infection	https://clinicaltrials.gov/show/NCT04360980
NCT04361032	668	Tocilizumab / Deferoxamine	Assessment of Efficacy and Safety of Tocilizumab Compared to DefeROxamine, Associated With Standards Treatments in COVID-19 (+) Patients Hospitalized In Intensive Care in Tunisia	https://clinicaltrials.gov/show/NCT04361032
NCT04361253	669	Convalescent Plasma	Evaluation of SARS-CoV-2 Antibody-containing Plasma thErapy	https://clinicaltrials.gov/show/NCT04361253
NCT04361318	670	Chloroquine / nitazoxanide	Hydroxychloroquine and Nitazoxanide Combination Therapy for COVID-19	https://clinicaltrials.gov/show/NCT04361318
NCT04361422	671	Vitamin A	Isotretinoin in Treatment of COVID-19	https://clinicaltrials.gov/show/NCT04361422
NCT04361435	672	Physiotherapy oscillating device	Lung Recruitment Device for COVID-19	https://clinicaltrials.gov/show/NCT04361435
NCT04361461	673	Azithromycin	Use of Hydroxychloroquine Alone or Associated for Inpatients With SARS-CoV2 Virus (COVID-19)	https://clinicaltrials.gov/show/NCT04361461
NCT04361526	674	Cytokine adsorption	Pilot Study on Cytokine Filtration in COVID-19 ARDS	https://clinicaltrials.gov/show/NCT04361526
NCT04361643	675	Lenalidomide	LENALIDOMIDE COVID-19 TRIAL	https://clinicaltrials.gov/show/NCT04361643
NCT04362059	676	alveofact	A Clinical Trial of Nebulized Bovactant (Alveofact¬Æ) for the Treatment of Moderate to Severe COVID-19	https://clinicaltrials.gov/show/NCT04362059
NCT04362085	677	Dalteparin / enoxaparin	Coagulopathy of COVID-19: A Pragmatic Randomized Controlled Trial of Therapeutic Anticoagulation Versus Standard Care	https://clinicaltrials.gov/show/NCT04362085
NCT04362111	678	Anakinra	Early Identification and Treatment of Cytokine Storm Syndrome in Covid-19	https://clinicaltrials.gov/show/NCT04362111
NCT04362124	679	BCG vaccine	Performance Evaluation of BCG vs COVID-19	https://clinicaltrials.gov/show/NCT04362124
NCT04362176	680	Convalescent Plasma	Passive Immunity Trial of Nashville II	https://clinicaltrials.gov/show/NCT04362176
NCT04362189	681	Stem cells	Efficacy and Safety Study of Allogeneic HB-adMSCs for the Treatment of COVID-19	https://clinicaltrials.gov/show/NCT04362189
NCT04362813	682	Canakinumab	Study of Efficacy and Safety of Canakinumab Treatment for CRS in Participants With COVID-19-induced Pneumonia (CAN-COVID)	https://ClinicalTrials.gov/show/NCT04362813
NCT04363060	683	Azithromycin	Azithromycin+Amoxicillin/Clavulanate vs Amoxicillin/Clavulanate in COVID19 Patients With Pneumonia in Non-intensive Unit (AziA)	https://ClinicalTrials.gov/show/NCT04363060
NCT04363216	684	Vitamin C		https://ClinicalTrials.gov/show/NCT04363216
NCT04363372	685	MRx4DP0004		https://ClinicalTrials.gov/show/NCT04363372
NCT04363437	686	Colchicine		https://ClinicalTrials.gov/show/NCT04363437
NCT04363450	687	Chloroquine		https://ClinicalTrials.gov/show/NCT04363450
NCT04363463	688	Prone position		https://ClinicalTrials.gov/show/NCT04363463
NCT04363502	689	Clazakizumab		https://ClinicalTrials.gov/show/NCT04363502
NCT04363736	690	Tocilizumab		https://ClinicalTrials.gov/show/NCT04363736
NCT04363814	691	Bactek-R	Clinical Impact of BACTEK-R in Subject With Mild Pneumonia Due to COVID-19 Infection	https://ClinicalTrials.gov/show/NCT04363814
NCT04363827	692	Chloroquine		https://ClinicalTrials.gov/show/NCT04363827
NCT04363840	693	Vitamin D / aspirin		https://ClinicalTrials.gov/show/NCT04363840
NCT04363866	694	Chloroquine		https://ClinicalTrials.gov/show/NCT04363866
NCT04364009	695	Anakinra	Anakinra for COVID-19 Respiratory Symptoms (ANACONDA)	https://ClinicalTrials.gov/show/NCT04364009
NCT04364022	696	Chloroquine / Lopinavir/ Ritonavir		https://ClinicalTrials.gov/show/NCT04364022
NCT04364737	697	Convalescent Plasma		https://ClinicalTrials.gov/show/NCT04364737
NCT04364763	698	RBT-9		https://ClinicalTrials.gov/show/NCT04364763
NCT04364815	699	Chloroquine		https://ClinicalTrials.gov/show/NCT04364815
NCT04364893	700	ARB and ACE discontinuation	Angiotensin Receptor Blockers and Angiotensin-converting Enzyme Inhibitors and Adverse Outcomes in Patients With COVID19 (BRACE-CORONA)	https://ClinicalTrials.gov/show/NCT04364893
NCT04365101	701	NK Cells		https://ClinicalTrials.gov/show/NCT04365101
NCT04365127	702	Progesterone		https://ClinicalTrials.gov/show/NCT04365127
NCT04365153	703	Canakinumab		https://ClinicalTrials.gov/show/NCT04365153
NCT04365231	704	Chloroquine / Azithromycin		https://ClinicalTrials.gov/show/NCT04365231
NCT04365257	705	Prazosin		https://ClinicalTrials.gov/show/NCT04365257
NCT04365309	706	Aspirin	Protective Effect of Aspirin on COVID-19 Patients (PEAC)	https://ClinicalTrials.gov/show/NCT04365309
NCT04365517	707	Sitagliptin		https://ClinicalTrials.gov/show/NCT04365517
NCT04365582	708	Chloroquine / Lopinavir/Ritonavir / Azithromycin		https://ClinicalTrials.gov/show/NCT04365582
NCT04365985	709	Naltrexone / ketamine		https://ClinicalTrials.gov/show/NCT04365985
NCT04366050	710	Ramipril		https://ClinicalTrials.gov/show/NCT04366050
NCT04366063	711	Stem cells		https://ClinicalTrials.gov/show/NCT04366063
NCT04366089	712	ozone autohemotherapy		https://ClinicalTrials.gov/show/NCT04366089
NCT04366115	713	Hydrocortisone / AVM0703		https://ClinicalTrials.gov/show/NCT04366115
NCT04366180	714	Probiotic		https://ClinicalTrials.gov/show/NCT04366180
NCT04366232	715	Anakinra / ruxolitinib	Efficacy of Intravenous Anakinra and Ruxolitinib During COVID-19 Inflammation (JAKINCOV) (JAKINKOV)	https://ClinicalTrials.gov/show/NCT04366232
NCT04366245	716	Chloroquine / Azithromycin / Lopinavir/ritonavir / InF-B / Hyperimmune		https://ClinicalTrials.gov/show/NCT04366245
NCT04366271	717	Stem cells		https://ClinicalTrials.gov/show/NCT04366271
NCT04366323	718	Stem cells		https://ClinicalTrials.gov/show/NCT04366323

Registry entry ID	Included ID	Intervention	Title	Webpage
NL8523	719	Isoflurane	Volatile Anaesthetics in COVID-19 ,Äì a pilot study	https://trialregister.nl/trial/8523
IRCT20081019001369N5	720	Lithium	Clinical trial of lithium in improving the clinical and laboratory symptoms of patients with COVID-19	https://www.irct.ir/trial/47141
IRCT20200415047082N1	721	Herbal medicine	Effect evaluation of herbal supplementation from the combination of sugarcane, black myrobalan and mastic along with the treatment protocol of the Ministry of Health on the course of COVID-19 disease	https://www.irct.ir/trial/47193
IRCT20200406046963N1	722	Methylprednisolone	Evaluation of the efficacy and safety of methylprednisolone pulse therapy in treatment of Covid-19 patients with ARDS	https://www.irct.ir/trial/47088
IRCT20200421047155N1	723	Chloroquine / tenofovir	Therapeutic Effect of Tenofovir and hydroxychloroquine combination therapy compared to chloroquine alone in patients with COVID-19	https://www.irct.ir/trial/47387
IRCT20080901001165N48	724	Herbal medicine	Investigating the efficacy of Zofa (a herbal medicine) in symptom management of patients with COVID-19	https://www.irct.ir/trial/47318
IRCT20200410047009N1	725	Naproxen	Evaluation of the role of naproxen as adjunctive therapy with standard therapies and its efficacy in the early improvement and reduced mortality rate of COVID-19 patients	https://www.irct.ir/trial/47052
IRCT20120215009014N353	726	Convalescent Plasma	Effect of plasma of patients recovered from covid-19 versus control group on treatment of covid-19: a randomized clinical trial	https://www.irct.ir/trial/47501
IRCT20080901001165N49	727	Herbal medicine	Evaluation efficacy of a herbal combination (contains Glycyrrhiza glabra, Mentha pulegium and Urtica) in controlling symptoms in patients with COVID-19	https://www.irct.ir/trial/47401
IRCT20200420047147N1	728	Sitagliptin	Evaluation the efficacy and safety of Sitagliptin administration in patients with COVID-19	https://www.irct.ir/trial/47334
IRCT20200408046990N2	729	Colchicine	Evaluation of colchicine tablet efficacy as an adjuvant treatment for patients with mild to moderate COVID-19 in Qaem Hospital, Mashhad: A triple-blind randomized placebo controlled clinical trial	https://www.irct.ir/trial/47468
IRCT20200408046987N1	730	Ivermectin	Dose-Finding study of Ivermectin treatment on patients infected with Covid-19:A clinical trial	https://www.irct.ir/trial/47012
IRCT20200417047113N1	731	NK Cells	Evaluating the safety and efficacy of allogeneic NK cells on COVID-19 induced pneumonia, double blind, randomized clinical trial	https://www.irct.ir/trial/47264
IRCT20200416047104N1	732	TCM	The effect of pomegranate tea on symptom relief of COVID-19 patients: a double-blind randomized clinical trial study	https://www.irct.ir/trial/47251
IRCT20170207032444N3	733	Thalidomide	The efficacy and safety of Thalidomide in severe Covid19 pneumonia: Arandomized controlled clinical trial	https://www.irct.ir/trial/47119
IRCT20170210032478N3	734	Formoterol	The efficacy of inhaled formoterol on symptom improvement in covid 19 patients	https://www.irct.ir/trial/47173
IRCT20200422047168N1	735	Tenofovir	Comparison of the Effectiveness of Tenofovir antiviral drug beside (Kaletra and Chloroquine) with routine drug regime (Kaletra and Chloroquine) in COVID-19 patients	https://www.irct.ir/trial/47437
IRCT20140407017169N2	736	TCM	The Effect of a Persian Natural Medicine Product based on Tahini Oil on Clinical Signs in Suspected Patients with COVID-19	https://www.irct.ir/trial/47295
EUCTR2020-001678-31	737	BCG vaccine	Randomized controlled trial evaluating the efficacy of vaccination with Bacillus Calmette and Guérin (BCG) in the Prevention of COVID-19 via the strengthening of innate immunity in Health Care Workers	https://www.clinicaltrialsregister.eu/ctr-search/trial/2020-001678-31/FR
EUCTR2020-001717-20	738	Vitamin D	Prevention and treatment with Calcifediol of Coronavirus COVID-19-induced acute respiratory syndrome (SARS)	https://www.clinicaltrialsregister.eu/ctr-search/trial/2020-001717-20/ES
ChiCTR2000032487	739	Chloroquine	Study for using sulfate in the prevention and control of novel coronavirus pneumonia (COVID-19) in high and low prevalence communities	http://www.chictr.org.cn/showproj.aspx?proj=52394
ChiCTR2000032573	740	Bu-Fei-Huo-Xue Capsule	A randomized, double-blind, controlled trial for Bu-Fei-Huo-Xue Capsule in the treatment of novel coronavirus pneumonia (COVID-19) convalescence patient with "Fei-Pi-Qi-Xu zhen"	http://www.chictr.org.cn/showproj.aspx?proj=53225
DRKS00021447	741	Cyto-sorb	CytoResc - CytoSorb Rescue for COVID-19 Cytokine Storm	http://www.drks.de/DRKS00021447
EUCTR2020-001888-90	742	BCG Vaccine	BCG vaccine for healthcare workers against COVID-19	https://www.clinicaltrialsregister.eu/ctr-search/search?query=eudract_number:2020-001888-90
NCT04366739	743	Chlorpromazine	Repurposing of Chlorpromazine in Covid-19 Treatment	https://clinicaltrials.gov/show/NCT04366739
NCT04366856	744	Prone position	PROne Positioning in coVID-19 Oxygeno-dependent Patients in Spontaneous Ventilation (PROVID Study)	https://clinicaltrials.gov/show/NCT04366856
NCT04366960	745	Enoxaparin	Comparison of Two Doses of Enoxaparin for Thromboprophylaxis in Hospitalized COVID-19 Patients	https://clinicaltrials.gov/show/NCT04366960
NCT04367077	746	Stem cells	MultiStem Administration for COVID-19 Induced ARDS (MACoVIA)	https://clinicaltrials.gov/show/NCT04367077
NCT04367831	747	Heparin	Intermediate or Prophylactic-Dose Anticoagulation for Venous or Arterial Thromboembolism in Severe COVID-19	https://clinicaltrials.gov/show/NCT04367831
NCT04368000	748	Prone position	Prone Positioning in Awake Patients With COVID-19 Requiring Hospitalization	https://clinicaltrials.gov/show/NCT04368000
NCT04368156	749	Vagus nerve stimulation	Study Assessing Vagus Nerve Stimulation in CoViD-19 Respiratory Symptoms	https://clinicaltrials.gov/show/NCT04368156
NCT04368728	750	Vaccine SARS-COV-2 RNA	Study to Describe the Safety, Tolerability, Immunogenicity, and Potential Efficacy of RNA Vaccine Candidates Against COVID-19 in Healthy Adults	https://clinicaltrials.gov/show/NCT04368728
NCT04368988	751	Vaccine nanoparticle	Evaluation of the Safety and Immunogenicity of a SARS-CoV-2 rS (COVID-19) Nanoparticle Vaccine With/Without Matrix-M Adjuvant	https://clinicaltrials.gov/show/NCT04368988
NCT04369365	752	Azithromycin	A Single-blinded, Randomized, Placebo Controlled Phase II Trial of Prophylactic Treatment With Oral Azithromycin Versus Placebo in Cancer Patients Undergoing Antineoplastic Treatment During the Corona Virus Disease 19 (COVID-19) Pandemic	https://clinicaltrials.gov/show/NCT04369365
NCT04369469	753	Ravulizumab	Efficacy and Safety Study of IV Ravulizumab in Patients With COVID-19 Severe Pneumonia	https://clinicaltrials.gov/show/NCT04369469
NCT04369794	754	BCG Vaccine	COVID-19: BCG As Therapeutic Vaccine, Transmission Limitation, and Immunoglobulin Enhancement	https://clinicaltrials.gov/show/NCT04369794
NCT04370015	755	Chloroquine	Hydroxychloroquine Chemoprophylaxis for COVID-19 Infection in High-risk Healthcare Workers: Randomised Control Trial	https://clinicaltrials.gov/show/NCT04370015
NCT04370223	756	ozone autohemotherapy	Ozone Auto-hemotherapy for COVID-19 Pneumonia	https://clinicaltrials.gov/show/NCT04370223
NCT04370236	757	XPro1595	XPro1595 for the Treatment of Pulmonary Complications From COVID-19	https://clinicaltrials.gov/show/NCT04370236
NCT04370262	758	Chloroquine/Famotidine	Multi-site Adaptive Trials Using Hydroxycholoroquine for COVID-19	https://clinicaltrials.gov/show/NCT04370262
NCT04370288	759	Methylene Blue	Clinical Application of Methylene Blue for Treatment of Covid-19 Patients	https://clinicaltrials.gov/show/NCT04370288
NCT04370782	760	Azithromycin/Doxycycline	Hydroxychloroquine and Zinc With Either Azithromycin or Doxycycline for Treatment of COVID-19 in Outpatient Setting	https://clinicaltrials.gov/show/NCT04370782
NCT04371367	761	avdoralimab	Avdoralimab an Anti-C5aR Antibody, in Patients With COVID-19 Severe Pneumonia (FORCE)	https://clinicaltrials.gov/show/NCT04371367
NCT04371393	762	Stem cells	MSCs in COVID-19 ARDS	https://clinicaltrials.gov/show/NCT04371393
NCT04371406	763	Chloroquine / Azithromycin	Efficacy of Azithromycin-associated Hydroxychloroquine Therapy Given in General Practice in Early-stage Disease in COVID-19 Patients	https://clinicaltrials.gov/show/NCT04371406
NCT04371523	764	Chloroquine	Hydroxychloroquine to Prevent COVID-19 Disease Amongst Healthcare Workers	https://clinicaltrials.gov/show/NCT04371523
NCT04371601	765	Stem cells	Safety and Effectiveness of Mesenchymal Stem Cells in the Treatment of Pneumonia of Coronavirus Disease 2019	https://clinicaltrials.gov/show/NCT04371601
NCT04371640	766	Sirolimus	Sirolimus in COVID-19 Phase 1	https://clinicaltrials.gov/show/NCT04371640
NCT04371952	767	Doxycycline	DYNAMIC Study (DoxycYcliNe AMbulatoIre COVID-19)	https://clinicaltrials.gov/show/NCT04371952
NCT04371965	768	Povidone Iodine	Povidone Iodine Mouthwash, Gargle, and Nasal Spray to Reduce Naso- Pharyngeal Viral Load in Patients With COVID-19	https://clinicaltrials.gov/show/NCT04371965
NCT04371978	769	Linagliptin	Efficacy and Safety of Dipeptidyl Peptidase-4 Inhibitors in Diabetic Patients With Established COVID-19	https://clinicaltrials.gov/show/NCT04371978
NCT04372017	770	Chloroquine	Randomized, Double-Blind,Controlled Trial of Hydroxychloroquine as Post-Exposure Prophylaxis Against COVID-19 Infection	https://clinicaltrials.gov/show/NCT04372017
NCT04372082	771	Chloroquine/diltiazem	Hydroxychloroquine or Diltiazem-Niclosamide for the Treatment of COVID-19	https://clinicaltrials.gov/show/NCT04372082
NCT04372186	772	Tocilizumab	A Study to Evaluate the Efficacy and Safety of Tocilizumab in Hospitalized Participants With COVID-19 Pneumonia	https://clinicaltrials.gov/show/NCT04372186
EUCTR2020-001766-11-FR	773	Losartan and spironolactone	Losartan and spironolactone treatment for COVID-19 patients with acute respiratory failure in intensive care unit - COVIDANCE	https://www.clinicaltrialsregister.eu/ctr-search/search?query=eudract_number:2020-001766-11
EUCTR2020-001709-21-FR	774	Enoxaparin / tinzaparin / dalteparin / nadroparin	Effectiveness of low molecular weight heparin at increased doses prophylaxis weight-adjusted, compared with lower doses prophylaxis (intermediate or standard), on the onset of venous thromboembolism in coronavirus disease 2019 (COVID-19) hospitalized patients : The randomized multicentric controlled open-label	https://www.clinicaltrialsregister.eu/ctr-search/search?query=eudract_number:2020-001709-21
EUCTR2020-001934-37-ES	775	Methylprednisolone	USE OF CORTICOSTEROIDS IN PATIENTS WITH SARS-COV2 CORONAVIRUS INFECTION (GLUCOCOVID). Pragmatic trial inserted in real practice during a pandemic COVID-19 - USE OF CORTICOSTEROIDS IN PATIENTS WITH SARS-COV2 CORONAVIRUS INFECTION	https://www.clinicaltrialsregister.eu/ctr-search/search?query=eudract_number:2020-001934-37
EUCTR2020-001786-36-GB	776	IL-7	Recombinant InterLeukin-7 (CYT107) to Improve clinical outcomes in lymphopenic pAtients with COVID-19 infection ,ÄúILIAD 7 trial,Äù - ILIAD	https://www.clinicaltrialsregister.eu/ctr-search/search?query=eudract_number:2020-001786-36
EUCTR2020-001784-88-FI	777	Chloroquine / Remdesivir	WHO SOLIDARITY Finland: The multicenter trial on the efficacy of different anti-viral drugs in SARS-CoV-2 infected patients (COVID-19) - SOLIDARITY Finland	https://www.clinicaltrialsregister.eu/ctr-search/search?query=eudract_number:2020-001784-88
EUCTR2020-001603-16-ES	778	Colchicine	A RANDOMIZATION, MULTICENTRIC, OPEN-LABEL, CONTROLLED, CLINICAL TRIAL TO INVESTIGATE THE EFFECTIVENESS OF EARLY CHOLCHICINE ADMINISTRATION IN PATIENTS OVER 70 YEARS OF AGE WITH HIGH RISK OF DEVELOPING SEVERE PULMONARY	https://www.clinicaltrialsregister.eu/ctr-search/search?query=eudract_number:2020-001603-16
EUCTR2020-001736-95-GB	779	Bemcentinib / IgG / acalabrutinib / zilocuplan / heparin	ACCORD 2: A Multicentre, Seamless, Phase 2 Adaptive Randomisation Platform Study to Assess the Efficacy and Safety of Multiple Candidate Agents for the Treatment of COVID 19 in Hospitalised Patients - ACCORD 2-Treatment of COVID 19 in Hospitalised Patients	https://www.clinicaltrialsregister.eu/ctr-search/search?query=eudract_number:2020-001736-95
EUCTR2020-001740-26-GB	780	Azithromycin	A multi-centre open-label two-arm randomised superiority clinical trial of Azithromycin versus usual care In Ambulatory COVID-19 (ATOMIC2) - ATOMIC2, Version 1.0	https://www.clinicaltrialsregister.eu/ctr-search/search?query=eudract_number:2020-001740-26
EUCTR2020-001994-66-ES	781	Ivermectin	Ramdomised clinical trial of ivermectin for treatment and prophylaxis of COVID-19	https://www.clinicaltrialsregister.eu/ctr-search/search?query=eudract_number:2020-001994-66
EUCTR2020-001721-31-GB	782	Sinus-Wash	Can a sinus rinse and mouth wash reduce viral load in COVID-19 positive individuals? - SINUS WASH pilot study	https://www.clinicaltrialsregister.eu/ctr-search/search?query=eudract_number:2020-001721-31
EUCTR2020-000705-86-ES	783	Omega-3	Control of inflammatory parameters with omega-3 fatty acid supplementation in adult patients with parenteral nutrition and respiratory infection by SARS-CoV-2: randomized clinical trial COVID-19	https://www.clinicaltrialsregister.eu/ctr-search/search?query=eudract_number:2020-000705-86
EUCTR2020-001206-35-AT	784	ACE Discontinuation	Stopping ACE-inhibitors in COVID-19 - a randomized, controlled clinical trial	https://www.clinicaltrialsregister.eu/ctr-search/search?query=eudract_number:2020-001206-35
EUCTR2020-001296-33-DK	785	Prostacyclin	Efficacy and safety of 72-hour infusion of Prostacyclin (1 ng/kg/min) in patients with COVID-19 induced respiratory failure ,Äì a multicentre randomized, placebo-controlled, blinded, investigator-initiated trial	https://www.clinicaltrialsregister.eu/ctr-search/search?query=eudract_number:2020-001296-33
EUCTR2020-001228-32-GB	786	Vaccine COV002	A phase 2/3 study to determine the efficacy, safety and immunogenicity of the candidate Coronavirus Disease (COVID-19) vaccine ChAdOx1 nCoV-19 - Investigating a Vaccine Against COVID-19 (COV002)	https://www.clinicaltrialsregister.eu/ctr-search/search?query=eudract_number:2020-001228-32
EUCTR2020-001697-30-ES	787	Chloroquine	PRE-EXPOSURE PROPHYLAXIS WITH HYDROXYCHLOROQUINE IN SANITARIES HIGHLY EXPOSED TO COVID-19. (COVIDNA) - COVIDNA	https://www.clinicaltrialsregister.eu/ctr-search/trial/2020-001697-30/ES
EUCTR2020-001827-15-ES	788	Methylprednisolone	EARLY TREATMENT OF PNEUMONIA COVID-19 WITH GLUCOCORTICOIDS. RANDOMIZED CONTROLLED CLINICAL TRIAL - CORTIVID	https://www.clinicaltrialsregister.eu/ctr-search/trial/2020-001827-15/ES
EUCTR2020-002032-69-ES	789	Tocilizumab	Single-center, randomized, open-label clinical trial on the efficacy of tocilizumab in modifying the inflammatory parameters of patients with COVID-19	https://www.clinicaltrialsregister.eu/ctr-search/trial/2020-002032-69/ES
EUCTR2020-001891-14-ES	790	Enoxaparin	Impact of the use of low molecular weight heparins (LMWH), at prophylactic versus intermediate doses, on SARS-CoV2 infection (COVID-19) - Heparin-SARS-CoV2	https://www.clinicaltrialsregister.eu/ctr-search/trial/2020-001891-14/ES
EUCTR2020-001632-10-DE	791	Convalescent Plasma	A Randomized Open label Phase-II Clinical Trial with or without Infusion of Plasma from Subjects after Convalescence of SARS-CoV-2 Infection in High-Risk Patients with Confirmed Severe SARS-CoV-2 Disease - RECOVER	https://www.clinicaltrialsregister.eu/ctr-search/trial/2020-001632-10/DE
EUCTR2020-001635-27-FR	792	Chloroquine vs Masitinib/Isoquercetin	A Phase 2, Randomized, Open-label Clinical Trial comparing the Safety and Efficacy of Hydroxychloroquine, and Standard of Care to a Combination of Masitinib, Isoquercetin, and Standard of Care for the Treatment of Hospitalized Moderate and Severe COVID-19 Patients.	https://www.clinicaltrialsregister.eu/ctr-search/trial/2020-001635-27/FR
EUCTR2020-001696-32-ES	793	IVIG	A Multicenter, Randomized, Open-label Parallel Group Pilot Study to Evaluate Safety and Efficacy of High Dose Intravenous Immune Globulin (IVIG) plus Standard Medical Treatment (SMT) versus SMT alone in Hospitalized Subjects with COVID-19 - Study to Evaluate the Safety and Efficacy of High Dose IVIG	https://www.clinicaltrialsregister.eu/ctr-search/trial/2020-001696-32/ES
EUCTR2020-001953-36-ES	794	Prolastin	A Multicenter, Randomized, Open-label, Parallel Group Pilot Study to Evaluate the Safety and Efficacy of Prolastin¬Æ plus Standard Medical Treatment (SMT) versus SMT alone in Hospitalized Subjects with COVID-19.	https://www.clinicaltrialsregister.eu/ctr-search/search?query=eudract_number:2020-001953-36
EUCTR2020-001449-38-GB	795	Chloroquine/Azithromycin vs Zinc vs Favipiravir	A Randomised Controlled Trial of Early Intervention in Patients HospItalised with COVID-19: Favipiravir verses HydroxycholorquiNe & Azithromycin & Zinc vErsEs Standard CaRe - PIONEER	https://www.clinicaltrialsregister.eu/ctr-search/trial/2020-001449-38/GB
EUCTR2020-001783-28-HU	796	BCG Vaccine	REDUCING ABSENCE FROM WORK OF HEALTHCARE WORKERS DUE TO COVID-19 INFECTION BY BCG (BACILLUS CALMETTE-GU√âRIN) VACCINATION - BACH (BCG Against COVID-19 in Hunga	https://www.clinicaltrialsregister.eu/ctr-search/search?query=eudract_number:2020-001783-28
EUCTR2020-001598-66-FR	797	Curosurf surfactant	Preliminary randomized controlled trial of poractant alfa (Curosurf¬Æ) by fiberoptic bronchoscopy-directed endobronchial administration in acute respiratory distress syndrome (ARDS) due to COVID-19 viral pneumonia. - CAARDS-1	https://www.clinicaltrialsregister.eu/ctr-search/trial/2020-001598-66/FR
EUCTR2020-002123-11-ES	798	Chloroquine/ciclosporin	RANDOMIZED CLINICAL TRIAL OF PARALLEL GROUPS TO COMPARE THE EFFICACY OF HYDROXYCHLOROQUINE VS. CYCLOSPORINE - HYDROXYCHLOROQUINE IN THE TREATMENT OF COVID-19 PNEUMONIA. - HYDROCYCLO STUDY	https://www.clinicaltrialsregister.eu/ctr-search/trial/2020-002123-11/ES
EUCTR2020-001643-13-GB	799	Brensocatib	A randomised double-blind placebo-controlled trial of Brensocatib (INS1007) in patients with severe COVID-19 - STOP-COVID19: Superiority Trial Of Protease inhibition in COVID-19	https://www.clinicaltrialsregister.eu/ctr-search/trial/2020-001643-13/GB
NCT04374487	800	Convalescent Plasma	A Phase II, Open Label, Randomized Controlled Trial to Assess the Safety and Efficacy of Convalescent Plasma to Limit COVID-19 Associated Complications	https://clinicaltrials.gov/show/NCT04374487
NCT04380935	801	Convalescent Plasma	Effectiveness and Safety of Convalescent Plasma Therapy on COVID-19 Patients With Acute Respiratory Distress Syndrome in Referral Hospitals in Indonesia	https://clinicaltrials.gov/show/NCT04380935
NCT04377997	802	Enoxaparin	A Randomized, Open-Label Trial of Therapeutic Anticoagulation in COVID-19 Patients With an Elevated D-Dimer	https://clinicaltrials.gov/show/NCT04377997
NCT04373460	803	Convalescent Plasma	Comparison of the Efficacy and Safety of Human Coronavirus Immune Plasma (HCIP) vs. Control (SARS-CoV-2 Non-immune) Plasma Among Outpatients With Symptomatic COVID-19.	https://clinicaltrials.gov/show/NCT04373460
NCT04375735	804	Surfactant	London's Exogenous Surfactant Study for COVID19	https://clinicaltrials.gov/show/NCT04375735
NCT04380948	805	IL-7	A Pilot Study Evaluating the Effect of NT-I7, a Long Acting Interleukin-7, to Enhance Immune Clearance of SARS-CoV-2	https://clinicaltrials.gov/show/NCT04380948
NCT04379479	806	Dialyzable Leukocyte Extract	Assessment of the Clinical Effect of Dialyzable Leukocyte Extracts in Individuals With Acute Respiratory Infection (Suspected or Confirmed Cases of COVID-19) (FUTURE-T)	https://clinicaltrials.gov/show/NCT04379479
NCT04377568	807	Convalescent Plasma	CONCOR-KIDS: A Randomized, Multicentered, Open-label Phase 2 Clinical Trial of the Safety and Efficacy of Human Coronavirus-immune Convalescent Plasma for the Treatment of COVID-19 Disease in Hospitalized Children	https://clinicaltrials.gov/show/NCT04377568
ACTRN12620000557932	808	Chloroquine / zinc / Vitamin D3/B12 / azithromycin / IV Vitamin C	Therapies to prevent progression of COVID-19, including Hydroxychloroquine, Azithromycin, Zinc, Vitamin D, Vitamin B12 with or without Vitamin C, a multi-centre, international, randomized trial: The International ALLIANCE Study	https://anzctr.org.au/ACTRN12620000557932.aspx

Registry entry ID	Included ID	Intervention	Title	Webpage
NCT04381052	809	Clazakizumab	A Randomized Placebo-Controlled Safety and Dose-Finding Study for the Use of the IL-6 Inhibitor Clazakizumab in Patients With Life-threatening COVID-19 Infection	https://clinicaltrials.gov/show/NCT04381052
NCT04380402	810	Atorvastatin	Prospective Randomized Open-label Trial of Atorvastatin as Adjunctive Treatment of COVID-19	https://clinicaltrials.gov/show/NCT04380402
NCT04381364	811	Ciclosonide	Inhalation of Ciclesonide for Patients With COVID-19: A Randomised Open Treatment Study (HALT COVID-19)	https://clinicaltrials.gov/show/NCT04381364
NCT04376788	812	Convalescent Plasma	Exchange Transfusion Versus Plasma From Convalescent Patients With Methylene Blue in Patients With COVID-19	https://clinicaltrials.gov/show/NCT04376788
NCT04379076	813	IL-7	A Multicenter, Randomized, Double-blinded Placebo-controlled Study of Recombinant Interleukin-7 (CYT107) for Immune Restoration of Hospitalized Lymphopenic Patients With Coronavirus COVID-19 Infection in UK	https://clinicaltrials.gov/show/NCT04379076
NCT04372589	814	Heparin	Antithrombotic Therapy to Ameliorate Complications of COVID-19	https://clinicaltrials.gov/show/NCT04372589
NCT04377334	815	Stem cells	Prospective Phase II Study: MSCs in Inflammation-Resolution Programs of SARS-CoV-2 Induced ARDS	https://clinicaltrials.gov/show/NCT04377334
NCT04372628	816	Chloroquine / lopinavir/ritonavir	Trial of Early Therapies During Non-hospitalized Outpatient Window (TREAT NOW) for COVID-18	https://clinicaltrials.gov/show/NCT04372628
NCT04374279	817	Ivermectin / bicalutamide	A Phase II Trial to Promote Recovery From COVID-19 With Ivermectin or Endocrine Therapy	https://clinicaltrials.gov/show/NCT04374279
NCT04380688	818	Acalabrutinib	A Phase 2, Open Label, Randomized Study of the Efficacy and Safety of Acalabrutinib With Best Supportive Care Versus Best Supportive Care in Subjects Hospitalized With COVID-19	https://clinicaltrials.gov/show/NCT04380688
NCT04377503	819	Tocilizumab vs Methylprednisolone	Comparison of the Efficacy and Safety of Tocilizumab Versus Methylprednisolone in the Cytokine Release Syndrome of Patients With COVID-19. A Prospective Randomized Controlled Phase II Trial	https://clinicaltrials.gov/show/NCT04377503
NCT04375397	820	Ibrutinib	Treatment of Patients Hospitalized for COVID-19 Infection and Pulmonary Distress With Ibrutinib	https://clinicaltrials.gov/show/NCT04375397
NCT04377711	821	Ciclosonide	A Phase 3, Multicenter, Randomized, Double-Blind, Placebo-Controlled Study to Assess the Safety and Efficacy of Ciclesonide Metered-Dose Inhaler in Non-hospitalized Patients 12 Years of Age and Older With Symptomatic COVID-19 Infection	https://clinicaltrials.gov/show/NCT04377711
NCT04376684	822	Otilimab	A Randomized, Double-blind, Placebo-controlled, Study Evaluating the Efficacy and Safety of Otilimab IV in Patients With Severe Pulmonary COVID-19 Related Disease	https://clinicaltrials.gov/show/NCT04376684
NCT04379271	823	IMU-838	A Prospective, Multi-Center, Randomized, Placebo-Controlled, Double-Blinded Study to Evaluate the Efficacy, Safety and Tolerability of IMU-838 as Addition to Investigator's Choice of Standard of Care Therapy, in Patients With Coronavirus Disease 19	https://clinicaltrials.gov/show/NCT04379271
NCT04374526	824	Convalescent Plasma	Early transfusIon of COVID-19 Convalescent Plasma in Elderly COVID-19 Patients to Prevent Disease Progression.	https://clinicaltrials.gov/show/NCT04374526
ChiCTR2000032717	825	Vitamin C	Efficacy and safety of high-dose vitamin C combined with traditional Chinese medicine in the treatment of moderate and severe novel coronavirus pneumonia (COVID-19)	http://www.chictr.org.cn/showproj.aspx?proj=53322
NCT04377646	826	Chloroquine and Zinc	A Study of Hydroxychloroquine and Zinc in the Prevention of COVID-19 Infection in Military Healthcare Workers (COVID-Milit)	https://clinicaltrials.gov/show/NCT04377646
ChiCTR2000032737	827	Microbiota transplantation	Clinical trial for the washed microbiota transplantation in the treatment of novel coronavirus pneumonia (COVID-19) patients suspected with gut microbiota dysbiosis	http://www.chictr.org.cn/showproj.aspx?proj=53458
NCT04372979	828	Convalescent Plasma	Evaluation Of Efficacy Of COVID-19 Convalescent Plasma Versus Standard Plasma In The Early Care Of COVID-19 Patients Hospitalized Outside Intensive Care Units.	https://clinicaltrials.gov/show/NCT04372979
NCT04374552	829	Chloroquine / Azithromycin	RCT in Asymptomatic Volunteers With COVID-19 Comparing Azithromycin and Hydroxychloroquine vs. Hydroxychloroquine Alone vs Standard of Care Without Antibiotics	https://clinicaltrials.gov/show/NCT04374552
NCT04375046	830	ACE2 Receptors -Like Enzyme	A Randomized, Open Label, Controlled Clinical Study to Evaluate the Recombinant Bacterial ACE2 Receptors -Like Enzyme 2 (rbACE2) in Adult Patients With COVID-19	https://clinicaltrials.gov/show/NCT04375046
NCT04374903	831	Chloroquine/Azithromycin or Sirolimus	Hydroxychloroquine in Combination With Azithromycin or Sirolimus for Treating COVID-19 Patients: A Pilot, Multicenter Randomized Open-Label Trial	https://clinicaltrials.gov/show/NCT04374903
NCT04377659	832	Tocilizumab	A Phase II Study of IL-6 Receptor Antagonist Tocilizumab to Prevent Respiratory Failure and Death in Patients With Severe COVID-19 Infection	https://clinicaltrials.gov/show/NCT04377659
NCT04374019	833	Chloroquine /azithromycin / ivermectin / camostat	Randomized, Multi-arm Phase II Trial of Novel Agents for Treatment of High-risk COVID-19 Positive Patients	https://clinicaltrials.gov/show/NCT04374019
NCT04379492	834	Chloroquine	Single-center, Phase II, Randomized Double-blind, Placebo-controlled Study of Hydroxychloroquine Compared to Placebo as Treatment for Severe Acute Respiratory Syndrome Coronavirus 2 (SARS-CoV-2) Infection	https://clinicaltrials.gov/show/NCT04379492
NCT04381377	835	Polyoxidonium	A Multi-centre, Adaptive, Randomized, Double-blind, Placebo-controlled Comparative Clinical Study of the Safety and Efficacy of Polyoxidonium¬Æ, Lyophilizate for Solution for Injections and Topical Application, 6 mg (NPO Petrovax Pharm LLC, Russia) in Patients With Coronavirus Disease (COVID-19).	https://clinicaltrials.gov/show/NCT04381377
NCT04372680	836	Weaning	WeanINg From Mechanical Ventilation for ARDS CovId-19 Patients Guided by Combined Thoracic UltraSound: a Prospective, Multicenter, Randomized, Open-label, Parallel-group Clinical Trial	https://clinicaltrials.gov/show/NCT04372680
NCT04374539	837	Convalescent Plasma	Plasma Exchange in Patients With COVID-19 Disease and Invasive Mechanical Ventilation: a Randomized Controlled Trial	https://clinicaltrials.gov/show/NCT04374539
NCT04379336	838	BCG Vaccine	Reducing Morbidity and Mortality in Health Care Workers Exposed to SARS-CoV-2 by Enhancing Non-specific Immune Responses Through Bacillus Calmette-Gu√©rin Vaccination, a Randomized Controlled Trial	https://clinicaltrials.gov/show/NCT04379336
ChiCTR2000032769	839	Azvudine	A randomized,double blinded, parallel controlled clinical trial for azvudine in the treatment of novel coronavirus pneumonia (COVID-19)	http://www.chictr.org.cn/showproj.aspx?proj=53368
NCT04375098	840	Convalescent Plasma	Efficacy and Safety of Early Anti-SARS-COV-2 Convalescent Plasma in Patients Admitted for COVID-19 Infection: a Randomized Phase II Trial	https://clinicaltrials.gov/show/NCT04375098
NCT04377620	841	Ruxolitinib	A Phase 3, Randomized, Double-Blind, Placebo-Controlled, Multicenter Study to Assess the Efficacy and Safety of Ruxolitinib in Participants With COVID-19-Associated ARDS Who Require Mechanical Ventilation (RUXCOVID-DEVENT)	https://clinicaltrials.gov/show/NCT04377620
NCT04380519	842	Olokizumab and RPH-104	An International, Multicenter, Randomized, Double-blind, Adaptive Placebo-controlled Study of the Efficacy and Safety of a Single Administration of Olokizumab and RPH-104 With Standard Therapy in Patients With Severe SARS-CoV-2 Infection (COVID-19)	https://clinicaltrials.gov/show/NCT04380519
NCT04375202	843	Colchicine	Treatment With COLchicine of Patients Affected by COVID-19: a Pilot Study	https://clinicaltrials.gov/show/NCT04375202
NCT04377750	844	Tocilizumab	The Use of Tocilizumab in the Management of Patients Who Have Severe COVID-19 With Suspected Pulmonary Hyperinflammation	https://clinicaltrials.gov/show/NCT04377750
NCT04380961	845	Sirukumab	Phase 2, Randomized, Double-blind, Placebo-controlled Study to Evaluate the Efficacy and Safety of Sirukumab in Confirmed Severe or Critical COVID-19 Disease	https://clinicaltrials.gov/show/NCT04380961
NCT04374032	846	Metenkefalin / tridecactide	An Open-label, Prospective, Randomized, Comparative Clinical Trial to Evaluate the Efficacy and Safety of ENKORTEN¬Æ as an Immunomodulatory Therapy, Within the Usual Therapeutically Established Protocol, for the Treatment of Patients With Moderate to Severe COVID-19 Infection	https://clinicaltrials.gov/show/NCT04374032
NCT04381923	847	High flow/ CPAP helmet	COVIDNOCHE Trial (HFNO Versus CPAP Helmet) in COVID-19 Pneumonia	https://ClinicalTrials.gov/show/NCT04381923
NCT04382586	848	Zanubrutinib	Covid-19 Infection and Pulmonary Distress Treatment With Zanubrutinib in Hospitalized Participants	https://ClinicalTrials.gov/show/NCT04382586
NCT04382651	849	MAS825	Study of Efficacy and Safety of MAS825 in Patients With COVID-19	https://ClinicalTrials.gov/show/NCT04382651
NCT04381858	850	Convalescent plasma / Human immunoglobulin	Convalescent Plasma vs Human Immunoglobulin to Treat COVID-19 Pneumonia	https://ClinicalTrials.gov/show/NCT04381858
NCT04371107	851	Azithromycin	Proactive Care of Ambulatory COVID19 Patients	https://ClinicalTrials.gov/show/NCT04371107
NCT04382066	852	Plitidepsin	Proof of Concept Study to Evaluate the Safety Profile of Plitidepsin in Patients With COVID-19	https://ClinicalTrials.gov/show/NCT04382066
NCT04381884	853	Ivermectin	Ivermectin Effect on SARS-CoV-2 Replication in Patients With COVID-19	https://ClinicalTrials.gov/show/NCT04381884
NCT04382755	854	Zilucoplan	Zilucoplan¬Æ in Improving Oxygenation and Short- and Long-term Outcome of COVID-19 Patients With Acute Hypoxic Respiratory Failure	https://ClinicalTrials.gov/show/NCT04382755
NCT04383002	855	Nitric Oxide	High Dose Inhaled Nitric Oxide for COVID-19 (ICU Patients)	https://ClinicalTrials.gov/show/NCT04383002
NCT04382625	856	Chloroquine	Hydroxychloroquine in SARS-CoV-2 (COVID-19) Pneumonia Trial	https://ClinicalTrials.gov/show/NCT04382625
NCT04382053	857	DFV890	Study of Efficacy and Safety of DV890 in Patients With COVID-19 Pneumonia	https://ClinicalTrials.gov/show/NCT04382053
NCT04382131	858	Hypertonic NaCl nasal irrigation and gargling	Hypertonic Saline Nasal Irrigation and Gargling in Suspected or Confirmed COVID-19	https://ClinicalTrials.gov/show/NCT04382131
NCT04382924	859	NP-120 (Ifenprodil)	Safety and Efficacy of NP-120 (Ifenprodil) for the Treatment of Confirmed COVID-19 Infected Hospitalized Patients	https://ClinicalTrials.gov/show/NCT04382924
NCT04382391	860	Vagus nerve stimulation	Study Assessing Vagus Nerve Stimulation in CoViD-19 Respiratory Symptoms	https://ClinicalTrials.gov/show/NCT04382391
NCT04367168	861	Colchicine	Colchicine Twice Daily During 10 Days as an Option for the Treatment of Symptoms Induced by Inflammation in Patients With Mild and Severe Coronavirus Disease	https://ClinicalTrials.gov/show/NCT04367168
NCT04381871	862	Acacia Senegal / Pectin	Potential Role of Gum Arabic as Immunomodulatory Agent Among COVID 19 Patients	https://ClinicalTrials.gov/show/NCT04381871
NCT04382040	863	ArtemiC	A Phase II, Controlled Clinical Study Designed to Evaluate the Effect of ArtemiC in Patients Diagnosed With COVID-19	https://ClinicalTrials.gov/show/NCT04382040
NCT04381988	864	Chloroquine	A Study of Hydroxychloroquine vs Placebo to Prevent COVID-19 Infection in Patients Receiving Radiotherapy	https://ClinicalTrials.gov/show/NCT04381988
jRCTs031200026	865	Nafamostat Mesilate	Combination therapy of Favipiravir and Nafamostat Mesilate in Patients with COVID-19 Pneumonia	https://rctportal.niph.go.jp/en/detail?trial_id=jRCTs031200026
IRCT20200329046892N1	866	Trifluoperazine	Evaluation of Trifluoperazine Effectiveness in Treatment Process, Survival rate and Cure rate of COVID-19 Patients	https://www.irct.ir/trial/47300
IRCT20180114038350N3	867	Deferiprone	Investigating the effect of Deferiprone on the improvement of symptoms of Coronavirus 2019 (COVID 19)	https://www.irct.ir/trial/47475
IRCT20200418047121N1	868	Vitamin C / metformin / azithromycin / doxycyclin	Study of the effectiveness of concomitant use of vitamin C, metformin, azithromycin and doxycycline on improving patients with COVID-19	https://www.irct.ir/trial/47578
IRCT20200408046988N1	869	Melatonin	Evaluation of the efficacy of melatonin tablets as auxiliary medication in accelerating the improvement of the COVID-19 symptoms and clinical findings: A double-blind randomized and placebo controlled trial	https://www.irct.ir/trial/47142
IRCT20160131026298N3	870	Algro	Evaluation of the effect of Iranian medicine product, Algro, on the symptoms of patients with covid-19 (Coronavirus 19 disease)	https://www.irct.ir/trial/47019
IRCT20121216011763N46	871	Curcumin	Effect of curcumin-piperine supplementation on disease duration, severity and clinical signs, and inflammatory factors in patients with coronavirus (COVID-19)	https://www.irct.ir/trial/47529
IRCT20160706028815N5	872	Selenium	The effect of addition of selenium to treatment regimen of hospitalized patients with COVID-19 on the outcome of disease compared with control group	https://www.irct.ir/trial/47682
IRCT20200326046868N1	873	Propolis	The effect of propolis supplementation on clinical manifestations and inflammatory biomarkers in patients with COVID-19	https://www.irct.ir/trial/47934
IRCT20171213037866N2	874	Jujube	Efficacy of jujube product in accelerating the recovery process of Covid -19 patients	https://www.irct.ir/trial/47684
IRCT20131215015805N2	875	Levamisole	Assessment of effect of levamisole on pneumonia caused by COVID-19	https://www.irct.ir/trial/46887
IRCT20140305016852N4	876	Vitamin C / vitamin D	Comparison of three methods of treatment in patients with severe acute respiratory syndrome coronavirus 2 (SARS-CoV-2) with and without coronavirus positive test (Kovid-19)	https://www.irct.ir/trial/47508
IRCT20200413047063N1	877	Stem cells	Placental Mesenchymal Stem cells for treatment of ARDS in Coronavirus infection, Phase 1 and 2 Clinical Trials	https://www.irct.ir/trial/47156
IRCT20200418047122N1	878	Apocynin, niacin, tannin	Evaluation of the protective effect of a combination drug including Apocynin, Niacin, and Tannin on prevention of cardiovascular and respiratory morbidities and mortality in COVID-19 infection: A Clinical Trial	https://www.irct.ir/trial/47268
IRCT20200427047215N1	879	Indomethacin	Efficacy and safety of oral indomethacin for treatment of covid 19 induced pneumonia	https://www.irct.ir/trial/47520
IRCT20200501047258N1	880	Convalescent Plasma	Investigation of the effects of COVID-19 convalescent plasma in acute respiratory distress syndrome due to COVID-19	https://www.irct.ir/trial/47629
IRCT20200418047116N1	881	IVIG / Lopinavir/ritonavir	Effect of Intravenous immunoglobulin (IVIG) versus Kaletra (lopinavir and ritonavir) tablets in patients with acute respiratory infection (COVID-19): A clinical trial studies	https://www.irct.ir/trial/47219
IRCT20080901001165N50	882	Azithromycin	Investigating the efficacy and safety of Azithromycin inhaled spray in controlling the symptoms of patients with COVID-19	https://www.irct.ir/trial/47576
IRCT20080901001165N51	883	Chloroquine	Investigating the efficacy and safety of Hydroxychloroquine nasal spray in controlling the symptoms of patients with COVID-19	https://www.irct.ir/trial/47577
IRCT20080901001165N52	884	Methylprednisolone	Investigating the efficacy of high dose of glucocorticoid in patients with moderate to severe pneumonia related to COVID-19	https://www.irct.ir/trial/47641
IRCT20131129015584N2	885	Herbal medicine	Evaluation of the effect of herbal oral product (thyme syrup) in patients with COVID-19	https://www.irct.ir/trial/47639
IRCT20200406046968N3	886	INF-Beta	effect of Interfron-beta1(zifron) on clinical improvement and prognosis of COVID-19 in Tabriz Imam-Reza hospital	https://www.irct.ir/trial/47152
IRCT20150808023559N21	887	Convalescent Plasma	The Effect of Convalescent Plasma Therapy on the Outcomes of Patients with 19-COVID	https://www.irct.ir/trial/47594
IRCT20200426047212N1	888	Thyme oil	The Effect of Inhalation of Thyme Oil with Standard Nationwide Medication on Respiratory Markers and Laboratory Findings in Patients with COVID-19	https://www.irct.ir/trial/47572
IRCT20151222025660N2	889	Chloroquine	The prophylactic effect of Hydroxychloroquine on Novel Corona virus (COVID-19) in health care providers	https://www.irct.ir/trial/47319
IRCT20190618043923N4	890	ozone autohemotherapy	Investigation the effects medical Ozone Autohemotherapy on clinical and para clinical features of patients with Covid19	https://www.irct.ir/trial/47363
RBR-949z6v	891	Enoxaparin	Full versus prophylactic anticoagulation for the treatment of severe forms of Covid-19:	http://www.ensaiosclinicos.gov.br/rg/RBR-949z6v/
RBR-3k4wxb	892	Chloroquine / azithromycin	Evaluation of the use of Hydroxychlorochine in patients with discrete form of Covid-19: randomized clinical trial	http://www.ensaiosclinicos.gov.br/rg/RBR-3k4wxb/
RBR-3rdhgm	893	Dialysis	The role of early dialysis in the evolution of patients with COVID19	http://www.ensaiosclinicos.gov.br/rg/RBR-3rdhgm/
LBCTR2020043495	894	Chloroquine / azithromycin / lopinavir/ritonavir	An international randomised trial of additional treatments for COVID-19 in hospitalised patients who are all receiving the local standard of care	http://lbctr.moph.gov.lb/LBCTR/Trials/Details/3498
NCT04383613	895	Prone position	Prone Positioning for Patients on General Medical Wards With COVID19	https://clinicaltrials.gov/show/NCT04383613
NCT04383574	896	Vaccine inactivated	Safety and Immunogenicity Study of Inactivated Vaccine for Prevention of SARS-CoV-2 Infection(COVID-19)	https://clinicaltrials.gov/show/NCT04383574
NCT04389359	897	Chloroquine	PROphylaxis for paTiEnts at Risk of COVID-19 infecTion	https://clinicaltrials.gov/show/NCT04389359
NCT04386252	898	Dendritic Cell Vaccine	Phase Ib-II Trial of Dendritic Cell Vaccine to Prevent COVID-19 in Frontline Healthcare Workers and First Responders	https://clinicaltrials.gov/show/NCT04386252

Registry entry ID	Included ID	Intervention	Title	Webpage
NCT04384445	899	Organicell	Organicell Flow for Patients With COVID-19	https://clinicaltrials.gov/show/NCT04384445
NCT04386616	900	Astegolimab	A Study to Evaluate the Safety and Efficacy of MSTT1041A (Astegolimab) or UTTR1147A in Patients With Severe COVID-19 Pneumonia	https://clinicaltrials.gov/show/NCT04386616
NCT04390022	901	Ivermectin	Sars-CoV-2/COVID-19 Ivermectin Navarra-ISGlobal Trial	https://clinicaltrials.gov/show/NCT04390022
NCT04390503	902	Convalescent Plasma	Convalescent Plasma for COVID-19 Close Contacts	https://clinicaltrials.gov/show/NCT04390503
NCT04384380	903	Chloroquine	Efficacy and Tolerability of Hydroxychloroquine in Adult Patients With COVID-19	https://clinicaltrials.gov/show/NCT04384380
NCT04385043	904	Convalescent Plasma	Hyperimmune Plasma in Patients With COVID-19 Severe Infection	https://clinicaltrials.gov/show/NCT04385043
NCT04385199	905	Convalescent Plasma	Convalescent Plasma for Patients With COVID-19	https://clinicaltrials.gov/show/NCT04385199
NCT04385771	906	Cytokine Adsorption in ECMO	Cytokine Adsorption in Patients With Severe COVID-19 Pneumonia Requiring Extracorporeal Membrane Oxygenation	https://clinicaltrials.gov/show/NCT04385771
NCT04386447	907	Oxytocin	Phase II RCT to Assess Efficacy of Intravenous Administration of Oxytocin in Patients Affected by COVID-19	https://clinicaltrials.gov/show/NCT04386447
NCT04387760	908	Chloroquine / favipiravir	Favipiravir vs Hydroxychloroquine in COVID -19	https://clinicaltrials.gov/show/NCT04387760
NCT04388683	909	Nitric Oxide	The NO-COVID Study	https://clinicaltrials.gov/show/NCT04388683
NCT04390061	910	Tofacitinib	TOFAcitinib Plus Hydroxycloroquine vs Hydroxycloroquine in Patients With COVID-19 Interstitial Pneumonia	https://clinicaltrials.gov/show/NCT04390061
NCT04390191	911	CPAP	Early CPAP in COVID-19 Confirmed or Suspected Patients	https://clinicaltrials.gov/show/NCT04390191
NCT04390464	912	Ravulizumab / baricitinib	mulTi-Arm Therapeutic Study in Pre-ICu Patients Admitted With Covid-19 - Repurposed Drugs (TACTIC-R)	https://clinicaltrials.gov/show/NCT04390464
NCT04390477	913	Probiotic	Study to Evaluate the Effect of a Probiotic in COVID-19	https://clinicaltrials.gov/show/NCT04390477
NCT04390594	914	Azithromycin	Efficacy and Safety Evaluation of Treatment Regimens in Adult COVID-19 Patients in Senegal	https://clinicaltrials.gov/show/NCT04390594
NCT04383535	915	Convalescent Plasma	Convalescent Plasma and Placebo for the Treatment of COVID-19 Severe Pneumonia	https://clinicaltrials.gov/show/NCT04383535
NCT04385264	916	Chloroquine	#StayHome: Early Hydroxychloroquine to Reduce Secondary Hospitalisation and Household Transmission in COVID-19	https://clinicaltrials.gov/show/NCT04385264
NCT04385940	917	Vitamin D	Vitamin D and COVID-19 Management	https://clinicaltrials.gov/show/NCT04385940
NCT04386694	918	Photobiomodulation Therapy	Photobiomodulation Therapy Combined With Static Magnetic Field in Patients With COVID-19	https://clinicaltrials.gov/show/NCT04386694
NCT04388410	919	Convalescent Plasma	Safety and Efficacy of Convalescent Plasma Transfusion for Patients With COVID-19	https://clinicaltrials.gov/show/NCT04388410
NCT04389411	920	Montelukast	The COvid-19 Symptom MOntelukast Trial	https://clinicaltrials.gov/show/NCT04389411
NCT04389450	921	Stem cells	Double-Blind, Multicenter, Study to Evaluate the Efficacy of PLX PAD for the Treatment of COVID-19	https://clinicaltrials.gov/show/NCT04389450
NCT04389840	922	Heparin	Dociparstat for the Treatment of Severe COVID-19 in Adults at High Risk of Respiratory Failure	https://clinicaltrials.gov/show/NCT04389840
NCT04390139	923	Stem cells	Efficacy and Safety Evaluation of Mesenchymal Stem Cells for the Treatment of Patients With Respiratory Distress Due to COVID-19	https://clinicaltrials.gov/show/NCT04390139
NCT04390152	924	Stem cells	Safety and Efficacy of Intravenous Wharton's Jelly Derived Mesenchymal Stem Cells in Acute Respiratory Distress Syndrome Due to COVID 19	https://clinicaltrials.gov/show/NCT04390152
NCT04390217	925	LB1148	LB1148 for Pulmonary Dysfunction Associated With COVID-19 Pneumonia	https://clinicaltrials.gov/show/NCT04390217
NCT04385186	926	Convalescent Plasma	Inactivated Convalescent Plasma as a Therapeutic Alternative in Patients CoViD-19	https://clinicaltrials.gov/show/NCT04385186
NCT04384900	927	Prone Position	Accelerated Prone Position Ventilation of Patients With COVID-19	https://clinicaltrials.gov/show/NCT04384900
NCT04389801	928	Desferal	Use of Desferal for Prevention of ARDS in Hospitalised Cases Documented With Covid 19 Infection	https://clinicaltrials.gov/show/NCT04389801
NCT04385849	929	N-803	Study of the Safety of Therapeutic Treatment With an Immunomodulary Agent (N-803 in Adults With COVID-19	https://clinicaltrials.gov/show/NCT04385849
NCT04387240	930	Artemisinin	Evaluating the Efficacy of Artesunate in Adults With Mild Symptoms of COVID-19	https://clinicaltrials.gov/show/NCT04387240
NCT04389580	931	Vitamin A / tamoxifen	Combination Therapy With Isotretinoin and Tamoxifen May Provide Complete Protection Against Severe Acute Respiratory Syndrome Coronavirus	https://clinicaltrials.gov/show/NCT04389580
CTRI/2020/05/025049	932	Homeopathy	A CLINICAL TRIAL TO STUDY THE EFFICACY OF HOMOEOPATHIC MEDICINE IN PREVENTION AND CURE OF CORONA VIRUS DISEASE 19	http://www.ctri.nic.in/Clinicaltrials/pmaindet2.php?trialid=43313
ISRCTN40580903	933	Gemtuzumab	Which treatment could lessen the severity of a coronavirus infection when compared with usual care in an NHS setting?	http://isrctn.com/ISRCTN40580903
CTRI/2020/05/025022	934	Chloroquine	Hydroxychloroquine in patients with mild COVID-19 illness with risk factors for severe disease	http://www.ctri.nic.in/Clinicaltrials/pmaindet2.php?trialid=43183
CTRI/2020/05/025093	935	Yashtimadhu	Study of effect of Yashtimadhu tablet for the prevention of COVID -19 on healthy individuals.	http://www.ctri.nic.in/Clinicaltrials/pmaindet2.php?trialid=43539
PACTR202005681895696	936	Vaccine ChAdOx1	A study to determine if a new COVID-19 vaccine safely generates protective immune responses in adults in Kenya	https://pactr.samrc.ac.za/TrialDisplay.aspx?TrialID=10988
PACTR202005599385499	937	Folic acid	Folic acid: A new suggested prevention and treatment of COVID-19 infection.	https://pactr.samrc.ac.za/TrialDisplay.aspx?TrialID=10971
ACTRN12620000566932	938	Chloroquine	Randomised clinical trial of interventions for the treatment of COVID-19 in the community setting for high risk older people.	https://anzctr.org.au/ACTRN12620000566932.aspx
IRCT20200510047383N1	939	Tocilizumab	Evaluation of the effect of Tocilizumab on outcomes of the severe COVID-19 patients	http://en.irct.ir/trial/48024
IRCT20120215009014N354	940	Hydrocortisone, methylprednisolone, and dexamethasone	Evaluating the effect of intravenous hydrocortisone, methylprednisolone, and dexamethasone in treatment of patients with moderate to severe acute respiratory distress syndrome caused by COVID-19	http://en.irct.ir/trial/48043
IRCT20200426047206N1	941	Biosafety	The effect of NBS powder in treatment of patients with COVID-19	http://en.irct.ir/trial/47495
IRCT20200428047228N1	942	Favipiravir	Evaluation of the efficiency and safety of favipiravir + hydroxychloroquine drug regimen in comparison with hydroxychloroquine in hospitalized patients with covid-19	http://en.irct.ir/trial/47559
IRCT20180425039414N2	943	Zinc	The effect of zinc on the treatment and clinical course of patients with SARS-cov2 (COVID-19)	http://en.irct.ir/trial/47516
IRCT20080901001165N53	944	INF-Beta	Investigating the efficacy and safety of Interferon Beta1a nasal spray in controlling the symptoms of patients with COVID-19	http://en.irct.ir/trial/48066
IRCT20200508047346N1	945	CoviGlobulin	Evaluation of the effectiveness of rabbit antibody against coronavirus in patients	http://en.irct.ir/trial/47953
IRCT20200418047126N1	946	Colchicine	Evaluation of colchicine in treatment of coronavirus	http://en.irct.ir/trial/47707
IRCT20200421047150N1	947	Stem cells	Stem cell treatment for COVID-19	http://en.irct.ir/trial/47964
SLCTR/2020/011	948	Chloroquine	Hydroxychloroquine for Post-Exposure Prophylaxis of COVID-19 among naval personnel: a placebo-controlled, randomized, clinical trial	https://slctr.lk/trials/slctr-2020-011
TCTR20200514001	949	Favipiravir	An Investigation of the Efficacy and Safety of Favipiravir in COVID-19 Patients without Pneumonia √¢?? An open-label randomized controlled study √¢??	http://www.clinicaltrials.in.th/index.php?tp=regtrials&menu= trialsearch&smenu= fulltext&task=search&task2=
CTRI/2020/04/024948	950	Chloroquine / Ciclesonide / Ivermectin	A clinical Trial to Study the Effects of Hydroxychloroquine, Ciclesonide and Ivermectin in treatment of moderate COVID-19 illness	http://www.ctri.nic.in/Clinicaltrials/pmaindet2.php?trialid=43364
CTRI/2020/04/024949	951	Niclosamide	A study to evaluate the effect of Oral Niclosamide in mild and very mild COVID-19 cases	http://www.ctri.nic.in/Clinicaltrials/pmaindet2.php?trialid=43400
CTRI/2020/05/025178	952	Ayurveda	Evaluation of the Immuno-Stimulatory Potential (Shareera Bala) of an Ayurveda Management Protocol in Cohort of Quarantined Delhi Police under Covid-19 Care Centers- An Exploratory Clinical Study	http://www.ctri.nic.in/Clinicaltrials/pmaindet2.php?trialid=43557
CTRI/2020/04/024904	953	Azithromycin	Treatment of COVID19 : A randomised controlled trial	http://www.ctri.nic.in/Clinicaltrials/pmaindet2.php?trialid=43130
CTRI/2020/05/025067	954	Chloroquine	A randomized controlled trial of hydroxychloroquine prophylaxis for Healthcare Workers exposed to COVID-19	http://www.ctri.nic.in/Clinicaltrials/pmaindet2.php?trialid=43019
CTRI/2020/05/024981	955	Dabur Chyawanprash	Clinical study on Dabur Chyawanprash as a preventive remedy in pandemic of Covid-19.	http://www.ctri.nic.in/Clinicaltrials/pmaindet2.php?trialid=43374
CTRI/2020/05/025071	956	Ventilator settings	Clinical trial evaluating two ventilator settings in Corona virus related severe lung diseases (known as ARDS)	http://www.ctri.nic.in/Clinicaltrials/pmaindet2.php?trialid=43447
CTRI/2020/05/024962	957	Povidone Iodine	The use of topical Povidone Iodine (PVP-I) oropharyngeal and intranasal application during the current coronavirus pandemic as a potential measure to reduce viral transmission	http://www.ctri.nic.in/Clinicaltrials/pmaindet2.php?trialid=43237
CTRI/2020/05/025114	958	Favipiravir	A Clinical Study on Favipiravir Compared to Standard Supportive Care in Patients With Mild to Moderate COVID-19.	http://www.ctri.nic.in/Clinicaltrials/pmaindet2.php?trialid=43504
CTRI/2020/05/025156	959	Ayurveda	Effect of Ayurveda intervention AYUSH-64 add-on therapy for patients with COVID-19 infection (Stage I)	http://www.ctri.nic.in/Clinicaltrials/pmaindet2.php?trialid=43590
CTRI/2020/05/025068	960	Ivermectin	Can a medicine help in curing viral infection	http://www.ctri.nic.in/Clinicaltrials/pmaindet2.php?trialid=43449
CTRI/2020/05/024959	961	Itolizumab	Efficacy and Safety of Itolizumab in COVID-19 Complications	http://www.ctri.nic.in/Clinicaltrials/pmaindet2.php?trialid=42878
CTRI/2020/04/024947	962	Homeopathy	Clinical trial on effects of homeopathic medicine made from cadamba on COVID-19	http://www.ctri.nic.in/Clinicaltrials/pmaindet2.php?trialid=43259
CTRI/2020/05/025161	963	Herbal medicine	To study the Effectiveness of herbal formulation - Aayudh Advance as a supplementary treatment for the Corona Virus 2019 (Covid-19) infected patients	http://www.ctri.nic.in/Clinicaltrials/pmaindet2.php?trialid=43442
CTRI/2020/05/024969	964	Homeopathy	Homoeopathy as adjuvant in management of Covid-19 infection	http://www.ctri.nic.in/Clinicaltrials/pmaindet2.php?trialid=43393
ChiCTR2000032915	965	Artemisinin-pipequine	A randomized controlled trial for the efficacy and safety of artemisinin-pipequine tablets in the treatment of the mild and common type novel coronavirus pneumonia (COVID-19) patients whose nCoV Nucleic acid did not turn negative after treated by hydroxychloroquine and Abidor	http://www.chictr.org.cn/showproj.aspx?proj=53658
ChiCTR2000032919	966	Lu-Dang-Shen	A multicenter, randomized, double-blind, placebo-controlled trial for Lu-Dang-Shen oral liquid in the treatment of reduced digestion function of convalescent new coronavirus pneumonia (COVID-19) patient	http://www.chictr.org.cn/showproj.aspx?proj=53274
KCT0005003	967	Nafamostat Mesilate	Clinical efficacy of Nafamostat for COVID-19 pneumonia	http://cris.nih.go.kr/cris/en/search/search_result_st01.jsp?seq=16635
NCT04395976	968	Ayurveda	Ayurveda as Prophylaxis for Suspected COVID-19 Patients	https://clinicaltrials.gov/show/NCT04395976
NCT04386850	969	Vitamin D	Oral 25-hydroxyvitamin D3 and COVID-19	https://clinicaltrials.gov/show/NCT04386850
NCT04393415	970	Stem cells	Using Cord Blood in Treatment of Covid -19	https://clinicaltrials.gov/show/NCT04393415
NCT04391140	971	Prone position	Prone Positioning and High-flow Nasal Cannula in COVID-19 Induced ARDS	https://clinicaltrials.gov/show/NCT04391140
NCT04395768	972	Vitamin C	International ALLIANCE Study of Therapies to Prevent Progression of COVID-19	https://clinicaltrials.gov/show/NCT04395768
NCT04391127	973	Chloroquine / Ivermectin	Hydroxychloroquine and Ivermectin for the Treatment of COVID-19 Infection	https://clinicaltrials.gov/show/NCT04391127
NCT04394208	974	Silymarin	Silymarin in COVID-19 Pneumonia	https://clinicaltrials.gov/show/NCT04394208
NCT04392219	975	EIDD-2801	COVID-19 First In Human Study to Evaluate Safety, Tolerability, and Pharmacokinetics of EIDD-2801 in Healthy Volunteers	https://clinicaltrials.gov/show/NCT04392219
NCT04393311	976	Ulinastatin	Ulinastatin for the Treatment of COVID-19 in Hospitalized Patients	https://clinicaltrials.gov/show/NCT04393311
NCT04392141	977	Colchicine / Phenolic Monoterpenes	Colchicine Plus Phenolic Monoterpenes to Treat COVID-19	https://clinicaltrials.gov/show/NCT04392141
NCT04393727	978	Convalescent Plasma	Transfusion of Convalescent Plasma for the Early Treatment of Patients With COVID-19	https://clinicaltrials.gov/show/NCT04393727
NCT04395144	979	Prone position	COVid-19: Awake Proning and High-flow Nasal Cannula in respiratorY DistrEss	https://clinicaltrials.gov/show/NCT04395144
NCT04395170	980	Convalescent Plasma / Immunoglobulin	Convalescent Plasma Compared to Anti-COVID-19 Human Immunoglobulin and Standard Treatment (TE) in Hospitalized Patients	https://clinicaltrials.gov/show/NCT04395170
NCT04391101	981	Convalescent Plasma	Convalescent Plasma for the Treatment of Severe SARS-CoV-2 (COVID-19)	https://clinicaltrials.gov/show/NCT04391101
NCT04391712	982	Cold Laser Therapy	Cold Laser Therapy for COVID-19 Positive Patients With Pulmonary Disease	https://clinicaltrials.gov/show/NCT04391712
NCT04393051	983	Baricitinib	Baricitinib Compared to Standard Therapy in Patients With COVID-19	https://clinicaltrials.gov/show/NCT04393051
NCT04393246	984	EDP1815/Dapagliflozin/Ambrisentan	mulTi-Arm Therapeutic Study in Pre-ICu Patients Admitted With Covid-19 - Experimental Drugs and Mechanisms (TACTIC-E)	https://clinicaltrials.gov/show/NCT04393246
NCT04392713	985	Ivermectin	Efficacy of Ivermectin in COVID-19	https://clinicaltrials.gov/show/NCT04392713
NCT04396067	986	Vitamin A	Combination With Alvelestat and Isotretinoin May Enhances Neutralizing Antibodies in COVID -19 Infected Patients Better Than COVID-19 Inactivated Vaccines	https://clinicaltrials.gov/show/NCT04396067
NCT04394442	987	Chloroquine	Hydroxychloroquine in COVID-19 Patients	https://clinicaltrials.gov/show/NCT04394442
NCT04395105	988	Dexamethasone	Dexamethasone for COVID-19 Related ARDS: a Multicenter, Randomized Clinical Trial	https://clinicaltrials.gov/show/NCT04395105

Registry entry ID	Included ID	Intervention	Title	Webpage
NCT04388709	989	INF-Lambda	Interferon Lambda Therapy for COVID-19	https://clinicaltrials.gov/show/NCT04388709
NCT04392414	990	Convalescent Plasma	Hyperimmune Convalescent Plasma in Moderate and Severe COVID-19 Disease	https://clinicaltrials.gov/show/NCT04392414
NCT04392531	991	Cyclosporine	Clinical Trial to Assess Efficacy of cYclosporine Plus Standard of Care in Hospitalized Patients With COVID19	https://clinicaltrials.gov/show/NCT04392531
NCT04392973	992	Chloroquine / favipiravir	FAvipiravir and HydroxyChloroquine Combination Therapy	https://clinicaltrials.gov/show/NCT04392973
NCT04393948	993	Lung Irradiation	Lung Irradiation for COVID-19 Pneumonia	https://clinicaltrials.gov/show/NCT04393948
NCT04394416	994	Imatinib	Trial of Imatinib for Hospitalized Adults With COVID-19	https://clinicaltrials.gov/show/NCT04394416
NCT04392128	995	Chloroquine / Azithromycin	Study Evaluating the Efficacy of Hydroxychloroquine and Azithromycine in Patients With COVID-19 and Hematological Malignancies (HYACINTHE)	https://clinicaltrials.gov/show/NCT04392128
NCT04392778	996	Stem cells	Clinical Use of Stem Cells for the Treatment of Covid-19	https://clinicaltrials.gov/show/NCT04392778
NCT04391309	997	IC14	Phase 2 IC14 (Anti-CD14) Treatment in Patients With SARS-CoV-2 (COVID-19)	https://clinicaltrials.gov/show/NCT04391309
NCT04388826	998	Veru-111	COVID-19 Treatment of Severe Acute Respiratory Syndrome With Veru-111	https://clinicaltrials.gov/show/NCT04388826
NCT04394377	999	Rivaroxaban	Full Anticoagulation Versus Prophylaxis in COVID-19: COALIZAO ACTION Trial	https://clinicaltrials.gov/show/NCT04394377
NCT04394117	1000	Angiotensin Receptor Blockers	Controlled evaLuation of Angiotensin Receptor Blockers for COVID-19 respIraTorY Disease	https://clinicaltrials.gov/show/NCT04394117
NCT04395807	1001	Helmet CPAP	Helmet CPAP Versus HFNC in COVID-19	https://clinicaltrials.gov/show/NCT04395807
NCT04391179	1002	Dipyridamole	Dipyridamole to Prevent Coronavirus Exacerbation of Respiratory Status (DICER) in COVID-19	https://clinicaltrials.gov/show/NCT04391179
NCT04395456	1003	AMY-101	A Phase 2 Study of the C3 Inhibitor AMY-101 in Patients With ARDS Due to COVID-19 (SAVE)	https://clinicaltrials.gov/show/NCT04395456
NCT04393038	1004	ABX464	ABX464 in Treating Inflammation and Preventing Acute Respiratory Failure in Patients With COVID-19	https://clinicaltrials.gov/show/NCT04393038
NCT04396106	1005	AT-527	Safety and Efficacy of AT-527 in Subjects With Moderate Coronavirus Disease (COVID-19)	https://clinicaltrials.gov/show/NCT04396106
NCT04392427	1006	Nitazoxanide/Ribavirin/Ivermectin	New Antiviral Drugs for Treatment of COVID-19	https://clinicaltrials.gov/show/NCT04392427
NCT04399317	1007	Ventilator settings	Flow Controlled Ventilation in ARDS Associated With COVID-19	https://clinicaltrials.gov/show/NCT04399317
NCT04399980	1008	Mavrilimumab	Mavrilimumab to Reduce Progression of Acute Respiratory Failure in COVID-19 Pneumonia and Systemic Hyper-inflammation	https://clinicaltrials.gov/show/NCT04399980
NCT04399252	1009	Lactobacillus	Effect of Lactobacillus on the Microbiome of Household Contacts Exposed to COVID-19	https://clinicaltrials.gov/show/NCT04399252
NCT04397692	1010	Nitric Oxide	Inhaled NO for the Treatment of COVID-19 Caused by SARS-CoV-2 (US Trial)	https://clinicaltrials.gov/show/NCT04397692
NCT04397328	1011	Chloroquine	COVID-19 PEP- High-risk Individuals in Long-term and Specialized Care - Canada	https://clinicaltrials.gov/show/NCT04397328
NCT04400058	1012	Immunoglobulin	OCTAGAM 10% THERAPY IN COVID-19 PATIENTS WITH SEVERE DISEASE PROGRESSION	https://clinicaltrials.gov/show/NCT04400058
NCT04398147	1013	Vaccine Adenovirus Type 5 Vector	Phase I/II Clinical Trial of Recombinant Novel Coronavirus Vaccine (Adenovirus Type 5 Vector) (Ad5-nCoV) in Canada	https://clinicaltrials.gov/show/NCT04398147
NCT04397562	1014	Levilimab	A Clinical Trial of the Efficacy and Safety of Levilimab (BCD-089) in Patients With Severe COVID-19	https://clinicaltrials.gov/show/NCT04397562
NCT04397718	1015	Degarelix	Hormonal Intervention for the Treatment in Veterans With COVID-19 Requiring Hospitalization	https://clinicaltrials.gov/show/NCT04397718
NCT04398290	1016	Nitric Oxide	iNOPulse for COVID-19	https://clinicaltrials.gov/show/NCT04398290
NCT04398303	1017	Stem cells	ACT-20 in Patients With Severe COVID-19 Pneumonia	https://clinicaltrials.gov/show/NCT04398303
NCT04397757	1018	Convalescent Plasma	COVID-19 Convalescent Plasma for the Treatment of Hospitalized Patients With Pneumonia Caused by SARS-CoV-2.	https://clinicaltrials.gov/show/NCT04397757
NCT04397510	1019	Heparin	Nebulized Heparin for the Treatment of COVID-19 Induced Lung Injury	https://clinicaltrials.gov/show/NCT04397510
NCT04397497	1020	Mavrilimumab	Mavrilimumab in Severe COVID-19 Pneumonia and Hyper-inflammation (COMBAT-19)	https://clinicaltrials.gov/show/NCT04397497
NCT04397796	1021	Stem cells	Study of the Safety of Therapeutic Tx With Immunomodulatory MSC in Adults With COVID-19 Infection Requiring Mechanical Ventilation	https://clinicaltrials.gov/show/NCT04397796
NCT04399356	1022	Niclosamide	Niclosamide for Mild to Moderate COVID-19	https://clinicaltrials.gov/show/NCT04399356
EUCTR2020-001704-42-ES	1023	Chloroquine	Controlled and randomised trial to assess the safety and efficacy of hydroxychloroquine chemoprophylaxis in SARS CoV2 infection in hospital healthcare personnel (Sanitarios sin COVID-19).	https://www.clinicaltrialsregister.eu/ctr-search/search?query=eudract_number:2020-001704-42
EUCTR2020-001498-63-BE	1024	BIO101	Testing the Efficacy and Safety of BIO101, for the Prevention of Respiratory Deterioration, in Patients with COVID-19 Pneumonia (COVA study)	https://www.clinicaltrialsregister.eu/ctr-search/search?query=eudract_number:2020-001498-63
EUCTR2020-001807-18-GB	1025	TD-0903	TD-0903 for acute lung injury associated with COVID-19	https://www.clinicaltrialsregister.eu/ctr-search/search?query=eudract_number:2020-001807-18
EUCTR2020-001805-21-GB	1026	Oseltamivir / IMU-838	IMU-838 and Oseltamivir in treatment of Novel Coronavirus (COVID-19)	https://www.clinicaltrialsregister.eu/ctr-search/search?query=eudract_number:2020-001805-21
ISRCTN21363594	1027	Plasmapheresis	Therapeutic plasma exchange (removal of the non-cell portion of blood) in critically ill adult patients with serious SARS CoV-2 disease (COVID-19)	http://isrctn.com/ISRCTN21363594
ACTRN12620000588998	1028	STC3141	The safety and efficacy of STC3141 in patient with COVID-19 Acute Respiratory Distress Syndrome	https://anzctr.org.au/ACTRN12620000588998.aspx
ACTRN12620000580976	1029	Tocilizumab	Tocilizumab for tReatment Of COVID-19 in intensive cARe patients (‚ÄúTROCAR‚Äù)	https://anzctr.org.au/ACTRN12620000580976.aspx
ACTRN12620000612910	1030	Stem cells	The MEND (MEseNchymal coviD-19) Trial: a pilot study to investigate early efficacy of mesenchymal stem cells in adults with COVID-19	https://anzctr.org.au/ACTRN12620000612910.aspx
NCT04403100	1031	Chloroquine / Lopinavir/ Ritonavir	Hydroxychloroquine and Lopinavir/ Ritonavir to Improve the Health of People With COVID-19: "The Hope Coalition - 1"	https://clinicaltrials.gov/show/NCT04403100
NCT04402203	1032	Favipiravir	Study on Safety and Efficacy of Favipiravir (Favipira) for COVID-19 Patient in Selected Hospitals of Bangladesh	https://clinicaltrials.gov/show/NCT04402203
NCT04400929	1033	Sargramostim	Using GM-CSF as a Host Directed Therapeutic Against COVID-19	https://clinicaltrials.gov/show/NCT04400929
NCT04401579	1034	Remdesivir / baricitinib	Adaptive COVID-19 Treatment Trial 2 (ACTT-II)	https://clinicaltrials.gov/show/NCT04401579
NCT04402944	1035	Pulmozyme	Pulmozyme to Improve COVID-19 ARDS Outcomes	https://clinicaltrials.gov/show/NCT04402944
NCT04404634	1036	Convalescent Plasma	CONVALESCENT PLASMA TO LIMIT CORONAVIRUS ASSOCIATED COMPLICATIONS	https://clinicaltrials.gov/show/NCT04404634
NCT04404361	1037	Pacritinib	PRE-VENT Phase 3 Study in Hospitalized Patients With Severe COVID-19 With or Without Cancer	https://clinicaltrials.gov/show/NCT04404361
NCT04400799	1038	Enoxaparin	Enoxaparin for Primary Thromboprophylaxis in Ambulatory Patients With COVID-19	https://clinicaltrials.gov/show/NCT04400799
NCT04401202	1039	Nigella Sativa	Nigella Sativa in COVID-19	https://clinicaltrials.gov/show/NCT04401202
NCT04402879	1040	Prone position	CORONA: COvid pRONe hypoxemiA (CORONA)	https://clinicaltrials.gov/show/NCT04402879
NCT04403555	1041	Ivermectin / Doxycycline	Ivermectin and Doxycycine in COVID-19 Treatment	https://clinicaltrials.gov/show/NCT04403555
NCT04403243	1042	Colchicine / Ruxolitinib / Secukinumab	COLchicine Versus Ruxolitinib and Secukinumab In Open Prospective Randomized Trial	https://clinicaltrials.gov/show/NCT04403243
NCT04403477	1043	Convalescent Plasma	Convalescent Plasma Therapy in Severe COVID-19 Infection	https://clinicaltrials.gov/show/NCT04403477
NCT04403685	1044	Tocilizumab	Safety and Efficacy of Tocilizumab in COVID-19 and Inflammatory Markers: a Phase III Randomized Controlled Trial	https://clinicaltrials.gov/show/NCT04403685
NCT04400890	1045	Resveratrol	Randomized Proof-of-Concept Trial to Evaluate the Safety and Explore the Effectiveness of Resveratrol for COVID-19	https://clinicaltrials.gov/show/NCT04400890
NCT04401150	1046	Vitamin C	Lessening Organ Dysfunction With VITamin C - COVID-19	https://clinicaltrials.gov/show/NCT04401150
NCT04402957	1047	LSALT Peptide	LSALT Peptide vs. Placebo to Prevent ARDS and Other Organ Injuries in Patients Infected With SARS-CoV-2 (COVID-19)	https://clinicaltrials.gov/show/NCT04402957
NCT04401527	1048	Sodium Nitrite	Treatment of Lung Injury From COVID-19 Infection With Intravenous Sodium Nitrite	https://clinicaltrials.gov/show/NCT04401527
NCT04404218	1049	Acai Berry	The A√ßa√≠ Berry COVID-19 Anti-Inflammation Trial	https://clinicaltrials.gov/show/NCT04404218
NCT04401423	1050	Angiotensin 1-7	TXA COVID-19 Clinical Trial	https://clinicaltrials.gov/show/NCT04401423
NCT04401293	1051	Heparin	Full Dose Heparin Vs. Prophylactic Or Intermediate Dose Heparin in High Risk COVID-19 Patients	https://clinicaltrials.gov/show/NCT04401293
NCT04402060	1052	APL-9	A Study of APL-9 in Adults With Mild to Moderate ARDS Due to COVID-19	https://clinicaltrials.gov/show/NCT04402060
NCT04403646	1053	Tannin	Tannin Specific Natural Extract for COVID-19 Infection	https://clinicaltrials.gov/show/NCT04403646
NCT04404426	1054	Citrulline	CACOLAC : Citrulline Administration in the Hospital Patient in Intensive Care for COVID-19 Acute Respiratory Distress Syndrome	https://clinicaltrials.gov/show/NCT04404426
NCT04400838	1055	Vaccine ChAdOx1	Investigating a Vaccine Against COVID-19	https://clinicaltrials.gov/show/NCT04400838
NCT04401475	1056	EB05	A Randomized, Double-Blind, Placebo-Controlled Study to Evaluate the Safety and Efficacy of EB05 + SOC vs. Placebo + SOC in Adult Hospitalized Patients With Moderate to Severe COVID-19 Pneumonia	https://clinicaltrials.gov/show/NCT04401475
IRCT20160809029275N1	1057	Stem cells	stem cell therapy in Covid-19	http://en.irct.ir/trial/48187
IRCT20200411047019N1	1058	BCG Vaccine	Effect of BCG Vaccine on Preventing COVID-19	http://en.irct.ir/trial/47279
IRCT20080901001165N60	1059	Provita	Investigating the efficacy of Provita Capsule in controlling the symptoms of patients with COVID-19	http://en.irct.ir/trial/48102
IRCT20091012002582N21	1060	Intrapulmonary injection of processed COVID serum	Effect of intrapulmonary injection of processed serum derived from covid-19 patients in reduction of pulmonary complications	http://en.irct.ir/trial/47197
IRCT20200428047228N2	1061	Azithromycin	Hydroxychloroquine in comparison with hydroxychloroquine + azithromycin in patients with covid-19	http://en.irct.ir/trial/48096
IRCT20200209046427N1	1062	Honey	Effect of bee products on COVID-19	http://en.irct.ir/trial/47504
IRCT20080901001165N59	1063	Mouthwash	Investigating the efficacy and safety of Niwasha mouthwash in controlling the symptoms of patients with COVID-19	http://en.irct.ir/trial/47477
IRCT20080901001165N55	1064	N-Acetyl Cysteine	Investigating the efficacy and safety of N-Acetyl Cysteine (NAC) inhalation spray in controlling the symptoms of patients with COVID-19	http://en.irct.ir/trial/48061
IRCT20080901001165N56	1065	Curcumin	Evaluation efficacy of "Curcumin and Resveratrol" capsule in controlling symptoms in patients with COVID-19	http://en.irct.ir/trial/48116
IRCT20080901001165N58	1066	Plasmapheresis	Investigating the efficacy and safety of Plasmapheresis in patients with Moderate to severe COVID-19	http://en.irct.ir/trial/48394
IRCT20200502047268N1	1067	GSCF	Evaluation of the effect of GCSF injection in treatment of COVID-19 patients with lymphopenia	http://en.irct.ir/trial/47692
IRCT20081027001411N3	1068	Prednisolone	Effect of Prednisolone on treatment of COVID-19	http://en.irct.ir/trial/46975
IRCT20200412047042N1	1069	Raltegravir / interferon-beta	Evaluation the effect of raltegravir, and raltegravir/interferon beta combination on covid 19 patients	http://en.irct.ir/trial/48749
IRCT20200422047168N2	1070	Ivermectin	Clinical trial study of the therapeutic effect of Ivermectin, besides Kaletra and Chloroquinein in patients with Coronavirus disease 2019 (COVID-19)	http://en.irct.ir/trial/48444
IRCT20091201002804N12	1071	Surfactant	The effect of surfactant on clinical outcome of patients with COVID-19	http://en.irct.ir/trial/46983
IRCT20200314046774N1	1072	Vitamin A	treatment of COVID-19 whit vitamin A	http://en.irct.ir/trial/46519
DRKS00021732	1073	Valsartan	Treatment of Sars-CoV2 infections (Covid-19) with valsartan vs placebo, a three-armed randomized, partly blinded trial	http://www.drks.de/DRKS00021732
RPCEC00000315	1074	Immuno-1 / -2	Evaluation of the synergistic effect of sequential administration of two immunostimulant products: Immuno-1 and Immuno-2 for the stimulation of the immune response in older adults. Exploratory study	https://rpcec.sld.cu/en/trials/RPCEC00000315-En
RPCEC00000317	1075	CIGB 300	ATENEA-Co-300 study	https://rpcec.sld.cu/en/trials/RPCEC00000317-En
RPCEC00000320	1076	ozone autohemotherapy	Rectal Ozone in the COVID-19 treatment	https://rpcec.sld.cu/en/trials/RPCEC00000320-En
RPCEC00000316	1077	Dialysis	Exploratory study of BMT IM to stimulate immune response in patients with CKD-5 on iterated hemodialysis	https://rpcec.sld.cu/en/trials/RPCEC00000316-En
NCT04409834	1078	Heparin / clopidogrel / enoxaparin	Prevention of Arteriovenous Thrombotic Events in Critically-Ill COVID-19 Patients Trial	https://clinicaltrials.gov/show/NCT04409834

Registry entry ID	Included ID	Intervention	Title	Webpage
NCT04435795	1079	Ciclesonide	Inhaled Ciclesonide for Outpatients With COVID19	https://clinicaltrials.gov/show/NCT04435795
NCT04411628	1080	LY-CoV555	A Study of LY3819253 (LY-CoV555) in Participants Hospitalized for COVID-19	https://clinicaltrials.gov/show/NCT04411628
NCT04436276	1081	Ad26COVS1	A Study of Ad26COVS1 in Adults	https://clinicaltrials.gov/show/NCT04436276
NCT04414124	1082	KB109	A Clinical Study to Assess the Natural History of COVID-19 and Effects of KB109 and Supportive Self-care in Outpatients With Mild-to-moderate COVID-19	https://clinicaltrials.gov/show/NCT04414124
NCT04439045	1083	VPM1002 vaccine	Efficacy and Safety of VPM1002 in Reducing SARS-CoV-2 (COVID-19) Infection Rate and Severity	https://clinicaltrials.gov/show/NCT04439045
NCT04437693	1084	Chloroquine	Post Exposure Prophylaxis in Healthcare Workers Exposed to COVID-19 Patients	https://clinicaltrials.gov/show/NCT04437693
NCT04405999	1085	Bromhexine	Prevention of Infection and Incidence of COVID-19 in Medical Personnel Assisting Patients With New Coronavirus Disease	https://clinicaltrials.gov/show/NCT04405999
NCT04416048	1086	Rivaroxaban	Effect of Anticoagulation Therapy on Clinical Outcomes in COVID-19	https://clinicaltrials.gov/show/NCT04416048
NCT04422561	1087	Ivermectin	Prophylactic Ivermectin in COVID-19 Contacts	https://clinicaltrials.gov/show/NCT04422561
NCT04427865	1088	Lactoferrin	Utility of Lactoferrin as a Preventive Agent for Healthcare Workers Exposed to COVID-19	https://clinicaltrials.gov/show/NCT04427865
NCT04428008	1089	Thymosin alpha	Thymosin Alpha 1 to Prevent COVID-19 Infection in Elderly Renal Dialysis Patients	https://clinicaltrials.gov/show/NCT04428008
NCT04438837	1090	Chloroquine	Hydroxychloroquine Post-Exposure Prophylaxis for Coronavirus Disease (COVID-19) Among Health-Care Workers: A Randomized-Controlled Trial	https://clinicaltrials.gov/show/NCT04438837
NCT04405271	1091	Metricitabine	TAF/FTC for Pre-exposure Prophylaxis of COVID-19 in Healthcare Workers (CoviPrep Study)	https://clinicaltrials.gov/show/NCT04405271
NCT04409327	1092	Dactolisib	Phase 3 Study to Determine if RTB101 Reduces the Severity of COVID-19 in Older Adults Residing in Nursing Homes	https://clinicaltrials.gov/show/NCT04409327
NCT04412538	1093	COV-2 Vaccine	Safety and Immunogenicity Study of an Inactivated SARS-CoV-2 Vaccine for Preventing Against COVID-19	https://clinicaltrials.gov/show/NCT04412538
NCT04420260	1094	Immunostimulant spray	Primary Prevention of Infection by COVID-19 in Health Providers	https://clinicaltrials.gov/show/NCT04420260
NCT04414267	1095	BCG Vaccine	Bacillus Calmette-gu√©rin Vaccination to Prevent COVID-19	https://clinicaltrials.gov/show/NCT04414267
NCT04435314	1096	Nitazoxanide	Efficacy and Safety of Nitazoxanide for Post Exposure Prophylaxis of COVID-19	https://clinicaltrials.gov/show/NCT04435314
NCT04425031	1097	Oxygen target	Handling Oxygenation Targets in COVID-19	https://clinicaltrials.gov/show/NCT04425031
NCT04408183	1098	GLS1200 nasal spray	GLS-1200Topical Nasal Spray to Prevent SARS-CoV-2 Infection (COVID-19) in Health Care Personnel	https://clinicaltrials.gov/show/NCT04408183
NCT04409873	1099	Mouthwash	Antiseptic Mouthwash / Pre-Procedural Rinse on SARS-CoV-2 Load (COVID-19)	https://clinicaltrials.gov/show/NCT04409873
NCT04410510	1100	Caesalpinia extract	P2Et Extract in the Symptomatic Treatment of Subjects With COVID-19	https://clinicaltrials.gov/show/NCT04410510
NCT04407182	1101	Viusid and Asbrip	Safety and Efficacy of Viusid and Asbrip in Hospitalized Patients Infected by SARS-Cov-2 With COVID-19	https://clinicaltrials.gov/show/NCT04407182
NCT04409522	1102	Melatonin	Evaluation of Therapeutic Effects of Melatonin by Inhibition of NLRP3 Inflammasome in COVID19 Patients	https://clinicaltrials.gov/show/NCT04409522
NCT04428268	1103	Losartan	Chloroquine + Losartan Compared to Chloroquine Alone for the Treatment of COVID-19 Pneumonia	https://clinicaltrials.gov/show/NCT04428268
NCT04405310	1104	Convalescent Plasma	Convalescent Plasma of Covid-19 to Treat SARS-COV-2 a Randomized Doble Blind 2 Center Trial	https://clinicaltrials.gov/show/NCT04405310
NCT04415073	1105	Axatilimab	A Phase 2 Study to Evaluate Axatilimab for Hospitalized Patients With Respiratory Involvement Secondary to COVID-19	https://clinicaltrials.gov/show/NCT04415073
NCT04405921	1106	Chloroquine / Azithromycin	Hydroxychloroquine, Azithromycin in the Treatment of Covid-19	https://clinicaltrials.gov/show/NCT04405921
NCT04412772	1107	Tocilizumab	A RCT - Safety & Efficacy of Tocilizumab - Tx of Severe COVID-19: ARCHITECTS	https://clinicaltrials.gov/show/NCT04412772
NCT04407130	1108	Ivermectin / Doxycycline	Efficacy and Safety of Ivermectin and Doxycycline in Combination or IVE Alone in Patients With COVID-19 Infection.	https://clinicaltrials.gov/show/NCT04407130
NCT04405570	1109	EIDD-2801	A Safety, Tolerability and Efficacy of EIDD-2801 to Eliminate Infectious Virus Detection in Persons With COVID-19	https://clinicaltrials.gov/show/NCT04405570
NCT04405739	1110	EIDD-2801	The Effect of EIDD-2801 on Viral Shedding of SARS-CoV-2 (COVID-19)	https://clinicaltrials.gov/show/NCT04405739
NCT04409262	1111	Tocilizumab	A Study to Evaluate the Efficacy and Safety of Remdesivir Plus Tocilizumab Compared With Remdesivir Plus Placebo in Hospitalized Participants With Severe COVID-19 Pneumonia	https://clinicaltrials.gov/show/NCT04409262
NCT04412057	1112	CERC-002	Clinical Trial to Evaluate CERC-002 in Adults With COVID-19 Pneumonia and Acute Lung Injury	https://clinicaltrials.gov/show/NCT04412057
NCT04412252	1113	Tofacitinib	Safety and Efficacy of Tofacitinib in Hospitalized Participants With COVID-19 Pneumonia Who Are Receiving Standard of Care Therapy	https://clinicaltrials.gov/show/NCT04412252
NCT04421027	1114	Baricitinib	A Study of Baricitinib (LY3009104) in Participants With COVID-19	https://clinicaltrials.gov/show/NCT04421027
NCT04425460	1115	Favipiravir	A Multi-center,Randomized,Double-blind,Placebo-controlled,Phase 3 Study Evaluating Favipiravir in Treatment of COVID19	https://clinicaltrials.gov/show/NCT04425460
NCT04429867	1116	Chloroquine	Hydroxychloroquine Use in Hospitalized Patients With COVID-19: Impact on Progression to Severe or Critical Disease	https://clinicaltrials.gov/show/NCT04429867
NCT04432766	1117	BAT2020	A Phase 1 Study to Assess BAT2020 in Hospitalized Patients Infected With SARS-CoV-2 (COVID-19)	https://clinicaltrials.gov/show/NCT04432766
NCT04442230	1118	Nasovax	NasoVAX in Patients With Early Coronavirus Infectious Disease 2019 (COVID-19)	https://clinicaltrials.gov/show/NCT04442230
NCT04428801	1119	Stem cells	Autologous Adipose-derived Stem Cells (AdMSCs) for COVID-19	https://clinicaltrials.gov/show/NCT04428801
NCT04405102	1120	Ozanamid	COVID-19 Ozanimod Intervention Study	https://clinicaltrials.gov/show/NCT04405102
NCT04406389	1121	Enoxaparin / heparin / fondapariniux / argatroban	Anticoagulation in Critically Ill Patients With COVID-19 (The IMPACT Trial)	https://clinicaltrials.gov/show/NCT04406389
NCT04410328	1122	Aggrenox	Aggrenox To Treat Acute Covid-19	https://clinicaltrials.gov/show/NCT04410328
NCT04411433	1123	Favipiravir	Efficacy and Safety of Hydroxychloroquine and Favipiravir in the Treatment of Mild to Moderate COVID-19	https://clinicaltrials.gov/show/NCT04411433
NCT04411680	1124	Sargramostim	Study of Sargramostim in Patients With COVID-19	https://clinicaltrials.gov/show/NCT04411680
NCT04411667	1125	IVIG	Study of Standard of Care Plus Intravenous Immunoglobulin (IVIG) Compared to Standard of Care Alone in the Treatment of COVID-19 Infection	https://clinicaltrials.gov/show/NCT04411667
NCT04412018	1126	Icosapent	An Investigation on the Effects of Icosapent Ethyl (VascepaTM) on Inflammatory Biomarkers in Individuals With COVID-19	https://clinicaltrials.gov/show/NCT04412018
NCT04412291	1127	Tocilizumab	A Study in Patients With COVID-19 and Respiratory Distress Not Requiring Mechanical Ventilation, to Compare Standard-of-care With Anakinra and Tocilizumab Treatment The Immunomodulation-CoV Assessment (ImmCoVA) Study	https://clinicaltrials.gov/show/NCT04412291
NCT04413838	1128	Nivolumab	Efficiency and Security of NIVOLUMAB Therapy in Obese Individuals With COVID-19(COrona VIrus Disease) Infection	https://clinicaltrials.gov/show/NCT04413838
NCT04414631	1129	Conestat	Conestat Alfa in the Prevention of Severe SARS-CoV-2 Infection in Hospitalized Patients With COVID-19	https://clinicaltrials.gov/show/NCT04414631
NCT04419025	1130	N-Acetyl Cysteine	Efficacy of N-Acetylcysteine (NAC) in Preventing COVID-19 From Progressing to Severe Disease	https://clinicaltrials.gov/show/NCT04419025
NCT04415086	1131	Convalescent Plasma	Treatment of Patients With COVID-19 With Convalescent Plasma	https://clinicaltrials.gov/show/NCT04415086
NCT04416399	1132	Budesonide	STerOids in COVID-19 Study	https://clinicaltrials.gov/show/NCT04416399
NCT04421534	1133	Lactoferrin	Utility of Lactoferrin as an Adjunct Therapeutic Agent for COVID-19	https://clinicaltrials.gov/show/NCT04421534
NCT03376854	1134	Neuromuscular blockade and cooling	Pilot RCT of Therapeutic Hypothermia Plus Neuromuscular Blockade in COVID-19 Patients With ARDS	https://clinicaltrials.gov/show/NCT03376854
NCT04410159	1135	Povidone Iodine	Povidone-Iodine Vs Essential Oil Vs Tap Water Gargling For COVID-19 Patients	https://clinicaltrials.gov/show/NCT04410159
NCT04415060	1136	Sevoflurane / isoflurane	SedAting With Volatile Anesthetics Critically Ill COVID-19 Patients in ICU: Effects On Ventilatory Parameters And Survival	https://clinicaltrials.gov/show/NCT04415060
NCT04422678	1137	Imatinib	The Safety & Efficacy of Imatinib for the Treatment of SARS-COV-2 Induced Pneumonia	https://clinicaltrials.gov/show/NCT04422678
NCT04424797	1138	Prone position	Prone Positioning on Admission for Hospitalized COVID-19 Pneumonia Protocol	https://clinicaltrials.gov/show/NCT04424797
NCT04425252	1139	Brequinar	Safety and Anti-coronavirus Response of Suppression of Host Nucleotide Synthesis in Patients With COVID-19	https://clinicaltrials.gov/show/NCT04425252
NCT04425915	1140	Convalescent Plasma	Efficacy of Convalescent Plasma Therapy in Patients With COVID-19	https://clinicaltrials.gov/show/NCT04425915
NCT04429854	1141	Convalescent Plasma	Donated Antibodies Working Against nCoV	https://clinicaltrials.gov/show/NCT04429854
NCT04432987	1142	Pulmozyme	Dornase Alpha for the Treatment of COVID-19	https://clinicaltrials.gov/show/NCT04432987
NCT04433949	1143	Lung Irradiation	Best Supportive Care With or Without Low Dose Whole Lung Radiation Therapy for the Treatment of COVID-19	https://clinicaltrials.gov/show/NCT04433949
NCT04437823	1144	Stem cells	Efficacy of Intravenous Infusions of Stem Cells in the Treatment of COVID-19 Patients	https://clinicaltrials.gov/show/NCT04437823
NCT04438057	1145	Convalescent Plasma	Evaluating the Efficacy of Convalescent Plasma in Symptomatic Outpatients Infected With COVID-19	https://clinicaltrials.gov/show/NCT04438057
NCT04439006	1146	Ibrutinib	Ibrutinib for the Treatment of COVID-19 in Patients Requiring Hospitalization	https://clinicaltrials.gov/show/NCT04439006
NCT04440007	1147	Abivertinib	Study of the Efficacy and Safety of STI-5656 (Abivertinib Maleate) With SOC Versus SOC in Subjects With COVID-19	https://clinicaltrials.gov/show/NCT04440007
NCT04441385	1148	Maraviroc	Study to Evaluate the Efficacy and Safety of Maraviroc in SARS-CoV-2 Infection (COVID-19).	https://clinicaltrials.gov/show/NCT04441385
NCT04441424	1149	Convalescent Plasma	Convalescent Plasma Therapy on Critically-ill Novel Coronavirus (COVID-19) Patients	https://clinicaltrials.gov/show/NCT04441424
NCT04441996	1150	Plasma exchange	Therapeutic Plasma Exchange for COVID-19-associated Hyperviscosity	https://clinicaltrials.gov/show/NCT04441996
NCT04442048	1151	IM-101	Immunization With IMM-101 vs Observation for Prevention of Respiratory and Severe COVID-19 Related Infections in Cancer Patients at Increased Risk of Exposure	https://clinicaltrials.gov/show/NCT04442048
NCT04405843	1152	Ivermectin	Efficacy of Ivermectin in Adult Patients With Early Stages of COVID-19	https://clinicaltrials.gov/show/NCT04405843
NCT04407390	1153	Nicotinamide	Effects of Nicotinamide Riboside on the Clinical Outcome of Covid-19 in the Elderly	https://clinicaltrials.gov/show/NCT04407390
NCT04409509	1154	CSL312	Treatment With CSL312 in Adults With Coronavirus Disease 2019 (COVID-19)	https://clinicaltrials.gov/show/NCT04409509
NCT04412395	1155	Lactoferrin	Clinical Assessment of Oral Lactoferrin as a Safe Antiviral and Immunoregulatory in Treating COVID-19 Disease	https://clinicaltrials.gov/show/NCT04412395
NCT04411446	1156	Vitamin D	Cholecalciferol to Improve the Outcomes of COVID-19 Patients	https://clinicaltrials.gov/show/NCT04411446
NCT04420676	1157	Omnibiotic	Synbiotic Therapy of Gastrointestinal Symptoms During Covid-19 Infection	https://clinicaltrials.gov/show/NCT04420676
NCT04421508	1158	Nitric Oxide	A Study to Assess Pulsed Inhaled Nitric Oxide vs Placebo in Subjects With Mild or Moderate COVID-19	https://clinicaltrials.gov/show/NCT04421508
NCT04421664	1159	Chloroquine	Preemptive Therapy for SARS-Coronavirus-2 (COVID-19 PEP Canada)	https://clinicaltrials.gov/show/NCT04421664
NCT04407689	1160	IL-7	InterLeukin-7 to Improve Clinical Outcomes in Lymphopenic pAtients With COVID-19 Infection FR BL Cohort	https://clinicaltrials.gov/show/NCT04407689
NCT04412668	1161	ATYR1923	Study to Evaluate the Safety and Efficacy of ATYR1923 In Patients With Severe Pneumonia Related to COVID-19	https://clinicaltrials.gov/show/NCT04412668
NCT04414618	1162	Opaganib	A Study of Opaganib in Coronavirus Disease 2019 Pneumonia	https://clinicaltrials.gov/show/NCT04414618
NCT04417257	1163	LAU-7b	Study of LAU-7b for the Treatment of COVID-19 Disease in Adults	https://clinicaltrials.gov/show/NCT04417257
NCT04426695	1164	REGN10933	Safety, Tolerability, and Efficacy of Anti-Spike (S) SARS-CoV-2 Monoclonal Antibodies for Hospitalized Adult Patients With COVID-19	https://clinicaltrials.gov/show/NCT04426695
NCT04429334	1165	Nangibotide	Safety, Tolerability and Efficacy of Nangibotide in Mechanically Ventilated Patients With COVID-19 and Features of Systemic Inflammation	https://clinicaltrials.gov/show/NCT04429334
NCT04429711	1166	Ivermectin	Ivermectin vs. Placebo for the Treatment of Patients With Mild to Moderate COVID-19	https://clinicaltrials.gov/show/NCT04429711
NCT04432298	1167	Pamrevlumab	Phase 2 Study of the Efficacy and Safety of Intravenous Pamrevlumab, in Hospitalized Patients With Acute COVID-19 Disease	https://clinicaltrials.gov/show/NCT04432298
NCT04433546	1168	PB1046	PB1046, a Long-acting, Sustained Release Human VIP Analogue, Intended to Provide Clinical Improvement to Hospitalized COVID-19 Patients at High Risk for Rapid Clinical Deterioration and Acute Respiratory Distress Syndrome (ARDS).	https://clinicaltrials.gov/show/NCT04433546

Registry entry ID	Included ID	Intervention	Title	Webpage
NCT04435015	1169	Camostat mesilate	The Utility of Camostat Mesylate in Patients With COVID-19 Associated Coagulopathy (CAC) and Cardiovascular Complications	https://clinicaltrials.gov/show/NCT04435015
NCT04435184	1170	Crizanlizumab	Crizanlizumab for Treating COVID-19 Vasculopathy	https://clinicaltrials.gov/show/NCT04435184
NCT04436458	1171	Niclosamide	Niclosamide In Moderate COVID-19	https://clinicaltrials.gov/show/NCT04436458
NCT04439071	1172	PTC299	A Study to Evaluate Efficacy and Safety of PTC299 in Hospitalized Participants With Coronavirus (COVID-19)	https://clinicaltrials.gov/show/NCT04439071
NCT04441398	1173	Nitazoxamide	Efficacy and Safety of Nitazoxanide 600 mg to Treat Mild Ambulatory COVID-19 Patients	https://clinicaltrials.gov/show/NCT04441398
NCT04407507	1174	Ivermectin	Efficacy, Safety and Tolerability of Ivermectin in Subjects Infected With SARS-CoV-2 With or Without Symptoms	https://clinicaltrials.gov/show/NCT04407507
NCT04408235	1175	Heparin	High Versus Low LMWH Dosages in Hospitalized Patients With Severe COVID-19 Pneumonia and Coagulopathy	https://clinicaltrials.gov/show/NCT04408235
NCT04420364	1176	Azathioprine	Maintenance Versus Reduction of Immunosuppression for Renal Transplant Patients Hospitalized With COVID-19 Disease	https://clinicaltrials.gov/show/NCT04420364
NCT04424134	1177	Bromhexine / spironolactone	BromhexIne And Spironolactone For Coron?VirUs Infection Requiring HospiTalization	https://clinicaltrials.gov/show/NCT04424134
NCT03963622	1178	Ventilator settings	Careful Ventilation in COVID-19 -Induced Acute Respiratory Distress Syndrome	https://clinicaltrials.gov/show/NCT03963622
NCT04420247	1179	Chloroquine	Efficacy of Chloroquine or Hydroxychloroquine in Treating Pneumonia Caused by SARS-Cov-2 - COVID-19	https://clinicaltrials.gov/show/NCT04420247
NCT04425837	1180	Convalescent Plasma	Effectiveness and Safety of Convalescent Plasma in Patients With High-risk COVID-19	https://clinicaltrials.gov/show/NCT04425837
NCT04435353	1181	Vibroacoustics	Assessment of the Effectiveness of Vibroacoustic Therapy for Respiratory Failure Caused by COVID 19	https://clinicaltrials.gov/show/NCT04435353
NCT04435587	1182	Chloroquine / ritonavir / Ivermectin	Ivermectin vs Combined Hydroxychloroquine and Antiretroviral Drugs (ART) Among Asymptomatic COVID-19	https://clinicaltrials.gov/show/NCT04435587
NCT04412356	1183	Tracheotomy	Timing of Tracheotomy in Covid-19 Patients	https://clinicaltrials.gov/show/NCT04412356
NCT04409886	1184	Hyperbaric oxygen	Hyperbaric Oxygen Therapy in Non-ventilated COVID-19 Patients	https://clinicaltrials.gov/show/NCT04409886
NCT04420299	1185	Bemiparin	Clinical Trial on the Efficacy and Safety of Bemiparin in Patients Hospitalized Because of COVID-19	https://clinicaltrials.gov/show/NCT04420299
NCT04426344	1186	Warming	Core Warming of COVID-19 Patients	https://clinicaltrials.gov/show/NCT04426344
NCT04415151	1187	Tofacitinib	Tofacitinib for Treatment of Moderate COVID-19	https://clinicaltrials.gov/show/NCT04415151
NCT04193878	1188	Budesonide / Formoterol	ARrest RESpiraTory Failure From PNEUMONIA	https://clinicaltrials.gov/show/NCT04193878
NCT04410354	1189	Merimepodib	Study of Merimepodib in Combination With Remdesivir in Adult Patients With Advanced COVID-19	https://clinicaltrials.gov/show/NCT04410354
NCT04421404	1190	Convalescent Plasma	Effects of COVID-19 Convalescent Plasma (CCP) on Coronavirus-associated Complications in Hospitalized Patients	https://clinicaltrials.gov/show/NCT04421404
NCT04429555	1191	Ibudilast	Efficacy, Safety, Tolerability, and Biomarkers of MN-166 (Ibudilast) in Patients Hospitalized With COVID-19 and at Risk for ARDS	https://clinicaltrials.gov/show/NCT04429555
NCT04429763	1192	Stem cells	Safety and Efficacy of Mesenchymal Stem Cells in the Management of Severe COVID-19 Pneumonia	https://clinicaltrials.gov/show/NCT04429763
NCT04433078	1193	Doxycycline	RepurpoSing Old Drugs TO SuppRess a Modern Threat: COVID-19 STORM	https://clinicaltrials.gov/show/NCT04433078
NCT04428021	1194	Convalescent Plasma	Standard or Convalescent Plasma in Patients With Recent Onset of COVID-19 Respiratory Failure	https://clinicaltrials.gov/show/NCT04428021
NCT04433013	1195	Lianhua Qingwen	A Randomized Controlled Trial Assessing the Efficacy of Lianhua Qingwen as an Adjuvant Treatment in Patients With Mild Symptoms of COVID-19	https://clinicaltrials.gov/show/NCT04433013
NCT04431466	1196	Ivermectin	A Study to Compare the Efficacy and Safety of Different Doses of Ivermectin for COVID-19	https://clinicaltrials.gov/show/NCT04431466
NCT04442191	1197	Convalescent Plasma	Convalescent Plasma as a Possible Treatment for COVID-19	https://clinicaltrials.gov/show/NCT04442191
NCT04405076	1198	mRNA-1273 vaccine	Dose-Confirmation Study to Evaluate the Safety, Reactogenicity, and Immunogenicity of mRNA-1273 COVID-19 Vaccine in Adults Aged 18 Years and Older	https://clinicaltrials.gov/show/NCT04405076
NCT04405908	1199	SCB-2019 vaccine	SCB-2019 as COVID-19 Vaccine	https://clinicaltrials.gov/show/NCT04405908
NCT04425733	1200	MK-5475	MK-5475 in Participants With Hypoxemia Due to COVID-19 Pneumonia (MK-5475-009)	https://clinicaltrials.gov/show/NCT04425733
NCT04427501	1201	LY-CoV555	A Study of LY3819253 (LY-CoV555) in Participants With Early Mild to Moderate COVID-19 Illness	https://clinicaltrials.gov/show/NCT04427501
NCT04434248	1202	Favipiravir	An Adaptive Study of Favipiravir Compared to Standard of Care in Hospitalized Patients With COVID-19	https://clinicaltrials.gov/show/NCT04434248
NCT04438850	1203	Ivermectin	COVidIVERmectin: Ivermectin for Treatment of Covid-19	https://clinicaltrials.gov/show/NCT04438850
NCT04441918	1204	JS016 monoclonal antibody	Tolerability,Safety,Pharmacokinetic Profile and Immunogenicity of a Recombinant Humanized Anti-SARS-CoV-2 Monoclonal Antibody (JS016) for Injection in Chinese Health Subjects	https://clinicaltrials.gov/show/NCT04441918
EUCTR2020-001376-15-DE	1205	VPM1002 vaccine	A study to investigate whether vaccination with VPM1002 can reduce the sick days of healthcare professionals during the SARS-CoV-2 pandemic	https://www.clinicaltrialsregister.eu/ctr-search/search?query=eudract_number:2020-001376-15
EUCTR2020-001937-11-GB	1206	Pulmozyme	The COVASE study: Investigation of an approved nebulised human Dnase enzyme (Pulmozyme) to reduce hyperinflammation in hospitalised people with COVID-19	https://www.clinicaltrialsregister.eu/ctr-search/search?query=eudract_number:2020-001937-11
EUCTR2020-001960-28-ES	1207	Vitamin D	Efficacy of vitamin D treatment in patients diagnosed with pneumonia who require hospital admission and have vitamin D deficiency and a positive diagnosis for SARS-Cov-2 (COVID-19)	https://www.clinicaltrialsregister.eu/ctr-search/search?query=eudract_number:2020-001960-28
EUCTR2020-002111-22-PL	1208	BCG vaccine	Study evaluating the impact of BCG vaccination on the incidence and course of SARS-CoV-2 infection among health care workers in Poland during the COVID-19 pandemic	https://www.clinicaltrialsregister.eu/ctr-search/search?query=eudract_number:2020-002111-22
EUCTR2020-002091-12-BG	1209	Ivermectin	Study of ivermectin against COVID-19	https://www.clinicaltrialsregister.eu/ctr-search/search?query=eudract_number:2020-002091-12
EUCTR2020-001675-33-DE	1210	VPM1002 vaccine	A study to investigate whether vaccination with VPM1002 can reduce elderly people's days with severe respiratory infectious diseases at hospital and/or at home	https://www.clinicaltrialsregister.eu/ctr-search/search?query=eudract_number:2020-001675-33
EUCTR2020-002293-28-SE	1211	Omega-3	Treatment with Omega-3 polyunsaturated fatty acids in COVID-19 patients.	https://www.clinicaltrialsregister.eu/ctr-search/search?query=eudract_number:2020-002293-28
EUCTR2020-002233-15-DE	1212	Niclosamide / Camostat	A study to investigate the safety and efficacy of two approved drugs, niclosamide and camostat, administered alone and in combination and compared to placebo in COVID-19 patients	https://www.clinicaltrialsregister.eu/ctr-search/search?query=eudract_number:2020-002233-15
EUCTR2020-001739-28-BE	1213	Aprotinin	A randomized, open-label, adaptive, proof-of-concept clinical trial of modulation of host thromboinflammatory response in patients with COVID-19.	https://www.clinicaltrialsregister.eu/ctr-search/search?query=eudract_number:2020-001739-28
EUCTR2020-001636-95-GB	1214	Anakinra	Does subcutaneously(SC) administered of anakinra achieve good penetration in blood and result in reduce inflammation in patients with SARS-CoV-19?	https://www.clinicaltrialsregister.eu/ctr-search/search?query=eudract_number:2020-001636-95
EUCTR2020-001841-38-ES	1215	Colchicine	Colchicine for the Treatment of Hyperinflammation associated with Pneumonia due to COVID-19	https://www.clinicaltrialsregister.eu/ctr-search/search?query=eudract_number:2020-001841-38
EUCTR2020-001605-23-ES	1216	Chloroquine / azithromycin / Lopinavir/ Ritonavir	Effectiveness of the combined treatment with hydroxycloroquine and azithromycin vs lopinavir/ritonavir + hydroxycloroquine in hospitalized patients with confirmed COVID-19 infection	https://www.clinicaltrialsregister.eu/ctr-search/search?query=eudract_number:2020-001605-23
EUCTR2020-002312-43-ES	1217	Vitamin D	Clinical trial, randomized, open-label, to evaluate the efficacy of high-dose vitamin D in patients with COVID-19 pneumonia	https://www.clinicaltrialsregister.eu/ctr-search/search?query=eudract_number:2020-002312-43
EUCTR2020-000982-18-SE	1218	Chloroquine / Remdesivir	A clinical study to evaluate the efficacy of different anti-viral drugs in SARS-CoV-2 infected patients (COVID-19).	https://www.clinicaltrialsregister.eu/ctr-search/search?query=eudract_number:2020-000982-18
EUCTR2020-001671-32-ES	1219	BDB-001	A clinical trial to investigate the efficacy and safety of a new compound for the treatment of COVID-19, called BDB-001. Patients in the trial will be randomly allocated to either the new treatment or the conventional treatment and will be aware of which treatment they are receiving	https://www.clinicaltrialsregister.eu/ctr-search/search?query=eudract_number:2020-001671-32
EUCTR2020-001904-41-GB	1220	Favipiravir	A study comparing favipiravir versus standard of care management as an early treatment arm in COVID-19 Hospitalized patients	https://www.clinicaltrialsregister.eu/ctr-search/search?query=eudract_number:2020-001904-41
EUCTR2020-002120-37-GB	1221	Sodium Nitrite	An open-label, adaptive randomized, controlled multicenter study to evaluate the efficacy and safety of RESP301+SOC vs SOC in hospitalized participants with COVID-19 requiring supplemental oxygen	https://www.clinicaltrialsregister.eu/ctr-search/search?query=eudract_number:2020-002120-37
EUCTR2020-002037-15-ES	1222	Sarilumab	Multicenter, randomized, open-label study to evaluate the efficacy and safety of SOC + Sarilumab versus Standard of Care for the Early Treatment of COVID-19-pneumonia in Hospitalized Patients	https://www.clinicaltrialsregister.eu/ctr-search/search?query=eudract_number:2020-002037-15
EUCTR2020-001849-39-SE	1223	Pulmozyme	Treatment of COVID-19 with the inhalation drug pulmozyme	https://www.clinicaltrialsregister.eu/ctr-search/search?query=eudract_number:2020-001849-39
PACTR202006899597082	1224	Artesunate	Efficacy of a new COVID-19 treatment	https://pactr.samrc.ac.za/TrialDisplay.aspx?TrialID=11103
ISRCTN33578935	1225	Stem cells	To study the treatment of COVID-19 with severe viral pneumonia by using purified stem cell exosomes	http://isrctn.com/ISRCTN33578935
ISRCTN54917435	1226	Prone position	Could prone positioning increase lung function for patients with severe COVID-19 infection?	http://isrctn.com/ISRCTN54917435
ChiCTR2000033286	1227	Baicalein	A Randomized, Double-blind, Placebo-controlled trial for determination of the Efficacy and Safety of Baicalein in Hospitalized Adult Mild and Moderate Patients of novel coronavirus pneumonia (COVID-19)	http://www.chictr.org.cn/showproj.aspx?proj=54176
ChiCTR2000033627	1228	Ivermectin	In vivo use of ivermectin (IVR) for treatment for corona virus infected patients: a randomized controlled trial	http://www.chictr.org.cn/showproj.aspx?proj=54707
ChiCTR2000033774	1229	Bromhexine	Evaluating the Efficacy and Safety of Bromhexine Hydrochloride Tablets in Treating Pediatric novel coronavirus pneumonia (COVID-19)	http://www.chictr.org.cn/showproj.aspx?proj=55097
PACTR202005622389003	1230	Chloroquine / Azithromycin / Zinc	Hydroxychloroquine, Azithromycine and Zinc for the treatment of SARS-Cov2 infection in Senegal. COVID-19	https://pactr.samrc.ac.za/TrialDisplay.aspx?TrialID=11053
ACTRN12620000674932	1231	SARS-CoV-2 Sclamp Protein Subunit Vaccine	An interventional study to evaluate the safety and immune response of a vaccine against Severe Acute Respiratory Syndrome coronavirus 2 (SARS-CoV-2, the virus that causes COVID-19 infection) when given to healthy adult participants.	https://anzctr.org.au/ACTRN12620000674932.aspx
ACTRN12620000676910	1232	Stem cells	Amnion cells for COVID-19 related respiratory failure	https://anzctr.org.au/ACTRN12620000676910.aspx
ISRCTN17072692	1233	Vaccine LNP-nCoVsaRNA	Clinical trial to assess the safety of a coronavirus vaccine in healthy men and women	http://isrctn.com/ISRCTN17072692
ISRCTN40302986	1234	Ivermectin	Does ivermectin cure and/or prevent COVID-19?	http://isrctn.com/ISRCTN40302986
IRCT20200509047373N1	1235	Dinvi	Evaluation effect of Dinvl for Covid19 infection	http://en.irct.ir/trial/48001
IRCT20170731035423N2	1236	BCc1	Effect of the combination of BCc1 & Hep-S on hospitalized COVID-19 patients	http://en.irct.ir/trial/48049
IRCT20200516047462N1	1237	Methanolic extract	Intervention	http://en.irct.ir/trial/48141
IRCT20190810044500N5	1238	Colchicine	Effect of colchicine in treatment of COVID-19	http://en.irct.ir/trial/46867
IRCT20200511047396N1	1239	INF-Beta	Efficacy evaluation of inhalation therapy (nasal spray) of Interferon Beta-1a in Mild to Moderate cases of Covid-19 at Baqiyatallal Hospital	http://en.irct.ir/trial/48048
IRCT20200506047323N1	1240	Ginger	The effects of Ginger in treatment COVID-19	http://en.irct.ir/trial/48114
IRCT20090609002017N35	1241	Boron compounds	Assessment of boron-containing compounds and Oleoylethanolamid supplementation in the treatment of COVID-19	http://en.irct.ir/trial/48058
IRCT20190727044343N3	1242	ANIF-1	ANIF1 effect on COVID-19	http://en.irct.ir/trial/47062
IRCT20200411047016N1	1243	Stopcivir	The effect of Stopcivir syrup in patients with COVID-19	http://en.irct.ir/trial/47044
IRCT20200405046960N2	1244	Kelofan	Efficacy of Kelofan syrup on COVID-19	http://en.irct.ir/trial/48126
IRCT20200516047468N1	1245	Vitamin C	Investigation of the effect of intravenous vitamin C on definite patients with coronavirus pneumonia	http://en.irct.ir/trial/48176
IRCT20120215009014N355	1246	N-Acetyl Cysteine	Evaluating the effect of intravenous N-acetyl cysteine versus placebo in the treatment of patients with mild and moderate acute respiratory distress syndrome caused by COVID-19	http://en.irct.ir/trial/48039
IRCT20160126026217N4	1247	Prone position	Investigation of the effects of prone position on cardiac and respiratory status in patients with Coronavirus	http://en.irct.ir/trial/48403
IRCT20151227025726N17	1248	Dexamethasone	Evolution of the efficacy and safety of Dexamethasone administration in COVID-19	http://en.irct.ir/trial/48153
IRCT20151227025726N15	1249	Umifenovir	Evaluating the effects of Umifenovir (Arbidol) in COVID-19	http://en.irct.ir/trial/48018
IRCT20151227025726N18	1250	Pentaglobin	Pentaglobin effect in critically ill patients with COVID-19	http://en.irct.ir/trial/48312
IRCT20081011001323N25	1251	Zingiber	Efficacy of extract of of Zingiber cinnamomum and zataria multiflora in covid-19 disease	http://en.irct.ir/trial/47935
IRCT20180923041093N6	1252	Barley product	The efficacy of barely-based traditional remedy on COVID-19	http://en.irct.ir/trial/47974
IRCT20200506047323N2	1253	Licorice	Effects of Licorice in treatment of COVID-19	http://en.irct.ir/trial/47990
IRCT20081019001369N6	1254	Trans sodium crocetinate	Clinical trial of trans sodium crocetinate against COVID-19	http://en.irct.ir/trial/47188
IRCT20190418043307N1	1255	Selenium	Effect of selenium on covid-19 disease	http://en.irct.ir/trial/47209
IRCT20120703010178N20	1256	Anakinra	effect of anakinra in treatment of COVID-19	http://en.irct.ir/trial/48702
IRCT20200405046951N1	1257	Vitamin A	Vitamin A and COVID-19	http://en.irct.ir/trial/47722
IRCT20151226025699N3	1258	Omega-3	The effect of omega-3 supplementation in COVID-19	http://en.irct.ir/trial/48213

Registry entry ID	Included ID	Intervention	Title	Webpage
IRCT20161206031256N3	1259	INF-Alpha	Using interferon to treat COVID-19	http://en.irct.ir/trial/48329
IRCT20200503047280N1	1260	Herbal medicine	Effect of Laris-Teucrium Polium L.and Laris-Hyssop combined herbal medicine on the prevention of covid-19 disease	http://en.irct.ir/trial/47704
IRCT20200518047497N1	1261	Herbal medicine	Effect of Laris - Hyssop combined herbal distilled on the improving clinical and paraclinical symptoms in patients with COVID-19	http://en.irct.ir/trial/48289
IRCT20200518047497N2	1262	Dinvi	Effect of Dinvl on improving of clinical and paraclinical symptoms in patients with COVID-19	http://en.irct.ir/trial/48644
IRCT20200501047259N1	1263	IVIG	IVIG and COVID19	http://en.irct.ir/trial/47609
JPRN-jRCT2031200035	1264	Remdesivir	A Multicenter, Adaptive, Randomized Blinded Controlled Trial of the Safety and Efficacy of Investigational Therapeutics for the Treatment of COVID-19 in Hospitalized Adults	https://jrct.niph.go.jp/latest-detail/jRCT2031200035
JPRN-jRCTs061200002	1265	Teprenone	Teprenone for preventing aggravation of COVID-19	https://jrct.niph.go.jp/latest-detail/jRCTs061200002
DRKS00022203	1266	Sofosbuvir/Daclatasvir	Efficacy & Safety of Sofosbuvir/Daclatasvir treatment in COVID-19: A randomized, controlled study	https://www.drks.de/drks_web/navigate.do?navigationId=trial.HTML&TRIAL_ID=DRKS00022203
NCT04443725	1267	Chloroquine / Sofosbuvir / daclatasvir	Efficacy and Safety of Anti HCV Drugs in the Treatment of COVID-19	https://ClinicalTrials.gov/show/NCT04443725
NCT04444271	1268	Stem cells	Mesenchymal Stem Cell Infusion for COVID-19 Infection	https://ClinicalTrials.gov/show/NCT04444271
NCT04443881	1269	Anakinra	Clinical Trial of the Use of Anakinra in Cytokine Storm Syndrome Secondary to Covid-19 (ANA-COVID-GEAS)	https://ClinicalTrials.gov/show/NCT04443881
NCT04424056	1270	Tocilizumab / Anakinra / Ruxolitinib	A Trial Using ANAKINRA, TOCILIZUMAB Alone or in Association With RUXOLITINIB in Severe Stage 2b and 3 of COVID19-associated Disease	https://ClinicalTrials.gov/show/NCT04424056
NCT04414241	1271	Chloroquine	Hydroxychloroquine to Prevent SARS-CoV-2 Infection	https://ClinicalTrials.gov/show/NCT04414241
NCT04443673	1272	Glycine	Glycine Supplement for Severe COVID-19	https://ClinicalTrials.gov/show/NCT04443673
NCT04388514	1273	ozone autohemotherapy	Blood Ozonization in Patients With SARS-CoV-2 Respiratory Failure	https://ClinicalTrials.gov/show/NCT04388514
NCT04400019	1274	Chloroquine	Prevention of COVID19 Infection in Nursing Homes by Chemoprophylaxis With Hydroxychloroquine (PREVICHARM)	https://ClinicalTrials.gov/show/NCT04400019
NCT04443868	1275	Nitric Oxide	Nitric Oxide Releasing Solution to Treat and Prevent Exacerbation of Mild COVID-19 Infection	https://ClinicalTrials.gov/show/NCT04443868
NCT04444700	1276	Enoxaparin	A Pragmatic Randomized Controlled Trial of Therapeutic Anticoagulation Versus Standard Care as a Rapid Response to COVID-19 Pandemic	https://ClinicalTrials.gov/show/NCT04444700
NCT04383717	1277	Levamisole / Isoprinosine / azithromycin / Chloroquine	Levamisole and Isoprinosine in the Treatment of COVID19: A Proposed Therapeutic Trial	https://ClinicalTrials.gov/show/NCT04383717
NCT04444674	1278	Vaccine ChAdOx1	COVID-19 Vaccine (ChAdOx1 nCoV-19) Trial in South African Adults With and Without HIV-infection	https://ClinicalTrials.gov/show/NCT04444674
NCT04433988	1279	Pentoxifylline	Efficacy of Pentoxifylline as Add on Therapy in COVID19 Patients	https://ClinicalTrials.gov/show/NCT04433988
NCT04385836	1280	Alpha-1-Antitrypsin Inhalation	Trial of Alpha One Antitrypsin Inhalation in Treating Patient With Severe Acute Respiratory Syndrome Coronavirus 2 (SARS-CoV-2)	https://ClinicalTrials.gov/show/NCT04385836
NCT04442958	1281	Convalescent Plasma	Effectiveness of Convalescent Immune Plasma Therapy	https://ClinicalTrials.gov/show/NCT04442958
eudract002482-34	1282	Octagam		https://www.clinicaltrialsregister.eu/ctr-search/trial/2020-002482-34/3rd
eudract002060-31	1283	Ritonavir/lopinavir / Remdesivir		https://www.clinicaltrialsregister.eu/ctr-search/trial/2020-002060-31/CZ
eudract002668-29	1284	Plasma exchange		https://www.clinicaltrialsregister.eu/ctr-search/trial/2020-002668-29/GB
eudract002110-41	1285	Camostat mesilate		https://www.clinicaltrialsregister.eu/ctr-search/trial/2020-002110-41/GB
IRCT20200504047298N1	1286	Atazanavir/Ritonavir/lopinavir	Evaluation of Atazanavir/Ritonavir effect on COViD-19 patients treatment in comparison with Lopinavir / Ritonavir (/trial/48640) Lopinavir/Ritanavir on respiratory involvement including Pneumonia and COViD-19 induced ARDS comparison of (...) : Clinic and Para clinic findings in	https://www.irct.ir/trial/48640
IRCT20200504047298N2	1287	Opontia cactus	Effect of Opontia Cactus extract on improving pulmonary and clinical function in patients with Covid-19	https://www.irct.ir/trial/47675
IRCT20200330046899N1	1288	Herbal medicine	Evaluating the efficacy of herbal capsule in COVID-19 patients in a clinical trial	https://www.irct.ir/trial/48365
IRCT20180425039414N3	1289	Vitamin C and E	The effect of vitamin C and E in the treatment and clinical course of patients with SARS-cov2 (COVID-19)	https://www.irct.ir/trial/48083
IRCT20171105037262N4	1290	Adalimumab	Evaluation of adalimumab effect on clinical symptoms including respiratory distress, oxygen saturation and lung involvement of patients with COVID-19	https://www.irct.ir/trial/47696
IRCT20200317046797N6	1291	Edaravone	Effect of edaravone on clinical improvement and outcome of patients with respiratory distress syndrome caused by COVID-19	https://www.irct.ir/trial/48500
IRCT20200607047675N1	1292	Ferula assa-foetida gum	A prospective randomized controlled trial comparing the effectiveness of traditional medicine combination based on the Ferula assa-foetida gum - Tragacanth with moderate to severe Coronavirus disease (COVID-19) compared to standard treatment	https://www.irct.ir/trial/48700
IRCT20200611047735N1	1293	Nanomaterial	Evaluation of the effect of nano micelles containing curcumin (Sina Ccurcumin) as a therapeutic supplement in patients with COVID-19 and investigating of immune responses balance changes following treatment: A randomized double blind clinical trial.	https://www.irct.ir/trial/48843
NL8633	1294	Convalescent Plasma	A randomized, double blinded clinical trial of convalescent plasma compared to standard plasma for treatment of hospitalized non-ICU patients with COVID-19 infections	https://www.trialregister.nl/trial/8633
PACTR202006537901307	1295	Chloroquine / Lopinavir/ Ritonavir	An open-label, multicentre, randomised, adaptive platform trial of the safety and efficacy of several therapies, including antiviral therapies, versus control in mild/moderate cases of COVID-19	https://pactr.samrc.ac.za/TrialDisplay.aspx?TrialID=12150
PACTR202006760881890	1296	Convalescent Plasma	A Multi-Center, Randomized, Double-Blind, Placebo- Controlled Clinical Trial of the Safety and Efficacy of Convalescent Plasma for the Treatment of COVID-19 in Hospitalized Patients in Lagos State	https://pactr.samrc.ac.za/TrialDisplay.aspx?TrialID=12168
CTRI/2020/05/025209	1297	Convalescent Plasma	A clinical trial for validating therapeutic efficacy of convalescent plasma in severe COVID-19 disease	http://ctri.nic.in/Clinicaltrials/pmaindet2.php?trialid=43251&EncHid=&userName=covid
CTRI/2020/05/025215	1298	Siddha, Kabasura, vitamin c, zinc	Effectiveness of Siddha medicine, Kabasura kudineer and vitamin c-zinc supplementation in the management of Mild COVID 19 patients	http://ctri.nic.in/Clinicaltrials/pmaindet2.php?trialid=43769&EncHid=&userName=covid
CTRI/2020/05/025483	1299	Ayurveda	Use of Clevira in COVID19 patients	http://ctri.nic.in/Clinicaltrials/pmaindet2.php?trialid=44041&EncHid=&userName=covid
CTRI/2020/06/025796	1300	Ayurveda	Effect of aragwadhadi kwath (kadha in Hindi) in treatment of mild corona virus disease	http://ctri.nic.in/Clinicaltrials/pmaindet2.php?trialid=44181&EncHid=&userName=covid
CTRI/2020/06/025762	1301	Ayurveda	An Open-Label Randomized Controlled, Proof-of-Concept (PoC) Study to Evaluate the Safety and Efficacy of selected Siddha formulations in patients diagnosed with COVID-19	http://ctri.nic.in/Clinicaltrials/pmaindet2.php?trialid=44259&EncHid=&userName=covid
CTRI/2020/06/025855	1302	Ayurveda	Effect of AYUSH 64 in COVID 19	http://ctri.nic.in/Clinicaltrials/pmaindet2.php?trialid=44386&EncHid=&userName=covid
CTRI/2020/06/025957	1303	Umifenovir	A Clinical Study on Favipiravir and Umifenovir Compared to Favipiravir alone in Hospitalized Patients with Moderate COVID-19	http://ctri.nic.in/Clinicaltrials/pmaindet2.php?trialid=44709&EncHid=&userName=covid
